# Systemic Metabolic Signatures of Oral Diseases

**DOI:** 10.1177/00220345231203562

**Published:** 2023-11-15

**Authors:** A. Salminen, A.M. Määttä, P. Mäntylä, J. Leskelä, M. Pietiäinen, K. Buhlin, A.L. Suominen, S. Paju, W. Sattler, J. Sinisalo, P.J. Pussinen

**Affiliations:** 1 Oral and Maxillofacial diseases, University of Helsinki and Helsinki University Hospital, Helsinki, Finland; 2 Institute of Dentistry, University of Eastern Finland, Kuopio, Finland; 3 Odontology Education, Kuopio University Hospital, Kuopio, Finland; 4 Division of Periodontology, Department of Dental Medicine, Karolinska Institutet, Huddinge, Sweden; 5 Department of Public Health and Welfare, National Institute for Health and Welfare, Helsinki, Finland; 6 Division of Molecular Biology and Biochemistry, Gottfried Schatz Research Center, Medical University of Graz, Graz, Austria; 7 HUCH Heart and Lung Center, Helsinki University Hospital and University of Helsinki, Finland

**Keywords:** metabolomics, inflammation, periodontitis, dental caries, endodontics, root canal treatment

## Abstract

Systemic metabolic signatures of oral diseases have been rarely investigated, and prospective studies do not exist. We analyzed whether signs of current or past infectious/inflammatory oral diseases are associated with circulating metabolites. Two study populations were included: the population-based Health-2000 (*n* = 6,229) and Parogene (*n* = 452), a cohort of patients with an indication to coronary angiography. Health-2000 participants (*n* = 4,116) provided follow-up serum samples 11 y after the baseline. Serum concentrations of 157 metabolites were determined with a nuclear magnetic resonance spectroscopy-based method. The associations between oral parameters and metabolite concentrations were analyzed using linear regression models adjusted for age, sex, number of teeth, smoking, presence of diabetes, and education (in Health-2000 only). The number of decayed teeth presented positive associations with low-density lipoprotein diameter and the concentrations of pyruvate and citrate. Negative associations were found between caries and the unsaturation degree of fatty acids (FA) and relative proportions of docosahexaenoic and omega-3 FAs. The number of root canal fillings was positively associated with very low-density lipoprotein parameters, such as diameter, cholesterol, triglycerides, and number of particles. Deepened periodontal pockets were positively associated with concentrations of cholesterol, triglycerides, pyruvate, leucine, valine, phenylalanine, and glycoprotein acetyls and negatively associated with high-density lipoprotein (HDL) diameter, FA unsaturation degree, and relative proportions of omega-6 and polyunsaturated FAs. Bleeding on probing (BOP) was associated with increased concentrations of triglycerides and glycoprotein acetyls, as well as decreased proportions of omega-3 and omega-6 FAs. Caries at baseline predicted alterations in apolipoprotein B–containing lipoproteins and HDL-related metabolites in the follow-up, and both caries and BOP were associated with changes in HDL-related metabolites and omega-3 FAs in the follow-up. Signs of current or past infectious/inflammatory oral diseases, especially periodontitis, were associated with metabolic profiles typical for inflammation. Oral diseases may represent a modifiable risk factor for systemic chronic inflammation and thus cardiometabolic disorders.

## Introduction

Metabolomics refers to the large-scale study of small molecules called metabolites, which collectively form the metabolic phenotype of an individual. Diet, clinical measurements such as blood pressure, body mass index, use of medications, and the composition of gut microbiome predicted 76% of the profiled serum metabolites (Bar et al. 2020). Considering the importance of oral health in cardiovascular diseases (CVDs), its contribution to metabolic profiles in serum is a relevant target for investigation. Although the mechanisms behind the association of oral inflammations and CVD are partially unknown, systemic inflammation and endotoxemia are among the most studied (Pietiäinen et al. 2018), and they both induce multiple metabolic alterations (Khovidhunkit et al. 2004; Määttä et al. 2021; Jiménez et al. 2022).

A widely used high-throughput nuclear magnetic resonance (NMR) platform provides a broad range of clinically and analytically validated metabolic biomarker concentrations. The biomarkers represent cell metabolism and pathways, such as fatty acid balance, glycolysis, ketone bodies, inflammation, lipoprotein class/subclass profiles, and other lipids (Würtz et al. 2017). They have been used extensively to predict disease events (Würtz et al. 2015) and all-cause morbidity (Fischer et al. 2014), measure chronic inflammation (Ritchie et al. 2015), and identify metabolic risk factors, such as insulin resistance (Wang et al. 2012).

The NMR platform provides detailed lipoprotein subclass profiling. All classes—namely, very low-density lipoprotein (VLDL), intermediate-density lipoprotein (IDL), low-density lipoprotein (LDL), and high-density lipoprotein (HDL)—are divided into subclasses from XXL to XS based on particle size. The compositions comprise the particle core containing cholesterol esters and triglycerides, as well as the surface containing free cholesterol, phospholipids, and apolipoproteins. Clinical laboratories traditionally produce only total cholesterol and lipid determinations. For example, plasma triglyceride or cholesterol concentration refers to the sum of these lipids, making no differentiation between carrier lipoprotein classes, sizes, or overall composition. Detailed metabolic profiling extends the knowledge of disease mechanisms and aids to identify modifiable risk factors. For example, trials investigating predictors of future CVD events imply that inflammation is one of the most important sources of the “residual cardiovascular risk” requiring adjunctive treatments (Ridker et al. 2023).

There are only few previous studies concerning systemic metabolic profiling of oral inflammations (Barnes et al. 2014; Chen et al. 2018), and further investigations using untargeted, modern methods are warranted. In the cross-sectional part of our study, we analyzed whether signs of current or past infectious/inflammatory oral diseases are associated with circulating metabolites, including concentrations, compositions, and subclass distributions of lipoprotein particles, as well as levels of fatty acids (FAs), amino acids, ketone bodies, metabolic substrates, and various other metabolic markers determined by NMR spectroscopy-based methods in 2 cohorts. In the longitudinal part of our study, we analyzed whether signs of oral diseases at baseline are associated with metabolic changes during 11 y of follow-up.

## Materials and Methods

### Study Populations

The Parogene study and the Health-2000 survey are described in detail in the Appendix. The oral parameters registered included radiographically determined caries lesions, periapical infections, the number and quality of root canal fillings (RCFs), and alveolar bone loss (ABL), as well as clinically determined number of teeth with probing pocket depths (PPDs) ≥4 mm and bleeding on probing (BOP). The periodontal inflammatory burden index (PIBI) was calculated by adding the number of teeth with PPD from 4 to 5 mm to the number of teeth with PPD ≥6 mm multiplied by 2.

### NMR Metabolomics

Serum samples of the participants were analyzed with a high-throughput serum NMR metabolomics platform as described previously (Würtz et al. 2017) by Nightingale Health. Concentrations of 157 metabolites were available for 6,229 and 452 participants of the Health-2000 survey and Parogene, respectively. In addition, 4,116 participants of the Health-2000 gave follow-up serum samples for metabolic profiling 11 y after the baseline examination.

### Statistical Analyses

All statistical analyses were performed with R (v3.6.1 or higher, http://www.r-project.org/).

Metabolite concentrations were log(x + 1) transformed to obtain approximately normal distributions. Thereafter, the measures were scaled to a mean of zero and a standard deviation of 1. We fitted linear regression models between metabolite measures and oral parameters describing periodontal and endodontic conditions in both study populations. The results from the 2 cohorts were combined by a fixed-effect meta-analysis.

For follow-up data of Health-2000, we fitted linear regression models with metabolite concentrations at follow-up as the outcome and oral parameters at baseline as predictors. Both cross-sectional and longitudinal analyses were adjusted for number of teeth, age, sex, smoking (current/former/never), diabetes, and the level of education (only Health-2000), and longitudinal analyses were additionally adjusted for baseline metabolite concentration. Due to the correlated nature of data, we conducted a principal component analysis to define the threshold for statistical significance. The first 18 principal components explained more than 95% of the variation of the metabolomics data. Thus, the *P* value threshold for statistical significance was set to 0.05/18 = 0.0028 (false discovery rate FDR values shown in Appendix tables).

## Results

The main characteristics of the study participants are presented in Table 1. The mean age of Parogene patients was higher than that of Health-2000 participants (63.5 vs. 52.8 y), and Parogene included fewer female participants (34.5 vs. 54.6%). As expected, diabetes, hypertension, and CVD were more often present in Parogene patients. Decayed teeth (mean 0.98 vs. 0.45) and RCF (mean 2.18 vs. 1.50), ABL (76.4 vs. 29.5%), and pathologically deepened periodontal pockets (mean 7.16 vs. 4.14) were more common in the Parogene than the Health-2000 population.

**Table 1.** Characteristics of the Participants.

Characteristic	Parogene	Health-2000
*n*	452	6,229
Age, mean (SD), y	63.5 (8.9)	52.8 (14.9)
BMI, mean (SD), kg/m^2^	27.8 (5.0)	27.0 (4.4)
Women, n (%)	156 (34.5)	3,402 (54.6)
Smoking, n (%)		
Current	54 (12.0)	1,641 (26.3)
Former	181 (40.1)	1,353 (21.7)
Never	216 (47.8)	3,235 (51.9)
Diabetes, n (%)	105 (23.2)	346 (5.6)
Hypertension^a^, n (%)	288 (63.7)	1,907 (30.6)
Cardiovascular disease^b^, n (%)	305 (67.5)	597 (9.6)
Number of teeth, mean (SD)	20.0 (8.9)	19.4 (10.8)
BOP, mean (SD), %^c^	37.8 (19.0)	—
BOP, mean (SD), number of sextants^c^	—	1.98 (2.13)
PPD ≥4 mm, mean (SD) (*n* of teeth)	7.15 (5.96)	4.14 (5.53)
Caries, mean (SD)	0.98 (1.52)	0.45 (1.14)
Apical rarefactions, mean (SD)	0.35 (0.95)	0.42 (0.91)
RCF, mean (SD) (*n* of teeth)	2.18 (2.24)	1.50 (1.95)
Quality of RCF, mean (SD)		
Adequate	1.11 (1.38)	0.78 (1.24)
Inadequate	1.07 (1.33)	0.71 (1.22)

BMI, body mass index; BOP, bleeding on probing; PPD, probing pocket depth; RCF, root canal filling.

a Having medication for hypertension (Parogene), self-reported (Health-2000).

b History of a cardiovascular disease (Health-2000), ≥50% stenosis in coronary angiography (Parogene).

c BOP was recorded as the percentage of bleeding sites in Parogene and as the number of sextants with BOP in Health-2000.

**Figure 1.**
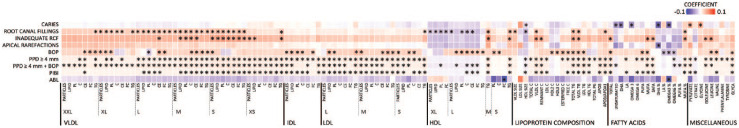
Strengths of associations between oral parameters and metabolite measures. From Parogene and Health-2000 cohorts, concentrations of 157 metabolites were determined by a nuclear magnetic resonance platform. Their associations with parameters from the oral examination were investigated by using linear regression analyses. The analyses were adjusted for age, gender, smoking, number of teeth, diabetes, and the level of education (Health-2000). The results from the 2 cohorts were combined in a meta-analysis, except for BOP, which could not be harmonized between the cohorts. The estimates are shown color coded and the statistically significant associations (*P* < 0.0028) are indicated with asterisks. Only metabolites that were associated significantly with at least 1 oral parameter are displayed. ABL, alveolar bone loss; APOA1, apolipoprotein A-I; APOB, apolipoprotein B; BOP, bleeding on probing; C, cholesterol; CE, cholesteryl ester; DHA, docosahexaenoic acid; FC, free cholesterol; GLYCA, glycoprotein acetyl; MUFA, monounsaturated fatty acid; PIBI, periodontal inflammatory burden index; PL, phospholipid; PPD, probing pocket depth; PUFA, polyunsaturated fatty acid; RCF, root canal filling; SAFA, saturated fatty acid; TG, triglyceride.

Associations of 157 metabolic measures with oral health parameters were analyzed with linear regression models (Appendix Tables 1 and 2). The associations between oral parameters and metabolite concentrations from the meta-analyses are illustrated in Figure 1. Overall, we found 13 significant associations between oral parameters and metabolic measures in Parogene, while the number of significant associations was 168 in Health-2000. In the meta-analyses, the number of teeth with PPD ≥4 mm displayed the highest number of significant associations (93) followed by PPD ≥4 mm combined with BOP (88), PIBI (77), the number of teeth with RCF (47), inadequate RCF (27), and the number of decayed teeth (8). ABL was associated with concentrations of 2 metabolites, and apical rarefactions were associated with the concentration of 1 metabolite.

**Figure 2.**
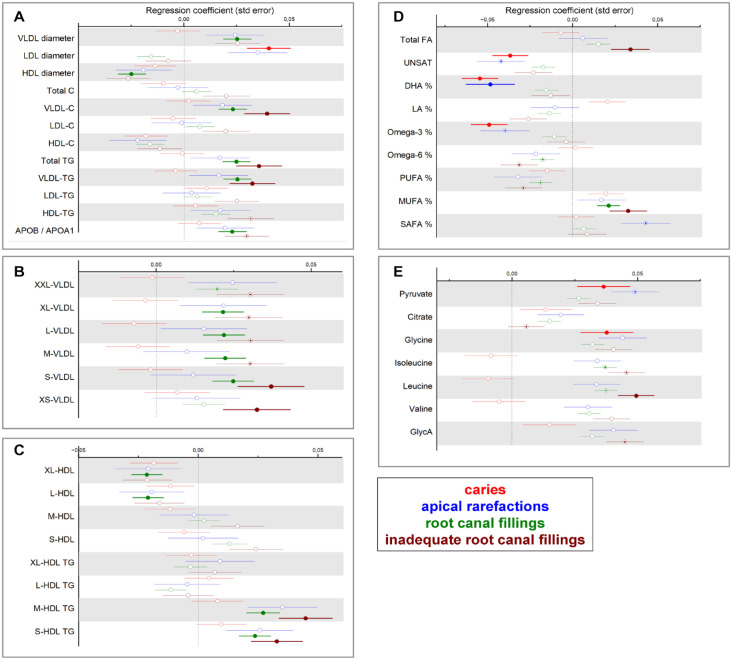
Associations between endodontic parameters and metabolite concentrations. From Parogene and Health-2000 cohorts, concentrations of 157 metabolites were determined by a nuclear magnetic resonance platform. Their associations with parameters from the oral examination were investigated by using linear regression analyses. (**A**) Lipoprotein diameters, cholesterol, triglyceride, and apolipoprotein concentrations; (**B**) very low-density lipoprotein particle sizes; (**C**) high-density lipoprotein particle sizes and triglyceride contents; (**D**) fatty acids; and (**E**) glycolysis-related parameters, amino acids, and inflammation marker GlycA. The analyses were adjusted for age, gender, smoking, number of teeth, diabetes, and the level of education (Health-2000). The results from the 2 cohorts were combined in a meta-analysis. Statistically significant (*P* < 0.0028) associations are indicated with closed circles and suggestively significant (*P* < 0.01) associations with dots inside circles.

Associations between caries and endodontic parameters and selected metabolic measures are presented in Figure 2. The number of decayed teeth presented positive associations with LDL diameter and the concentrations of pyruvate and glycine. Negative associations were found between caries and the unsaturation degree of FAs (UNSAT), the proportion of docosahexaenoic acid (DHA%), and omega-3 FAs (omega-3%). These negative associations displayed substantial heterogeneity in the meta-analysis (*I*^2^ > 75%, *P* < 0.05). However, when assessed separately in 2 cohorts, the direction and significance of associations were found to be similar. The number of teeth with RCF or inadequate RCF was positively associated with VLDL diameter, VLDL cholesterol, total triglyceride (TG) concentration, VLDL-TG, apolipoprotein B (apoB)/apolipoprotein A-I (apoA-I) ratio, number of VLDL particles, TG in medium and small HDL particles, the proportion of monounsaturated FAs (MUFA%), and leucine concentration.

**Figure 3.**
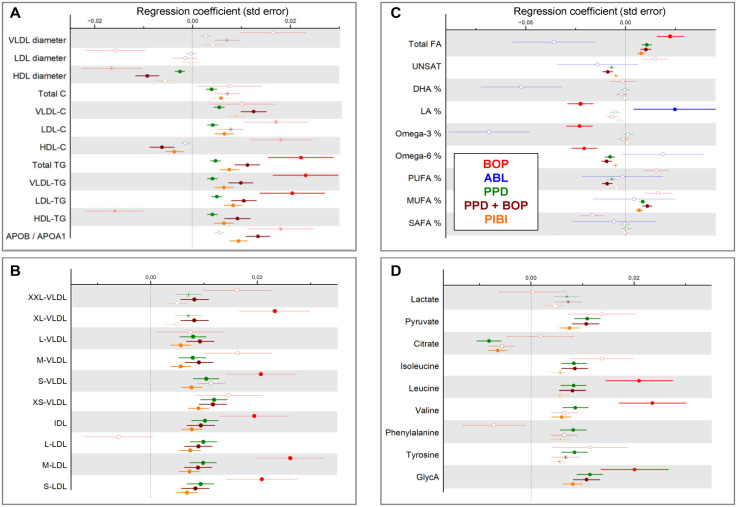
Associations between periodontal parameters and metabolite concentrations. From Parogene and Health-2000 cohorts, concentrations of 157 metabolites were determined by a nuclear magnetic resonance platform. Their associations with parameters from the oral examination were investigated by using linear regression analyses. (**A**) Lipoprotein diameters, cholesterol, triglyceride, and apolipoprotein concentrations; (**B**) very low-density lipoprotein and low-density lipoprotein particle sizes; (**C**) fatty acids; and (**D**) glycolysis-related parameters, amino acids, and inflammation marker GlycA. The analyses were adjusted for age, gender, smoking, number of teeth, diabetes, and the level of education (Health-2000). The results from the 2 cohorts were combined in a meta-analysis, except for bleeding on probing (BOP), which could not be meta-analyzed because of methodological differences in the dental examinations between the cohorts. For BOP, results from Health-2000 are shown. Alveolar bone loss is not shown in panels A, B, and D since it did not have any statistically significant associations with any metabolites in those panels. Statistically significant (*P* < 0.0028) associations are indicated with closed circles and suggestively significant (*P* < 0.01) associations with dots inside circles.

Associations between periodontal parameters and selected metabolic measures are presented in Figure 3. BOP was positively associated with several lipoprotein-related parameters, including total TG concentration and VLDL and LDL triglycerides, the number of IDL particles, and the number of medium and small LDL particles. PPD ≥4 mm was associated with total cholesterol, VLDL and LDL cholesterol, TG, and the numbers of VLDL, IDL, and LDL particles; total FA concentration; MUFA%, pyruvate; citrate; isoleucine; leucine; valine; phenylalanine; tyrosine; and GlycA. In addition, PPD ≥4 mm with BOP was associated positively with apoB/apoA-I ratio and negatively with HDL diameter, HDL cholesterol, FA unsaturation degree, omega-6%, and proportion of polyunsaturated FAs (PUFA%).

Participants of the Health-2000 survey (*n* = 4,116) gave serum samples 11 y after the baseline. Overall, Appendix Table 3 shows the average delta between the baseline and the follow-up measures.

We analyzed whether the oral health parameters at baseline were associated with changes of metabolic measures during follow-up. Number of teeth was a strong determinant of estimate levels, and thus we analyzed the results separately for those with ≥20 teeth or <20 teeth at baseline (Appendix Tables 4 and 5). Number of significant associations (≥20 teeth/<20 teeth) were for caries 30/11, for RCF 3/0, for inadequate RCF 5/0, for apical rarefactions 3/0, for PPD ≥4 mm 1/2, for BOP 8/0, for PPD ≥4 mm + BOP 1/0, for PIBI 1/2, and for ABL 0/0. Significant associations between caries, BOP, and metabolites are presented for subjects with ≥20 teeth at baseline in Table 2. In the follow-up, baseline caries was associated with changes of IDL lipids, phospholipids, free cholesterol, non-TG lipids in XL- and L-HDL, HDL_3_ cholesterol, apoA-I, and several FA levels. Mean changes of VLDL-, IDL-, and LDL-related parameters were positive in those with fewer decayed teeth at baseline and negative in those with more decayed teeth (Appendix Table 6). The number of XL-HDL particles and XL-HDL lipid concentration, serum total cholesterol and HDL_3_ cholesterol, PUFA, and omega-3 increased in individuals with fewer decayed teeth and decreased in those with more decayed teeth.

**Table 2.** Longitudinal Associations of Oral Health Parameters with Changes of Metabolic Measures in the Follow-up in Participants with Functional Dentition (Number of Teeth ≥20) at Baseline.

Target	Subclass	Metabolite	Caries (Teeth with Caries)			BOP (Number of Sextants)		
			β	SD for β	*P*	β	SD for β	*P*
VLDL	XS	Cholesterol	−0.055	0.018	**0.0025**			NS
	XS	Cholesterol esters	−0.056	0.018	**0.0021**			NS
	XS	Free cholesterol	−0.051	0.018	0.0047			NS
IDL		Particles	−0.055	0.018	**0.0026**			NS
		Lipids	−0.055	0.018	**0.0024**			NS
		Phospholipids	−0.058	0.018	**0.0014**			NS
		Cholesterol	−0.054	0.018	0.0031			NS
		Free cholesterol	−0.064	0.018	**0.0004**			NS
LDL	L	Particles	−0.054	0.018	0.0035			NS
	L	Lipids	−0.051	0.018	0.0045			NS
	L	Phospholipids	−0.052	0.018	0.0043			NS
	L	Cholesterol	−0.053	0.018	0.0037			NS
	L	Cholesterol esters	−0.051	0.018	0.0052			NS
	L	Free cholesterol	−0.060	0.018	**0.0010**			NS
	M	Cholesterol	−0.051	0.018	0.0048			NS
	M	Cholesterol esters	−0.052	0.018	0.0042			NS
	S	Cholesterol	−0.055	0.018	**0.0026**			NS
	S	Cholesterol esters	−0.055	0.018	**0.0024**			NS
	S	Free cholesterol	−0.053	0.018	0.0034			NS
HDL	XL	Particles	−0.038	0.012	**0.0024**	−0.018	0.006	**0.0023**
	XL	Lipids	−0.041	0.012	**0.0010**	−0.017	0.006	0.0037
	XL	Phospholipids	−0.035	0.012	0.0039	−0.016	0.006	0.0056
	XL	Cholesterol	−0.043	0.013	**0.0010**	−0.017	0.006	0.0051
	XL	Cholesterol esters	−0.045	0.013	**0.0007**	−0.017	0.006	0.0069
	XL	Free cholesterol	−0.036	0.013	0.0049	−0.019	0.006	**0.0019**
	L	Particles	−0.041	0.014	0.0036	−0.020	0.007	0.0031
	L	Lipids	−0.043	0.014	**0.0017**			NS
	L	Phospholipids	−0.047	0.014	**0.0013**	−0.019	0.007	0.0072
	L	Cholesterol	−0.035	0.013	0.0080	−0.017	0.006	0.0085
	L	Cholesterol esters	−0.035	0.013	0.0085	−0.017	0.006	0.0086
	L	Free cholesterol			NS	−0.019	0.006	0.0028
Diameter		VLDL	0.041	0.015	0.0089	0.024	0.007	**0.0012**
		HDL	−0.035	0.012	0.0031			NS
Cholesterol		Serum	−0.059	0.018	**0.0010**	−0.024	0.009	0.0047
		LDL	−0.051	0.018	0.0045			NS
		HDL	−0.044	0.015	0.0040	−0.024	0.007	**0.0010**
		HDL_2_	−0.040	0.015	0.0070	−0.022	0.007	**0.0016**
		HDL_3_	−0.063	0.017	**0.0002**	−0.025	0.008	**0.0016**
		Esterified cholesterol	−0.058	0.018	**0.0013**	−0.025	0.009	0.0043
		Free cholesterol	−0.063	0.018	**0.0005**	−0.023	0.009	0.0068
Apolipoprotein		ApoA-I	−0.054	0.017	**0.0017**	−0.031	0.008	**0.0001**
Fatty acids		Total phosphatidylglycerol	−0.059	0.018	**0.0010**			NS
		Phosphatidylcholine	−0.062	0.018	**0.0006**			NS
		Sphingomyelin	−0.074	0.017	**1.3E-05**	−0.022	0.008	0.0053
		Total choline	−0.071	0.018	**0.0001**	−0.023	0.008	0.0064
		PUFA	−0.063	0.018	**0.0006**			NS
		Omega-6	−0.057	0.018	**0.0020**			NS
		DHA%	−0.054	0.016	**0.0011**			NS
		Omega-3%	−0.052	0.017	**0.0018**	−0.025	0.008	**0.0015**
		MUFA%			NS	0.021	0.007	0.0042
Other		Pyruvate	0.049	0.017	0.0051			NS
		Glycerol	0.067	0.021	**0.0011**			NS
		Creatinine	−0.048	0.015	**0.0020**	−0.020	0.007	0.0069

*P* values under the threshold 0.0028 are bolded; other *P* values are on a suggestive level (*P* < 0.01). Models were adjusted for age, sex, smoking, diabetes, level of education, and baseline metabolite concentrations.

BOP, bleeding on probing; DHA, docosahexaenoic acid; HDL, high-density lipoprotein; IDL, intermediate-density lipoprotein; LDL, low-density lipoprotein; MUFA, monounsaturated fatty acid; NS, nonsignificant; PUFA, polyunsaturated fatty acid; VLDL, very low-density lipoprotein.

BOP at baseline was associated with changes in VLDL particle size; composition of L-HDL particles; serum cholesterol including HDL, HDL_2_, and HDL_3_ cholesterol; apoA-I; and omega-3% in follow-up. The increase in VLDL particle size during follow-up was larger in the subgroup with BOP (Appendix Table 6).

## Discussion

In this study including 2 populations, a follow-up, and altogether 6,681 participants, we show that signs of current or past infectious/inflammatory oral diseases were associated with a large number of unbeneficial circulating metabolic measures. We found that various signs of oral diseases were associated with rises in VLDL-related parameters, including particle number, size, and neutral lipids. In addition, we found marked associations between oral diseases and increased saturation degree of FAs, lower levels of omega-3 FAs, and increased levels of glycolysis-related metabolites. These metabolic profiles, which were independent of age, sex, number of teeth, diabetes, education, or smoking status, are inflammatory in nature. Furthermore, periodontal inflammation was associated with GlycA, a novel biomarker of systemic inflammation.

Systemic inflammation is an essential feature in the onset, maintenance, and exacerbation of cardiometabolic disorders. So far, metabolomics studies on the contribution of oral diseases to metabolic profiles are scarce, and prospective studies do not exist. Thus, our study is one of those in the frontline. Caries and periodontitis are putatively associated with systemic inflammation through overlapping mechanisms, although the role of caries in systemic health has been far less investigated. Caries reflects a high-carbohydrate/sugar-rich diet, which induces receptors of advanced glycation end products, promoting oxidative stress and inflammation (Sabharwal et al. 2021). Local inflammation in the oral cavity may lead to the presence of proinflammatory mediators in circulation promoting systemic inflammation (Sharma et al. 2017; Garrido et al. 2019). An additional mechanism resulting in systemic inflammation in periodontitis is the translocation of bacteria from the oral cavity directly to blood or down via the gastrointestinal tract (Kitamoto and Kamada 2022). Our results suggest that metabolic profiles in individuals with caries or periodontal inflammation resemble each other, but caries/endodontic parameters are associated with fewer metabolic markers than periodontal parameters, suggesting smaller systemic impact.

The main lipoprotein class whose compositional alterations were associated with periodontal inflammation and the number of RCF was apoB-containing, proatherogenic lipoproteins. RCFs were directly linked to VLDL diameter and the number of VLDL particles of all sizes, whereas BOP and PPD ≥4 mm were positively associated with VLDL cholesterol, apoB, and the number of smaller apoB-containing particles, such as S-VLDL, IDL, and L-LDL. The NMR platform categorizes VLDL into 6 subclasses (Wang et al. 2012); the diameter of XXL- and XL-particles exceeds 64 nm, whereas the S-sized have an average size of 37 nm. Although XX- or XL-VLDL cannot enter the intima of the vessel walls, they have been associated with cardiometabolic diseases (Lee et al. 2022). Large VLDL particle size has been associated with carbohydrate-rich diet (Feingold and Grunfeld 2022), which may explain the observed associations between RCF and VLDL parameters. The smaller VLDL, IDL, and LDL, however, can more readily cross the endothelium (Borén et al. 2000). During inflammation, activity of lipoprotein lipase decreases maintaining large VLDL size, whereas lipolysis of adipose tissue provides FAs for increased hepatic triglyceride synthesis (Khovidhunkit et al. 2004). Thus, an increased amount of triglycerides is available for formation and hepatic secretion of VLDL (Feingold and Grunfeld 2022). Our observations indicate disturbances in both VLDL production and clearance in oral diseases.

In addition to an overall decrease of HDL, inflammation leads to structural changes diminishing the antiatherogenic character of HDL (Khovidhunkit et al. 2004): HDL remodeling produces large amounts of small HDL due to replacement of apoA-I with serum amyloid A, and the particles become enriched with triglycerides. Earlier publications suggest that childhood caries and cumulative signs of oral inflammations are associated with low HDL cholesterol levels in adulthood (Pussinen et al. 2019, 2020). In addition, adult periodontitis patients have lower HDL cholesterol levels than those without periodontitis (Buhlin et al. 2003), and HDL in periodontitis patients displays decreased ability to function in reverse cholesterol transport (Pussinen et al. 2004). Also in the present study, the amount of HDL-TG increased in the presence of periodontal inflammation and in individuals with RCFs. In addition, HDL particle size was smaller and the number of large HDL particles lower in individuals with RCFs. Thereby, oral diseases were associated with HDL characteristics resembling those found in acute-phase HDL (Khovidhunkit et al. 2004).

ApoB/apoA-I ratio was positively associated with RCFs and PPD ≥4 mm. ApoB/apoA-I ratio, which constitutes from the major proteins of LDL and HDL, is regarded as an indicator of the balance of proatherogenic and antiatherogenic lipoproteins as well as inflammatory status (Wu et al. 2019). Markers of periodontal inflammation were also positively associated with a novel inflammatory biomarker, GlycA. The GlycA signal arises largely from the glycan portions of acute-phase proteins. Its levels are associated with interleukin-6, C-reactive protein, and serum amyloid A (Ritchie et al. 2015; Connelly et al. 2017), and it reflects chronic inflammation, cardiometabolic risk, and poor survival (Ritchie et al. 2015; Julkunen et al. 2023).

Unlike other periodontal parameters, ABL had mostly nonsignificant associations with metabolites. The development of ABL is usually a slow process that takes place over decades. Conventional radiographs demonstrate bone loss only after a significant amount of destruction (Zaki et al. 2015). Thus, panoramic radiographs are not optimal for detecting current disease activity, which might explain the weak association with metabolic measures. Instead, periodontal pockets are a favorable growth site for dysbiotic biofilm, which initiates and maintains the host immune-inflammatory response. It is clinically marked by BOP, which still is a gold standard marker of active periodontal inflammation (Bartold and Van Dyke 2017).

Several parameters related to FA synthesis/turnover were associated with caries, endodontic lesions, RCFs, and periodontal inflammation. BOP was associated with increased levels of saturated FAs, as well as PPD ≥4 mm and caries with a clear decrease in unsaturated FAs. The omega-3 PUFAs decreased in the presence of caries and periapical inflammations, and omega-6 PUFAs decreased in individuals with BOP and PPD ≥4 mm. Several pathways are likely to contribute to these findings: first, an oxidative response to inflammation could induce peroxidation/modification of these highly unsaturated FAs (Khovidhunkit et al. 2004). Second, the omega-3 and omega-6 FA pool is fueled into proresolving and proinflammatory lipid mediator synthesis in oral diseases (Sommakia and Baker 2017). In addition, the FA composition of serum reflects diet, which obviously has a major effect on oral health. In the recent atlas of plasma NMR metabolites, MUFA was the biomarker that was associated with the highest number of disease end points and displayed similar disease clustering as GlycA (Julkunen et al. 2023). The authors suggested that several FA-related biomarkers should be considered markers of systemic inflammation rather than markers of recent diet (Julkunen et al. 2023).

Infectious/inflammatory oral diseases were also associated with non-lipid-related metabolic measures. Pyruvate concentrations were elevated in patients with caries, endodontic lesions, and periodontal inflammation. Our findings could be indicative of increased aerobic glycolysis and disrupted carbon flux through the citric acid cycle similarly as in endotoxemia (Määttä et al. 2021). These metabolites may also reflect higher sugar content in diet (Wang et al. 2012). Amino acids, which have been consistently associated earlier with periodontitis in different biofluids (Brito et al. 2022), were in the present study upregulated in individuals with caries, inadequate RCF, and signs of periodontal inflammation.

In addition to cross-sectional associations, our observations indicate that infectious/inflammatory oral diseases may predict future metabolic profiles. In the follow-up of the Health-2000 population, baseline caries and BOP were associated with decreases of L-HDL particle concentration, their non-TG lipid content, and apoA-I, but associations with proatherogenic apoB-containing lipoproteins were also observed. It is known that serum cholesterol concentration decreases during inflammation due to its reduction in both HDL and LDL (Khovidhunkit et al. 2004). These associations were more obvious in participants with functional dentition, since the number of teeth was a strong confounder. Although no information on the number of occluding pairs was available, 20 teeth was selected as a cutoff for “adequate dentition” because functional dentition comprises ≥10 occluding pairs (Ikebe et al. 2007). Any professional intervention, such as the number of RCFs (Liljestrand et al. 2021), may be a sign of better health-consciousness and the utilization of health care services. This may be a more relevant predictor of future metabolic profiles than oral health. Unfortunately, we did not have information on potential periodontal/restorative/endodontic treatment during the follow-up, which warrants for further research.

Strengths of the study include the sample including both a population-based study with adults of all ages and a cohort of elderly patients with cardiac symptoms. The study populations were obviously different regarding the presence of CVD or their risk factors, such as hypertension, and some parameters were collected from the populations using different methods. However, virtually all significant associations observed between oral diseases and metabolic measures were similar in both study populations, strengthening the credibility of the findings. Limitations of the study include the exclusion of several potentially important determinants of the metabolic profiles: we only adjusted the analyses for diabetes, but other cardiometabolic diseases, such as CVD or hypertension, and their medications may also have an impact. In addition, reverse causality may introduce a bias in the analyses, as the results from the cross-sectional part of our study were stronger than those from the follow-up part. The contribution of unbeneficial metabolic profiles on future oral disease risk remains unknown. Diet may be reflected in virtually all metabolites investigated, which was clearly shown in the short term when comparing postprandial and fasting samples (Bermingham et al. 2023): 85% of the metabolites of the present panel changed significantly during fasting. In the present study, both caries and BOP were associated with several FA measures, such as saturation degree, suggesting an unhealthy diet. Thus, consumption of fat, sugar, vegetables, fibers, and alcohol would have been of interest but represents a topic for a separate study. Overall, the metabolic measures are affected by several lifestyle choices, diseases, medications, and even genetics (Nath et al. 2017). Despite these flaws, our study is the first to investigate the association between serum metabolomics and oral health with a large sample including 2 study populations, both caries- and periodontitis-related parameters, and with follow-up data.

We conclude that both caries- and periodontitis-related signs of oral diseases are associated with unbeneficial metabolic profiles with an inflammatory nature. Due to the high prevalence of oral diseases in most populations, their role in the total inflammatory burden is significant, and they may lead to deteriorating public health. Infectious/inflammatory oral diseases may represent a modifiable risk factor for systemic chronic inflammation and thus cardiometabolic disorders.

## Author Contributions

A. Salminen, contributed to design, data acquisition, analysis, and interpretation, drafted the manuscript; A.M. Määttä, J. Leskelä, contributed to data analysis and interpretation, critically revised the manuscript; P. Mäntylä, A.L. Suominen, contributed to design, data acquisition and interpretation, critically revised the manuscript; M. Pietiäinen, contributed to conception, data interpretation, critically revised the manuscript; K. Buhlin, S. Paju, contributed to data acquisition and interpretation, critically revised the manuscript; W. Sattler, contributed to data interpretation, critically revised the manuscript; J. Sinisalo, contributed to conception, design, data acquisition and interpretation, critically revised the manuscript; P.J. Pussinen, contributed to conception, design, data acquisition and interpretation, drafted the manuscript. All authors gave final approval and agree to be accountable for all aspects of the work.

## Supplemental Material

sj-docx-1-jdr-10.1177_00220345231203562 – Supplemental material for Systemic Metabolic Signatures of Oral Diseases Click here for additional data file. Supplemental material, sj-docx-1-jdr-10.1177_00220345231203562 for Systemic Metabolic Signatures of Oral Diseases by A. Salminen, A.M. Määttä, P. Mäntylä, J. Leskelä, M. Pietiäinen, K. Buhlin, A.L. Suominen, S. Paju, W. Sattler, J. Sinisalo and P.J. Pussinen in Journal of Dental Research
