# Supplementary material for: Systemic Metabolic Signatures of Oral Diseases
Source: J Dent Res. 2023 Nov 15;103(1):13–21. doi: 10.1177/00220345231203562 (PMC10734208; doi:10.1177/00220345231203562)
Supplement: sj-docx-1-jdr-10.1177_00220345231203562 – Supplemental material for Systemic Metabolic Signatures of Oral Diseases [file sj-docx-1-jdr-10.1177_00220345231203562.docx]

**Circulating metabolomic signatures of oral inflammations**

Salminen A, Määttä AM, Mäntylä P, Leskelä J, Pietiäinen M, Buhlin K, Suominen AL, Paju S, Sattler W, Sinisalo J, Pussinen PJ

**APPENDIX**

**Appendix Materials and methods**

*Parogene*

The COROGENE study included 5,297 patients who underwent coronary angiography for any reason in Helsinki University Hospital, Helsinki, Finland, between 2006 and 2008 (Vaara et al. 2012). All participants signed an informed consent form and the study was approved by the ethics committee of the Helsinki University Hospital. A random sample of 10% of the COROGENE patients were invited to participate in the Parogene substudy (Buhlin et al. 2011). In the Parogene study, 506 participants underwent an extensive clinical oral examination. Oral examinations were done for at least 6 weeks, but no later than 5 months after the angiography. Probing pocket depths (PPDs) were registered from six sites around each tooth by manual probing, and bleeding on probing (BOP) from four sites around each tooth excluding wisdom teeth. The number of teeth with at least one site with a PPD of ≥4 mm was registered. Digital panoramic radiographs were taken and alveolar bone loss (ABL) was calculated by choosing the tooth with the most severe bone loss from each dentate sextant. ABL was categorized as follows: 0, no bone loss; 1, ABL in the cervical third of the root; 2, ABL in the middle third of the root; 3, ABL in the apical third of the root; 4, total bone support loss. The mean value of these six measurements was calculated for each patient. In addition, the numbers of teeth with decay, root canal fillings, inadequate root canal fillings, widened periapical spaces (periapical index 3) (Ørstavik et al. 1986), and apical rarefactions (periapical index 4 to 5) were determined from the radiographs. The periodontal inflammatory burden index (PIBI) was calculated by adding the number of teeth with PPD from 4 to 5 mm to the number of teeth with PPD ≥ 6 mm multiplied by two. A variable describing active periodontal inflammation (PPD + BOP) was created from the number of teeth with PPD of ≥ 4 mm by excluding individuals with BOP < 10%.

*Health 2000 survey*

The Health 2000 survey was a nationwide study in Finland, which used a stratified two-stage cluster sample of 8,028 individuals aged 30 years and older. Data collection included structured health interviews, blood sample collection, and clinical examinations. Oral examinations were conducted for 6,335 participants and panoramic radiographs were taken from 6,115 participants (Suominen-Taipale et al. 2008). The ethics committees of the Helsinki University Hospital and the National Public Health Institute gave permission for the study. Each survey participant signed an informed consent form.

In the clinical oral examination, PPD was measured from four sites around each tooth, excluding the wisdom teeth, and the deepest measurement was recorded by tooth as a PPD of 0 to 3 mm, a PPD of 4 to 5 mm, or a PPD of ≥6 mm. Immediately after pocket probing, BOP was recorded by sextants (bleeding or no bleeding). ABL was determined from panoramic radiographs in each dentate sextant and categorized similarly as in the Parogene study. In addition, the numbers of teeth with caries, root canal fillings, inadequate root canal fillings, and apical rarefactions were determined from the radiographs. A variable describing active periodontal inflammation (PPD + BOP) was created from the number of teeth with PPD of ≥ 4 mm by including only those teeth with PPD that had bleeding on probing in the same sextant.

*NMR metabolomics*

There were data on 157 metabolites including concentrations and compositions of lipoprotein subclass particles as well as concentrations of fatty acids, amino acids, ketone bodies, and other molecules involved in cell metabolism. Relative proportions of fatty acids and apo(lipoprotein)B/apoA-I were included, while other ratios were not used in the analyses.

*Statistical significance*

The NMR metabolomics platform measures the concentrations of different molecules from the same particles, and thus, these concentrations are highly correlated (Würtz et al. 2017). Due to this correlated nature of the data, we conducted a principal component analysis to define the threshold for statistical significance. The first 18 principal components explained more than 95% of the variation of the metabolomics data. Thus, the P value threshold for statistical significance was set to 0.05/18 = 0.0028. In Appendix tables, FDR values (Benjamini Hochberg correction) are also shown.

**APPENDIX TABLE 1.** **Associations of 157 metabolic measures with caries and endodontic parameters.** Linear regression adjusted for age, gender, smoking, number of teeth, and diabetes (in Health-2000 and Parogene), and level of education (in Health-2000 only). Significant p-values (< 0.0028) are indicated by red colour. Significant FDRs (< 0.05) for meta-analysis are indicated by purple colour.

| **CARIES** | | | | | | | | | | | | |
| --- | --- | --- | --- | --- | --- | --- | --- | --- | --- | --- | --- | --- |
| **Metabolite** | | | **Parogene** | | | **Health 2000** | | | **Meta-analysis** | | | |
|  | | | Beta | SE | p | Beta | SE | p | Beta | SE | p | FDR |
| VLDL | XXL | particles | -0.004 | 0.032 | 0.90 | -0.001 | 0.011 | 0.92 | -0.001 | 0.010 | 0.889 | 0.99 |
|  |  | lipid | -0.002 | 0.032 | 0.94 | -0.001 | 0.011 | 0.94 | -0.001 | 0.010 | 0.924 | 0.99 |
|  |  | phospholipid | -0.008 | 0.032 | 0.80 | 0.000 | 0.011 | 0.97 | -0.001 | 0.011 | 0.906 | 0.99 |
|  |  | cholesterol | -0.002 | 0.032 | 0.96 | -0.001 | 0.011 | 0.90 | -0.001 | 0.010 | 0.888 | 0.99 |
|  |  | cholesteryl ester | 0.009 | 0.032 | 0.79 | -0.005 | 0.011 | 0.63 | -0.004 | 0.011 | 0.715 | 0.99 |
|  |  | free cholesterol | -0.013 | 0.032 | 0.69 | 0.000 | 0.011 | 0.99 | -0.001 | 0.010 | 0.906 | 0.99 |
|  |  | triglycerides | -0.003 | 0.032 | 0.94 | -0.002 | 0.011 | 0.89 | -0.002 | 0.011 | 0.871 | 0.99 |
|  | XL | particles | -0.011 | 0.032 | 0.73 | -0.003 | 0.011 | 0.80 | -0.004 | 0.010 | 0.723 | 0.99 |
|  |  | lipid | -0.008 | 0.032 | 0.81 | -0.003 | 0.011 | 0.81 | -0.003 | 0.010 | 0.761 | 0.99 |
|  |  | phospholipid | -0.012 | 0.032 | 0.70 | 0.000 | 0.011 | 0.98 | -0.002 | 0.010 | 0.879 | 0.99 |
|  |  | cholesterol | -0.009 | 0.032 | 0.79 | -0.001 | 0.011 | 0.91 | -0.002 | 0.010 | 0.849 | 0.99 |
|  |  | cholesteryl ester | -0.004 | 0.032 | 0.91 | -0.005 | 0.011 | 0.64 | -0.005 | 0.010 | 0.628 | 0.99 |
|  |  | free cholesterol | -0.015 | 0.032 | 0.65 | 0.000 | 0.011 | 0.98 | -0.001 | 0.010 | 0.901 | 0.99 |
|  |  | triglycerides | -0.009 | 0.032 | 0.78 | -0.003 | 0.011 | 0.77 | -0.004 | 0.010 | 0.709 | 0.99 |
|  | L | particles | -0.011 | 0.032 | 0.72 | -0.007 | 0.011 | 0.53 | -0.007 | 0.010 | 0.476 | 0.99 |
|  |  | lipid | -0.004 | 0.032 | 0.89 | -0.005 | 0.011 | 0.62 | -0.005 | 0.010 | 0.607 | 0.99 |
|  |  | phospholipid | -0.010 | 0.032 | 0.75 | -0.006 | 0.011 | 0.61 | -0.006 | 0.010 | 0.558 | 0.99 |
|  |  | cholesterol | -0.002 | 0.032 | 0.94 | -0.006 | 0.011 | 0.59 | -0.006 | 0.010 | 0.590 | 0.99 |
|  |  | cholesteryl ester | 0.009 | 0.032 | 0.78 | -0.007 | 0.011 | 0.55 | -0.005 | 0.010 | 0.632 | 0.99 |
|  |  | free cholesterol | -0.015 | 0.032 | 0.64 | -0.006 | 0.011 | 0.61 | -0.007 | 0.010 | 0.528 | 0.99 |
|  |  | triglycerides | -0.009 | 0.031 | 0.79 | -0.007 | 0.011 | 0.53 | -0.007 | 0.010 | 0.500 | 0.99 |
|  | M | particles | -0.004 | 0.032 | 0.90 | -0.006 | 0.011 | 0.57 | -0.006 | 0.010 | 0.563 | 0.99 |
|  |  | lipid | 0.006 | 0.032 | 0.85 | -0.005 | 0.011 | 0.68 | -0.003 | 0.010 | 0.740 | 0.99 |
|  |  | phospholipid | 0.001 | 0.032 | 0.98 | -0.004 | 0.011 | 0.69 | -0.004 | 0.010 | 0.710 | 0.99 |
|  |  | cholesterol | 0.009 | 0.032 | 0.79 | -0.004 | 0.011 | 0.74 | -0.002 | 0.010 | 0.823 | 0.99 |
|  |  | cholesteryl ester | 0.018 | 0.032 | 0.58 | -0.003 | 0.011 | 0.78 | -0.001 | 0.010 | 0.934 | 0.99 |
|  |  | free cholesterol | -0.004 | 0.032 | 0.91 | -0.005 | 0.011 | 0.63 | -0.005 | 0.010 | 0.624 | 0.99 |
|  |  | triglycerides | -0.001 | 0.031 | 0.97 | -0.008 | 0.011 | 0.46 | -0.007 | 0.010 | 0.481 | 0.99 |
|  | S | particles | 0.012 | 0.032 | 0.70 | -0.003 | 0.011 | 0.75 | -0.002 | 0.010 | 0.859 | 0.99 |
|  |  | lipid | 0.019 | 0.032 | 0.56 | -0.001 | 0.011 | 0.91 | 0.001 | 0.010 | 0.940 | 0.99 |
|  |  | phospholipid | 0.022 | 0.032 | 0.49 | -0.002 | 0.011 | 0.84 | 0.000 | 0.010 | 0.979 | 0.99 |
|  |  | cholesterol | 0.014 | 0.033 | 0.68 | 0.002 | 0.011 | 0.87 | 0.003 | 0.010 | 0.774 | 0.99 |
|  |  | cholesteryl ester | 0.012 | 0.033 | 0.71 | 0.004 | 0.011 | 0.75 | 0.004 | 0.010 | 0.672 | 0.99 |
|  |  | free cholesterol | 0.013 | 0.032 | 0.69 | -0.001 | 0.011 | 0.90 | 0.000 | 0.010 | 0.992 | 0.99 |
|  |  | triglycerides | 0.013 | 0.032 | 0.68 | -0.005 | 0.011 | 0.64 | -0.003 | 0.010 | 0.752 | 0.99 |
|  | XS | particles | 0.026 | 0.032 | 0.41 | 0.005 | 0.011 | 0.67 | 0.007 | 0.010 | 0.507 | 0.99 |
|  |  | lipid | 0.024 | 0.032 | 0.45 | 0.004 | 0.011 | 0.70 | 0.006 | 0.010 | 0.540 | 0.99 |
|  |  | phospholipid | 0.018 | 0.032 | 0.57 | 0.004 | 0.011 | 0.70 | 0.006 | 0.011 | 0.577 | 0.99 |
|  |  | cholesterol | 0.018 | 0.032 | 0.58 | 0.007 | 0.011 | 0.53 | 0.008 | 0.011 | 0.442 | 0.99 |
|  |  | cholesteryl ester | 0.021 | 0.032 | 0.51 | 0.010 | 0.011 | 0.39 | 0.011 | 0.011 | 0.307 | 0.93 |
|  |  | free cholesterol | 0.010 | 0.032 | 0.76 | 0.002 | 0.011 | 0.84 | 0.003 | 0.011 | 0.771 | 0.99 |
|  |  | triglycerides | 0.030 | 0.032 | 0.35 | 0.000 | 0.011 | 0.99 | 0.003 | 0.010 | 0.749 | 0.99 |
| IDL |  | particles | 0.007 | 0.032 | 0.83 | 0.003 | 0.011 | 0.76 | 0.004 | 0.011 | 0.715 | 0.99 |
|  |  | lipid | 0.001 | 0.032 | 0.96 | 0.001 | 0.011 | 0.90 | 0.001 | 0.011 | 0.890 | 0.99 |
|  |  | phospholipid | 0.009 | 0.032 | 0.77 | 0.003 | 0.011 | 0.78 | 0.004 | 0.011 | 0.721 | 0.99 |
|  |  | cholesterol | -0.007 | 0.032 | 0.83 | 0.001 | 0.011 | 0.92 | 0.000 | 0.011 | 0.980 | 0.99 |
|  |  | cholesteryl ester | -0.008 | 0.032 | 0.81 | 0.001 | 0.011 | 0.91 | 0.000 | 0.011 | 0.982 | 0.99 |
|  |  | free cholesterol | -0.002 | 0.032 | 0.96 | 0.003 | 0.011 | 0.82 | 0.002 | 0.011 | 0.842 | 0.99 |
|  |  | triglycerides | 0.049 | 0.032 | 0.12 | 0.009 | 0.011 | 0.42 | 0.013 | 0.010 | 0.206 | 0.83 |
| LDL | L | particles | 0.002 | 0.032 | 0.95 | 0.001 | 0.011 | 0.96 | 0.001 | 0.011 | 0.947 | 0.99 |
|  |  | lipid | -0.001 | 0.032 | 0.98 | -0.002 | 0.011 | 0.89 | -0.002 | 0.011 | 0.885 | 0.99 |
|  |  | phospholipid | 0.000 | 0.032 | 0.99 | -0.001 | 0.011 | 0.90 | -0.001 | 0.011 | 0.906 | 0.99 |
|  |  | cholesterol | -0.008 | 0.032 | 0.80 | -0.002 | 0.011 | 0.87 | -0.003 | 0.011 | 0.814 | 0.99 |
|  |  | cholesteryl ester | -0.009 | 0.032 | 0.78 | -0.002 | 0.011 | 0.86 | -0.003 | 0.011 | 0.800 | 0.99 |
|  |  | free cholesterol | -0.003 | 0.032 | 0.93 | 0.001 | 0.011 | 0.96 | 0.000 | 0.011 | 0.981 | 0.99 |
|  |  | triglycerides | 0.049 | 0.031 | 0.12 | 0.009 | 0.011 | 0.41 | 0.013 | 0.010 | 0.202 | 0.83 |
|  | M | particles | -0.001 | 0.032 | 0.97 | -0.002 | 0.011 | 0.85 | -0.002 | 0.011 | 0.845 | 0.99 |
|  |  | lipid | -0.004 | 0.032 | 0.90 | -0.004 | 0.011 | 0.73 | -0.004 | 0.011 | 0.713 | 0.99 |
|  |  | phospholipid | -0.001 | 0.032 | 0.98 | -0.006 | 0.011 | 0.61 | -0.005 | 0.011 | 0.625 | 0.99 |
|  |  | cholesterol | -0.011 | 0.032 | 0.73 | -0.003 | 0.011 | 0.76 | -0.004 | 0.011 | 0.692 | 0.99 |
|  |  | cholesteryl ester | -0.011 | 0.032 | 0.74 | -0.003 | 0.011 | 0.82 | -0.003 | 0.011 | 0.745 | 0.99 |
|  |  | free cholesterol | -0.011 | 0.032 | 0.74 | -0.005 | 0.011 | 0.66 | -0.006 | 0.011 | 0.596 | 0.99 |
|  |  | triglycerides | 0.048 | 0.032 | 0.13 | 0.006 | 0.011 | 0.55 | 0.010 | 0.010 | 0.297 | 0.93 |
|  | S | particles | -0.005 | 0.032 | 0.88 | -0.006 | 0.011 | 0.58 | -0.006 | 0.011 | 0.562 | 0.99 |
|  |  | lipid | -0.007 | 0.032 | 0.82 | -0.007 | 0.011 | 0.51 | -0.007 | 0.011 | 0.485 | 0.99 |
|  |  | phospholipid | -0.012 | 0.031 | 0.70 | -0.013 | 0.011 | 0.26 | -0.013 | 0.011 | 0.235 | 0.92 |
|  |  | cholesterol | -0.010 | 0.032 | 0.75 | -0.005 | 0.011 | 0.65 | -0.006 | 0.011 | 0.597 | 0.99 |
|  |  | cholesteryl ester | -0.008 | 0.032 | 0.80 | -0.003 | 0.011 | 0.76 | -0.004 | 0.011 | 0.710 | 0.99 |
|  |  | free cholesterol | -0.016 | 0.031 | 0.62 | -0.010 | 0.011 | 0.35 | -0.011 | 0.011 | 0.298 | 0.93 |
|  |  | triglycerides | 0.033 | 0.032 | 0.30 | -0.004 | 0.011 | 0.74 | 0.000 | 0.010 | 0.986 | 0.99 |
| HDL | XL | particles | -0.023 | 0.032 | 0.47 | -0.018 | 0.011 | 0.09 | -0.018 | 0.010 | 0.064 | 0.49 |
|  |  | lipid | -0.021 | 0.031 | 0.50 | -0.020 | 0.010 | 0.05 | -0.020 | 0.010 | 0.041 | 0.44 |
|  |  | phospholipid | -0.022 | 0.031 | 0.48 | -0.015 | 0.010 | 0.14 | -0.016 | 0.010 | 0.101 | 0.63 |
|  |  | cholesterol | -0.020 | 0.032 | 0.52 | -0.024 | 0.011 | 0.02 | -0.024 | 0.010 | 0.018 | 0.26 |
|  |  | cholesteryl ester | -0.021 | 0.032 | 0.51 | -0.025 | 0.011 | 0.02 | -0.024 | 0.010 | 0.017 | 0.26 |
|  |  | free cholesterol | -0.019 | 0.031 | 0.55 | -0.021 | 0.011 | 0.04 | -0.021 | 0.010 | 0.036 | 0.43 |
|  |  | triglycerides | -0.007 | 0.033 | 0.82 | -0.003 | 0.011 | 0.82 | -0.003 | 0.011 | 0.771 | 0.99 |
|  | L | particles | -0.044 | 0.031 | 0.16 | -0.008 | 0.011 | 0.47 | -0.012 | 0.010 | 0.250 | 0.93 |
|  |  | lipid | -0.038 | 0.031 | 0.21 | -0.008 | 0.011 | 0.46 | -0.011 | 0.010 | 0.268 | 0.93 |
|  |  | phospholipid | -0.040 | 0.031 | 0.20 | -0.006 | 0.011 | 0.54 | -0.010 | 0.010 | 0.318 | 0.93 |
|  |  | cholesterol | -0.043 | 0.031 | 0.17 | -0.011 | 0.010 | 0.30 | -0.014 | 0.010 | 0.153 | 0.81 |
|  |  | cholesteryl ester | -0.043 | 0.031 | 0.17 | -0.010 | 0.010 | 0.32 | -0.014 | 0.010 | 0.166 | 0.81 |
|  |  | free cholesterol | -0.045 | 0.031 | 0.14 | -0.012 | 0.011 | 0.24 | -0.016 | 0.010 | 0.115 | 0.67 |
|  |  | triglycerides | -0.022 | 0.031 | 0.48 | 0.008 | 0.011 | 0.48 | 0.004 | 0.010 | 0.660 | 0.99 |
|  | M | particles | -0.054 | 0.031 | 0.08 | -0.006 | 0.011 | 0.62 | -0.011 | 0.011 | 0.293 | 0.93 |
|  |  | lipid | -0.055 | 0.031 | 0.08 | -0.005 | 0.011 | 0.65 | -0.011 | 0.011 | 0.303 | 0.93 |
|  |  | phospholipid | -0.045 | 0.031 | 0.15 | -0.005 | 0.011 | 0.65 | -0.010 | 0.011 | 0.361 | 0.97 |
|  |  | cholesterol | -0.068 | 0.031 | 0.03 | -0.006 | 0.011 | 0.57 | -0.013 | 0.011 | 0.206 | 0.83 |
|  |  | cholesteryl ester | -0.070 | 0.032 | 0.03 | -0.007 | 0.011 | 0.54 | -0.014 | 0.011 | 0.188 | 0.83 |
|  |  | free cholesterol | -0.059 | 0.031 | 0.06 | -0.005 | 0.011 | 0.63 | -0.012 | 0.011 | 0.271 | 0.93 |
|  |  | triglycerides | 0.034 | 0.032 | 0.29 | 0.005 | 0.011 | 0.66 | 0.008 | 0.011 | 0.444 | 0.99 |
|  | S | particles | -0.037 | 0.032 | 0.26 | -0.001 | 0.012 | 0.90 | -0.005 | 0.011 | 0.618 | 0.99 |
|  |  | lipid | -0.035 | 0.032 | 0.28 | -0.002 | 0.011 | 0.86 | -0.006 | 0.011 | 0.596 | 0.99 |
|  |  | phospholipid | -0.031 | 0.032 | 0.33 | -0.004 | 0.011 | 0.76 | -0.007 | 0.011 | 0.540 | 0.99 |
|  |  | cholesterol | -0.044 | 0.032 | 0.17 | -0.001 | 0.011 | 0.95 | -0.006 | 0.011 | 0.606 | 0.99 |
|  |  | cholesteryl ester | -0.036 | 0.032 | 0.27 | 0.000 | 0.011 | 0.97 | -0.004 | 0.011 | 0.688 | 0.99 |
|  |  | free cholesterol | -0.059 | 0.032 | 0.07 | -0.002 | 0.012 | 0.87 | -0.008 | 0.011 | 0.445 | 0.99 |
|  |  | triglycerides | 0.043 | 0.032 | 0.18 | 0.006 | 0.011 | 0.58 | 0.010 | 0.010 | 0.341 | 0.96 |
| VLDL diameter | | | 0.012 | 0.032 | 0.70 | -0.005 | 0.011 | 0.65 | -0.003 | 0.010 | 0.758 | 0.99 |
| LDL diameter | | | 0.030 | 0.031 | 0.32 | 0.041 | 0.011 | **0.0001** | 0.040 | 0.010 | **0.0001** | 0.004 |
| HDL diameter | | | -0.020 | 0.031 | 0.52 | -0.013 | 0.010 | 0.22 | -0.014 | 0.010 | 0.171 | 0.81 |
| Cholesterol | serum total | | -0.030 | 0.031 | 0.34 | -0.007 | 0.011 | 0.54 | -0.010 | 0.011 | 0.364 | 0.97 |
|  | VLDL | | 0.014 | 0.032 | 0.66 | 0.001 | 0.011 | 0.93 | 0.002 | 0.010 | 0.824 | 0.99 |
|  | remnant | | 0.004 | 0.032 | 0.89 | 0.000 | 0.011 | 0.99 | 0.000 | 0.011 | 0.971 | 0.99 |
|  | LDL | | -0.012 | 0.032 | 0.71 | -0.004 | 0.011 | 0.71 | -0.005 | 0.011 | 0.631 | 0.99 |
|  | HDL | | -0.054 | 0.030 | 0.08 | -0.013 | 0.011 | 0.23 | -0.018 | 0.010 | 0.084 | 0.57 |
|  | HDL2 | | -0.052 | 0.030 | 0.09 | -0.012 | 0.011 | 0.26 | -0.017 | 0.010 | 0.101 | 0.63 |
|  | HDL3 | | -0.055 | 0.030 | 0.07 | -0.015 | 0.011 | 0.19 | -0.019 | 0.010 | 0.064 | 0.49 |
|  | esterified | | -0.038 | 0.031 | 0.23 | -0.007 | 0.011 | 0.54 | -0.011 | 0.011 | 0.320 | 0.93 |
|  | free | | -0.016 | 0.031 | 0.61 | -0.005 | 0.011 | 0.66 | -0.006 | 0.011 | 0.559 | 0.99 |
| Triglycerides | serum total | | 0.013 | 0.032 | 0.67 | -0.002 | 0.011 | 0.83 | -0.001 | 0.010 | 0.944 | 0.99 |
|  | VLDL | | 0.008 | 0.032 | 0.80 | -0.005 | 0.011 | 0.62 | -0.004 | 0.010 | 0.700 | 0.99 |
|  | LDL | | 0.041 | 0.032 | 0.19 | 0.008 | 0.011 | 0.47 | 0.011 | 0.010 | 0.271 | 0.93 |
|  | HDL | | 0.009 | 0.032 | 0.79 | 0.005 | 0.011 | 0.65 | 0.005 | 0.011 | 0.603 | 0.99 |
| Total phosphoglycerides |  | | -0.037 | 0.031 | 0.23 | -0.008 | 0.011 | 0.49 | -0.011 | 0.011 | 0.288 | 0.93 |
| Phosphatidylcholine |  |  | -0.035 | 0.031 | 0.26 | -0.006 | 0.011 | 0.59 | -0.010 | 0.011 | 0.369 | 0.97 |
| Sphingomyelins |  |  | -0.025 | 0.031 | 0.42 | -0.009 | 0.011 | 0.42 | -0.011 | 0.010 | 0.299 | 0.93 |
| Total cholines |  |  | -0.033 | 0.031 | 0.29 | -0.007 | 0.011 | 0.52 | -0.010 | 0.011 | 0.336 | 0.96 |
| Apolipoprotein A-I |  |  | -0.073 | 0.031 | 0.02 | -0.013 | 0.011 | 0.25 | -0.020 | 0.011 | 0.055 | 0.48 |
| Apolipoprotein B |  |  | 0.000 | 0.032 | 0.99 | -0.003 | 0.011 | 0.77 | -0.003 | 0.011 | 0.780 | 0.99 |
| Apo-B / apoA-I |  |  | 0.035 | 0.032 | 0.28 | 0.004 | 0.011 | 0.70 | 0.007 | 0.010 | 0.478 | 0.99 |
| Fatty acids | total | | -0.023 | 0.032 | 0.46 | -0.005 | 0.011 | 0.67 | -0.007 | 0.011 | 0.511 | 0.99 |
|  | unsaturated | | -0.097 | 0.032 | **0.002** | -0.029 | 0.011 | 0.01 | -0.037 | 0.010 | **0.0004** | 0.01 |
|  | DHA (docosahexaenoic acid) | | -0.112 | 0.033 | **0.001** | -0.038 | 0.011 | **0.001** | -0.046 | 0.011 | **1.5E-05** | 0.001 |
|  | LA (linoleic acid) | | 0.004 | 0.032 | 0.90 | 0.005 | 0.011 | 0.69 | 0.004 | 0.011 | 0.678 | 0.99 |
|  | omega-3 | | -0.114 | 0.032 | **0.0005** | -0.028 | 0.011 | 0.01 | -0.038 | 0.011 | **0.0004** | 0.009 |
|  | omega-6 | | -0.029 | 0.032 | 0.36 | -0.004 | 0.011 | 0.73 | -0.007 | 0.011 | 0.526 | 0.99 |
|  | PUFA (polyunsaturated) | | -0.050 | 0.032 | 0.11 | -0.009 | 0.011 | 0.41 | -0.014 | 0.011 | 0.191 | 0.83 |
|  | MUFA (monounsaturated) | | -0.006 | 0.032 | 0.85 | 0.002 | 0.011 | 0.87 | 0.001 | 0.011 | 0.928 | 0.99 |
|  | SAFA (saturated) | | -0.013 | 0.032 | 0.69 | -0.005 | 0.011 | 0.65 | -0.006 | 0.011 | 0.576 | 0.99 |
|  | DHA% | | -0.118 | 0.032 | **0.0002** | -0.047 | 0.011 | **3.7E-05** | -0.055 | 0.011 | **2.8E-07** | 0.00004 |
|  | LA% | | 0.040 | 0.033 | 0.23 | 0.018 | 0.011 | 0.11 | 0.020 | 0.011 | 0.054 | 0.48 |
|  | omega-3% | | -0.121 | 0.032 | **0.0001** | -0.040 | 0.011 | **0.0004** | -0.050 | 0.011 | **3.4E-06** | 0.0003 |
|  | omega-6% | | -0.007 | 0.032 | 0.84 | 0.003 | 0.011 | 0.80 | 0.002 | 0.010 | 0.862 | 0.99 |
|  | PUFA% | | -0.047 | 0.032 | 0.15 | -0.011 | 0.011 | 0.32 | -0.015 | 0.010 | 0.157 | 0.81 |
|  | MUFA% | | 0.034 | 0.032 | 0.29 | 0.018 | 0.011 | 0.12 | 0.019 | 0.011 | 0.066 | 0.49 |
|  | SAFA% | | 0.049 | 0.031 | 0.12 | -0.003 | 0.011 | 0.77 | 0.003 | 0.011 | 0.798 | 0.99 |
| Glucose | | | -0.012 | 0.032 | 0.70 | 0.022 | 0.011 | 0.04 | 0.018 | 0.010 | 0.072 | 0.51 |
| Lactate | | | 0.018 | 0.032 | 0.58 | 0.032 | 0.011 | 0.003 | 0.031 | 0.010 | 0.003 | 0.05 |
| Pyruvate | | | 0.035 | 0.034 | 0.29 | 0.037 | 0.011 | **0.001** | 0.036 | 0.011 | **0.001** | 0.020 |
| Citrate | | | 0.028 | 0.033 | 0.41 | 0.012 | 0.011 | 0.26 | 0.013 | 0.010 | 0.186 | 0.83 |
| Glycerol | | | 0.066 | 0.033 | 0.04 | 0.021 | 0.013 | 0.10 | 0.027 | 0.012 | 0.023 | 0.30 |
| Alanine | | | -0.002 | 0.032 | 0.95 | 0.009 | 0.011 | 0.42 | 0.008 | 0.011 | 0.457 | 0.99 |
| Glutamine | | | -0.016 | 0.033 | 0.63 | 0.011 | 0.011 | 0.31 | 0.008 | 0.011 | 0.430 | 0.99 |
| Glycine | | | 0.021 | 0.031 | 0.51 | 0.040 | 0.011 | **0.0003** | 0.038 | 0.010 | **0.0003** | 0.009 |
| Histidine | | | -0.050 | 0.031 | 0.12 | -0.013 | 0.011 | 0.27 | -0.017 | 0.011 | 0.114 | 0.67 |
| Isoleucine | | | -0.003 | 0.032 | 0.92 | -0.009 | 0.011 | 0.41 | -0.008 | 0.010 | 0.419 | 0.99 |
| Leucine | | | -0.021 | 0.031 | 0.50 | -0.008 | 0.011 | 0.45 | -0.010 | 0.010 | 0.354 | 0.97 |
| Valine | | | -0.021 | 0.031 | 0.50 | -0.003 | 0.011 | 0.77 | -0.005 | 0.010 | 0.620 | 0.99 |
| Phenylalanine | | | 0.002 | 0.033 | 0.96 | 0.023 | 0.011 | 0.04 | 0.020 | 0.011 | 0.054 | 0.48 |
| Tyrosine | | | -0.039 | 0.032 | 0.22 | -0.004 | 0.011 | 0.69 | -0.008 | 0.011 | 0.435 | 0.99 |
| Acetate | | | 0.019 | 0.033 | 0.57 | 0.007 | 0.012 | 0.57 | 0.008 | 0.011 | 0.469 | 0.99 |
| Acetoacetate | | | 0.029 | 0.033 | 0.38 | 0.015 | 0.011 | 0.18 | 0.017 | 0.011 | 0.119 | 0.67 |
| 3-hydroxybutyrate | | | 0.003 | 0.033 | 0.93 | 0.024 | 0.011 | 0.03 | 0.022 | 0.011 | 0.042 | 0.44 |
| Creatinine | | | 0.054 | 0.033 | 0.10 | 0.006 | 0.010 | 0.56 | 0.011 | 0.010 | 0.290 | 0.93 |
| Albumin | | | -0.051 | 0.032 | 0.11 | 0.012 | 0.011 | 0.31 | 0.004 | 0.011 | 0.684 | 0.99 |
| GlycA (glycoprotein acetylation) | | | -0.006 | 0.031 | 0.83 | 0.018 | 0.011 | 0.12 | 0.015 | 0.011 | 0.160 | 0.81 |

| **APICAL RAREFACTIONS** | | | | | | | | | | | | |
| --- | --- | --- | --- | --- | --- | --- | --- | --- | --- | --- | --- | --- |
| **Metabolite** | | | **Parogene** | | | **Health 2000** | | | **Meta-analysis** | | | |
|  | | | Beta | SE | p | Beta | SE | p | Beta | SE | p | FDR |
| VLDL | XXL | particles | 0.034 | 0.052 | 0.51 | 0.024 | 1.630 | 0.10 | 0.025 | 0.014 | 0.08 | 0.46 |
|  |  | lipid | 0.037 | 0.052 | 0.47 | 0.026 | 1.746 | 0.08 | 0.026 | 0.014 | 0.06 | 0.46 |
|  |  | phospholipid | 0.023 | 0.052 | 0.66 | 0.025 | 1.690 | 0.09 | 0.025 | 0.014 | 0.08 | 0.46 |
|  |  | cholesterol | 0.045 | 0.052 | 0.39 | 0.024 | 1.651 | 0.10 | 0.026 | 0.014 | 0.07 | 0.46 |
|  |  | cholesteryl ester | 0.074 | 0.052 | 0.15 | 0.020 | 1.354 | 0.18 | 0.024 | 0.014 | 0.09 | 0.46 |
|  |  | free cholesterol | 0.012 | 0.052 | 0.82 | 0.025 | 1.688 | 0.09 | 0.024 | 0.014 | 0.09 | 0.46 |
|  |  | triglycerides | 0.036 | 0.052 | 0.49 | 0.026 | 1.764 | 0.08 | 0.027 | 0.014 | 0.06 | 0.46 |
|  | XL | particles | 0.015 | 0.052 | 0.77 | 0.022 | 1.522 | 0.13 | 0.022 | 0.014 | 0.12 | 0.49 |
|  |  | lipid | 0.020 | 0.052 | 0.70 | 0.023 | 1.609 | 0.11 | 0.023 | 0.014 | 0.10 | 0.47 |
|  |  | phospholipid | 0.015 | 0.052 | 0.77 | 0.025 | 1.711 | 0.09 | 0.024 | 0.014 | 0.08 | 0.46 |
|  |  | cholesterol | 0.024 | 0.052 | 0.65 | 0.024 | 1.634 | 0.10 | 0.024 | 0.014 | 0.09 | 0.46 |
|  |  | cholesteryl ester | 0.033 | 0.052 | 0.52 | 0.019 | 1.289 | 0.20 | 0.020 | 0.014 | 0.16 | 0.51 |
|  |  | free cholesterol | 0.012 | 0.052 | 0.82 | 0.025 | 1.709 | 0.09 | 0.024 | 0.014 | 0.09 | 0.46 |
|  |  | triglycerides | 0.017 | 0.051 | 0.74 | 0.023 | 1.546 | 0.12 | 0.022 | 0.014 | 0.11 | 0.49 |
|  | L | particles | 0.016 | 0.051 | 0.75 | 0.015 | 1.056 | 0.29 | 0.015 | 0.014 | 0.27 | 0.63 |
|  |  | lipid | 0.027 | 0.051 | 0.59 | 0.018 | 1.250 | 0.21 | 0.019 | 0.014 | 0.18 | 0.53 |
|  |  | phospholipid | 0.019 | 0.051 | 0.71 | 0.017 | 1.152 | 0.25 | 0.017 | 0.014 | 0.23 | 0.58 |
|  |  | cholesterol | 0.034 | 0.051 | 0.51 | 0.018 | 1.205 | 0.23 | 0.019 | 0.014 | 0.18 | 0.53 |
|  |  | cholesteryl ester | 0.057 | 0.051 | 0.27 | 0.016 | 1.108 | 0.27 | 0.019 | 0.014 | 0.17 | 0.53 |
|  |  | free cholesterol | 0.008 | 0.052 | 0.87 | 0.016 | 1.123 | 0.26 | 0.016 | 0.014 | 0.26 | 0.62 |
|  |  | triglycerides | 0.018 | 0.051 | 0.72 | 0.016 | 1.114 | 0.27 | 0.016 | 0.014 | 0.24 | 0.59 |
|  | M | particles | 0.022 | 0.051 | 0.66 | 0.009 | 0.614 | 0.54 | 0.010 | 0.014 | 0.48 | 0.77 |
|  |  | lipid | 0.037 | 0.051 | 0.47 | 0.014 | 0.965 | 0.33 | 0.016 | 0.014 | 0.26 | 0.62 |
|  |  | phospholipid | 0.031 | 0.051 | 0.55 | 0.012 | 0.816 | 0.41 | 0.013 | 0.014 | 0.34 | 0.68 |
|  |  | cholesterol | 0.056 | 0.052 | 0.28 | 0.014 | 0.976 | 0.33 | 0.017 | 0.014 | 0.22 | 0.58 |
|  |  | cholesteryl ester | 0.082 | 0.052 | 0.11 | 0.016 | 1.083 | 0.28 | 0.021 | 0.014 | 0.14 | 0.50 |
|  |  | free cholesterol | 0.024 | 0.051 | 0.64 | 0.010 | 0.680 | 0.50 | 0.011 | 0.014 | 0.43 | 0.77 |
|  |  | triglycerides | 0.019 | 0.051 | 0.71 | 0.013 | 0.903 | 0.37 | 0.013 | 0.014 | 0.33 | 0.68 |
|  | S | particles | 0.063 | 0.052 | 0.22 | 0.008 | 0.551 | 0.58 | 0.012 | 0.014 | 0.39 | 0.71 |
|  |  | lipid | 0.075 | 0.051 | 0.14 | 0.010 | 0.727 | 0.47 | 0.015 | 0.014 | 0.27 | 0.63 |
|  |  | phospholipid | 0.083 | 0.052 | 0.11 | 0.008 | 0.536 | 0.59 | 0.013 | 0.014 | 0.34 | 0.68 |
|  |  | cholesterol | 0.085 | 0.052 | 0.10 | 0.013 | 0.875 | 0.38 | 0.018 | 0.014 | 0.20 | 0.56 |
|  |  | cholesteryl ester | 0.090 | 0.052 | 0.08 | 0.014 | 0.964 | 0.34 | 0.020 | 0.014 | 0.16 | 0.51 |
|  |  | free cholesterol | 0.065 | 0.052 | 0.21 | 0.006 | 0.445 | 0.66 | 0.011 | 0.014 | 0.44 | 0.77 |
|  |  | triglycerides | 0.049 | 0.051 | 0.33 | 0.008 | 0.542 | 0.59 | 0.011 | 0.014 | 0.43 | 0.77 |
|  | XS | particles | 0.123 | 0.052 | 0.02 | 0.004 | 0.296 | 0.77 | 0.013 | 0.014 | 0.35 | 0.68 |
|  |  | lipid | 0.121 | 0.052 | 0.02 | 0.003 | 0.204 | 0.84 | 0.012 | 0.014 | 0.40 | 0.73 |
|  |  | phospholipid | 0.120 | 0.051 | 0.02 | 0.001 | 0.086 | 0.93 | 0.010 | 0.014 | 0.47 | 0.77 |
|  |  | cholesterol | 0.111 | 0.051 | 0.03 | 0.001 | 0.077 | 0.94 | 0.010 | 0.014 | 0.50 | 0.80 |
|  |  | cholesteryl ester | 0.118 | 0.051 | 0.02 | 0.004 | 0.303 | 0.76 | 0.013 | 0.014 | 0.34 | 0.68 |
|  |  | free cholesterol | 0.086 | 0.052 | 0.10 | -0.005 | -0.329 | 0.74 | 0.002 | 0.014 | 0.89 | 0.97 |
|  |  | triglycerides | 0.085 | 0.051 | 0.10 | 0.004 | 0.292 | 0.77 | 0.010 | 0.014 | 0.46 | 0.77 |
| IDL |  | particles | 0.093 | 0.051 | 0.07 | 0.002 | 0.121 | 0.90 | 0.009 | 0.014 | 0.54 | 0.80 |
|  |  | lipid | 0.082 | 0.051 | 0.11 | -0.001 | -0.043 | 0.97 | 0.006 | 0.014 | 0.69 | 0.91 |
|  |  | phospholipid | 0.087 | 0.051 | 0.09 | -0.002 | -0.123 | 0.90 | 0.005 | 0.014 | 0.72 | 0.93 |
|  |  | cholesterol | 0.063 | 0.051 | 0.22 | -0.001 | -0.085 | 0.93 | 0.004 | 0.014 | 0.79 | 0.95 |
|  |  | cholesteryl ester | 0.063 | 0.051 | 0.22 | 0.001 | 0.082 | 0.93 | 0.006 | 0.014 | 0.67 | 0.91 |
|  |  | free cholesterol | 0.070 | 0.051 | 0.17 | -0.005 | -0.308 | 0.76 | 0.001 | 0.014 | 0.93 | 0.98 |
|  |  | triglycerides | 0.100 | 0.051 | 0.05 | -0.007 | -0.479 | 0.63 | 0.001 | 0.014 | 0.95 | 0.98 |
| LDL | L | particles | 0.074 | 0.051 | 0.15 | 0.001 | 0.083 | 0.93 | 0.007 | 0.014 | 0.63 | 0.90 |
|  |  | lipid | 0.066 | 0.051 | 0.20 | -0.001 | -0.091 | 0.93 | 0.004 | 0.014 | 0.79 | 0.95 |
|  |  | phospholipid | 0.071 | 0.051 | 0.17 | 0.001 | 0.087 | 0.93 | 0.007 | 0.014 | 0.64 | 0.91 |
|  |  | cholesterol | 0.056 | 0.051 | 0.27 | -0.001 | -0.098 | 0.92 | 0.003 | 0.014 | 0.83 | 0.95 |
|  |  | cholesteryl ester | 0.056 | 0.051 | 0.27 | 0.000 | 0.018 | 0.99 | 0.005 | 0.014 | 0.75 | 0.94 |
|  |  | free cholesterol | 0.062 | 0.051 | 0.23 | -0.004 | -0.278 | 0.78 | 0.001 | 0.014 | 0.94 | 0.98 |
|  |  | triglycerides | 0.107 | 0.051 | 0.04 | -0.001 | -0.066 | 0.95 | 0.007 | 0.014 | 0.62 | 0.90 |
|  | M | particles | 0.058 | 0.052 | 0.26 | -0.002 | -0.113 | 0.91 | 0.003 | 0.014 | 0.84 | 0.95 |
|  |  | lipid | 0.051 | 0.052 | 0.32 | -0.004 | -0.278 | 0.78 | 0.000 | 0.014 | 0.99 | 0.998 |
|  |  | phospholipid | 0.052 | 0.052 | 0.31 | -0.001 | -0.035 | 0.97 | 0.004 | 0.014 | 0.80 | 0.95 |
|  |  | cholesterol | 0.043 | 0.052 | 0.41 | -0.004 | -0.284 | 0.78 | -0.001 | 0.014 | 0.97 | 0.98 |
|  |  | cholesteryl ester | 0.048 | 0.052 | 0.36 | -0.003 | -0.212 | 0.83 | 0.001 | 0.014 | 0.96 | 0.98 |
|  |  | free cholesterol | 0.028 | 0.051 | 0.59 | -0.006 | -0.423 | 0.67 | -0.004 | 0.014 | 0.80 | 0.95 |
|  |  | triglycerides | 0.097 | 0.051 | 0.06 | -0.004 | -0.253 | 0.80 | 0.003 | 0.013 | 0.80 | 0.95 |
|  | S | particles | 0.055 | 0.051 | 0.29 | -0.005 | -0.310 | 0.76 | 0.000 | 0.014 | 0.998 | 0.998 |
|  |  | lipid | 0.049 | 0.051 | 0.34 | -0.007 | -0.438 | 0.66 | -0.002 | 0.014 | 0.88 | 0.97 |
|  |  | phospholipid | 0.033 | 0.051 | 0.51 | -0.006 | -0.403 | 0.69 | -0.003 | 0.014 | 0.84 | 0.95 |
|  |  | cholesterol | 0.045 | 0.051 | 0.38 | -0.007 | -0.460 | 0.65 | -0.003 | 0.014 | 0.85 | 0.96 |
|  |  | cholesteryl ester | 0.052 | 0.051 | 0.31 | -0.005 | -0.359 | 0.72 | -0.001 | 0.014 | 0.95 | 0.98 |
|  |  | free cholesterol | 0.021 | 0.051 | 0.68 | -0.011 | -0.756 | 0.45 | -0.009 | 0.014 | 0.54 | 0.81 |
|  |  | triglycerides | 0.091 | 0.052 | 0.08 | -0.001 | -0.099 | 0.92 | 0.005 | 0.014 | 0.71 | 0.92 |
| HDL | XL | particles | 0.035 | 0.049 | 0.47 | -0.026 | -1.833 | 0.07 | -0.021 | 0.013 | 0.12 | 0.49 |
|  |  | lipid | 0.037 | 0.049 | 0.45 | -0.027 | -1.922 | 0.05 | -0.022 | 0.013 | 0.10 | 0.47 |
|  |  | phospholipid | 0.036 | 0.049 | 0.46 | -0.024 | -1.788 | 0.07 | -0.020 | 0.013 | 0.13 | 0.49 |
|  |  | cholesterol | 0.035 | 0.049 | 0.48 | -0.030 | -2.120 | 0.03 | -0.025 | 0.014 | 0.07 | 0.46 |
|  |  | cholesteryl ester | 0.038 | 0.050 | 0.44 | -0.029 | -2.049 | 0.04 | -0.024 | 0.014 | 0.08 | 0.46 |
|  |  | free cholesterol | 0.022 | 0.049 | 0.66 | -0.031 | -2.225 | 0.03 | -0.027 | 0.013 | 0.04 | 0.46 |
|  |  | triglycerides | 0.024 | 0.052 | 0.65 | 0.008 | 0.522 | 0.60 | 0.009 | 0.014 | 0.53 | 0.80 |
|  | L | particles | -0.030 | 0.048 | 0.53 | -0.019 | -1.332 | 0.18 | -0.020 | 0.013 | 0.15 | 0.50 |
|  |  | lipid | -0.026 | 0.049 | 0.60 | -0.020 | -1.434 | 0.15 | -0.020 | 0.013 | 0.13 | 0.49 |
|  |  | phospholipid | -0.024 | 0.049 | 0.62 | -0.018 | -1.271 | 0.20 | -0.018 | 0.013 | 0.17 | 0.53 |
|  |  | cholesterol | -0.033 | 0.049 | 0.50 | -0.022 | -1.576 | 0.12 | -0.023 | 0.013 | 0.09 | 0.46 |
|  |  | cholesteryl ester | -0.033 | 0.049 | 0.50 | -0.021 | -1.548 | 0.12 | -0.022 | 0.013 | 0.09 | 0.46 |
|  |  | free cholesterol | -0.039 | 0.049 | 0.42 | -0.022 | -1.614 | 0.11 | -0.024 | 0.013 | 0.08 | 0.46 |
|  |  | triglycerides | -0.002 | 0.049 | 0.96 | -0.005 | -0.342 | 0.73 | -0.005 | 0.014 | 0.73 | 0.94 |
|  | M | particles | -0.043 | 0.049 | 0.38 | 0.002 | 0.132 | 0.90 | -0.002 | 0.014 | 0.90 | 0.97 |
|  |  | lipid | -0.050 | 0.049 | 0.31 | 0.001 | 0.083 | 0.93 | -0.003 | 0.014 | 0.83 | 0.95 |
|  |  | phospholipid | -0.037 | 0.049 | 0.45 | 0.000 | -0.022 | 0.98 | -0.003 | 0.014 | 0.81 | 0.95 |
|  |  | cholesterol | -0.070 | 0.049 | 0.16 | -0.001 | -0.079 | 0.94 | -0.007 | 0.014 | 0.63 | 0.90 |
|  |  | cholesteryl ester | -0.073 | 0.050 | 0.14 | 0.000 | 0.007 | 0.99 | -0.006 | 0.014 | 0.68 | 0.91 |
|  |  | free cholesterol | -0.055 | 0.049 | 0.26 | -0.006 | -0.401 | 0.69 | -0.010 | 0.014 | 0.47 | 0.77 |
|  |  | triglycerides | 0.099 | 0.052 | 0.06 | 0.030 | 1.971 | 0.05 | 0.035 | 0.014 | 0.02 | 0.26 |
|  | S | particles | -0.072 | 0.051 | 0.16 | 0.009 | 0.561 | 0.57 | 0.002 | 0.015 | 0.89 | 0.97 |
|  |  | lipid | -0.071 | 0.051 | 0.16 | 0.008 | 0.514 | 0.61 | 0.001 | 0.015 | 0.93 | 0.98 |
|  |  | phospholipid | -0.064 | 0.051 | 0.21 | 0.012 | 0.807 | 0.42 | 0.006 | 0.014 | 0.68 | 0.91 |
|  |  | cholesterol | -0.079 | 0.051 | 0.12 | -0.003 | -0.226 | 0.82 | -0.010 | 0.015 | 0.51 | 0.80 |
|  |  | cholesteryl ester | -0.057 | 0.051 | 0.26 | -0.005 | -0.322 | 0.75 | -0.009 | 0.014 | 0.53 | 0.80 |
|  |  | free cholesterol | -0.133 | 0.051 | 0.01 | 0.002 | 0.133 | 0.89 | -0.009 | 0.015 | 0.53 | 0.80 |
|  |  | triglycerides | 0.046 | 0.051 | 0.37 | 0.024 | 1.657 | 0.10 | 0.026 | 0.014 | 0.07 | 0.46 |
| VLDL diameter | | | 0.016 | 0.051 | 0.75 | 0.025 | 1.735 | 0.08 | 0.024 | 0.014 | 0.08 | 0.46 |
| LDL diameter | | | 0.072 | 0.049 | 0.14 | 0.032 | 2.203 | 0.03 | 0.035 | 0.014 | 0.01 | 0.26 |
| HDL diameter | | | 0.007 | 0.049 | 0.88 | -0.021 | -1.555 | 0.12 | -0.019 | 0.013 | 0.15 | 0.50 |
| Cholesterol | serum total | | 0.039 | 0.050 | 0.44 | -0.007 | -0.446 | 0.66 | -0.003 | 0.014 | 0.84 | 0.95 |
|  | VLDL | | 0.084 | 0.052 | 0.11 | 0.013 | 0.900 | 0.37 | 0.018 | 0.014 | 0.19 | 0.55 |
|  | remnant | | 0.086 | 0.052 | 0.10 | 0.009 | 0.589 | 0.56 | 0.014 | 0.014 | 0.31 | 0.65 |
|  | LDL | | 0.048 | 0.051 | 0.35 | -0.005 | -0.349 | 0.73 | -0.001 | 0.014 | 0.94 | 0.98 |
|  | HDL | | -0.052 | 0.048 | 0.28 | -0.019 | -1.352 | 0.18 | -0.022 | 0.014 | 0.11 | 0.48 |
|  | HDL2 | | -0.052 | 0.048 | 0.28 | -0.018 | -1.261 | 0.21 | -0.021 | 0.014 | 0.13 | 0.49 |
|  | HDL3 | | -0.037 | 0.049 | 0.45 | -0.025 | -1.700 | 0.09 | -0.026 | 0.014 | 0.06 | 0.46 |
|  | esterified | | 0.031 | 0.051 | 0.54 | -0.007 | -0.478 | 0.63 | -0.004 | 0.014 | 0.78 | 0.95 |
|  | free | | 0.048 | 0.051 | 0.35 | -0.005 | -0.319 | 0.75 | -0.001 | 0.014 | 0.97 | 0.98 |
| Triglycerides | serum total | | 0.049 | 0.051 | 0.34 | 0.014 | 0.996 | 0.32 | 0.017 | 0.014 | 0.22 | 0.58 |
|  | VLDL | | 0.036 | 0.051 | 0.48 | 0.015 | 1.038 | 0.30 | 0.016 | 0.014 | 0.23 | 0.58 |
|  | LDL | | 0.105 | 0.051 | 0.04 | -0.004 | -0.291 | 0.77 | 0.004 | 0.014 | 0.79 | 0.95 |
|  | HDL | | 0.050 | 0.052 | 0.34 | 0.015 | 0.991 | 0.32 | 0.017 | 0.014 | 0.22 | 0.58 |
| Total phosphoglycerides | | | 0.023 | 0.050 | 0.64 | -0.007 | -0.453 | 0.65 | -0.004 | 0.014 | 0.76 | 0.95 |
| Phosphatidylcholine | | | 0.032 | 0.050 | 0.53 | -0.008 | -0.537 | 0.59 | -0.005 | 0.014 | 0.74 | 0.94 |
| Sphingomyelins | | | 0.003 | 0.049 | 0.96 | -0.012 | -0.798 | 0.43 | -0.011 | 0.014 | 0.45 | 0.77 |
| Total cholines | | | 0.023 | 0.050 | 0.64 | -0.009 | -0.597 | 0.55 | -0.006 | 0.014 | 0.66 | 0.91 |
| Apolipoprotein A-I | | | -0.055 | 0.048 | 0.26 | -0.012 | -0.780 | 0.44 | -0.015 | 0.014 | 0.28 | 0.63 |
| Apolipoprotein B | | | 0.073 | 0.052 | 0.16 | 0.007 | 0.502 | 0.62 | 0.012 | 0.014 | 0.39 | 0.71 |
| Apo-B / apoA-I | | | 0.094 | 0.051 | 0.06 | 0.014 | 0.977 | 0.33 | 0.020 | 0.014 | 0.15 | 0.50 |
| Fatty acids | total | | 0.033 | 0.052 | 0.52 | 0.004 | 0.239 | 0.81 | 0.006 | 0.014 | 0.68 | 0.91 |
|  | unsaturated | | -0.097 | 0.051 | 0.06 | -0.038 | -2.558 | 0.01 | -0.042 | 0.014 | 0.00 | 0.16 |
|  | DHA (docosahexaenoic acid) | | -0.078 | 0.052 | 0.13 | -0.025 | -1.649 | 0.10 | -0.029 | 0.014 | 0.05 | 0.46 |
|  | LA (linoleic acid) | | 0.045 | 0.052 | 0.39 | -0.002 | -0.103 | 0.92 | 0.002 | 0.015 | 0.88 | 0.97 |
|  | omega-3 | | -0.050 | 0.052 | 0.34 | -0.014 | -0.960 | 0.34 | -0.017 | 0.014 | 0.23 | 0.58 |
|  | omega-6 | | 0.027 | 0.051 | 0.60 | -0.005 | -0.331 | 0.74 | -0.002 | 0.015 | 0.87 | 0.97 |
|  | PUFA (polyunsaturated) | | 0.011 | 0.051 | 0.83 | -0.008 | -0.517 | 0.60 | -0.006 | 0.015 | 0.66 | 0.91 |
|  | MUFA (monounsaturated) | | 0.032 | 0.051 | 0.54 | 0.009 | 0.596 | 0.55 | 0.011 | 0.014 | 0.46 | 0.77 |
|  | SAFA (saturated) | | 0.051 | 0.052 | 0.33 | 0.010 | 0.653 | 0.51 | 0.013 | 0.014 | 0.37 | 0.71 |
|  | DHA% | | -0.112 | 0.051 | 0.03 | -0.043 | -2.875 | 0.00 | -0.048 | 0.014 | **0.001** | 0.16 |
|  | LA% | | 0.021 | 0.051 | 0.68 | -0.013 | -0.885 | 0.38 | -0.011 | 0.014 | 0.46 | 0.77 |
|  | omega-3% | | -0.077 | 0.051 | 0.13 | -0.037 | -2.440 | 0.01 | -0.040 | 0.014 | 0.01 | 0.24 |
|  | omega-6% | | -0.018 | 0.051 | 0.72 | -0.022 | -1.501 | 0.13 | -0.022 | 0.014 | 0.12 | 0.49 |
|  | PUFA% | | -0.043 | 0.050 | 0.39 | -0.031 | -2.127 | 0.03 | -0.032 | 0.014 | 0.02 | 0.35 |
|  | MUFA% | | 0.024 | 0.050 | 0.63 | 0.016 | 1.102 | 0.27 | 0.017 | 0.014 | 0.23 | 0.58 |
|  | SAFA% | | 0.080 | 0.051 | 0.12 | 0.040 | 2.617 | 0.01 | 0.043 | 0.015 | 0.00 | 0.16 |
| Glucose | | | -0.004 | 0.049 | 0.93 | 0.022 | 1.512 | 0.13 | 0.020 | 0.014 | 0.15 | 0.50 |
| Lactate | | | 0.134 | 0.051 | 0.01 | 0.027 | 1.828 | 0.07 | 0.035 | 0.014 | 0.01 | 0.26 |
| Pyruvate | | | 0.181 | 0.051 | 0.00 | 0.025 | 1.691 | 0.09 | 0.037 | 0.014 | 0.01 | 0.26 |
| Citrate | | | 0.034 | 0.052 | 0.51 | -0.010 | -0.710 | 0.48 | -0.007 | 0.014 | 0.61 | 0.89 |
| Glycerol | | | 0.138 | 0.052 | 0.01 | 0.002 | 0.152 | 0.88 | 0.014 | 0.015 | 0.35 | 0.68 |
| Alanine | | | 0.144 | 0.051 | 0.005 | -0.002 | -0.157 | 0.87 | 0.009 | 0.014 | 0.52 | 0.80 |
| Glutamine | | | 0.081 | 0.051 | 0.11 | 0.009 | 0.595 | 0.55 | 0.015 | 0.014 | 0.31 | 0.65 |
| Glycine | | | 0.056 | 0.049 | 0.26 | 0.027 | 1.822 | 0.07 | 0.029 | 0.014 | 0.04 | 0.46 |
| Histidine | | | -0.065 | 0.050 | 0.20 | -0.008 | -0.520 | 0.60 | -0.012 | 0.014 | 0.39 | 0.71 |
| Isoleucine | | | 0.077 | 0.050 | 0.13 | 0.009 | 0.643 | 0.52 | 0.014 | 0.014 | 0.30 | 0.65 |
| Leucine | | | 0.083 | 0.050 | 0.10 | 0.008 | 0.568 | 0.57 | 0.014 | 0.014 | 0.31 | 0.66 |
| Valine | | | 0.033 | 0.050 | 0.50 | 0.007 | 0.461 | 0.64 | 0.009 | 0.014 | 0.53 | 0.80 |
| Phenylalanine | | | 0.129 | 0.051 | 0.01 | 0.012 | 0.811 | 0.42 | 0.021 | 0.014 | 0.14 | 0.50 |
| Tyrosine | | | -0.003 | 0.050 | 0.96 | 0.017 | 1.144 | 0.25 | 0.015 | 0.014 | 0.28 | 0.63 |
| Acetate | | | 0.000 | 0.052 | 1.00 | -0.007 | -0.439 | 0.66 | -0.006 | 0.015 | 0.67 | 0.91 |
| Acetoacetate | | | 0.038 | 0.053 | 0.47 | -0.019 | -1.287 | 0.20 | -0.015 | 0.014 | 0.30 | 0.65 |
| 3-hydroxybutyrate | | | -0.002 | 0.052 | 0.98 | -0.014 | -0.918 | 0.36 | -0.013 | 0.015 | 0.37 | 0.71 |
| Creatinine | | | 0.017 | 0.051 | 0.73 | -0.037 | -2.680 | 0.01 | -0.033 | 0.013 | 0.01 | 0.26 |
| Albumin | | | -0.053 | 0.050 | 0.29 | 0.003 | 0.202 | 0.84 | -0.002 | 0.014 | 0.91 | 0.98 |
| GlycA (glycoprotein acetylation) | | | 0.039 | 0.049 | 0.42 | 0.023 | 1.508 | 0.13 | 0.024 | 0.014 | 0.09 | 0.46 |

| **ROOT FILLINGS** | | | | | | | | | | | | |
| --- | --- | --- | --- | --- | --- | --- | --- | --- | --- | --- | --- | --- |
| **Metabolite** | | | **Parogene** | | | **Health 2000** | | | **Meta-analysis** | | | |
|  | | | Beta | SE | p | Beta | SE | p | Beta | SE | p | FDR |
| VLDL | XXL | particles | 0.039 | 0.023 | 0.09 | 0.018 | 0.007 | 0.01 | 0.020 | 0.007 | 0.003 | 0.01 |
|  |  | lipid | 0.040 | 0.023 | 0.08 | 0.018 | 0.007 | 0.01 | 0.020 | 0.007 | 0.003 | 0.01 |
|  |  | phospholipid | 0.034 | 0.023 | 0.15 | 0.019 | 0.007 | 0.01 | 0.020 | 0.007 | 0.003 | 0.01 |
|  |  | cholesterol | 0.035 | 0.023 | 0.13 | 0.018 | 0.007 | 0.01 | 0.019 | 0.007 | 0.004 | 0.01 |
|  |  | cholesteryl ester | 0.037 | 0.023 | 0.11 | 0.017 | 0.007 | 0.02 | 0.019 | 0.007 | 0.01 | 0.01 |
|  |  | free cholesterol | 0.031 | 0.023 | 0.18 | 0.018 | 0.007 | 0.01 | 0.019 | 0.007 | 0.005 | 0.01 |
|  |  | triglycerides | 0.041 | 0.023 | 0.08 | 0.019 | 0.007 | 0.01 | 0.021 | 0.007 | **0.002** | 0.01 |
|  | XL | particles | 0.039 | 0.023 | 0.09 | 0.020 | 0.007 | 0.004 | 0.022 | 0.007 | **0.001** | 0.004 |
|  |  | lipid | 0.043 | 0.023 | 0.07 | 0.020 | 0.007 | 0.004 | 0.022 | 0.007 | **0.001** | 0.004 |
|  |  | phospholipid | 0.035 | 0.023 | 0.13 | 0.020 | 0.007 | 0.005 | 0.021 | 0.007 | **0.002** | 0.01 |
|  |  | cholesterol | 0.036 | 0.023 | 0.12 | 0.019 | 0.007 | 0.01 | 0.020 | 0.007 | **0.002** | 0.01 |
|  |  | cholesteryl ester | 0.040 | 0.023 | 0.08 | 0.018 | 0.007 | 0.01 | 0.020 | 0.007 | **0.0026** | 0.01 |
|  |  | free cholesterol | 0.031 | 0.023 | 0.19 | 0.019 | 0.007 | 0.01 | 0.020 | 0.007 | 0.003 | 0.01 |
|  |  | triglycerides | 0.043 | 0.023 | 0.06 | 0.020 | 0.007 | 0.004 | 0.022 | 0.007 | **0.001** | 0.004 |
|  | L | particles | 0.044 | 0.023 | 0.05 | 0.020 | 0.007 | 0.004 | 0.022 | 0.007 | **0.001** | 0.004 |
|  |  | lipid | 0.053 | 0.023 | 0.02 | 0.022 | 0.007 | **0.002** | 0.024 | 0.007 | **0.0003** | 0.002 |
|  |  | phospholipid | 0.045 | 0.023 | 0.05 | 0.020 | 0.007 | 0.003 | 0.022 | 0.007 | **0.001** | 0.004 |
|  |  | cholesterol | 0.047 | 0.023 | 0.04 | 0.021 | 0.007 | **0.0026** | 0.023 | 0.007 | **0.001** | 0.004 |
|  |  | cholesteryl ester | 0.053 | 0.023 | 0.02 | 0.021 | 0.007 | **0.002** | 0.024 | 0.007 | **0.0003** | 0.002 |
|  |  | free cholesterol | 0.038 | 0.023 | 0.10 | 0.019 | 0.007 | 0.01 | 0.021 | 0.007 | **0.002** | 0.01 |
|  |  | triglycerides | 0.050 | 0.023 | 0.03 | 0.021 | 0.007 | **0.0026** | 0.023 | 0.007 | **0.0004** | 0.003 |
|  | M | particles | 0.044 | 0.023 | 0.06 | 0.020 | 0.007 | 0.003 | 0.022 | 0.007 | **0.001** | 0.004 |
|  |  | lipid | 0.052 | 0.023 | 0.02 | 0.022 | 0.007 | **0.001** | 0.025 | 0.007 | **0.0002** | 0.002 |
|  |  | phospholipid | 0.046 | 0.023 | 0.04 | 0.021 | 0.007 | **0.002** | 0.023 | 0.007 | **0.0004** | 0.003 |
|  |  | cholesterol | 0.047 | 0.023 | 0.04 | 0.022 | 0.007 | **0.002** | 0.024 | 0.007 | **0.0003** | 0.002 |
|  |  | cholesteryl ester | 0.047 | 0.023 | 0.04 | 0.022 | 0.007 | **0.002** | 0.024 | 0.007 | **0.0003** | 0.002 |
|  |  | free cholesterol | 0.042 | 0.023 | 0.07 | 0.021 | 0.007 | 0.003 | 0.022 | 0.007 | **0.001** | 0.004 |
|  |  | triglycerides | 0.049 | 0.023 | 0.03 | 0.023 | 0.007 | **0.001** | 0.025 | 0.007 | **0.0002** | 0.002 |
|  | S | particles | 0.041 | 0.023 | 0.08 | 0.023 | 0.007 | **0.001** | 0.025 | 0.007 | **0.0002** | 0.002 |
|  |  | lipid | 0.044 | 0.023 | 0.06 | 0.024 | 0.007 | **0.001** | 0.026 | 0.007 | **0.0001** | 0.002 |
|  |  | phospholipid | 0.043 | 0.023 | 0.07 | 0.025 | 0.007 | **0.0004** | 0.026 | 0.007 | **0.0001** | 0.002 |
|  |  | cholesterol | 0.042 | 0.023 | 0.08 | 0.022 | 0.007 | **0.002** | 0.023 | 0.007 | **0.0005** | 0.003 |
|  |  | cholesteryl ester | 0.040 | 0.023 | 0.09 | 0.020 | 0.007 | 0.01 | 0.021 | 0.007 | **0.002** | 0.01 |
|  |  | free cholesterol | 0.038 | 0.023 | 0.11 | 0.023 | 0.007 | **0.001** | 0.024 | 0.007 | **0.0003** | 0.002 |
|  |  | triglycerides | 0.040 | 0.023 | 0.08 | 0.023 | 0.007 | **0.001** | 0.024 | 0.007 | **0.0002** | 0.002 |
|  | XS | particles | 0.013 | 0.023 | 0.59 | 0.016 | 0.007 | 0.03 | 0.015 | 0.007 | 0.02 | 0.049 |
|  |  | lipid | 0.011 | 0.023 | 0.65 | 0.015 | 0.007 | 0.03 | 0.015 | 0.007 | 0.03 | 0.06 |
|  |  | phospholipid | 0.003 | 0.023 | 0.89 | 0.012 | 0.007 | 0.10 | 0.011 | 0.007 | 0.10 | 0.19 |
|  |  | cholesterol | 0.011 | 0.023 | 0.64 | 0.010 | 0.007 | 0.15 | 0.010 | 0.007 | 0.13 | 0.23 |
|  |  | cholesteryl ester | 0.013 | 0.023 | 0.57 | 0.009 | 0.007 | 0.19 | 0.010 | 0.007 | 0.16 | 0.26 |
|  |  | free cholesterol | 0.006 | 0.023 | 0.80 | 0.011 | 0.007 | 0.12 | 0.011 | 0.007 | 0.12 | 0.21 |
|  |  | triglycerides | 0.015 | 0.023 | 0.52 | 0.022 | 0.007 | **0.001** | 0.021 | 0.007 | **0.001** | 0.004 |
| IDL | | particles | 0.010 | 0.023 | 0.68 | 0.007 | 0.007 | 0.36 | 0.007 | 0.007 | 0.32 | 0.42 |
|  |  | lipid | 0.007 | 0.023 | 0.77 | 0.007 | 0.007 | 0.34 | 0.007 | 0.007 | 0.32 | 0.42 |
|  |  | phospholipid | 0.003 | 0.023 | 0.90 | 0.005 | 0.007 | 0.52 | 0.004 | 0.007 | 0.51 | 0.60 |
|  |  | cholesterol | 0.011 | 0.023 | 0.63 | 0.005 | 0.007 | 0.45 | 0.006 | 0.007 | 0.38 | 0.48 |
|  |  | cholesteryl ester | 0.018 | 0.023 | 0.45 | 0.007 | 0.007 | 0.34 | 0.008 | 0.007 | 0.25 | 0.37 |
|  |  | free cholesterol | -0.003 | 0.023 | 0.89 | 0.001 | 0.007 | 0.88 | 0.001 | 0.007 | 0.92 | 0.94 |
|  |  | triglycerides | -0.022 | 0.023 | 0.33 | 0.011 | 0.007 | 0.09 | 0.009 | 0.006 | 0.18 | 0.29 |
| LDL | L | particles | 0.012 | 0.023 | 0.59 | 0.007 | 0.007 | 0.35 | 0.007 | 0.007 | 0.30 | 0.41 |
|  |  | lipid | 0.010 | 0.023 | 0.66 | 0.007 | 0.007 | 0.32 | 0.007 | 0.007 | 0.28 | 0.39 |
|  |  | phospholipid | 0.014 | 0.023 | 0.55 | 0.007 | 0.007 | 0.33 | 0.008 | 0.007 | 0.27 | 0.38 |
|  |  | cholesterol | 0.013 | 0.023 | 0.57 | 0.007 | 0.007 | 0.34 | 0.007 | 0.007 | 0.28 | 0.39 |
|  |  | cholesteryl ester | 0.017 | 0.023 | 0.46 | 0.008 | 0.007 | 0.25 | 0.009 | 0.007 | 0.19 | 0.30 |
|  |  | free cholesterol | 0.003 | 0.023 | 0.88 | 0.002 | 0.007 | 0.82 | 0.002 | 0.007 | 0.80 | 0.84 |
|  |  | triglycerides | -0.017 | 0.023 | 0.47 | 0.007 | 0.007 | 0.28 | 0.005 | 0.006 | 0.40 | 0.50 |
|  | M | particles | 0.012 | 0.023 | 0.62 | 0.007 | 0.007 | 0.30 | 0.008 | 0.007 | 0.25 | 0.37 |
|  |  | lipid | 0.010 | 0.023 | 0.68 | 0.008 | 0.007 | 0.26 | 0.008 | 0.007 | 0.23 | 0.35 |
|  |  | phospholipid | 0.015 | 0.023 | 0.52 | 0.010 | 0.007 | 0.15 | 0.011 | 0.007 | 0.12 | 0.21 |
|  |  | cholesterol | 0.013 | 0.023 | 0.59 | 0.007 | 0.007 | 0.31 | 0.008 | 0.007 | 0.26 | 0.38 |
|  |  | cholesteryl ester | 0.015 | 0.023 | 0.52 | 0.008 | 0.007 | 0.29 | 0.008 | 0.007 | 0.23 | 0.35 |
|  |  | free cholesterol | 0.006 | 0.023 | 0.80 | 0.005 | 0.007 | 0.48 | 0.005 | 0.007 | 0.46 | 0.54 |
|  |  | triglycerides | -0.024 | 0.023 | 0.30 | 0.006 | 0.007 | 0.36 | 0.004 | 0.006 | 0.56 | 0.65 |
|  | S | particles | 0.016 | 0.023 | 0.50 | 0.008 | 0.007 | 0.29 | 0.008 | 0.007 | 0.23 | 0.35 |
|  |  | lipid | 0.014 | 0.023 | 0.55 | 0.008 | 0.007 | 0.28 | 0.008 | 0.007 | 0.22 | 0.35 |
|  |  | phospholipid | 0.018 | 0.023 | 0.43 | 0.009 | 0.007 | 0.21 | 0.010 | 0.007 | 0.15 | 0.26 |
|  |  | cholesterol | 0.013 | 0.023 | 0.57 | 0.006 | 0.007 | 0.39 | 0.007 | 0.007 | 0.32 | 0.42 |
|  |  | cholesteryl ester | 0.013 | 0.023 | 0.57 | 0.006 | 0.007 | 0.39 | 0.007 | 0.007 | 0.32 | 0.42 |
|  |  | free cholesterol | 0.014 | 0.023 | 0.55 | 0.006 | 0.007 | 0.44 | 0.006 | 0.007 | 0.36 | 0.47 |
|  |  | triglycerides | 0.006 | 0.023 | 0.80 | 0.013 | 0.007 | 0.06 | 0.013 | 0.007 | 0.06 | 0.12 |
| HDL | XL | particles | -0.037 | 0.022 | 0.09 | -0.020 | 0.007 | 0.003 | -0.021 | 0.006 | **0.001** | 0.004 |
|  |  | lipid | -0.035 | 0.022 | 0.11 | -0.020 | 0.007 | **0.0025** | -0.021 | 0.006 | **0.001** | 0.004 |
|  |  | phospholipid | -0.045 | 0.022 | 0.04 | -0.021 | 0.007 | **0.001** | -0.023 | 0.006 | **0.0002** | 0.002 |
|  |  | cholesterol | -0.026 | 0.022 | 0.24 | -0.019 | 0.007 | 0.01 | -0.019 | 0.007 | 0.003 | 0.01 |
|  |  | cholesteryl ester | -0.023 | 0.022 | 0.30 | -0.018 | 0.007 | 0.01 | -0.018 | 0.007 | 0.01 | 0.01 |
|  |  | free cholesterol | -0.034 | 0.022 | 0.12 | -0.020 | 0.007 | **0.002** | -0.022 | 0.006 | **0.001** | 0.004 |
|  |  | triglycerides | 0.006 | 0.024 | 0.81 | -0.004 | 0.007 | 0.58 | -0.003 | 0.007 | 0.64 | 0.71 |
|  | L | particles | -0.056 | 0.022 | 0.01 | -0.017 | 0.007 | 0.01 | -0.021 | 0.006 | **0.001** | 0.004 |
|  |  | lipid | -0.055 | 0.022 | 0.01 | -0.018 | 0.007 | 0.01 | -0.021 | 0.006 | **0.001** | 0.004 |
|  |  | phospholipid | -0.052 | 0.022 | 0.02 | -0.016 | 0.007 | 0.02 | -0.019 | 0.006 | 0.003 | 0.01 |
|  |  | cholesterol | -0.060 | 0.022 | 0.01 | -0.020 | 0.007 | 0.003 | -0.023 | 0.006 | **0.0002** | 0.002 |
|  |  | cholesteryl ester | -0.060 | 0.022 | 0.01 | -0.020 | 0.007 | 0.003 | -0.023 | 0.006 | **0.0003** | 0.002 |
|  |  | free cholesterol | -0.062 | 0.022 | 0.00 | -0.020 | 0.007 | **0.0025** | -0.024 | 0.006 | **0.0002** | 0.002 |
|  |  | triglycerides | -0.032 | 0.022 | 0.15 | -0.009 | 0.007 | 0.17 | -0.011 | 0.007 | 0.08 | 0.16 |
|  | M | particles | -0.011 | 0.022 | 0.62 | 0.004 | 0.007 | 0.59 | 0.003 | 0.007 | 0.71 | 0.77 |
|  |  | lipid | -0.013 | 0.022 | 0.55 | 0.004 | 0.007 | 0.59 | 0.002 | 0.007 | 0.74 | 0.79 |
|  |  | phospholipid | -0.012 | 0.022 | 0.57 | 0.004 | 0.007 | 0.59 | 0.002 | 0.007 | 0.74 | 0.79 |
|  |  | cholesterol | -0.020 | 0.022 | 0.37 | 0.001 | 0.007 | 0.94 | -0.001 | 0.007 | 0.83 | 0.87 |
|  |  | cholesteryl ester | -0.019 | 0.022 | 0.39 | 0.000 | 0.007 | 0.95 | -0.001 | 0.007 | 0.84 | 0.87 |
|  |  | free cholesterol | -0.021 | 0.022 | 0.33 | 0.000 | 0.007 | 0.98 | -0.002 | 0.007 | 0.74 | 0.79 |
|  |  | triglycerides | 0.049 | 0.023 | 0.04 | 0.025 | 0.007 | **0.001** | 0.027 | 0.007 | **0.0001** | 0.002 |
|  | S | particles | 0.020 | 0.023 | 0.38 | 0.012 | 0.007 | 0.09 | 0.013 | 0.007 | 0.06 | 0.12 |
|  |  | lipid | 0.017 | 0.023 | 0.45 | 0.011 | 0.007 | 0.15 | 0.011 | 0.007 | 0.11 | 0.20 |
|  |  | phospholipid | 0.020 | 0.023 | 0.39 | 0.011 | 0.007 | 0.13 | 0.012 | 0.007 | 0.09 | 0.17 |
|  |  | cholesterol | 0.000 | 0.023 | 1.00 | 0.006 | 0.007 | 0.41 | 0.005 | 0.007 | 0.44 | 0.53 |
|  |  | cholesteryl ester | -0.001 | 0.023 | 0.97 | 0.005 | 0.007 | 0.46 | 0.005 | 0.007 | 0.48 | 0.57 |
|  |  | free cholesterol | 0.002 | 0.023 | 0.93 | 0.007 | 0.007 | 0.37 | 0.006 | 0.007 | 0.38 | 0.47 |
|  |  | triglycerides | 0.045 | 0.023 | 0.05 | 0.022 | 0.007 | **0.002** | 0.024 | 0.007 | **0.0004** | 0.003 |
| VLDL diameter | | | 0.064 | 0.023 | 0.01 | 0.022 | 0.007 | **0.002** | 0.025 | 0.007 | **0.0001** | 0.002 |
| LDL diameter | | | -0.033 | 0.022 | 0.14 | -0.014 | 0.007 | 0.05 | -0.015 | 0.007 | 0.02 | 0.04 |
| HDL diameter | | | -0.055 | 0.022 | 0.01 | -0.022 | 0.007 | **0.001** | -0.025 | 0.006 | **0.0001** | 0.002 |
| Cholesterol | serum total | | 0.000 | 0.023 | 1.00 | 0.007 | 0.007 | 0.35 | 0.006 | 0.007 | 0.37 | 0.47 |
|  | VLDL | | 0.046 | 0.023 | 0.05 | 0.021 | 0.007 | **0.002** | 0.023 | 0.007 | **0.0005** | 0.003 |
|  | remnant | | 0.036 | 0.023 | 0.12 | 0.017 | 0.007 | 0.02 | 0.018 | 0.007 | 0.01 | 0.02 |
|  | LDL | | 0.012 | 0.023 | 0.62 | 0.007 | 0.007 | 0.31 | 0.008 | 0.007 | 0.26 | 0.38 |
|  | HDL | | -0.047 | 0.021 | 0.03 | -0.013 | 0.007 | 0.06 | -0.016 | 0.007 | 0.01 | 0.03 |
|  | HDL2 | | -0.046 | 0.021 | 0.03 | -0.013 | 0.007 | 0.06 | -0.016 | 0.007 | 0.02 | 0.03 |
|  | HDL3 | | -0.035 | 0.022 | 0.11 | -0.008 | 0.007 | 0.26 | -0.011 | 0.007 | 0.12 | 0.21 |
|  | esterified | | -0.001 | 0.023 | 0.97 | 0.007 | 0.007 | 0.35 | 0.006 | 0.007 | 0.37 | 0.47 |
|  | free | | -0.005 | 0.023 | 0.84 | 0.006 | 0.007 | 0.40 | 0.005 | 0.007 | 0.45 | 0.54 |
| Triglycerides | serum total | | 0.043 | 0.023 | 0.06 | 0.023 | 0.007 | **0.001** | 0.025 | 0.007 | **0.0002** | 0.002 |
|  | VLDL | | 0.050 | 0.023 | 0.03 | 0.023 | 0.007 | **0.001** | 0.025 | 0.007 | **0.0001** | 0.002 |
|  | LDL | | -0.017 | 0.023 | 0.45 | 0.009 | 0.007 | 0.21 | 0.006 | 0.006 | 0.32 | 0.42 |
|  | HDL | | 0.023 | 0.023 | 0.33 | 0.015 | 0.007 | 0.04 | 0.015 | 0.007 | 0.02 | 0.05 |
| Total phosphoglycerides | | | -0.007 | 0.023 | 0.77 | 0.007 | 0.007 | 0.35 | 0.006 | 0.007 | 0.42 | 0.52 |
| Phosphatidylcholine | | | -0.006 | 0.023 | 0.79 | 0.006 | 0.007 | 0.39 | 0.005 | 0.007 | 0.46 | 0.54 |
| Sphingomyelins | | | -0.008 | 0.022 | 0.74 | 0.000 | 0.007 | 1.00 | -0.001 | 0.007 | 0.92 | 0.94 |
| Total cholines | | | -0.005 | 0.023 | 0.81 | 0.005 | 0.007 | 0.49 | 0.004 | 0.007 | 0.56 | 0.64 |
| Apolipoprotein A-I | | | -0.034 | 0.022 | 0.12 | -0.005 | 0.007 | 0.53 | -0.007 | 0.007 | 0.27 | 0.39 |
| Apolipoprotein B | | | 0.035 | 0.023 | 0.13 | 0.017 | 0.007 | 0.01 | 0.019 | 0.007 | 0.01 | 0.02 |
| Apo-B / apoA-I | | | 0.049 | 0.023 | 0.03 | 0.021 | 0.007 | **0.002** | 0.023 | 0.006 | **0.0004** | 0.003 |
| Fatty acids | total | | 0.019 | 0.024 | 0.43 | 0.015 | 0.007 | 0.04 | 0.015 | 0.007 | 0.03 | 0.06 |
|  | unsaturated | | -0.053 | 0.023 | 0.02 | -0.014 | 0.007 | 0.05 | -0.017 | 0.007 | 0.01 | 0.03 |
|  | DHA (docosahexaenoic acid) | | -0.045 | 0.024 | 0.06 | -0.002 | 0.007 | 0.80 | -0.005 | 0.007 | 0.43 | 0.52 |
|  | LA (linoleic acid) | | 0.013 | 0.024 | 0.59 | 0.009 | 0.007 | 0.23 | 0.009 | 0.007 | 0.19 | 0.30 |
|  | omega-3 | | -0.031 | 0.024 | 0.19 | 0.007 | 0.007 | 0.36 | 0.003 | 0.007 | 0.62 | 0.70 |
|  | omega-6 | | 0.006 | 0.023 | 0.80 | 0.010 | 0.007 | 0.19 | 0.009 | 0.007 | 0.18 | 0.30 |
|  | PUFA (polyunsaturated) | | -0.003 | 0.023 | 0.91 | 0.009 | 0.007 | 0.19 | 0.008 | 0.007 | 0.23 | 0.35 |
|  | MUFA (monounsaturated) | | 0.026 | 0.023 | 0.27 | 0.019 | 0.007 | 0.01 | 0.020 | 0.007 | 0.004 | 0.01 |
|  | SAFA (saturated) | | 0.024 | 0.024 | 0.31 | 0.015 | 0.007 | 0.03 | 0.016 | 0.007 | 0.02 | 0.04 |
|  | DHA% | | -0.067 | 0.023 | 0.00 | -0.011 | 0.007 | 0.14 | -0.015 | 0.007 | 0.02 | 0.05 |
|  | LA% | | -0.014 | 0.024 | 0.55 | -0.014 | 0.007 | 0.06 | -0.014 | 0.007 | 0.05 | 0.09 |
|  | omega-3% | | -0.059 | 0.023 | 0.01 | -0.006 | 0.007 | 0.41 | -0.011 | 0.007 | 0.12 | 0.22 |
|  | omega-6% | | -0.028 | 0.023 | 0.23 | -0.017 | 0.007 | 0.02 | -0.017 | 0.007 | 0.01 | 0.02 |
|  | PUFA% | | -0.043 | 0.023 | 0.06 | -0.016 | 0.007 | 0.02 | -0.019 | 0.007 | 0.01 | 0.02 |
|  | MUFA% | | 0.033 | 0.023 | 0.15 | 0.020 | 0.007 | 0.004 | 0.021 | 0.007 | **0.002** | 0.01 |
|  | SAFA% | | 0.028 | 0.024 | 0.23 | 0.005 | 0.007 | 0.53 | 0.007 | 0.007 | 0.34 | 0.44 |
| Glucose | | | 0.049 | 0.022 | 0.03 | -0.002 | 0.007 | 0.76 | 0.002 | 0.007 | 0.71 | 0.77 |
| Lactate | | | 0.020 | 0.023 | 0.38 | -0.002 | 0.007 | 0.74 | 0.000 | 0.007 | 0.95 | 0.96 |
| Pyruvate | | | 0.043 | 0.024 | 0.07 | 0.000 | 0.007 | 0.98 | 0.004 | 0.007 | 0.59 | 0.67 |
| Citrate | | | 0.001 | 0.023 | 0.98 | -0.015 | 0.007 | 0.03 | -0.013 | 0.007 | 0.04 | 0.08 |
| Glycerol | | | 0.008 | 0.024 | 0.72 | -0.003 | 0.008 | 0.70 | -0.002 | 0.007 | 0.80 | 0.84 |
| Alanine | | | 0.003 | 0.023 | 0.89 | -0.001 | 0.007 | 0.92 | 0.000 | 0.007 | 0.95 | 0.96 |
| Glutamine | | | 0.003 | 0.023 | 0.88 | 0.009 | 0.007 | 0.23 | 0.008 | 0.007 | 0.24 | 0.35 |
| Glycine | | | -0.023 | 0.022 | 0.30 | 0.015 | 0.007 | 0.03 | 0.012 | 0.007 | 0.08 | 0.16 |
| Histidine | | | -0.031 | 0.023 | 0.17 | 0.000 | 0.007 | 0.98 | -0.003 | 0.007 | 0.69 | 0.76 |
| Isoleucine | | | 0.030 | 0.023 | 0.19 | 0.018 | 0.007 | 0.01 | 0.019 | 0.007 | 0.004 | 0.01 |
| Leucine | | | 0.031 | 0.023 | 0.17 | 0.019 | 0.007 | 0.01 | 0.020 | 0.007 | 0.003 | 0.01 |
| Valine | | | 0.010 | 0.023 | 0.67 | 0.010 | 0.007 | 0.17 | 0.010 | 0.007 | 0.15 | 0.25 |
| Phenylalanine | | | -0.014 | 0.023 | 0.55 | 0.001 | 0.007 | 0.84 | 0.000 | 0.007 | 0.99 | 0.99 |
| Tyrosine | | | -0.010 | 0.023 | 0.65 | 0.010 | 0.007 | 0.16 | 0.008 | 0.007 | 0.24 | 0.35 |
| Acetate | | | 0.018 | 0.024 | 0.45 | 0.003 | 0.007 | 0.73 | 0.004 | 0.007 | 0.58 | 0.66 |
| Acetoacetate | | | -0.010 | 0.024 | 0.69 | 0.004 | 0.007 | 0.59 | 0.003 | 0.007 | 0.69 | 0.76 |
| 3-hydroxybutyrate | | | -0.018 | 0.023 | 0.43 | -0.009 | 0.007 | 0.19 | -0.010 | 0.007 | 0.14 | 0.24 |
| Creatinine | | | 0.020 | 0.023 | 0.38 | -0.019 | 0.007 | 0.01 | -0.016 | 0.006 | 0.01 | 0.03 |
| Albumin | | | -0.031 | 0.023 | 0.16 | -0.003 | 0.007 | 0.69 | -0.006 | 0.007 | 0.42 | 0.51 |
| GlycA (glycoprotein acetylation) | | | 0.004 | 0.022 | 0.86 | 0.012 | 0.007 | 0.09 | 0.012 | 0.007 | 0.09 | 0.17 |

| **INADEQUATE ROOT FILLINGS** | | | | | | | | | | | | |
| --- | --- | --- | --- | --- | --- | --- | --- | --- | --- | --- | --- | --- |
| **Metabolite** | | | **Parogene** | | | **Health 2000** | | | **Meta-analysis** | | | |
|  | | | Beta | SE | p | Beta | SE | p | Beta | SE | p | FDR |
| VLDL | XXL | particles | 0.024 | 0.039 | 0.55 | 0.031 | 0.011 | 0.01 | 0.030 | 0.011 | 0.01 | 0.02 |
|  |  | lipid | 0.025 | 0.039 | 0.53 | 0.031 | 0.011 | 0.01 | 0.030 | 0.011 | 0.01 | 0.02 |
|  |  | phospholipid | 0.015 | 0.039 | 0.70 | 0.032 | 0.011 | 0.005 | 0.031 | 0.011 | 0.005 | 0.02 |
|  |  | cholesterol | 0.019 | 0.039 | 0.62 | 0.030 | 0.011 | 0.01 | 0.029 | 0.011 | 0.01 | 0.02 |
|  |  | cholesteryl ester | 0.028 | 0.039 | 0.48 | 0.029 | 0.011 | 0.01 | 0.029 | 0.011 | 0.01 | 0.02 |
|  |  | free cholesterol | 0.009 | 0.039 | 0.81 | 0.031 | 0.011 | 0.01 | 0.029 | 0.011 | 0.01 | 0.02 |
|  |  | triglycerides | 0.026 | 0.039 | 0.50 | 0.032 | 0.011 | 0.01 | 0.031 | 0.011 | 0.004 | 0.02 |
|  | XL | particles | 0.019 | 0.039 | 0.62 | 0.031 | 0.011 | 0.01 | 0.030 | 0.011 | 0.01 | 0.02 |
|  |  | lipid | 0.024 | 0.039 | 0.54 | 0.030 | 0.011 | 0.01 | 0.030 | 0.011 | 0.01 | 0.02 |
|  |  | phospholipid | 0.015 | 0.039 | 0.69 | 0.033 | 0.011 | 0.004 | 0.031 | 0.011 | 0.004 | 0.02 |
|  |  | cholesterol | 0.019 | 0.039 | 0.63 | 0.030 | 0.011 | 0.01 | 0.030 | 0.011 | 0.01 | 0.02 |
|  |  | cholesteryl ester | 0.025 | 0.039 | 0.53 | 0.030 | 0.011 | 0.01 | 0.029 | 0.011 | 0.01 | 0.02 |
|  |  | free cholesterol | 0.011 | 0.039 | 0.79 | 0.032 | 0.011 | 0.00 | 0.030 | 0.011 | 0.01 | 0.02 |
|  |  | triglycerides | 0.024 | 0.039 | 0.54 | 0.030 | 0.011 | 0.01 | 0.030 | 0.011 | 0.01 | 0.02 |
|  | L | particles | 0.028 | 0.039 | 0.48 | 0.031 | 0.011 | 0.01 | 0.030 | 0.011 | 0.005 | 0.02 |
|  |  | lipid | 0.043 | 0.039 | 0.27 | 0.031 | 0.011 | 0.01 | 0.032 | 0.011 | 0.003 | 0.02 |
|  |  | phospholipid | 0.030 | 0.039 | 0.45 | 0.031 | 0.011 | 0.01 | 0.031 | 0.011 | 0.004 | 0.02 |
|  |  | cholesterol | 0.037 | 0.039 | 0.35 | 0.032 | 0.011 | 0.005 | 0.032 | 0.011 | 0.003 | 0.02 |
|  |  | cholesteryl ester | 0.050 | 0.039 | 0.20 | 0.032 | 0.011 | 0.005 | 0.033 | 0.011 | **0.002** | 0.01 |
|  |  | free cholesterol | 0.020 | 0.039 | 0.61 | 0.030 | 0.011 | 0.01 | 0.030 | 0.011 | 0.01 | 0.02 |
|  |  | triglycerides | 0.038 | 0.039 | 0.33 | 0.030 | 0.011 | 0.01 | 0.031 | 0.011 | 0.004 | 0.02 |
|  | M | particles | 0.032 | 0.039 | 0.41 | 0.030 | 0.011 | 0.01 | 0.030 | 0.011 | 0.005 | 0.02 |
|  |  | lipid | 0.050 | 0.039 | 0.20 | 0.031 | 0.011 | 0.01 | 0.032 | 0.011 | **0.002** | 0.01 |
|  |  | phospholipid | 0.039 | 0.039 | 0.32 | 0.032 | 0.011 | 0.005 | 0.032 | 0.011 | **0.0027** | 0.02 |
|  |  | cholesterol | 0.044 | 0.039 | 0.26 | 0.034 | 0.011 | **0.003** | 0.034 | 0.011 | **0.001** | 0.01 |
|  |  | cholesteryl ester | 0.052 | 0.039 | 0.19 | 0.035 | 0.011 | **0.002** | 0.036 | 0.011 | **0.001** | 0.01 |
|  |  | free cholesterol | 0.030 | 0.039 | 0.44 | 0.031 | 0.011 | 0.01 | 0.031 | 0.011 | 0.004 | 0.02 |
|  |  | triglycerides | 0.043 | 0.038 | 0.27 | 0.030 | 0.011 | 0.01 | 0.031 | 0.011 | 0.004 | 0.02 |
|  | S | particles | 0.038 | 0.039 | 0.33 | 0.037 | 0.011 | **0.001** | 0.037 | 0.011 | **0.001** | 0.01 |
|  |  | lipid | 0.050 | 0.039 | 0.20 | 0.038 | 0.011 | **0.001** | 0.039 | 0.011 | **0.0003** | 0.01 |
|  |  | phospholipid | 0.045 | 0.039 | 0.26 | 0.041 | 0.011 | **0.0003** | 0.041 | 0.011 | **0.0001** | 0.01 |
|  |  | cholesterol | 0.052 | 0.039 | 0.19 | 0.038 | 0.011 | **0.001** | 0.039 | 0.011 | **0.0003** | 0.01 |
|  |  | cholesteryl ester | 0.057 | 0.039 | 0.15 | 0.035 | 0.011 | **0.002** | 0.037 | 0.011 | **0.001** | 0.01 |
|  |  | free cholesterol | 0.035 | 0.039 | 0.37 | 0.039 | 0.011 | **0.001** | 0.039 | 0.011 | **0.0004** | 0.01 |
|  |  | triglycerides | 0.038 | 0.039 | 0.33 | 0.033 | 0.011 | 0.004 | 0.033 | 0.011 | **0.002** | 0.01 |
|  | XS | particles | 0.021 | 0.039 | 0.59 | 0.033 | 0.011 | 0.003 | 0.033 | 0.011 | **0.0027** | 0.02 |
|  |  | lipid | 0.021 | 0.039 | 0.59 | 0.034 | 0.011 | 0.003 | 0.033 | 0.011 | **0.0026** | 0.02 |
|  |  | phospholipid | 0.013 | 0.039 | 0.73 | 0.030 | 0.011 | 0.01 | 0.029 | 0.011 | 0.01 | 0.02 |
|  |  | cholesterol | 0.031 | 0.039 | 0.43 | 0.028 | 0.011 | 0.01 | 0.029 | 0.011 | 0.01 | 0.02 |
|  |  | cholesteryl ester | 0.038 | 0.038 | 0.33 | 0.028 | 0.011 | 0.02 | 0.029 | 0.011 | 0.01 | 0.02 |
|  |  | free cholesterol | 0.015 | 0.039 | 0.70 | 0.028 | 0.011 | 0.01 | 0.027 | 0.011 | 0.01 | 0.03 |
|  |  | triglycerides | 0.008 | 0.039 | 0.84 | 0.037 | 0.011 | **0.001** | 0.035 | 0.011 | **0.001** | 0.01 |
| IDL | | particles | 0.017 | 0.039 | 0.66 | 0.022 | 0.012 | 0.06 | 0.022 | 0.011 | 0.05 | 0.09 |
|  |  | lipid | 0.013 | 0.039 | 0.74 | 0.023 | 0.012 | 0.05 | 0.022 | 0.011 | 0.04 | 0.08 |
|  |  | phospholipid | 0.019 | 0.039 | 0.62 | 0.019 | 0.012 | 0.09 | 0.019 | 0.011 | 0.08 | 0.11 |
|  |  | cholesterol | 0.024 | 0.039 | 0.54 | 0.021 | 0.012 | 0.07 | 0.021 | 0.011 | 0.06 | 0.09 |
|  |  | cholesteryl ester | 0.031 | 0.039 | 0.43 | 0.022 | 0.012 | 0.05 | 0.023 | 0.011 | 0.04 | 0.07 |
|  |  | free cholesterol | 0.006 | 0.039 | 0.88 | 0.015 | 0.012 | 0.21 | 0.014 | 0.011 | 0.21 | 0.25 |
|  |  | triglycerides | -0.002 | 0.038 | 0.96 | 0.030 | 0.011 | 0.01 | 0.028 | 0.010 | 0.01 | 0.02 |
| LDL | L | particles | 0.018 | 0.039 | 0.64 | 0.021 | 0.012 | 0.08 | 0.020 | 0.011 | 0.07 | 0.10 |
|  |  | lipid | 0.015 | 0.039 | 0.69 | 0.022 | 0.012 | 0.05 | 0.022 | 0.011 | 0.05 | 0.09 |
|  |  | phospholipid | 0.020 | 0.039 | 0.61 | 0.021 | 0.012 | 0.06 | 0.021 | 0.011 | 0.05 | 0.09 |
|  |  | cholesterol | 0.020 | 0.039 | 0.62 | 0.021 | 0.012 | 0.08 | 0.021 | 0.011 | 0.06 | 0.10 |
|  |  | cholesteryl ester | 0.023 | 0.039 | 0.56 | 0.022 | 0.012 | 0.06 | 0.022 | 0.011 | 0.05 | 0.08 |
|  |  | free cholesterol | 0.013 | 0.039 | 0.75 | 0.014 | 0.012 | 0.23 | 0.014 | 0.011 | 0.21 | 0.25 |
|  |  | triglycerides | -0.020 | 0.039 | 0.61 | 0.027 | 0.011 | 0.01 | 0.024 | 0.010 | 0.02 | 0.05 |
|  | M | particles | 0.010 | 0.039 | 0.79 | 0.020 | 0.012 | 0.09 | 0.019 | 0.011 | 0.09 | 0.12 |
|  |  | lipid | 0.006 | 0.039 | 0.87 | 0.021 | 0.012 | 0.07 | 0.020 | 0.011 | 0.07 | 0.11 |
|  |  | phospholipid | 0.007 | 0.039 | 0.86 | 0.024 | 0.012 | 0.04 | 0.022 | 0.011 | 0.04 | 0.08 |
|  |  | cholesterol | 0.015 | 0.039 | 0.70 | 0.019 | 0.012 | 0.10 | 0.018 | 0.011 | 0.10 | 0.13 |
|  |  | cholesteryl ester | 0.020 | 0.039 | 0.61 | 0.019 | 0.012 | 0.10 | 0.019 | 0.011 | 0.09 | 0.12 |
|  |  | free cholesterol | 0.000 | 0.039 | 1.00 | 0.016 | 0.012 | 0.16 | 0.015 | 0.011 | 0.17 | 0.22 |
|  |  | triglycerides | -0.045 | 0.039 | 0.25 | 0.025 | 0.011 | 0.02 | 0.020 | 0.010 | 0.05 | 0.09 |
|  | S | particles | 0.009 | 0.039 | 0.81 | 0.020 | 0.012 | 0.09 | 0.019 | 0.011 | 0.09 | 0.12 |
|  |  | lipid | 0.007 | 0.039 | 0.87 | 0.020 | 0.012 | 0.08 | 0.019 | 0.011 | 0.08 | 0.12 |
|  |  | phospholipid | 0.000 | 0.039 | 0.99 | 0.022 | 0.012 | 0.06 | 0.020 | 0.011 | 0.07 | 0.10 |
|  |  | cholesterol | 0.014 | 0.039 | 0.72 | 0.017 | 0.012 | 0.14 | 0.017 | 0.011 | 0.13 | 0.17 |
|  |  | cholesteryl ester | 0.017 | 0.039 | 0.66 | 0.017 | 0.012 | 0.14 | 0.017 | 0.011 | 0.13 | 0.16 |
|  |  | free cholesterol | 0.003 | 0.039 | 0.94 | 0.016 | 0.012 | 0.16 | 0.015 | 0.011 | 0.17 | 0.21 |
|  |  | triglycerides | -0.020 | 0.039 | 0.62 | 0.032 | 0.011 | 0.004 | 0.029 | 0.011 | 0.01 | 0.02 |
| HDL | XL | particles | -0.077 | 0.037 | 0.04 | -0.016 | 0.011 | 0.13 | -0.021 | 0.010 | 0.04 | 0.08 |
|  |  | lipid | -0.072 | 0.037 | 0.05 | -0.019 | 0.011 | 0.08 | -0.023 | 0.010 | 0.02 | 0.05 |
|  |  | phospholipid | -0.079 | 0.037 | 0.03 | -0.019 | 0.011 | 0.07 | -0.024 | 0.010 | 0.02 | 0.04 |
|  |  | cholesterol | -0.066 | 0.037 | 0.08 | -0.018 | 0.011 | 0.11 | -0.021 | 0.011 | 0.04 | 0.08 |
|  |  | cholesteryl ester | -0.063 | 0.037 | 0.09 | -0.016 | 0.011 | 0.15 | -0.020 | 0.011 | 0.06 | 0.10 |
|  |  | free cholesterol | -0.075 | 0.037 | 0.04 | -0.019 | 0.011 | 0.07 | -0.024 | 0.010 | 0.02 | 0.049 |
|  |  | triglycerides | -0.027 | 0.040 | 0.49 | 0.010 | 0.012 | 0.39 | 0.007 | 0.011 | 0.53 | 0.56 |
|  | L | particles | -0.103 | 0.036 | 0.00 | -0.008 | 0.011 | 0.44 | -0.016 | 0.010 | 0.12 | 0.16 |
|  |  | lipid | -0.096 | 0.036 | 0.01 | -0.012 | 0.011 | 0.27 | -0.019 | 0.010 | 0.07 | 0.10 |
|  |  | phospholipid | -0.092 | 0.036 | 0.01 | -0.007 | 0.011 | 0.50 | -0.014 | 0.010 | 0.17 | 0.21 |
|  |  | cholesterol | -0.107 | 0.036 | 0.004 | -0.014 | 0.011 | 0.18 | -0.022 | 0.010 | 0.03 | 0.07 |
|  |  | cholesteryl ester | -0.106 | 0.036 | 0.004 | -0.014 | 0.011 | 0.20 | -0.021 | 0.010 | 0.04 | 0.07 |
|  |  | free cholesterol | -0.112 | 0.036 | **0.002** | -0.014 | 0.011 | 0.18 | -0.022 | 0.010 | 0.03 | 0.06 |
|  |  | triglycerides | -0.062 | 0.037 | 0.09 | 0.001 | 0.011 | 0.91 | -0.004 | 0.011 | 0.70 | 0.72 |
|  | M | particles | -0.044 | 0.037 | 0.24 | 0.022 | 0.012 | 0.05 | 0.016 | 0.011 | 0.14 | 0.18 |
|  |  | lipid | -0.046 | 0.037 | 0.21 | 0.021 | 0.012 | 0.07 | 0.015 | 0.011 | 0.18 | 0.22 |
|  |  | phospholipid | -0.046 | 0.037 | 0.22 | 0.021 | 0.012 | 0.06 | 0.015 | 0.011 | 0.16 | 0.20 |
|  |  | cholesterol | -0.052 | 0.037 | 0.16 | 0.016 | 0.012 | 0.17 | 0.010 | 0.011 | 0.37 | 0.41 |
|  |  | cholesteryl ester | -0.052 | 0.038 | 0.17 | 0.016 | 0.012 | 0.17 | 0.010 | 0.011 | 0.37 | 0.41 |
|  |  | free cholesterol | -0.051 | 0.037 | 0.17 | 0.016 | 0.012 | 0.18 | 0.010 | 0.011 | 0.38 | 0.42 |
|  |  | triglycerides | 0.051 | 0.039 | 0.20 | 0.044 | 0.012 | **0.0001** | 0.045 | 0.011 | **0.0001** | 0.01 |
|  | S | particles | 0.013 | 0.039 | 0.74 | 0.025 | 0.012 | 0.03 | 0.024 | 0.011 | 0.03 | 0.07 |
|  |  | lipid | 0.012 | 0.039 | 0.77 | 0.021 | 0.012 | 0.07 | 0.021 | 0.011 | 0.07 | 0.10 |
|  |  | phospholipid | 0.009 | 0.039 | 0.83 | 0.023 | 0.012 | 0.05 | 0.021 | 0.011 | 0.06 | 0.09 |
|  |  | cholesterol | -0.002 | 0.039 | 0.96 | 0.014 | 0.012 | 0.24 | 0.013 | 0.011 | 0.26 | 0.31 |
|  |  | cholesteryl ester | 0.003 | 0.039 | 0.94 | 0.011 | 0.012 | 0.33 | 0.011 | 0.011 | 0.34 | 0.39 |
|  |  | free cholesterol | -0.022 | 0.039 | 0.57 | 0.020 | 0.012 | 0.09 | 0.016 | 0.011 | 0.15 | 0.19 |
|  |  | triglycerides | 0.057 | 0.039 | 0.14 | 0.031 | 0.011 | 0.01 | 0.033 | 0.011 | **0.002** | 0.01 |
| VLDL diameter | | | 0.068 | 0.038 | 0.08 | 0.022 | 0.011 | 0.05 | 0.025 | 0.011 | 0.02 | 0.04 |
| LDL diameter | | | 0.006 | 0.038 | 0.87 | -0.009 | 0.011 | 0.44 | -0.007 | 0.011 | 0.49 | 0.53 |
| HDL diameter | | | -0.090 | 0.037 | 0.01 | -0.021 | 0.011 | 0.05 | -0.026 | 0.010 | 0.01 | 0.02 |
| Cholesterol | serum total | | -0.020 | 0.038 | 0.60 | 0.024 | 0.012 | 0.04 | 0.020 | 0.011 | 0.07 | 0.10 |
|  | VLDL | | 0.050 | 0.039 | 0.21 | 0.039 | 0.011 | **0.001** | 0.040 | 0.011 | **0.0003** | 0.01 |
|  | remnant | | 0.039 | 0.039 | 0.32 | 0.035 | 0.011 | **0.002** | 0.035 | 0.011 | **0.001** | 0.01 |
|  | LDL | | 0.015 | 0.039 | 0.71 | 0.020 | 0.012 | 0.08 | 0.020 | 0.011 | 0.07 | 0.10 |
|  | HDL | | -0.092 | 0.036 | 0.01 | -0.004 | 0.011 | 0.75 | -0.011 | 0.011 | 0.29 | 0.33 |
|  | HDL2 | | -0.087 | 0.036 | 0.02 | -0.004 | 0.011 | 0.73 | -0.011 | 0.011 | 0.30 | 0.34 |
|  | HDL3 | | -0.090 | 0.037 | 0.01 | 0.002 | 0.011 | 0.89 | -0.006 | 0.011 | 0.55 | 0.57 |
|  | esterified | | -0.018 | 0.039 | 0.64 | 0.024 | 0.012 | 0.04 | 0.021 | 0.011 | 0.06 | 0.10 |
|  | free | | -0.021 | 0.039 | 0.58 | 0.023 | 0.012 | 0.05 | 0.019 | 0.011 | 0.08 | 0.12 |
| Triglycerides | serum total | | 0.038 | 0.039 | 0.33 | 0.036 | 0.011 | **0.002** | 0.036 | 0.011 | **0.001** | 0.01 |
|  | VLDL | | 0.046 | 0.039 | 0.23 | 0.032 | 0.011 | 0.005 | 0.033 | 0.011 | **0.002** | 0.01 |
|  | LDL | | -0.032 | 0.039 | 0.40 | 0.030 | 0.011 | 0.01 | 0.025 | 0.011 | 0.02 | 0.04 |
|  | HDL | | 0.004 | 0.039 | 0.92 | 0.034 | 0.011 | 0.003 | 0.032 | 0.011 | 0.004 | 0.02 |
| Total phosphoglycerides | | | -0.042 | 0.038 | 0.27 | 0.030 | 0.012 | 0.01 | 0.024 | 0.011 | 0.03 | 0.06 |
| Phosphatidylcholine | | | -0.036 | 0.038 | 0.35 | 0.030 | 0.012 | 0.01 | 0.024 | 0.011 | 0.03 | 0.06 |
| Sphingomyelins | | | -0.026 | 0.038 | 0.50 | 0.014 | 0.011 | 0.23 | 0.010 | 0.011 | 0.34 | 0.39 |
| Total cholines | | | -0.034 | 0.038 | 0.38 | 0.027 | 0.012 | 0.02 | 0.022 | 0.011 | 0.05 | 0.09 |
| Apolipoprotein A-I | | | -0.090 | 0.036 | 0.01 | 0.012 | 0.012 | 0.31 | 0.002 | 0.011 | 0.83 | 0.84 |
| Apolipoprotein B | | | 0.031 | 0.039 | 0.43 | 0.032 | 0.011 | 0.005 | 0.032 | 0.011 | 0.003 | 0.02 |
| Apo-B / apoA-I | | | 0.068 | 0.038 | 0.08 | 0.027 | 0.011 | 0.01 | 0.030 | 0.011 | 0.005 | 0.02 |
| Fatty acids | total | | 0.002 | 0.040 | 0.95 | 0.037 | 0.012 | 0.00 | 0.034 | 0.011 | **0.002** | 0.01 |
|  | unsaturated | | -0.040 | 0.039 | 0.31 | -0.022 | 0.011 | 0.06 | -0.023 | 0.011 | 0.04 | 0.07 |
|  | DHA (docosahexaenoic acid) | | -0.061 | 0.040 | 0.12 | 0.013 | 0.012 | 0.26 | 0.007 | 0.011 | 0.51 | 0.55 |
|  | LA (linoleic acid) | | 0.004 | 0.040 | 0.92 | 0.025 | 0.012 | 0.04 | 0.023 | 0.011 | 0.04 | 0.08 |
|  | omega-3 | | -0.044 | 0.040 | 0.27 | 0.026 | 0.012 | 0.02 | 0.021 | 0.011 | 0.06 | 0.10 |
|  | omega-6 | | -0.006 | 0.039 | 0.88 | 0.027 | 0.012 | 0.02 | 0.025 | 0.011 | 0.03 | 0.06 |
|  | PUFA (polyunsaturated) | | -0.017 | 0.039 | 0.66 | 0.029 | 0.012 | 0.02 | 0.025 | 0.011 | 0.03 | 0.06 |
|  | MUFA (monounsaturated) | | 0.018 | 0.039 | 0.65 | 0.041 | 0.012 | **0.0004** | 0.039 | 0.011 | **0.0004** | 0.01 |
|  | SAFA (saturated) | | 0.005 | 0.040 | 0.90 | 0.037 | 0.012 | **0.002** | 0.034 | 0.011 | **0.002** | 0.01 |
|  | DHA% | | -0.094 | 0.039 | 0.02 | -0.006 | 0.012 | 0.63 | -0.013 | 0.011 | 0.25 | 0.30 |
|  | LA% | | -0.005 | 0.040 | 0.89 | -0.028 | 0.012 | 0.02 | -0.026 | 0.011 | 0.02 | 0.046 |
|  | omega-3% | | -0.080 | 0.039 | 0.04 | 0.003 | 0.012 | 0.79 | -0.004 | 0.011 | 0.74 | 0.76 |
|  | omega-6% | | -0.018 | 0.039 | 0.64 | -0.032 | 0.011 | 0.004 | -0.031 | 0.011 | 0.004 | 0.02 |
|  | PUFA% | | -0.040 | 0.039 | 0.30 | -0.028 | 0.011 | 0.02 | -0.029 | 0.011 | 0.01 | 0.02 |
|  | MUFA% | | 0.040 | 0.038 | 0.30 | 0.032 | 0.011 | 0.005 | 0.033 | 0.011 | **0.0027** | 0.02 |
|  | SAFA% | | 0.012 | 0.040 | 0.77 | 0.008 | 0.012 | 0.48 | 0.009 | 0.011 | 0.44 | 0.48 |
| Glucose | | | 0.044 | 0.037 | 0.24 | 0.011 | 0.011 | 0.31 | 0.014 | 0.011 | 0.19 | 0.23 |
| Lactate | | | -0.004 | 0.039 | 0.92 | 0.010 | 0.011 | 0.39 | 0.009 | 0.011 | 0.43 | 0.46 |
| Pyruvate | | | 0.057 | 0.040 | 0.15 | 0.011 | 0.011 | 0.33 | 0.015 | 0.011 | 0.18 | 0.22 |
| Citrate | | | -0.036 | 0.039 | 0.36 | -0.027 | 0.011 | 0.02 | -0.027 | 0.011 | 0.01 | 0.02 |
| Glycerol | | | -0.020 | 0.040 | 0.61 | 0.008 | 0.013 | 0.52 | 0.006 | 0.012 | 0.65 | 0.67 |
| Alanine | | | -0.001 | 0.039 | 0.97 | 0.008 | 0.012 | 0.51 | 0.007 | 0.011 | 0.54 | 0.56 |
| Glutamine | | | 0.021 | 0.039 | 0.59 | 0.016 | 0.012 | 0.18 | 0.016 | 0.011 | 0.15 | 0.19 |
| Glycine | | | -0.020 | 0.038 | 0.60 | 0.028 | 0.011 | 0.01 | 0.024 | 0.011 | 0.03 | 0.06 |
| Histidine | | | -0.077 | 0.038 | 0.04 | 0.008 | 0.012 | 0.49 | 0.001 | 0.011 | 0.95 | 0.96 |
| Isoleucine | | | 0.032 | 0.038 | 0.40 | 0.032 | 0.011 | 0.005 | 0.032 | 0.011 | 0.003 | 0.02 |
| Leucine | | | 0.038 | 0.038 | 0.32 | 0.037 | 0.011 | **0.001** | 0.037 | 0.011 | **0.0005** | 0.01 |
| Valine | | | 0.009 | 0.038 | 0.81 | 0.024 | 0.011 | 0.03 | 0.023 | 0.011 | 0.03 | 0.07 |
| Phenylalanine | | | -0.015 | 0.039 | 0.70 | 0.014 | 0.011 | 0.21 | 0.012 | 0.011 | 0.28 | 0.32 |
| Tyrosine | | | 0.000 | 0.038 | 0.99 | 0.024 | 0.012 | 0.04 | 0.022 | 0.011 | 0.04 | 0.08 |
| Acetate | | | 0.054 | 0.040 | 0.18 | 0.009 | 0.012 | 0.45 | 0.013 | 0.011 | 0.27 | 0.31 |
| Acetoacetate | | | -0.006 | 0.041 | 0.87 | 0.010 | 0.012 | 0.38 | 0.009 | 0.011 | 0.42 | 0.46 |
| 3-hydroxybutyrate | | | -0.035 | 0.039 | 0.37 | -0.016 | 0.012 | 0.16 | -0.018 | 0.011 | 0.11 | 0.15 |
| Creatinine | | | -0.001 | 0.039 | 0.98 | -0.025 | 0.011 | 0.02 | -0.024 | 0.010 | 0.02 | 0.049 |
| Albumin | | | -0.061 | 0.038 | 0.11 | 0.005 | 0.012 | 0.64 | 0.000 | 0.011 | 0.98 | 0.98 |
| GlycA (glycoprotein acetylation) | | | 0.023 | 0.037 | 0.54 | 0.031 | 0.012 | 0.01 | 0.031 | 0.011 | 0.01 | 0.02 |

**APPENDIX TABLE 2.** **Associations of 157 metabolic measures with periodontal parameters.** Linear regression adjusted for age, gender, smoking, number of teeth, and diabetes (in Health-2000 and Parogene), and level of education (in Health-2000 only). Significant p-values (< 0.0028) are indicated by red colour. Significant FDRs (< 0.05) for meta-analysis are indicated by purple colour.

| **BOP** | | | | | | | | | | | | |
| --- | --- | --- | --- | --- | --- | --- | --- | --- | --- | --- | --- | --- |
| **Metabolite** | | | **Parogene** | | | **Health 2000** | | | **Meta-analysis** | | | |
|  | | | Beta | SE | p | Beta | SE | p | Beta | SE | p | FDR |
| VLDL | XXL | particles | 0.002 | 0.002 | 0.33 | 0.016 | 0.006 | 0.01 |  |  |  |  |
|  |  | lipid | 0.002 | 0.002 | 0.33 | 0.016 | 0.006 | 0.01 |  |  |  |  |
|  |  | phospholipid | 0.002 | 0.002 | 0.42 | 0.017 | 0.006 | 0.01 |  |  |  |  |
|  |  | cholesterol | 0.002 | 0.002 | 0.39 | 0.017 | 0.006 | 0.01 |  |  |  |  |
|  |  | cholesteryl ester | 0.002 | 0.002 | 0.33 | 0.021 | 0.006 | **0.001** |  |  |  |  |
|  |  | free cholesterol | 0.002 | 0.002 | 0.49 | 0.025 | 0.007 | **0.0001** |  |  |  |  |
|  |  | triglycerides | 0.002 | 0.002 | 0.30 | 0.025 | 0.007 | **0.0002** |  |  |  |  |
|  | XL | particles | 0.002 | 0.002 | 0.39 | 0.023 | 0.007 | **0.0004** |  |  |  |  |
|  |  | lipid | 0.002 | 0.002 | 0.40 | 0.022 | 0.007 | **0.001** |  |  |  |  |
|  |  | phospholipid | 0.002 | 0.002 | 0.46 | 0.021 | 0.007 | **0.002** |  |  |  |  |
|  |  | cholesterol | 0.002 | 0.002 | 0.43 | -0.015 | 0.006 | 0.01 |  |  |  |  |
|  |  | cholesteryl ester | 0.002 | 0.002 | 0.37 | -0.012 | 0.006 | 0.04 |  |  |  |  |
|  |  | free cholesterol | 0.001 | 0.002 | 0.53 | 0.010 | 0.007 | 0.12 |  |  |  |  |
|  |  | triglycerides | 0.002 | 0.002 | 0.38 | 0.019 | 0.007 | 0.01 |  |  |  |  |
|  | L | particles | 0.002 | 0.002 | 0.35 | 0.007 | 0.006 | 0.24 |  |  |  |  |
|  |  | lipid | 0.002 | 0.002 | 0.34 | 0.000 | 0.006 | 0.98 |  |  |  |  |
|  |  | phospholipid | 0.002 | 0.002 | 0.39 | -0.020 | 0.006 | **0.001** |  |  |  |  |
|  |  | cholesterol | 0.002 | 0.002 | 0.34 | 0.005 | 0.007 | 0.47 |  |  |  |  |
|  |  | cholesteryl ester | 0.003 | 0.002 | 0.26 | 0.027 | 0.007 | **4.1E-05** |  |  |  |  |
|  |  | free cholesterol | 0.002 | 0.002 | 0.46 | 0.025 | 0.006 | **4.7E-05** |  |  |  |  |
|  |  | triglycerides | 0.002 | 0.002 | 0.34 | 0.016 | 0.006 | 0.01 |  |  |  |  |
|  | M | particles | 0.002 | 0.002 | 0.29 | 0.016 | 0.006 | 0.01 |  |  |  |  |
|  |  | lipid | 0.003 | 0.002 | 0.24 | 0.017 | 0.006 | 0.01 |  |  |  |  |
|  |  | phospholipid | 0.002 | 0.002 | 0.30 | 0.018 | 0.006 | 0.005 |  |  |  |  |
|  |  | cholesterol | 0.002 | 0.002 | 0.28 | 0.022 | 0.006 | **0.0005** |  |  |  |  |
|  |  | cholesteryl ester | 0.003 | 0.002 | 0.23 | 0.024 | 0.007 | **0.0002** |  |  |  |  |
|  |  | free cholesterol | 0.002 | 0.002 | 0.38 | 0.023 | 0.007 | **0.001** |  |  |  |  |
|  |  | triglycerides | 0.003 | 0.002 | 0.26 | 0.021 | 0.007 | **0.001** |  |  |  |  |
|  | S | particles | 0.002 | 0.002 | 0.32 | 0.021 | 0.007 | **0.002** |  |  |  |  |
|  |  | lipid | 0.002 | 0.002 | 0.30 | 0.019 | 0.007 | 0.003 |  |  |  |  |
|  |  | phospholipid | 0.003 | 0.002 | 0.26 | -0.016 | 0.006 | 0.01 |  |  |  |  |
|  |  | cholesterol | 0.001 | 0.002 | 0.55 | -0.014 | 0.006 | 0.02 |  |  |  |  |
|  |  | cholesteryl ester | 0.001 | 0.002 | 0.67 | 0.008 | 0.007 | 0.22 |  |  |  |  |
|  |  | free cholesterol | 0.002 | 0.002 | 0.45 | 0.015 | 0.007 | 0.02 |  |  |  |  |
|  |  | triglycerides | 0.003 | 0.002 | 0.26 | 0.019 | 0.006 | 0.004 |  |  |  |  |
|  | XS | particles | 0.000 | 0.002 | 0.94 | 0.015 | 0.006 | 0.02 |  |  |  |  |
|  |  | lipid | 0.000 | 0.002 | 0.87 | 0.017 | 0.006 | 0.01 |  |  |  |  |
|  |  | phospholipid | -0.001 | 0.002 | 0.74 | 0.016 | 0.006 | 0.01 |  |  |  |  |
|  |  | cholesterol | -0.001 | 0.002 | 0.62 | 0.015 | 0.006 | 0.02 |  |  |  |  |
|  |  | cholesteryl ester | -0.001 | 0.002 | 0.70 | 0.014 | 0.006 | 0.03 |  |  |  |  |
|  |  | free cholesterol | -0.001 | 0.002 | 0.52 | 0.017 | 0.006 | 0.01 |  |  |  |  |
|  |  | triglycerides | 0.001 | 0.002 | 0.58 | 0.018 | 0.006 | 0.01 |  |  |  |  |
| IDL | | particles | -0.001 | 0.002 | 0.54 | 0.019 | 0.006 | **0.0025** |  |  |  |  |
|  |  | lipid | -0.001 | 0.002 | 0.54 | 0.023 | 0.006 | **0.0003** |  |  |  |  |
|  |  | phospholipid | -0.001 | 0.002 | 0.51 | 0.022 | 0.006 | **0.001** |  |  |  |  |
|  |  | cholesterol | -0.001 | 0.002 | 0.54 | 0.021 | 0.006 | **0.001** |  |  |  |  |
|  |  | cholesteryl ester | -0.001 | 0.002 | 0.62 | 0.019 | 0.007 | 0.003 |  |  |  |  |
|  |  | free cholesterol | -0.002 | 0.002 | 0.36 | 0.020 | 0.007 | **0.002** |  |  |  |  |
|  |  | triglycerides | -0.001 | 0.002 | 0.79 | 0.004 | 0.007 | 0.54 |  |  |  |  |
| LDL | L | particles | -0.001 | 0.002 | 0.53 | -0.006 | 0.006 | 0.34 |  |  |  |  |
|  |  | lipid | -0.001 | 0.002 | 0.56 | 0.026 | 0.007 | **0.0001** |  |  |  |  |
|  |  | phospholipid | -0.001 | 0.002 | 0.62 | 0.020 | 0.006 | **0.002** |  |  |  |  |
|  |  | cholesterol | -0.001 | 0.002 | 0.52 | 0.019 | 0.007 | 0.005 |  |  |  |  |
|  |  | cholesteryl ester | -0.001 | 0.002 | 0.55 | 0.019 | 0.007 | 0.004 |  |  |  |  |
|  |  | free cholesterol | -0.002 | 0.002 | 0.42 | 0.019 | 0.007 | 0.005 |  |  |  |  |
|  |  | triglycerides | -0.001 | 0.002 | 0.64 | 0.027 | 0.007 | **2.8E-05** |  |  |  |  |
|  | M | particles | -0.002 | 0.002 | 0.42 | 0.026 | 0.006 | **4.9E-05** |  |  |  |  |
|  |  | lipid | -0.002 | 0.002 | 0.41 | 0.018 | 0.006 | 0.01 |  |  |  |  |
|  |  | phospholipid | -0.002 | 0.002 | 0.43 | 0.017 | 0.006 | 0.01 |  |  |  |  |
|  |  | cholesterol | -0.002 | 0.002 | 0.45 | 0.017 | 0.006 | 0.01 |  |  |  |  |
|  |  | cholesteryl ester | -0.002 | 0.002 | 0.51 | 0.021 | 0.006 | **0.001** |  |  |  |  |
|  |  | free cholesterol | -0.003 | 0.002 | 0.28 | 0.026 | 0.006 | **0.0001** |  |  |  |  |
|  |  | triglycerides | -0.002 | 0.002 | 0.33 | 0.023 | 0.007 | **0.0003** |  |  |  |  |
|  | S | particles | -0.002 | 0.002 | 0.47 | 0.021 | 0.007 | **0.002** |  |  |  |  |
|  |  | lipid | -0.002 | 0.002 | 0.45 | 0.019 | 0.007 | 0.004 |  |  |  |  |
|  |  | phospholipid | -0.002 | 0.002 | 0.43 | 0.021 | 0.007 | **0.002** |  |  |  |  |
|  |  | cholesterol | -0.002 | 0.002 | 0.47 | 0.020 | 0.007 | **0.0027** |  |  |  |  |
|  |  | cholesteryl ester | -0.001 | 0.002 | 0.53 | 0.019 | 0.007 | 0.01 |  |  |  |  |
|  |  | free cholesterol | -0.002 | 0.002 | 0.31 | -0.007 | 0.006 | 0.25 |  |  |  |  |
|  |  | triglycerides | -0.001 | 0.002 | 0.75 | -0.008 | 0.006 | 0.19 |  |  |  |  |
| HDL | XL | particles | -0.002 | 0.002 | 0.35 | 0.002 | 0.007 | 0.77 |  |  |  |  |
|  |  | lipid | -0.002 | 0.002 | 0.38 | -0.016 | 0.006 | 0.01 |  |  |  |  |
|  |  | phospholipid | -0.002 | 0.002 | 0.35 | -0.016 | 0.006 | 0.01 |  |  |  |  |
|  |  | cholesterol | -0.002 | 0.002 | 0.47 | 0.005 | 0.007 | 0.43 |  |  |  |  |
|  |  | cholesteryl ester | -0.001 | 0.002 | 0.51 | 0.015 | 0.007 | 0.02 |  |  |  |  |
|  |  | free cholesterol | -0.002 | 0.002 | 0.36 | 0.018 | 0.006 | 0.005 |  |  |  |  |
|  |  | triglycerides | -0.003 | 0.002 | 0.21 | 0.015 | 0.006 | 0.02 |  |  |  |  |
|  | L | particles | -0.003 | 0.002 | 0.16 | 0.018 | 0.006 | 0.004 |  |  |  |  |
|  |  | lipid | -0.003 | 0.002 | 0.20 | 0.023 | 0.006 | **0.0004** |  |  |  |  |
|  |  | phospholipid | -0.003 | 0.002 | 0.20 | 0.026 | 0.006 | **0.0001** |  |  |  |  |
|  |  | cholesterol | -0.003 | 0.002 | 0.17 | 0.025 | 0.007 | **0.0001** |  |  |  |  |
|  |  | cholesteryl ester | -0.003 | 0.002 | 0.17 | 0.022 | 0.007 | **0.001** |  |  |  |  |
|  |  | free cholesterol | -0.003 | 0.002 | 0.14 | 0.022 | 0.007 | **0.001** |  |  |  |  |
|  |  | triglycerides | -0.003 | 0.002 | 0.17 | 0.020 | 0.007 | **0.002** |  |  |  |  |
|  | M | particles | -0.002 | 0.002 | 0.36 | 0.019 | 0.007 | 0.004 |  |  |  |  |
|  |  | lipid | -0.002 | 0.002 | 0.32 | -0.016 | 0.006 | 0.01 |  |  |  |  |
|  |  | phospholipid | -0.002 | 0.002 | 0.44 | -0.016 | 0.006 | 0.01 |  |  |  |  |
|  |  | cholesterol | -0.003 | 0.002 | 0.17 | 0.005 | 0.007 | 0.46 |  |  |  |  |
|  |  | cholesteryl ester | -0.003 | 0.002 | 0.16 | 0.016 | 0.007 | 0.02 |  |  |  |  |
|  |  | free cholesterol | -0.003 | 0.002 | 0.18 | 0.015 | 0.006 | 0.02 |  |  |  |  |
|  |  | triglycerides | 0.004 | 0.002 | 0.08 | 0.017 | 0.006 | 0.01 |  |  |  |  |
|  | S | particles | 0.000 | 0.002 | 0.96 | 0.017 | 0.006 | 0.01 |  |  |  |  |
|  |  | lipid | 0.000 | 0.002 | 0.95 | 0.018 | 0.006 | 0.005 |  |  |  |  |
|  |  | phospholipid | 0.001 | 0.002 | 0.78 | 0.022 | 0.006 | **0.001** |  |  |  |  |
|  |  | cholesterol | -0.002 | 0.002 | 0.34 | 0.020 | 0.007 | **0.00276** |  |  |  |  |
|  |  | cholesteryl ester | -0.002 | 0.002 | 0.38 | 0.018 | 0.007 | 0.01 |  |  |  |  |
|  |  | free cholesterol | -0.002 | 0.002 | 0.36 | 0.019 | 0.007 | 0.005 |  |  |  |  |
|  |  | triglycerides | 0.004 | 0.002 | 0.12 | 0.019 | 0.007 | 0.004 |  |  |  |  |
| VLDL diameter | | | 0.004 | 0.002 | 0.09 | 0.016 | 0.007 | 0.01 |  |  |  |  |
| LDL diameter | | | -0.001 | 0.002 | 0.69 | -0.016 | 0.006 | 0.01 |  |  |  |  |
| HDL diameter | | | -0.003 | 0.002 | 0.20 | -0.017 | 0.006 | 0.01 |  |  |  |  |
| Cholesterol | serum total | | -0.002 | 0.002 | 0.35 | 0.007 | 0.007 | 0.25 |  |  |  |  |
|  | VLDL | | 0.002 | 0.002 | 0.49 | 0.010 | 0.007 | 0.14 |  |  |  |  |
|  | remnant | | 0.000 | 0.002 | 0.87 | 0.018 | 0.006 | 0.01 |  |  |  |  |
|  | LDL | | -0.001 | 0.002 | 0.52 | 0.017 | 0.006 | 0.01 |  |  |  |  |
|  | HDL | | -0.004 | 0.002 | 0.09 | 0.018 | 0.006 | 0.005 |  |  |  |  |
|  | HDL2 | | -0.004 | 0.002 | 0.10 | 0.020 | 0.006 | **0.002** |  |  |  |  |
|  | HDL3 | | -0.004 | 0.002 | 0.09 | 0.023 | 0.006 | **0.0003** |  |  |  |  |
|  | esterified | | -0.002 | 0.002 | 0.47 | 0.025 | 0.007 | **0.0001** |  |  |  |  |
|  | free | | -0.002 | 0.002 | 0.42 | 0.021 | 0.007 | **0.001** |  |  |  |  |
| Triglycerides | serum total | | 0.002 | 0.002 | 0.32 | 0.022 | 0.007 | **0.001** |  |  |  |  |
|  | VLDL | | 0.003 | 0.002 | 0.27 | 0.023 | 0.007 | **0.0005** |  |  |  |  |
|  | LDL | | -0.001 | 0.002 | 0.70 | 0.020 | 0.007 | **0.002** |  |  |  |  |
|  | HDL | | 0.001 | 0.002 | 0.58 | -0.016 | 0.006 | 0.01 |  |  |  |  |
| Total phosphoglycerides | | | -0.002 | 0.002 | 0.43 | -0.011 | 0.006 | 0.08 |  |  |  |  |
| Phosphatidylcholine | | | -0.002 | 0.002 | 0.38 | 0.009 | 0.007 | 0.15 |  |  |  |  |
| Sphingomyelins | | | -0.003 | 0.002 | 0.12 | 0.011 | 0.007 | 0.09 |  |  |  |  |
| Total cholines | | | -0.002 | 0.002 | 0.30 | 0.016 | 0.007 | 0.02 |  |  |  |  |
| Apolipoprotein A-I | | | -0.004 | 0.002 | 0.07 | 0.017 | 0.007 | 0.01 |  |  |  |  |
| Apolipoprotein B | | | 0.001 | 0.002 | 0.83 | 0.015 | 0.007 | 0.02 |  |  |  |  |
| Apo-B / apoA-I | | | 0.002 | 0.002 | 0.29 | 0.018 | 0.007 | 0.01 |  |  |  |  |
| Fatty acids | total | | -0.001 | 0.002 | 0.78 | 0.022 | 0.007 | **0.001** |  |  |  |  |
|  | unsaturated | | -0.002 | 0.002 | 0.32 | 0.015 | 0.007 | 0.02 |  |  |  |  |
|  | DHA (docosahexaenoic acid) | | -0.003 | 0.002 | 0.26 | 0.014 | 0.007 | 0.04 |  |  |  |  |
|  | LA (linoleic acid) | | -0.001 | 0.002 | 0.67 | 0.016 | 0.007 | 0.02 |  |  |  |  |
|  | omega-3 | | -0.002 | 0.002 | 0.41 | 0.012 | 0.007 | 0.08 |  |  |  |  |
|  | omega-6 | | -0.002 | 0.002 | 0.48 | 0.016 | 0.007 | 0.02 |  |  |  |  |
|  | PUFA (polyunsaturated) | | -0.002 | 0.002 | 0.40 | 0.024 | 0.007 | **0.0003** |  |  |  |  |
|  | MUFA (monounsaturated) | | 0.001 | 0.002 | 0.75 | 0.024 | 0.007 | **0.0003** |  |  |  |  |
|  | SAFA (saturated) | | -0.001 | 0.002 | 0.82 | 0.000 | 0.007 | 0.94 |  |  |  |  |
|  | DHA% | | -0.004 | 0.002 | 0.10 | -0.001 | 0.007 | 0.83 |  |  |  |  |
|  | LA% | | -0.001 | 0.002 | 0.77 | -0.023 | 0.006 | **0.0005** |  |  |  |  |
|  | omega-3% | | -0.002 | 0.002 | 0.28 | -0.023 | 0.007 | **0.0005** |  |  |  |  |
|  | omega-6% | | -0.002 | 0.002 | 0.46 | -0.021 | 0.006 | **0.001** |  |  |  |  |
|  | PUFA% | | -0.002 | 0.002 | 0.28 | 0.015 | 0.007 | 0.02 |  |  |  |  |
|  | MUFA% | | 0.003 | 0.002 | 0.17 | 0.016 | 0.007 | 0.02 |  |  |  |  |
|  | SAFA% | | 0.000 | 0.002 | 0.90 | -0.017 | 0.007 | 0.01 |  |  |  |  |
| Glucose | | | 0.001 | 0.002 | 0.58 | 0.000 | 0.007 | 0.99 |  |  |  |  |
| Lactate | | | -0.003 | 0.002 | 0.14 | 0.000 | 0.007 | 0.96 |  |  |  |  |
| Pyruvate | | | 0.002 | 0.002 | 0.52 | 0.014 | 0.007 | 0.04 |  |  |  |  |
| Citrate | | | 0.001 | 0.002 | 0.73 | 0.002 | 0.007 | 0.78 |  |  |  |  |
| Glycerol | | | 0.001 | 0.002 | 0.73 | 0.015 | 0.006 | 0.02 |  |  |  |  |
| Alanine | | | 0.001 | 0.002 | 0.64 | 0.017 | 0.006 | 0.01 |  |  |  |  |
| Glutamine | | | -0.002 | 0.002 | 0.48 | 0.010 | 0.006 | 0.13 |  |  |  |  |
| Glycine | | | -0.003 | 0.002 | 0.25 | 0.017 | 0.007 | 0.01 |  |  |  |  |
| Histidine | | | 0.000 | 0.002 | 0.88 | 0.005 | 0.007 | 0.43 |  |  |  |  |
| Isoleucine | | | 0.002 | 0.002 | 0.37 | 0.014 | 0.006 | 0.03 |  |  |  |  |
| Leucine | | | -0.001 | 0.002 | 0.80 | 0.021 | 0.006 | **0.001** |  |  |  |  |
| Valine | | | -0.002 | 0.002 | 0.35 | 0.024 | 0.006 | **0.0003** |  |  |  |  |
| Phenylalanine | | | -0.005 | 0.002 | 0.03 | -0.007 | 0.006 | 0.24 |  |  |  |  |
| Tyrosine | | | 0.000 | 0.002 | 0.99 | 0.011 | 0.007 | 0.12 |  |  |  |  |
| Acetate | | | -0.001 | 0.002 | 0.68 | 0.011 | 0.007 | 0.09 |  |  |  |  |
| Acetoacetate | | | 0.000 | 0.002 | 0.91 | 0.008 | 0.007 | 0.22 |  |  |  |  |
| 3-hydroxybutyrate | | | -0.002 | 0.002 | 0.44 | 0.010 | 0.007 | 0.16 |  |  |  |  |
| Creatinine | | | 0.000 | 0.002 | 0.93 | 0.004 | 0.007 | 0.51 |  |  |  |  |
| Albumin | | | -0.006 | 0.002 | 0.01 | -0.017 | 0.006 | 0.01 |  |  |  |  |
| GlycA (glycoprotein acetylation) | | | -0.003 | 0.002 | 0.15 | 0.020 | 0.007 | **0.002** |  |  |  |  |

| **ABL** | | | | | | | | | | | | |
| --- | --- | --- | --- | --- | --- | --- | --- | --- | --- | --- | --- | --- |
| **Metabolite** | | | **Parogene** | | | **Health 2000** | | | **Meta-analysis** | | | |
|  | | | Beta | SE | p | Beta | SE | p | Beta | SE | p | FDR |
| VLDL | XXL | particles | 0.056 | 0.065 | 0.39 | -0.017 | 0.021 | 0.43 | -0.010 | 0.020 | 0.63 | 0.73 |
|  |  | lipid | 0.055 | 0.065 | 0.40 | -0.019 | 0.021 | 0.38 | -0.012 | 0.020 | 0.57 | 0.70 |
|  |  | phospholipid | 0.055 | 0.065 | 0.40 | -0.017 | 0.022 | 0.42 | -0.010 | 0.020 | 0.62 | 0.73 |
|  |  | cholesterol | 0.075 | 0.065 | 0.25 | -0.022 | 0.022 | 0.30 | -0.013 | 0.020 | 0.53 | 0.67 |
|  |  | cholesteryl ester | 0.087 | 0.065 | 0.18 | -0.025 | 0.022 | 0.25 | -0.014 | 0.021 | 0.49 | 0.67 |
|  |  | free cholesterol | 0.060 | 0.065 | 0.36 | -0.023 | 0.021 | 0.28 | -0.015 | 0.020 | 0.46 | 0.66 |
|  |  | triglycerides | 0.051 | 0.065 | 0.43 | -0.013 | 0.022 | 0.54 | -0.007 | 0.020 | 0.74 | 0.82 |
|  | XL | particles | 0.070 | 0.064 | 0.28 | -0.026 | 0.021 | 0.23 | -0.016 | 0.020 | 0.42 | 0.63 |
|  |  | lipid | 0.064 | 0.064 | 0.32 | -0.024 | 0.021 | 0.26 | -0.015 | 0.020 | 0.46 | 0.66 |
|  |  | phospholipid | 0.069 | 0.065 | 0.29 | -0.023 | 0.021 | 0.28 | -0.014 | 0.020 | 0.50 | 0.67 |
|  |  | cholesterol | 0.077 | 0.065 | 0.23 | -0.023 | 0.021 | 0.28 | -0.013 | 0.020 | 0.52 | 0.67 |
|  |  | cholesteryl ester | 0.085 | 0.065 | 0.19 | -0.028 | 0.021 | 0.20 | -0.016 | 0.020 | 0.42 | 0.63 |
|  |  | free cholesterol | 0.069 | 0.065 | 0.29 | -0.023 | 0.021 | 0.29 | -0.014 | 0.020 | 0.51 | 0.67 |
|  |  | triglycerides | 0.063 | 0.064 | 0.33 | -0.025 | 0.021 | 0.24 | -0.016 | 0.020 | 0.42 | 0.63 |
|  | L | particles | 0.082 | 0.064 | 0.20 | -0.036 | 0.021 | 0.10 | -0.024 | 0.020 | 0.24 | 0.58 |
|  |  | lipid | 0.073 | 0.064 | 0.26 | -0.031 | 0.021 | 0.14 | -0.021 | 0.020 | 0.30 | 0.58 |
|  |  | phospholipid | 0.081 | 0.064 | 0.21 | -0.035 | 0.021 | 0.10 | -0.023 | 0.020 | 0.25 | 0.58 |
|  |  | cholesterol | 0.090 | 0.064 | 0.16 | -0.032 | 0.021 | 0.13 | -0.020 | 0.020 | 0.32 | 0.58 |
|  |  | cholesteryl ester | 0.102 | 0.065 | 0.11 | -0.033 | 0.021 | 0.12 | -0.020 | 0.020 | 0.33 | 0.59 |
|  |  | free cholesterol | 0.080 | 0.064 | 0.22 | -0.035 | 0.021 | 0.10 | -0.023 | 0.020 | 0.25 | 0.58 |
|  |  | triglycerides | 0.072 | 0.064 | 0.26 | -0.031 | 0.021 | 0.14 | -0.021 | 0.020 | 0.30 | 0.58 |
|  | M | particles | 0.105 | 0.064 | 0.10 | -0.036 | 0.021 | 0.09 | -0.022 | 0.020 | 0.28 | 0.58 |
|  |  | lipid | 0.100 | 0.064 | 0.12 | -0.033 | 0.021 | 0.12 | -0.020 | 0.020 | 0.31 | 0.58 |
|  |  | phospholipid | 0.106 | 0.064 | 0.10 | -0.038 | 0.021 | 0.08 | -0.024 | 0.020 | 0.24 | 0.58 |
|  |  | cholesterol | 0.109 | 0.065 | 0.09 | -0.035 | 0.021 | 0.10 | -0.021 | 0.020 | 0.29 | 0.58 |
|  |  | cholesteryl ester | 0.112 | 0.065 | 0.09 | -0.033 | 0.021 | 0.12 | -0.019 | 0.020 | 0.34 | 0.60 |
|  |  | free cholesterol | 0.105 | 0.064 | 0.10 | -0.037 | 0.021 | 0.09 | -0.023 | 0.020 | 0.27 | 0.58 |
|  |  | triglycerides | 0.098 | 0.064 | 0.13 | -0.034 | 0.021 | 0.11 | -0.021 | 0.020 | 0.30 | 0.58 |
|  | S | particles | 0.118 | 0.065 | 0.07 | -0.043 | 0.021 | 0.04 | -0.028 | 0.020 | 0.16 | 0.58 |
|  |  | lipid | 0.111 | 0.066 | 0.09 | -0.042 | 0.021 | 0.05 | -0.027 | 0.020 | 0.17 | 0.58 |
|  |  | phospholipid | 0.113 | 0.066 | 0.09 | -0.047 | 0.021 | 0.03 | -0.032 | 0.020 | 0.12 | 0.47 |
|  |  | cholesterol | 0.105 | 0.066 | 0.11 | -0.040 | 0.021 | 0.06 | -0.027 | 0.020 | 0.18 | 0.58 |
|  |  | cholesteryl ester | 0.090 | 0.067 | 0.18 | -0.036 | 0.021 | 0.09 | -0.024 | 0.020 | 0.23 | 0.58 |
|  |  | free cholesterol | 0.118 | 0.066 | 0.07 | -0.047 | 0.021 | 0.03 | -0.031 | 0.020 | 0.12 | 0.47 |
|  |  | triglycerides | 0.113 | 0.065 | 0.08 | -0.038 | 0.021 | 0.07 | -0.024 | 0.020 | 0.24 | 0.58 |
|  | XS | particles | 0.081 | 0.066 | 0.22 | -0.027 | 0.021 | 0.21 | -0.017 | 0.020 | 0.41 | 0.63 |
|  |  | lipid | 0.069 | 0.066 | 0.30 | -0.030 | 0.021 | 0.16 | -0.020 | 0.020 | 0.31 | 0.58 |
|  |  | phospholipid | 0.053 | 0.066 | 0.42 | -0.022 | 0.021 | 0.30 | -0.015 | 0.020 | 0.46 | 0.66 |
|  |  | cholesterol | 0.019 | 0.066 | 0.77 | -0.027 | 0.021 | 0.21 | -0.022 | 0.020 | 0.27 | 0.58 |
|  |  | cholesteryl ester | 0.003 | 0.066 | 0.96 | -0.021 | 0.021 | 0.33 | -0.018 | 0.020 | 0.36 | 0.60 |
|  |  | free cholesterol | 0.050 | 0.066 | 0.45 | -0.035 | 0.021 | 0.10 | -0.027 | 0.020 | 0.18 | 0.58 |
|  |  | triglycerides | 0.137 | 0.065 | 0.04 | -0.032 | 0.021 | 0.13 | -0.016 | 0.020 | 0.42 | 0.63 |
| IDL | | particles | 0.021 | 0.065 | 0.74 | -0.014 | 0.021 | 0.50 | -0.011 | 0.020 | 0.59 | 0.72 |
|  |  | lipid | 0.017 | 0.065 | 0.80 | -0.022 | 0.022 | 0.31 | -0.018 | 0.020 | 0.38 | 0.62 |
|  |  | phospholipid | -0.010 | 0.065 | 0.88 | -0.013 | 0.021 | 0.55 | -0.013 | 0.020 | 0.54 | 0.67 |
|  |  | cholesterol | -0.013 | 0.065 | 0.84 | -0.022 | 0.022 | 0.32 | -0.021 | 0.021 | 0.31 | 0.58 |
|  |  | cholesteryl ester | -0.014 | 0.065 | 0.83 | -0.022 | 0.022 | 0.30 | -0.021 | 0.021 | 0.29 | 0.58 |
|  |  | free cholesterol | -0.009 | 0.065 | 0.89 | -0.011 | 0.022 | 0.61 | -0.011 | 0.020 | 0.60 | 0.72 |
|  |  | triglycerides | 0.060 | 0.065 | 0.36 | -0.016 | 0.020 | 0.42 | -0.010 | 0.019 | 0.62 | 0.73 |
| LDL | L | particles | 0.011 | 0.065 | 0.86 | -0.017 | 0.021 | 0.43 | -0.014 | 0.020 | 0.48 | 0.66 |
|  |  | lipid | 0.005 | 0.065 | 0.94 | -0.026 | 0.022 | 0.23 | -0.023 | 0.020 | 0.27 | 0.58 |
|  |  | phospholipid | -0.009 | 0.065 | 0.89 | -0.022 | 0.022 | 0.32 | -0.020 | 0.020 | 0.32 | 0.58 |
|  |  | cholesterol | 0.009 | 0.065 | 0.89 | -0.025 | 0.022 | 0.26 | -0.021 | 0.021 | 0.30 | 0.58 |
|  |  | cholesteryl ester | 0.015 | 0.065 | 0.82 | -0.025 | 0.022 | 0.25 | -0.021 | 0.020 | 0.31 | 0.58 |
|  |  | free cholesterol | -0.009 | 0.065 | 0.89 | -0.014 | 0.022 | 0.53 | -0.013 | 0.020 | 0.52 | 0.67 |
|  |  | triglycerides | 0.044 | 0.065 | 0.49 | -0.012 | 0.020 | 0.56 | -0.007 | 0.019 | 0.73 | 0.81 |
|  | M | particles | 0.040 | 0.065 | 0.54 | -0.021 | 0.021 | 0.33 | -0.015 | 0.020 | 0.46 | 0.66 |
|  |  | lipid | 0.039 | 0.065 | 0.55 | -0.029 | 0.022 | 0.18 | -0.022 | 0.020 | 0.28 | 0.58 |
|  |  | phospholipid | 0.025 | 0.065 | 0.70 | -0.032 | 0.022 | 0.14 | -0.026 | 0.020 | 0.20 | 0.58 |
|  |  | cholesterol | 0.035 | 0.065 | 0.59 | -0.025 | 0.022 | 0.24 | -0.020 | 0.020 | 0.34 | 0.60 |
|  |  | cholesteryl ester | 0.039 | 0.065 | 0.55 | -0.023 | 0.022 | 0.29 | -0.017 | 0.020 | 0.41 | 0.63 |
|  |  | free cholesterol | 0.015 | 0.065 | 0.81 | -0.027 | 0.022 | 0.20 | -0.023 | 0.020 | 0.26 | 0.58 |
|  |  | triglycerides | 0.081 | 0.065 | 0.21 | -0.008 | 0.020 | 0.68 | 0.000 | 0.019 | 0.98 | 0.99 |
|  | S | particles | 0.033 | 0.065 | 0.61 | -0.026 | 0.021 | 0.22 | -0.020 | 0.020 | 0.32 | 0.58 |
|  |  | lipid | 0.030 | 0.065 | 0.65 | -0.031 | 0.022 | 0.15 | -0.025 | 0.020 | 0.22 | 0.58 |
|  |  | phospholipid | -0.001 | 0.064 | 0.99 | -0.041 | 0.022 | 0.06 | -0.037 | 0.021 | 0.07 | 0.37 |
|  |  | cholesterol | 0.033 | 0.065 | 0.61 | -0.025 | 0.022 | 0.25 | -0.019 | 0.020 | 0.35 | 0.60 |
|  |  | cholesteryl ester | 0.040 | 0.065 | 0.54 | -0.021 | 0.022 | 0.34 | -0.015 | 0.020 | 0.47 | 0.66 |
|  |  | free cholesterol | 0.008 | 0.064 | 0.91 | -0.036 | 0.022 | 0.10 | -0.032 | 0.020 | 0.12 | 0.47 |
|  |  | triglycerides | 0.076 | 0.065 | 0.24 | -0.030 | 0.021 | 0.15 | -0.020 | 0.020 | 0.31 | 0.58 |
| HDL | XL | particles | -0.076 | 0.064 | 0.24 | 0.021 | 0.020 | 0.29 | 0.013 | 0.019 | 0.52 | 0.67 |
|  |  | lipid | -0.073 | 0.064 | 0.26 | 0.025 | 0.020 | 0.21 | 0.017 | 0.019 | 0.39 | 0.62 |
|  |  | phospholipid | -0.082 | 0.063 | 0.20 | 0.029 | 0.020 | 0.14 | 0.019 | 0.019 | 0.31 | 0.58 |
|  |  | cholesterol | -0.066 | 0.065 | 0.31 | 0.017 | 0.021 | 0.41 | 0.009 | 0.020 | 0.63 | 0.73 |
|  |  | cholesteryl ester | -0.056 | 0.066 | 0.40 | 0.014 | 0.021 | 0.49 | 0.008 | 0.020 | 0.69 | 0.78 |
|  |  | free cholesterol | -0.095 | 0.064 | 0.14 | 0.020 | 0.020 | 0.33 | 0.009 | 0.019 | 0.63 | 0.73 |
|  |  | triglycerides | -0.012 | 0.067 | 0.86 | 0.004 | 0.022 | 0.87 | 0.002 | 0.021 | 0.91 | 0.94 |
|  | L | particles | -0.114 | 0.063 | 0.07 | 0.001 | 0.020 | 0.94 | -0.010 | 0.019 | 0.62 | 0.73 |
|  |  | lipid | -0.113 | 0.062 | 0.07 | 0.005 | 0.020 | 0.82 | -0.007 | 0.019 | 0.73 | 0.81 |
|  |  | phospholipid | -0.114 | 0.062 | 0.07 | -0.001 | 0.020 | 0.95 | -0.012 | 0.019 | 0.53 | 0.67 |
|  |  | cholesterol | -0.116 | 0.063 | 0.06 | 0.008 | 0.020 | 0.69 | -0.004 | 0.019 | 0.85 | 0.90 |
|  |  | cholesteryl ester | -0.116 | 0.063 | 0.07 | 0.007 | 0.020 | 0.72 | -0.004 | 0.019 | 0.82 | 0.89 |
|  |  | free cholesterol | -0.118 | 0.063 | 0.06 | 0.008 | 0.020 | 0.70 | -0.004 | 0.019 | 0.84 | 0.89 |
|  |  | triglycerides | -0.045 | 0.062 | 0.47 | 0.002 | 0.021 | 0.93 | -0.003 | 0.020 | 0.89 | 0.92 |
|  | M | particles | -0.089 | 0.066 | 0.18 | -0.045 | 0.022 | 0.04 | -0.049 | 0.021 | 0.02 | 0.16 |
|  |  | lipid | -0.089 | 0.066 | 0.18 | -0.046 | 0.022 | 0.04 | -0.050 | 0.021 | 0.02 | 0.16 |
|  |  | phospholipid | -0.080 | 0.065 | 0.22 | -0.043 | 0.022 | 0.05 | -0.047 | 0.021 | 0.02 | 0.17 |
|  |  | cholesterol | -0.114 | 0.066 | 0.08 | -0.045 | 0.022 | 0.04 | -0.051 | 0.021 | 0.01 | 0.15 |
|  |  | cholesteryl ester | -0.116 | 0.066 | 0.08 | -0.044 | 0.022 | 0.04 | -0.051 | 0.021 | 0.01 | 0.15 |
|  |  | free cholesterol | -0.106 | 0.065 | 0.10 | -0.043 | 0.022 | 0.05 | -0.050 | 0.021 | 0.02 | 0.16 |
|  |  | triglycerides | 0.112 | 0.066 | 0.09 | -0.031 | 0.022 | 0.16 | -0.017 | 0.021 | 0.42 | 0.63 |
|  | S | particles | -0.073 | 0.067 | 0.27 | -0.060 | 0.022 | 0.01 | -0.061 | 0.021 | 0.004 | 0.12 |
|  |  | lipid | -0.089 | 0.067 | 0.19 | -0.055 | 0.022 | 0.01 | -0.058 | 0.021 | 0.01 | 0.12 |
|  |  | phospholipid | -0.118 | 0.067 | 0.08 | -0.055 | 0.022 | 0.01 | -0.062 | 0.021 | 0.003 | 0.12 |
|  |  | cholesterol | -0.032 | 0.066 | 0.63 | -0.047 | 0.022 | 0.04 | -0.045 | 0.021 | 0.03 | 0.23 |
|  |  | cholesteryl ester | -0.004 | 0.066 | 0.95 | -0.037 | 0.022 | 0.09 | -0.034 | 0.021 | 0.11 | 0.45 |
|  |  | free cholesterol | -0.116 | 0.067 | 0.08 | -0.067 | 0.022 | 0.00 | -0.072 | 0.021 | **0.001** | 0.08 |
|  |  | triglycerides | 0.078 | 0.065 | 0.23 | -0.025 | 0.021 | 0.25 | -0.015 | 0.020 | 0.46 | 0.66 |
| VLDL diameter | | | 0.051 | 0.064 | 0.43 | -0.006 | 0.021 | 0.77 | -0.001 | 0.020 | 0.97 | 0.98 |
| LDL diameter | | | -0.081 | 0.065 | 0.21 | 0.066 | 0.021 | **0.002** | 0.051 | 0.020 | 0.01 | 0.15 |
| HDL diameter | | | -0.121 | 0.063 | 0.06 | 0.025 | 0.020 | 0.21 | 0.012 | 0.019 | 0.54 | 0.67 |
| Cholesterol | serum total | | -0.001 | 0.064 | 0.98 | -0.039 | 0.022 | 0.08 | -0.035 | 0.021 | 0.09 | 0.40 |
|  | VLDL | | 0.096 | 0.066 | 0.14 | -0.039 | 0.021 | 0.07 | -0.026 | 0.020 | 0.20 | 0.58 |
|  | remnant | | 0.066 | 0.066 | 0.31 | -0.035 | 0.021 | 0.10 | -0.026 | 0.020 | 0.21 | 0.58 |
|  | LDL | | 0.021 | 0.065 | 0.75 | -0.030 | 0.022 | 0.16 | -0.025 | 0.021 | 0.22 | 0.58 |
|  | HDL | | -0.126 | 0.063 | 0.05 | -0.018 | 0.021 | 0.38 | -0.029 | 0.020 | 0.14 | 0.54 |
|  | HDL2 | | -0.133 | 0.063 | 0.04 | -0.015 | 0.021 | 0.47 | -0.026 | 0.020 | 0.18 | 0.58 |
|  | HDL3 | | -0.023 | 0.063 | 0.71 | -0.040 | 0.021 | 0.06 | -0.038 | 0.020 | 0.06 | 0.36 |
|  | esterified | | -0.022 | 0.066 | 0.74 | -0.040 | 0.022 | 0.07 | -0.038 | 0.021 | 0.06 | 0.36 |
|  | free | | 0.010 | 0.065 | 0.88 | -0.028 | 0.022 | 0.20 | -0.024 | 0.021 | 0.24 | 0.58 |
| Triglycerides | serum total | | 0.105 | 0.064 | 0.10 | -0.031 | 0.021 | 0.14 | -0.018 | 0.020 | 0.37 | 0.61 |
|  | VLDL | | 0.097 | 0.064 | 0.13 | -0.030 | 0.021 | 0.16 | -0.017 | 0.020 | 0.39 | 0.62 |
|  | LDL | | 0.089 | 0.065 | 0.17 | -0.014 | 0.020 | 0.51 | -0.004 | 0.019 | 0.82 | 0.89 |
|  | HDL | | 0.091 | 0.066 | 0.17 | -0.025 | 0.022 | 0.26 | -0.013 | 0.021 | 0.52 | 0.67 |
| Total phosphoglycerides | | | -0.015 | 0.066 | 0.82 | -0.040 | 0.022 | 0.07 | -0.037 | 0.021 | 0.08 | 0.37 |
| Phosphatidylcholine | | | 0.000 | 0.065 | 0.99 | -0.039 | 0.022 | 0.07 | -0.035 | 0.021 | 0.09 | 0.40 |
| Sphingomyelins | | | -0.034 | 0.064 | 0.60 | -0.016 | 0.021 | 0.46 | -0.018 | 0.020 | 0.38 | 0.62 |
| Total cholines | | | -0.009 | 0.065 | 0.89 | -0.031 | 0.022 | 0.16 | -0.028 | 0.021 | 0.17 | 0.58 |
| Apolipoprotein A-I | | | -0.103 | 0.064 | 0.11 | -0.038 | 0.022 | 0.08 | -0.045 | 0.021 | 0.03 | 0.22 |
| Apolipoprotein B | | | 0.079 | 0.065 | 0.23 | -0.034 | 0.021 | 0.11 | -0.023 | 0.020 | 0.26 | 0.58 |
| Apo-B / apoA-I | | | 0.128 | 0.065 | 0.05 | -0.013 | 0.020 | 0.53 | 0.000 | 0.019 | 0.99 | 0.99 |
| Fatty acids | total | | 0.029 | 0.067 | 0.66 | -0.043 | 0.022 | 0.05 | -0.036 | 0.021 | 0.08 | 0.40 |
|  | unsaturated | | -0.076 | 0.066 | 0.25 | -0.008 | 0.021 | 0.72 | -0.014 | 0.020 | 0.49 | 0.66 |
|  | DHA (docosahexaenoic acid) | | -0.064 | 0.069 | 0.35 | -0.050 | 0.022 | 0.02 | -0.051 | 0.021 | 0.01 | 0.15 |
|  | LA (linoleic acid) | | -0.006 | 0.067 | 0.93 | -0.026 | 0.022 | 0.24 | -0.024 | 0.021 | 0.26 | 0.58 |
|  | omega-3 | | -0.072 | 0.068 | 0.29 | -0.057 | 0.022 | 0.01 | -0.058 | 0.021 | 0.005 | 0.12 |
|  | omega-6 | | -0.018 | 0.067 | 0.79 | -0.034 | 0.022 | 0.12 | -0.033 | 0.021 | 0.12 | 0.47 |
|  | PUFA (polyunsaturated) | | -0.031 | 0.067 | 0.65 | -0.041 | 0.022 | 0.06 | -0.040 | 0.021 | 0.06 | 0.36 |
|  | MUFA (monounsaturated) | | 0.074 | 0.066 | 0.26 | -0.039 | 0.022 | 0.07 | -0.028 | 0.021 | 0.17 | 0.58 |
|  | SAFA (saturated) | | 0.028 | 0.067 | 0.67 | -0.042 | 0.022 | 0.05 | -0.035 | 0.021 | 0.09 | 0.40 |
|  | DHA% | | -0.093 | 0.066 | 0.16 | -0.048 | 0.021 | 0.03 | -0.052 | 0.020 | 0.01 | 0.15 |
|  | LA% | | -0.049 | 0.068 | 0.47 | 0.032 | 0.022 | 0.14 | 0.025 | 0.021 | 0.23 | 0.58 |
|  | omega-3% | | -0.111 | 0.066 | 0.10 | -0.064 | 0.022 | 0.003 | -0.068 | 0.020 | **0.001** | 0.08 |
|  | omega-6% | | -0.080 | 0.066 | 0.23 | 0.029 | 0.021 | 0.18 | 0.019 | 0.020 | 0.36 | 0.60 |
|  | PUFA% | | -0.104 | 0.066 | 0.11 | 0.009 | 0.021 | 0.66 | -0.002 | 0.020 | 0.94 | 0.96 |
|  | MUFA% | | 0.116 | 0.065 | 0.08 | -0.008 | 0.022 | 0.71 | 0.004 | 0.021 | 0.84 | 0.89 |
|  | SAFA% | | -0.013 | 0.066 | 0.85 | -0.005 | 0.022 | 0.82 | -0.006 | 0.021 | 0.78 | 0.86 |
| Glucose | | | -0.102 | 0.064 | 0.11 | -0.028 | 0.020 | 0.17 | -0.034 | 0.019 | 0.07 | 0.37 |
| Lactate | | | -0.041 | 0.066 | 0.53 | -0.036 | 0.021 | 0.09 | -0.036 | 0.020 | 0.07 | 0.37 |
| Pyruvate | | | -0.032 | 0.068 | 0.64 | -0.053 | 0.021 | 0.01 | -0.051 | 0.020 | 0.01 | 0.15 |
| Citrate | | | 0.020 | 0.068 | 0.77 | -0.012 | 0.020 | 0.55 | -0.010 | 0.019 | 0.62 | 0.73 |
| Glycerol | | | 0.080 | 0.068 | 0.24 | -0.075 | 0.023 | **0.001** | -0.060 | 0.022 | 0.01 | 0.12 |
| Alanine | | | -0.038 | 0.067 | 0.57 | -0.050 | 0.022 | 0.02 | -0.048 | 0.021 | 0.02 | 0.16 |
| Glutamine | | | 0.094 | 0.067 | 0.16 | -0.034 | 0.022 | 0.11 | -0.022 | 0.021 | 0.28 | 0.58 |
| Glycine | | | -0.035 | 0.065 | 0.60 | 0.000 | 0.021 | 0.99 | -0.003 | 0.020 | 0.88 | 0.92 |
| Histidine | | | -0.052 | 0.065 | 0.42 | -0.036 | 0.022 | 0.09 | -0.038 | 0.021 | 0.06 | 0.36 |
| Isoleucine | | | 0.104 | 0.064 | 0.11 | -0.036 | 0.021 | 0.09 | -0.022 | 0.020 | 0.26 | 0.58 |
| Leucine | | | 0.033 | 0.064 | 0.60 | -0.048 | 0.021 | 0.02 | -0.041 | 0.020 | 0.04 | 0.28 |
| Valine | | | -0.018 | 0.064 | 0.78 | -0.040 | 0.021 | 0.06 | -0.038 | 0.020 | 0.06 | 0.36 |
| Phenylalanine | | | 0.068 | 0.067 | 0.31 | -0.005 | 0.021 | 0.83 | 0.002 | 0.020 | 0.92 | 0.94 |
| Tyrosine | | | 0.007 | 0.067 | 0.92 | -0.017 | 0.021 | 0.43 | -0.015 | 0.020 | 0.48 | 0.66 |
| Acetate | | | 0.043 | 0.067 | 0.52 | 0.011 | 0.022 | 0.62 | 0.014 | 0.021 | 0.51 | 0.67 |
| Acetoacetate | | | 0.062 | 0.068 | 0.36 | -0.016 | 0.022 | 0.46 | -0.009 | 0.021 | 0.68 | 0.77 |
| 3-hydroxybutyrate | | | -0.003 | 0.068 | 0.97 | 0.005 | 0.022 | 0.81 | 0.005 | 0.021 | 0.83 | 0.89 |
| Creatinine | | | 0.053 | 0.068 | 0.44 | -0.058 | 0.019 | **0.002** | -0.050 | 0.018 | 0.01 | 0.12 |
| Albumin | | | -0.012 | 0.067 | 0.86 | -0.052 | 0.022 | 0.02 | -0.048 | 0.021 | 0.02 | 0.17 |
| GlycA (glycoprotein acetylation) | | | 0.074 | 0.063 | 0.24 | -0.030 | 0.022 | 0.17 | -0.019 | 0.021 | 0.36 | 0.60 |

| **PERIODONTAL POCKETS ≥ 4 mm** | | | | | | | | | | | | |
| --- | --- | --- | --- | --- | --- | --- | --- | --- | --- | --- | --- | --- |
| **Metabolite** | | | **Parogene** | | | **Health 2000** | | | **Meta-analysis** | | | |
|  | | | Beta | SE | p | Beta | SE | p | Beta | SE | p | FDR |
| VLDL | XXL | particles | 0.017 | 0.009 | 0.06 | 0.006 | 0.003 | 0.01 | 0.007 | 0.002 | 0.004 | 0.01 |
|  |  | lipid | 0.017 | 0.009 | 0.05 | 0.006 | 0.003 | 0.02 | 0.007 | 0.002 | 0.004 | 0.01 |
|  |  | phospholipid | 0.016 | 0.009 | 0.07 | 0.007 | 0.003 | 0.01 | 0.007 | 0.002 | 0.003 | 0.005 |
|  |  | cholesterol | 0.018 | 0.009 | 0.05 | 0.007 | 0.003 | 0.01 | 0.008 | 0.002 | **0.002** | 0.004 |
|  |  | cholesteryl ester | 0.019 | 0.009 | 0.03 | 0.009 | 0.003 | **0.0004** | 0.008 | 0.002 | **0.001** | 0.002 |
|  |  | free cholesterol | 0.016 | 0.009 | 0.08 | 0.011 | 0.003 | **1.5E-05** | 0.007 | 0.002 | 0.01 | 0.01 |
|  |  | triglycerides | 0.017 | 0.009 | 0.06 | 0.010 | 0.003 | **0.0002** | 0.007 | 0.002 | **0.0026** | 0.004 |
|  | XL | particles | 0.018 | 0.009 | 0.04 | 0.009 | 0.003 | **0.0004** | 0.007 | 0.002 | 0.004 | 0.01 |
|  |  | lipid | 0.019 | 0.009 | 0.03 | 0.009 | 0.003 | **0.0005** | 0.008 | 0.002 | **0.002** | 0.004 |
|  |  | phospholipid | 0.018 | 0.009 | 0.05 | 0.008 | 0.003 | **0.001** | 0.007 | 0.002 | **0.00277** | 0.005 |
|  |  | cholesterol | 0.018 | 0.009 | 0.05 | -0.005 | 0.002 | 0.04 | 0.007 | 0.002 | 0.003 | 0.005 |
|  |  | cholesteryl ester | 0.018 | 0.009 | 0.04 | -0.005 | 0.002 | 0.05 | 0.008 | 0.002 | **0.002** | 0.004 |
|  |  | free cholesterol | 0.016 | 0.009 | 0.07 | 0.001 | 0.003 | 0.78 | 0.007 | 0.002 | 0.003 | 0.005 |
|  |  | triglycerides | 0.019 | 0.009 | 0.03 | 0.004 | 0.003 | 0.14 | 0.007 | 0.002 | 0.003 | 0.005 |
|  | L | particles | 0.021 | 0.009 | 0.02 | 0.004 | 0.003 | 0.09 | 0.008 | 0.002 | **0.001** | 0.002 |
|  |  | lipid | 0.022 | 0.009 | 0.01 | -0.001 | 0.003 | 0.72 | 0.009 | 0.002 | **0.0004** | 0.001 |
|  |  | phospholipid | 0.021 | 0.009 | 0.02 | -0.006 | 0.002 | 0.01 | 0.008 | 0.002 | **0.001** | 0.002 |
|  |  | cholesterol | 0.022 | 0.009 | 0.01 | 0.001 | 0.003 | 0.75 | 0.009 | 0.002 | **0.0005** | 0.001 |
|  |  | cholesteryl ester | 0.024 | 0.009 | 0.01 | 0.011 | 0.003 | **3.0E-05** | 0.008 | 0.002 | **0.001** | 0.002 |
|  |  | free cholesterol | 0.020 | 0.009 | 0.03 | 0.011 | 0.002 | **8.6E-06** | 0.008 | 0.002 | **0.001** | 0.002 |
|  |  | triglycerides | 0.022 | 0.009 | 0.01 | 0.006 | 0.003 | 0.01 | 0.008 | 0.002 | **0.001** | 0.002 |
|  | M | particles | 0.024 | 0.009 | 0.01 | 0.007 | 0.003 | 0.01 | 0.008 | 0.002 | **0.001** | 0.002 |
|  |  | lipid | 0.026 | 0.009 | 0.004 | 0.008 | 0.003 | 0.003 | 0.009 | 0.002 | **0.0002** | 0.001 |
|  |  | phospholipid | 0.025 | 0.009 | 0.01 | 0.008 | 0.003 | **0.002** | 0.009 | 0.002 | **0.0002** | 0.001 |
|  |  | cholesterol | 0.026 | 0.009 | 0.003 | 0.010 | 0.003 | **0.0001** | 0.010 | 0.002 | **4.7E-05** | 0.0003 |
|  |  | cholesteryl ester | 0.028 | 0.009 | **0.002** | 0.011 | 0.003 | **2.6E-05** | 0.010 | 0.002 | **2.0E-05** | 0.0001 |
|  |  | free cholesterol | 0.024 | 0.009 | 0.01 | 0.009 | 0.003 | **0.0004** | 0.009 | 0.002 | **0.0005** | 0.001 |
|  |  | triglycerides | 0.024 | 0.009 | 0.01 | 0.009 | 0.003 | **0.001** | 0.008 | 0.002 | **0.001** | 0.002 |
|  | S | particles | 0.027 | 0.009 | **0.002** | 0.009 | 0.003 | **0.001** | 0.010 | 0.002 | **2.0E-05** | 0.0001 |
|  |  | lipid | 0.029 | 0.009 | **0.001** | 0.008 | 0.003 | **0.002** | 0.011 | 0.002 | **3.8E-06** | 5.0E-05 |
|  |  | phospholipid | 0.029 | 0.009 | **0.001** | -0.005 | 0.002 | 0.03 | 0.011 | 0.002 | **6.7E-06** | 6.9E-05 |
|  |  | cholesterol | 0.027 | 0.009 | **0.002** | -0.005 | 0.002 | 0.03 | 0.012 | 0.002 | **1.1E-06** | 2.5E-05 |
|  |  | cholesteryl ester | 0.025 | 0.009 | 0.01 | 0.000 | 0.003 | 0.87 | 0.012 | 0.002 | **2.2E-06** | 3.5E-05 |
|  |  | free cholesterol | 0.027 | 0.009 | **0.002** | 0.003 | 0.003 | 0.23 | 0.011 | 0.002 | **8.3E-06** | 0.0001 |
|  |  | triglycerides | 0.026 | 0.009 | 0.003 | 0.009 | 0.003 | **0.0004** | 0.009 | 0.002 | **0.0001** | 0.0004 |
|  | XS | particles | 0.020 | 0.009 | 0.03 | 0.007 | 0.003 | 0.003 | 0.012 | 0.003 | **1.9E-06** | 3.3E-05 |
|  |  | lipid | 0.020 | 0.009 | 0.03 | 0.007 | 0.003 | 0.01 | 0.012 | 0.003 | **3.1E-06** | 4.4E-05 |
|  |  | phospholipid | 0.016 | 0.009 | 0.08 | 0.006 | 0.003 | 0.02 | 0.011 | 0.003 | **8.3E-06** | 6.9E-05 |
|  |  | cholesterol | 0.015 | 0.009 | 0.09 | 0.007 | 0.003 | 0.01 | 0.010 | 0.003 | **4.6E-05** | 0.0003 |
|  |  | cholesteryl ester | 0.014 | 0.009 | 0.12 | 0.007 | 0.003 | 0.01 | 0.010 | 0.003 | **3.7E-05** | 0.0002 |
|  |  | free cholesterol | 0.015 | 0.009 | 0.10 | 0.008 | 0.003 | **0.002** | 0.010 | 0.003 | **0.0001** | 0.0004 |
|  |  | triglycerides | 0.021 | 0.009 | 0.02 | 0.010 | 0.003 | **0.0002** | 0.011 | 0.002 | **2.0E-05** | 0.0001 |
| IDL | | particles | 0.015 | 0.009 | 0.09 | 0.010 | 0.003 | **0.0001** | 0.010 | 0.003 | **5.2E-05** | 0.0003 |
|  |  | lipid | 0.016 | 0.009 | 0.07 | 0.011 | 0.003 | **2.3E-05** | 0.010 | 0.003 | **9.2E-05** | 0.0004 |
|  |  | phospholipid | 0.012 | 0.009 | 0.16 | 0.011 | 0.003 | **1.8E-05** | 0.009 | 0.003 | **0.001** | 0.002 |
|  |  | cholesterol | 0.014 | 0.009 | 0.10 | 0.011 | 0.003 | **2.1E-05** | 0.009 | 0.003 | **0.001** | 0.002 |
|  |  | cholesteryl ester | 0.016 | 0.009 | 0.07 | 0.010 | 0.003 | **0.0002** | 0.009 | 0.003 | **0.0003** | 0.001 |
|  |  | free cholesterol | 0.009 | 0.009 | 0.32 | 0.008 | 0.003 | 0.004 | 0.008 | 0.003 | **0.002** | 0.004 |
|  |  | triglycerides | 0.007 | 0.009 | 0.40 | 0.004 | 0.003 | 0.10 | 0.010 | 0.002 | **3.9E-05** | 0.0002 |
| LDL | L | particles | 0.016 | 0.009 | 0.07 | 0.000 | 0.003 | 0.95 | 0.010 | 0.003 | **0.0001** | 0.0004 |
|  |  | lipid | 0.017 | 0.009 | 0.05 | 0.009 | 0.003 | **0.001** | 0.010 | 0.003 | **0.0001** | 0.0004 |
|  |  | phospholipid | 0.018 | 0.009 | 0.04 | 0.008 | 0.003 | **0.002** | 0.010 | 0.003 | **0.0002** | 0.0007 |
|  |  | cholesterol | 0.016 | 0.009 | 0.07 | 0.008 | 0.003 | 0.005 | 0.009 | 0.003 | **0.0003** | 0.0009 |
|  |  | cholesteryl ester | 0.017 | 0.009 | 0.06 | 0.007 | 0.003 | 0.01 | 0.010 | 0.003 | **0.0001** | 0.0004 |
|  |  | free cholesterol | 0.012 | 0.009 | 0.18 | 0.008 | 0.003 | 0.003 | 0.008 | 0.003 | **0.002** | 0.004 |
|  |  | triglycerides | 0.012 | 0.009 | 0.19 | 0.011 | 0.003 | **1.9E-05** | 0.011 | 0.002 | **7.4E-06** | 0.0001 |
|  | M | particles | 0.018 | 0.009 | 0.05 | 0.011 | 0.003 | **2.8E-05** | 0.010 | 0.003 | **0.0001** | 0.0004 |
|  |  | lipid | 0.019 | 0.009 | 0.04 | 0.007 | 0.003 | 0.01 | 0.010 | 0.003 | **0.0001** | 0.0004 |
|  |  | phospholipid | 0.023 | 0.009 | 0.01 | 0.006 | 0.003 | 0.01 | 0.010 | 0.003 | **0.0001** | 0.0004 |
|  |  | cholesterol | 0.016 | 0.009 | 0.07 | 0.007 | 0.003 | 0.004 | 0.009 | 0.003 | **0.0003** | 0.0009 |
|  |  | cholesteryl ester | 0.015 | 0.009 | 0.09 | 0.009 | 0.003 | **0.001** | 0.009 | 0.003 | **0.0003** | 0.001 |
|  |  | free cholesterol | 0.017 | 0.009 | 0.05 | 0.011 | 0.003 | **2.8E-05** | 0.009 | 0.003 | **0.001** | 0.002 |
|  |  | triglycerides | 0.013 | 0.009 | 0.16 | 0.010 | 0.003 | **0.0002** | 0.011 | 0.002 | **7.5E-06** | 0.0001 |
|  | S | particles | 0.020 | 0.009 | 0.03 | 0.008 | 0.003 | **0.002** | 0.009 | 0.003 | **0.0002** | 0.0007 |
|  |  | lipid | 0.020 | 0.009 | 0.03 | 0.008 | 0.003 | **0.002** | 0.009 | 0.003 | **0.0003** | 0.001 |
|  |  | phospholipid | 0.023 | 0.009 | 0.01 | 0.009 | 0.003 | **0.001** | 0.009 | 0.003 | **0.001** | 0.002 |
|  |  | cholesterol | 0.016 | 0.009 | 0.07 | 0.008 | 0.003 | **0.001** | 0.009 | 0.003 | **0.001** | 0.002 |
|  |  | cholesteryl ester | 0.015 | 0.009 | 0.09 | 0.008 | 0.003 | **0.00277** | 0.009 | 0.003 | **0.001** | 0.002 |
|  |  | free cholesterol | 0.019 | 0.009 | 0.04 | -0.004 | 0.003 | 0.14 | 0.008 | 0.003 | 0.003 | 0.005 |
|  |  | triglycerides | 0.022 | 0.009 | 0.01 | -0.004 | 0.002 | 0.09 | 0.011 | 0.002 | **2.2E-05** | 0.0001 |
| HDL | XL | particles | -0.004 | 0.008 | 0.67 | 0.001 | 0.003 | 0.74 | -0.005 | 0.002 | 0.04 | 0.048 |
|  |  | lipid | -0.002 | 0.008 | 0.81 | -0.005 | 0.002 | 0.03 | -0.005 | 0.002 | 0.03 | 0.04 |
|  |  | phospholipid | -0.007 | 0.008 | 0.42 | -0.006 | 0.002 | 0.01 | -0.006 | 0.002 | 0.01 | 0.02 |
|  |  | cholesterol | 0.001 | 0.008 | 0.92 | -0.001 | 0.003 | 0.75 | -0.005 | 0.002 | 0.04 | 0.05 |
|  |  | cholesteryl ester | 0.002 | 0.008 | 0.81 | 0.004 | 0.003 | 0.16 | -0.005 | 0.002 | 0.05 | 0.07 |
|  |  | free cholesterol | -0.004 | 0.008 | 0.67 | 0.008 | 0.003 | 0.003 | -0.005 | 0.002 | 0.02 | 0.03 |
|  |  | triglycerides | 0.011 | 0.009 | 0.24 | 0.007 | 0.003 | 0.01 | 0.005 | 0.003 | 0.05 | 0.07 |
|  | L | particles | -0.012 | 0.008 | 0.14 | 0.007 | 0.003 | 0.005 | -0.005 | 0.002 | 0.02 | 0.03 |
|  |  | lipid | -0.012 | 0.008 | 0.14 | 0.009 | 0.003 | **0.0004** | -0.006 | 0.002 | 0.01 | 0.02 |
|  |  | phospholipid | -0.011 | 0.008 | 0.19 | 0.011 | 0.003 | **3.7E-05** | -0.005 | 0.002 | 0.04 | 0.046 |
|  |  | cholesterol | -0.014 | 0.008 | 0.09 | 0.010 | 0.003 | **0.0001** | -0.007 | 0.002 | 0.003 | 0.005 |
|  |  | cholesteryl ester | -0.014 | 0.008 | 0.09 | 0.009 | 0.003 | **0.001** | -0.007 | 0.002 | 0.004 | 0.006 |
|  |  | free cholesterol | -0.014 | 0.008 | 0.09 | 0.009 | 0.003 | **0.001** | -0.007 | 0.002 | 0.004 | 0.006 |
|  |  | triglycerides | -0.004 | 0.008 | 0.59 | 0.009 | 0.003 | **0.001** | 0.000 | 0.002 | 0.93 | 0.93 |
|  | M | particles | 0.000 | 0.009 | 0.98 | 0.008 | 0.003 | **0.002** | 0.001 | 0.003 | 0.80 | 0.83 |
|  |  | lipid | -0.001 | 0.009 | 0.90 | -0.005 | 0.002 | 0.04 | 0.000 | 0.003 | 0.90 | 0.92 |
|  |  | phospholipid | 0.000 | 0.009 | 0.99 | -0.006 | 0.002 | 0.01 | 0.001 | 0.003 | 0.79 | 0.83 |
|  |  | cholesterol | -0.005 | 0.009 | 0.55 | -0.001 | 0.003 | 0.69 | -0.001 | 0.003 | 0.64 | 0.68 |
|  |  | cholesteryl ester | -0.006 | 0.009 | 0.51 | 0.004 | 0.003 | 0.12 | -0.001 | 0.003 | 0.57 | 0.62 |
|  |  | free cholesterol | -0.003 | 0.009 | 0.73 | 0.006 | 0.003 | 0.02 | 0.000 | 0.003 | 0.91 | 0.92 |
|  |  | triglycerides | 0.025 | 0.009 | 0.01 | 0.007 | 0.003 | 0.01 | 0.010 | 0.003 | **0.0001** | 0.0004 |
|  | S | particles | 0.013 | 0.009 | 0.15 | 0.007 | 0.003 | 0.01 | 0.005 | 0.003 | 0.07 | 0.08 |
|  |  | lipid | 0.012 | 0.009 | 0.16 | 0.007 | 0.003 | 0.004 | 0.004 | 0.003 | 0.12 | 0.14 |
|  |  | phospholipid | 0.010 | 0.009 | 0.28 | 0.010 | 0.003 | **0.0002** | 0.002 | 0.003 | 0.46 | 0.52 |
|  |  | cholesterol | 0.007 | 0.009 | 0.41 | 0.009 | 0.003 | **0.001** | 0.004 | 0.003 | 0.11 | 0.13 |
|  |  | cholesteryl ester | 0.007 | 0.009 | 0.45 | 0.008 | 0.003 | 0.004 | 0.004 | 0.003 | 0.09 | 0.11 |
|  |  | free cholesterol | 0.006 | 0.009 | 0.52 | 0.008 | 0.003 | 0.004 | 0.002 | 0.003 | 0.49 | 0.54 |
|  |  | triglycerides | 0.024 | 0.009 | 0.01 | 0.008 | 0.003 | 0.003 | 0.009 | 0.002 | **0.0002** | 0.001 |
| VLDL diameter | | | 0.021 | 0.009 | 0.02 | 0.007 | 0.003 | 0.01 | 0.006 | 0.002 | 0.02 | 0.03 |
| LDL diameter | | | -0.010 | 0.009 | 0.27 | -0.005 | 0.002 | 0.03 | -0.002 | 0.002 | 0.51 | 0.57 |
| HDL diameter | | | -0.015 | 0.008 | 0.08 | -0.006 | 0.002 | 0.01 | -0.007 | 0.002 | **0.0027** | 0.005 |
| Cholesterol | serum total | | 0.017 | 0.009 | 0.06 | 0.000 | 0.003 | 0.99 | 0.008 | 0.003 | **0.001** | 0.002 |
|  | VLDL | | 0.028 | 0.009 | **0.002** | 0.001 | 0.003 | 0.59 | 0.012 | 0.002 | **1.0E-06** | 2.5E-05 |
|  | remnant | | 0.026 | 0.009 | 0.004 | 0.007 | 0.003 | 0.01 | 0.012 | 0.003 | **8.4E-07** | 2.5E-05 |
|  | LDL | | 0.016 | 0.009 | 0.07 | 0.007 | 0.003 | 0.01 | 0.009 | 0.003 | **0.0004** | 0.001 |
|  | HDL | | -0.010 | 0.008 | 0.25 | 0.007 | 0.003 | 0.004 | -0.004 | 0.002 | 0.08 | 0.10 |
|  | HDL2 | | -0.011 | 0.008 | 0.19 | 0.008 | 0.003 | **0.002** | -0.005 | 0.002 | 0.05 | 0.06 |
|  | HDL3 | | 0.005 | 0.009 | 0.56 | 0.010 | 0.003 | **0.0002** | 0.001 | 0.002 | 0.63 | 0.68 |
|  | esterified | | 0.018 | 0.009 | 0.05 | 0.011 | 0.003 | **3.8E-05** | 0.008 | 0.003 | **0.001** | 0.002 |
|  | free | | 0.017 | 0.009 | 0.06 | 0.008 | 0.003 | **0.001** | 0.009 | 0.003 | **0.001** | 0.002 |
| Triglycerides | serum total | | 0.024 | 0.009 | 0.01 | 0.009 | 0.003 | **0.001** | 0.010 | 0.002 | **2.8E-05** | 0.000 |
|  | VLDL | | 0.024 | 0.009 | 0.01 | 0.009 | 0.003 | **0.001** | 0.009 | 0.002 | **0.0003** | 0.000 |
|  | LDL | | 0.011 | 0.009 | 0.20 | 0.007 | 0.003 | 0.01 | 0.011 | 0.002 | **9.7E-06** | 0.000 |
|  | HDL | | 0.021 | 0.009 | 0.02 | -0.006 | 0.002 | 0.02 | 0.009 | 0.003 | **0.001** | 0.002 |
| Total phosphoglycerides | | | 0.013 | 0.009 | 0.13 | -0.004 | 0.002 | 0.07 | 0.008 | 0.003 | **0.0026** | 0.004 |
| Phosphatidylcholine | | | 0.013 | 0.009 | 0.13 | 0.001 | 0.003 | 0.79 | 0.007 | 0.003 | 0.003 | 0.005 |
| Sphingomyelins | | | 0.007 | 0.009 | 0.41 | 0.001 | 0.003 | 0.66 | 0.006 | 0.003 | 0.02 | 0.03 |
| Total cholines | | | 0.011 | 0.009 | 0.21 | 0.007 | 0.003 | 0.01 | 0.007 | 0.003 | 0.004 | 0.006 |
| Apolipoprotein A-I | | | 0.000 | 0.009 | 0.98 | 0.007 | 0.003 | 0.01 | 0.001 | 0.003 | 0.77 | 0.81 |
| Apolipoprotein B | | | 0.027 | 0.009 | 0.003 | 0.005 | 0.003 | 0.04 | 0.012 | 0.002 | **1.3E-06** | 2.6E-05 |
| Apo-B / apoA-I | | | 0.025 | 0.009 | 0.004 | 0.007 | 0.003 | 0.01 | 0.012 | 0.002 | **3.8E-07** | 1.5E-05 |
| Fatty acids | total | | 0.021 | 0.009 | 0.02 | 0.010 | 0.003 | **0.0002** | 0.011 | 0.003 | **2.4E-05** | 0.0002 |
|  | unsaturated | | -0.017 | 0.009 | 0.06 | 0.007 | 0.003 | 0.01 | -0.007 | 0.002 | 0.01 | 0.01 |
|  | DHA (docosahexaenoic acid) | | 0.000 | 0.009 | 0.96 | 0.007 | 0.003 | 0.01 | 0.006 | 0.003 | 0.02 | 0.02 |
|  | LA (linoleic acid) | | 0.021 | 0.009 | 0.02 | 0.008 | 0.003 | **0.0027** | 0.009 | 0.003 | **0.001** | 0.002 |
|  | omega-3 | | 0.004 | 0.009 | 0.65 | 0.008 | 0.003 | 0.004 | 0.007 | 0.003 | 0.01 | 0.01 |
|  | omega-6 | | 0.019 | 0.009 | 0.03 | 0.008 | 0.003 | **0.002** | 0.009 | 0.003 | **0.0005** | 0.001 |
|  | PUFA (polyunsaturated) | | 0.017 | 0.009 | 0.06 | 0.011 | 0.003 | **5.2E-05** | 0.009 | 0.003 | **0.001** | 0.002 |
|  | MUFA (monounsaturated) | | 0.023 | 0.009 | 0.01 | 0.010 | 0.003 | **0.0003** | 0.012 | 0.003 | **4.1E-06** | 5.0E-05 |
|  | SAFA (saturated) | | 0.019 | 0.009 | 0.04 | 0.002 | 0.003 | 0.39 | 0.010 | 0.003 | **0.0001** | 0.0004 |
|  | DHA% | | -0.013 | 0.009 | 0.15 | 0.001 | 0.003 | 0.69 | 0.000 | 0.003 | 0.97 | 0.97 |
|  | LA% | | 0.001 | 0.009 | 0.92 | -0.008 | 0.003 | 0.003 | -0.005 | 0.003 | 0.04 | 0.05 |
|  | omega-3% | | -0.009 | 0.009 | 0.31 | -0.006 | 0.003 | 0.03 | 0.001 | 0.003 | 0.59 | 0.64 |
|  | omega-6% | | -0.007 | 0.009 | 0.41 | -0.007 | 0.003 | 0.01 | -0.008 | 0.002 | **0.002** | 0.004 |
|  | PUFA% | | -0.009 | 0.009 | 0.30 | 0.008 | 0.003 | 0.003 | -0.007 | 0.002 | 0.01 | 0.01 |
|  | MUFA% | | 0.020 | 0.009 | 0.02 | 0.001 | 0.003 | 0.82 | 0.009 | 0.002 | **0.0005** | 0.001 |
|  | SAFA% | | -0.011 | 0.009 | 0.21 | -0.006 | 0.003 | 0.02 | 0.000 | 0.003 | 0.89 | 0.92 |
| Glucose | | | -0.009 | 0.008 | 0.30 | 0.004 | 0.003 | 0.17 | 0.006 | 0.002 | 0.02 | 0.03 |
| Lactate | | | 0.003 | 0.009 | 0.71 | -0.002 | 0.003 | 0.50 | 0.007 | 0.002 | 0.004 | 5.9E-03 |
| Pyruvate | | | 0.000 | 0.009 | 0.97 | 0.007 | 0.003 | 0.01 | 0.011 | 0.002 | **1.1E-05** | 8.6E-05 |
| Citrate | | | 0.005 | 0.009 | 0.55 | 0.001 | 0.003 | 0.64 | -0.008 | 0.002 | **0.001** | 0.002 |
| Glycerol | | | 0.013 | 0.009 | 0.16 | 0.007 | 0.003 | 0.004 | 0.006 | 0.003 | 0.02 | 0.03 |
| Alanine | | | 0.003 | 0.009 | 0.75 | 0.008 | 0.003 | **0.002** | 0.004 | 0.003 | 0.16 | 0.18 |
| Glutamine | | | -0.004 | 0.009 | 0.65 | 0.008 | 0.003 | **0.001** | -0.002 | 0.003 | 0.44 | 0.50 |
| Glycine | | | -0.019 | 0.008 | 0.02 | 0.010 | 0.003 | **0.0002** | 0.004 | 0.002 | 0.09 | 0.11 |
| Histidine | | | -0.008 | 0.009 | 0.37 | 0.009 | 0.003 | **0.001** | 0.000 | 0.003 | 0.85 | 0.88 |
| Isoleucine | | | 0.021 | 0.009 | 0.02 | 0.007 | 0.002 | 0.01 | 0.008 | 0.002 | **0.001** | 0.002 |
| Leucine | | | 0.015 | 0.009 | 0.08 | 0.007 | 0.003 | 0.004 | 0.008 | 0.002 | **0.001** | 0.002 |
| Valine | | | 0.014 | 0.009 | 0.11 | 0.012 | 0.003 | **5.3E-06** | 0.009 | 0.002 | **0.0004** | 0.001 |
| Phenylalanine | | | -0.008 | 0.009 | 0.39 | -0.009 | 0.002 | **0.0002** | 0.008 | 0.003 | **0.001** | 0.002 |
| Tyrosine | | | 0.006 | 0.009 | 0.52 | 0.006 | 0.003 | 0.05 | 0.008 | 0.002 | **0.001** | 0.002 |
| Acetate | | | 0.007 | 0.009 | 0.42 | 0.007 | 0.003 | 0.01 | 0.007 | 0.003 | 0.01 | 0.01 |
| Acetoacetate | | | -0.001 | 0.009 | 0.88 | 0.007 | 0.003 | 0.01 | 0.006 | 0.003 | 0.02 | 0.02 |
| 3-hydroxybutyrate | | | -0.021 | 0.009 | 0.02 | 0.003 | 0.003 | 0.25 | 0.001 | 0.003 | 0.67 | 0.71 |
| Creatinine | | | 0.005 | 0.009 | 0.61 | 0.001 | 0.003 | 0.71 | -0.004 | 0.002 | 0.09 | 0.11 |
| Albumin | | | 0.001 | 0.009 | 0.94 | -0.005 | 0.002 | 0.06 | 0.001 | 0.003 | 0.70 | 0.75 |
| GlycA (glycoprotein acetylation) | | | 0.010 | 0.009 | 0.26 | 0.012 | 0.003 | **9.2E-06** | 0.011 | 0.003 | **4.8E-06** | 5.4E-05 |

| **PERIODONTAL POCKETS ≥ 4 mm + BOP** | | | | | | | | | | | | |
| --- | --- | --- | --- | --- | --- | --- | --- | --- | --- | --- | --- | --- |
| **Metabolite** | | | **Parogene** | | | **Health 2000** | | | **Meta-analysis** | | | |
|  | | | Beta | SE | p | Beta | SE | p | Beta | SE | p | FDR |
| VLDL | XXL | particles | 0.017 | 0.009 | 0.05 | 0.007 | 0.003 | 0.01 | 0.008 | 0.003 | **0.001** | 0.002 |
|  |  | lipid | 0.017 | 0.009 | 0.05 | 0.007 | 0.003 | 0.01 | 0.008 | 0.003 | **0.002** | 0.004 |
|  |  | phospholipid | 0.016 | 0.009 | 0.07 | 0.008 | 0.003 | 0.004 | 0.009 | 0.003 | **0.001** | 0.002 |
|  |  | cholesterol | 0.018 | 0.009 | 0.04 | 0.008 | 0.003 | 0.01 | 0.008 | 0.003 | **0.001** | 0.002 |
|  |  | cholesteryl ester | 0.019 | 0.009 | 0.03 | 0.008 | 0.003 | **0.002** | 0.009 | 0.003 | **0.0004** | 0.001 |
|  |  | free cholesterol | 0.016 | 0.009 | 0.07 | 0.007 | 0.003 | 0.01 | 0.008 | 0.003 | 0.003 | 0.01 |
|  |  | triglycerides | 0.017 | 0.009 | 0.05 | 0.008 | 0.003 | 0.003 | 0.009 | 0.003 | **0.001** | 0.002 |
|  | XL | particles | 0.019 | 0.009 | 0.03 | 0.007 | 0.003 | 0.01 | 0.008 | 0.003 | **0.001** | 0.002 |
|  |  | lipid | 0.020 | 0.009 | 0.02 | 0.008 | 0.003 | 0.01 | 0.009 | 0.003 | **0.001** | 0.002 |
|  |  | phospholipid | 0.018 | 0.009 | 0.04 | 0.007 | 0.003 | 0.01 | 0.008 | 0.003 | **0.001** | 0.002 |
|  |  | cholesterol | 0.018 | 0.009 | 0.04 | 0.007 | 0.003 | 0.01 | 0.008 | 0.003 | **0.001** | 0.002 |
|  |  | cholesteryl ester | 0.019 | 0.009 | 0.03 | 0.008 | 0.003 | 0.01 | 0.008 | 0.003 | **0.001** | 0.002 |
|  |  | free cholesterol | 0.017 | 0.009 | 0.06 | 0.007 | 0.003 | 0.01 | 0.008 | 0.003 | **0.002** | 0.004 |
|  |  | triglycerides | 0.020 | 0.009 | 0.02 | 0.007 | 0.003 | 0.01 | 0.008 | 0.003 | **0.001** | 0.002 |
|  | L | particles | 0.022 | 0.009 | 0.01 | 0.008 | 0.003 | **0.0025** | 0.009 | 0.003 | **0.0003** | 0.001 |
|  |  | lipid | 0.023 | 0.009 | 0.01 | 0.009 | 0.003 | **0.001** | 0.010 | 0.003 | **0.0001** | 0.0004 |
|  |  | phospholipid | 0.022 | 0.009 | 0.01 | 0.009 | 0.003 | **0.001** | 0.010 | 0.003 | **0.0002** | 0.001 |
|  |  | cholesterol | 0.023 | 0.009 | 0.01 | 0.009 | 0.003 | **0.001** | 0.010 | 0.003 | **0.0001** | 0.0004 |
|  |  | cholesteryl ester | 0.024 | 0.009 | 0.01 | 0.008 | 0.003 | **0.002** | 0.010 | 0.003 | **0.0001** | 0.0004 |
|  |  | free cholesterol | 0.020 | 0.009 | 0.02 | 0.008 | 0.003 | **0.0026** | 0.009 | 0.003 | **0.0004** | 0.001 |
|  |  | triglycerides | 0.023 | 0.009 | 0.01 | 0.008 | 0.003 | **0.0025** | 0.009 | 0.003 | **0.0003** | 0.001 |
|  | M | particles | 0.024 | 0.009 | 0.01 | 0.008 | 0.003 | 0.004 | 0.009 | 0.003 | **0.0004** | 0.001 |
|  |  | lipid | 0.026 | 0.009 | 0.003 | 0.009 | 0.003 | **0.001** | 0.010 | 0.003 | **0.0001** | 0.0004 |
|  |  | phospholipid | 0.025 | 0.009 | 0.004 | 0.009 | 0.003 | **0.001** | 0.010 | 0.003 | **0.0001** | 0.0004 |
|  |  | cholesterol | 0.027 | 0.009 | **0.002** | 0.009 | 0.003 | **0.0005** | 0.011 | 0.003 | **2.3E-05** | 0.0002 |
|  |  | cholesteryl ester | 0.028 | 0.009 | **0.002** | 0.010 | 0.003 | **0.0003** | 0.011 | 0.003 | **1.3E-05** | 0.0001 |
|  |  | free cholesterol | 0.024 | 0.009 | 0.01 | 0.008 | 0.003 | **0.002** | 0.010 | 0.003 | **0.0002** | 0.001 |
|  |  | triglycerides | 0.025 | 0.009 | 0.005 | 0.008 | 0.003 | 0.003 | 0.009 | 0.003 | **0.0003** | 0.001 |
|  | S | particles | 0.028 | 0.009 | **0.002** | 0.010 | 0.003 | **0.0003** | 0.011 | 0.003 | **9.5E-06** | 0.0001 |
|  |  | lipid | 0.029 | 0.009 | **0.001** | 0.011 | 0.003 | **0.0001** | 0.012 | 0.003 | **2.2E-06** | 6.5E-05 |
|  |  | phospholipid | 0.029 | 0.009 | **0.001** | 0.010 | 0.003 | **0.0001** | 0.012 | 0.003 | **2.7E-06** | 6.5E-05 |
|  |  | cholesterol | 0.027 | 0.009 | **0.002** | 0.011 | 0.003 | **0.0001** | 0.012 | 0.003 | **1.9E-06** | 6.5E-05 |
|  |  | cholesteryl ester | 0.025 | 0.009 | 0.01 | 0.011 | 0.003 | **0.0001** | 0.012 | 0.003 | **6.1E-06** | 8.7E-05 |
|  |  | free cholesterol | 0.028 | 0.009 | **0.002** | 0.010 | 0.003 | **0.0002** | 0.012 | 0.003 | **6.1E-06** | 8.7E-05 |
|  |  | triglycerides | 0.027 | 0.009 | **0.002** | 0.009 | 0.003 | **0.001** | 0.010 | 0.003 | **4.7E-05** | 0.0003 |
|  | XS | particles | 0.020 | 0.009 | 0.03 | 0.011 | 0.003 | **0.0001** | 0.012 | 0.003 | **7.5E-06** | 9.8E-05 |
|  |  | lipid | 0.020 | 0.009 | 0.03 | 0.010 | 0.003 | **0.0002** | 0.011 | 0.003 | **1.9E-05** | 0.0002 |
|  |  | phospholipid | 0.016 | 0.009 | 0.08 | 0.010 | 0.003 | **0.0003** | 0.011 | 0.003 | **6.3E-05** | 0.0004 |
|  |  | cholesterol | 0.015 | 0.009 | 0.09 | 0.009 | 0.003 | **0.001** | 0.010 | 0.003 | **0.0003** | 0.001 |
|  |  | cholesteryl ester | 0.013 | 0.009 | 0.12 | 0.009 | 0.003 | **0.001** | 0.010 | 0.003 | **0.0002** | 0.001 |
|  |  | free cholesterol | 0.015 | 0.009 | 0.10 | 0.008 | 0.003 | 0.004 | 0.009 | 0.003 | **0.001** | 0.002 |
|  |  | triglycerides | 0.022 | 0.009 | 0.01 | 0.010 | 0.003 | **0.0002** | 0.011 | 0.003 | **1.9E-05** | 0.0002 |
| IDL | | particles | 0.014 | 0.009 | 0.11 | 0.009 | 0.003 | **0.001** | 0.009 | 0.003 | **0.0004** | 0.001 |
|  |  | lipid | 0.015 | 0.009 | 0.10 | 0.008 | 0.003 | **0.0025** | 0.009 | 0.003 | **0.001** | 0.002 |
|  |  | phospholipid | 0.011 | 0.009 | 0.20 | 0.007 | 0.003 | 0.01 | 0.008 | 0.003 | 0.004 | 0.01 |
|  |  | cholesterol | 0.013 | 0.009 | 0.13 | 0.007 | 0.003 | 0.01 | 0.008 | 0.003 | 0.004 | 0.01 |
|  |  | cholesteryl ester | 0.015 | 0.009 | 0.09 | 0.008 | 0.003 | 0.01 | 0.008 | 0.003 | **0.002** | 0.004 |
|  |  | free cholesterol | 0.008 | 0.009 | 0.38 | 0.006 | 0.003 | 0.02 | 0.006 | 0.003 | 0.02 | 0.03 |
|  |  | triglycerides | 0.008 | 0.009 | 0.38 | 0.010 | 0.003 | **0.0002** | 0.010 | 0.003 | **0.0001** | 0.0004 |
| LDL | L | particles | 0.015 | 0.009 | 0.08 | 0.008 | 0.003 | **0.0025** | 0.009 | 0.003 | **0.001** | 0.002 |
|  |  | lipid | 0.016 | 0.009 | 0.07 | 0.008 | 0.003 | 0.005 | 0.009 | 0.003 | **0.001** | 0.002 |
|  |  | phospholipid | 0.017 | 0.009 | 0.06 | 0.008 | 0.003 | 0.005 | 0.009 | 0.003 | **0.001** | 0.002 |
|  |  | cholesterol | 0.015 | 0.009 | 0.10 | 0.007 | 0.003 | 0.01 | 0.008 | 0.003 | **0.002** | 0.004 |
|  |  | cholesteryl ester | 0.016 | 0.009 | 0.08 | 0.008 | 0.003 | 0.004 | 0.009 | 0.003 | **0.001** | 0.002 |
|  |  | free cholesterol | 0.010 | 0.009 | 0.23 | 0.006 | 0.003 | 0.02 | 0.007 | 0.003 | 0.01 | 0.01 |
|  |  | triglycerides | 0.011 | 0.009 | 0.22 | 0.011 | 0.003 | **0.0001** | 0.011 | 0.003 | **3.0E-05** | 0.0002 |
|  | M | particles | 0.017 | 0.009 | 0.05 | 0.008 | 0.003 | 0.003 | 0.009 | 0.003 | **0.001** | 0.002 |
|  |  | lipid | 0.018 | 0.009 | 0.05 | 0.008 | 0.003 | 0.01 | 0.009 | 0.003 | **0.001** | 0.002 |
|  |  | phospholipid | 0.022 | 0.009 | 0.01 | 0.008 | 0.003 | 0.003 | 0.009 | 0.003 | **0.0004** | 0.001 |
|  |  | cholesterol | 0.015 | 0.009 | 0.08 | 0.007 | 0.003 | 0.01 | 0.008 | 0.003 | **0.0027** | 0.005 |
|  |  | cholesteryl ester | 0.015 | 0.009 | 0.10 | 0.007 | 0.003 | 0.01 | 0.008 | 0.003 | **0.002** | 0.004 |
|  |  | free cholesterol | 0.016 | 0.009 | 0.06 | 0.007 | 0.003 | 0.02 | 0.007 | 0.003 | 0.01 | 0.01 |
|  |  | triglycerides | 0.012 | 0.009 | 0.18 | 0.010 | 0.003 | **0.0001** | 0.011 | 0.003 | **3.8E-05** | 0.0002 |
|  | S | particles | 0.019 | 0.009 | 0.03 | 0.007 | 0.003 | 0.01 | 0.008 | 0.003 | **0.001** | 0.002 |
|  |  | lipid | 0.019 | 0.009 | 0.03 | 0.007 | 0.003 | 0.01 | 0.008 | 0.003 | **0.002** | 0.004 |
|  |  | phospholipid | 0.022 | 0.009 | 0.01 | 0.006 | 0.003 | 0.02 | 0.008 | 0.003 | **0.0027** | 0.005 |
|  |  | cholesterol | 0.015 | 0.009 | 0.08 | 0.007 | 0.003 | 0.02 | 0.007 | 0.003 | 0.01 | 0.01 |
|  |  | cholesteryl ester | 0.014 | 0.009 | 0.10 | 0.007 | 0.003 | 0.01 | 0.008 | 0.003 | 0.004 | 0.01 |
|  |  | free cholesterol | 0.018 | 0.009 | 0.04 | 0.005 | 0.003 | 0.06 | 0.006 | 0.003 | 0.02 | 0.03 |
|  |  | triglycerides | 0.022 | 0.009 | 0.01 | 0.009 | 0.003 | **0.001** | 0.010 | 0.003 | **0.0001** | 0.0004 |
| HDL | XL | particles | -0.005 | 0.008 | 0.55 | -0.007 | 0.003 | 0.01 | -0.007 | 0.002 | 0.01 | 0.01 |
|  |  | lipid | -0.003 | 0.008 | 0.68 | -0.007 | 0.003 | 0.01 | -0.007 | 0.002 | 0.01 | 0.01 |
|  |  | phospholipid | -0.008 | 0.008 | 0.33 | -0.007 | 0.003 | 0.004 | -0.007 | 0.002 | **0.002** | 0.004 |
|  |  | cholesterol | 0.000 | 0.008 | 0.96 | -0.007 | 0.003 | 0.01 | -0.007 | 0.002 | 0.01 | 0.01 |
|  |  | cholesteryl ester | 0.001 | 0.008 | 0.93 | -0.007 | 0.003 | 0.01 | -0.006 | 0.003 | 0.01 | 0.01 |
|  |  | free cholesterol | -0.005 | 0.008 | 0.58 | -0.007 | 0.003 | 0.01 | -0.007 | 0.002 | 0.005 | 0.01 |
|  |  | triglycerides | 0.009 | 0.009 | 0.30 | 0.003 | 0.003 | 0.25 | 0.004 | 0.003 | 0.16 | 0.19 |
|  | L | particles | -0.014 | 0.008 | 0.10 | -0.007 | 0.003 | 0.01 | -0.007 | 0.002 | 0.003 | 0.01 |
|  |  | lipid | -0.014 | 0.008 | 0.09 | -0.008 | 0.003 | 0.003 | -0.008 | 0.002 | **0.001** | 0.002 |
|  |  | phospholipid | -0.012 | 0.008 | 0.14 | -0.006 | 0.003 | 0.01 | -0.007 | 0.002 | 0.005 | 0.01 |
|  |  | cholesterol | -0.016 | 0.008 | 0.06 | -0.008 | 0.003 | **0.001** | -0.009 | 0.002 | **0.0002** | 0.001 |
|  |  | cholesteryl ester | -0.016 | 0.008 | 0.06 | -0.008 | 0.003 | **0.001** | -0.009 | 0.002 | **0.0003** | 0.001 |
|  |  | free cholesterol | -0.016 | 0.008 | 0.06 | -0.008 | 0.003 | **0.001** | -0.009 | 0.002 | **0.0003** | 0.001 |
|  |  | triglycerides | -0.007 | 0.008 | 0.42 | -0.002 | 0.003 | 0.49 | -0.002 | 0.003 | 0.37 | 0.41 |
|  | M | particles | -0.001 | 0.009 | 0.91 | 0.000 | 0.003 | 0.96 | 0.000 | 0.003 | 0.99 | 0.996 |
|  |  | lipid | -0.002 | 0.009 | 0.82 | 0.000 | 0.003 | 0.88 | -0.001 | 0.003 | 0.83 | 0.85 |
|  |  | phospholipid | -0.001 | 0.009 | 0.93 | 0.000 | 0.003 | 0.98 | 0.000 | 0.003 | 1.00 | 1.00 |
|  |  | cholesterol | -0.006 | 0.009 | 0.49 | -0.002 | 0.003 | 0.49 | -0.002 | 0.003 | 0.38 | 0.42 |
|  |  | cholesteryl ester | -0.006 | 0.009 | 0.46 | -0.002 | 0.003 | 0.44 | -0.003 | 0.003 | 0.33 | 0.37 |
|  |  | free cholesterol | -0.004 | 0.009 | 0.65 | -0.001 | 0.003 | 0.74 | -0.001 | 0.003 | 0.65 | 0.69 |
|  |  | triglycerides | 0.025 | 0.009 | 0.01 | 0.010 | 0.003 | **0.0003** | 0.011 | 0.003 | **1.8E-05** | 0.0002 |
|  | S | particles | 0.013 | 0.009 | 0.14 | 0.004 | 0.003 | 0.13 | 0.005 | 0.003 | 0.06 | 0.07 |
|  |  | lipid | 0.012 | 0.009 | 0.16 | 0.003 | 0.003 | 0.25 | 0.004 | 0.003 | 0.13 | 0.15 |
|  |  | phospholipid | 0.010 | 0.009 | 0.27 | 0.002 | 0.003 | 0.53 | 0.002 | 0.003 | 0.35 | 0.39 |
|  |  | cholesterol | 0.007 | 0.009 | 0.43 | 0.003 | 0.003 | 0.27 | 0.003 | 0.003 | 0.19 | 0.22 |
|  |  | cholesteryl ester | 0.006 | 0.009 | 0.48 | 0.003 | 0.003 | 0.23 | 0.004 | 0.003 | 0.17 | 0.20 |
|  |  | free cholesterol | 0.006 | 0.009 | 0.49 | 0.001 | 0.003 | 0.64 | 0.002 | 0.003 | 0.51 | 0.55 |
|  |  | triglycerides | 0.025 | 0.009 | 0.005 | 0.010 | 0.003 | **0.0003** | 0.011 | 0.003 | **1.9E-05** | 0.0002 |
| VLDL diameter | | | 0.021 | 0.009 | 0.01 | 0.006 | 0.003 | 0.03 | 0.007 | 0.003 | 0.01 | 0.01 |
| LDL diameter | | | -0.011 | 0.009 | 0.22 | -0.001 | 0.003 | 0.83 | -0.001 | 0.003 | 0.57 | 0.61 |
| HDL diameter | | | -0.016 | 0.008 | 0.05 | -0.009 | 0.003 | **0.001** | -0.009 | 0.002 | **0.0001** | 0.0004 |
| Cholesterol | serum total | | 0.016 | 0.009 | 0.07 | 0.006 | 0.003 | 0.02 | 0.007 | 0.003 | 0.01 | 0.01 |
|  | VLDL | | 0.028 | 0.009 | **0.002** | 0.011 | 0.003 | **3.9E-05** | 0.012 | 0.003 | **1.2E-06** | 6.3E-05 |
|  | remnant | | 0.025 | 0.009 | 0.004 | 0.011 | 0.003 | **0.0001** | 0.012 | 0.003 | **2.9E-06** | 6.5E-05 |
|  | LDL | | 0.015 | 0.009 | 0.08 | 0.007 | 0.003 | 0.01 | 0.008 | 0.003 | 0.003 | 0.01 |
|  | HDL | | -0.011 | 0.008 | 0.19 | -0.006 | 0.003 | 0.03 | -0.006 | 0.003 | 0.01 | 0.01 |
|  | HDL2 | | -0.012 | 0.008 | 0.14 | -0.006 | 0.003 | 0.02 | -0.007 | 0.003 | 0.01 | 0.01 |
|  | HDL3 | | 0.003 | 0.008 | 0.69 | -0.001 | 0.003 | 0.73 | -0.001 | 0.003 | 0.83 | 0.85 |
|  | esterified | | 0.016 | 0.009 | 0.07 | 0.006 | 0.003 | 0.03 | 0.007 | 0.003 | 0.01 | 0.01 |
|  | free | | 0.015 | 0.009 | 0.08 | 0.007 | 0.003 | 0.02 | 0.007 | 0.003 | 0.01 | 0.01 |
| Triglycerides | serum total | | 0.025 | 0.009 | 0.005 | 0.010 | 0.003 | **0.0002** | 0.011 | 0.003 | **1.2E-05** | 0.0001 |
|  | VLDL | | 0.025 | 0.009 | 0.005 | 0.009 | 0.003 | **0.001** | 0.010 | 0.003 | **0.0001** | 0.0004 |
|  | LDL | | 0.011 | 0.009 | 0.23 | 0.010 | 0.003 | **0.0001** | 0.011 | 0.003 | **4.6E-05** | 0.0003 |
|  | HDL | | 0.020 | 0.009 | 0.02 | 0.008 | 0.003 | 0.003 | 0.009 | 0.003 | **0.0005** | 0.0014 |
| Total phosphoglycerides | | | 0.013 | 0.009 | 0.15 | 0.006 | 0.003 | 0.04 | 0.006 | 0.003 | 0.02 | 0.03 |
| Phosphatidylcholine | | | 0.013 | 0.009 | 0.15 | 0.005 | 0.003 | 0.06 | 0.006 | 0.003 | 0.02 | 0.03 |
| Sphingomyelins | | | 0.006 | 0.009 | 0.46 | 0.004 | 0.003 | 0.18 | 0.004 | 0.003 | 0.13 | 0.15 |
| Total cholines | | | 0.010 | 0.009 | 0.23 | 0.005 | 0.003 | 0.07 | 0.006 | 0.003 | 0.04 | 0.049 |
| Apolipoprotein A-I | | | -0.002 | 0.008 | 0.85 | -0.001 | 0.003 | 0.708 | -0.001 | 0.003 | 0.68 | 0.72 |
| Apolipoprotein B | | | 0.026 | 0.009 | 0.003 | 0.011 | 0.003 | **0.0001** | 0.012 | 0.003 | **3.5E-06** | 6.9E-05 |
| Apo-B / apoA-I | | | 0.026 | 0.009 | 0.003 | 0.012 | 0.003 | **2.5E-06** | 0.013 | 0.002 | **7.8E-08** | 6.1E-06 |
| Fatty acids | total | | 0.022 | 0.009 | 0.02 | 0.009 | 0.003 | **0.001** | 0.010 | 0.003 | **0.0001** | 0.0004 |
|  | unsaturated | | -0.016 | 0.009 | 0.07 | -0.008 | 0.003 | 0.003 | -0.009 | 0.003 | **0.001** | 0.002 |
|  | DHA (docosahexaenoic acid) | | 0.000 | 0.009 | 0.96 | 0.005 | 0.003 | 0.06 | 0.005 | 0.003 | 0.07 | 0.08 |
|  | LA (linoleic acid) | | 0.020 | 0.009 | 0.02 | 0.006 | 0.003 | 0.03 | 0.007 | 0.003 | 0.01 | 0.01 |
|  | omega-3 | | 0.005 | 0.009 | 0.54 | 0.006 | 0.003 | 0.05 | 0.006 | 0.003 | 0.04 | 0.049 |
|  | omega-6 | | 0.019 | 0.009 | 0.03 | 0.006 | 0.003 | 0.02 | 0.008 | 0.003 | 0.005 | 0.01 |
|  | PUFA (polyunsaturated) | | 0.017 | 0.009 | 0.05 | 0.006 | 0.003 | 0.02 | 0.007 | 0.003 | 0.01 | 0.01 |
|  | MUFA (monounsaturated) | | 0.023 | 0.009 | 0.01 | 0.011 | 0.003 | **0.0001** | 0.012 | 0.003 | **5.2E-06** | 8.7E-05 |
|  | SAFA (saturated) | | 0.019 | 0.009 | 0.04 | 0.009 | 0.003 | **0.001** | 0.010 | 0.003 | **0.0002** | 0.001 |
|  | DHA% | | -0.012 | 0.009 | 0.17 | -0.001 | 0.003 | 0.71 | -0.002 | 0.003 | 0.44 | 0.48 |
|  | LA% | | 0.000 | 0.009 | 0.98 | -0.007 | 0.003 | 0.01 | -0.007 | 0.003 | 0.01 | 0.01 |
|  | omega-3% | | -0.008 | 0.009 | 0.37 | 0.000 | 0.003 | 0.93 | -0.001 | 0.003 | 0.73 | 0.76 |
|  | omega-6% | | -0.008 | 0.009 | 0.38 | -0.010 | 0.003 | **0.0004** | -0.009 | 0.003 | **0.0003** | 0.001 |
|  | PUFA% | | -0.009 | 0.009 | 0.30 | -0.009 | 0.003 | **0.001** | -0.009 | 0.003 | **0.0005** | 0.001 |
|  | MUFA% | | 0.020 | 0.009 | 0.02 | 0.010 | 0.003 | **0.0002** | 0.011 | 0.003 | **2.13E-05** | 0.0002 |
|  | SAFA% | | -0.012 | 0.009 | 0.21 | 0.002 | 0.003 | 0.57 | 0.000 | 0.003 | 0.87 | 0.88 |
| Glucose | | | -0.011 | 0.008 | 0.18 | 0.007 | 0.003 | 0.01 | 0.005 | 0.003 | 0.04 | 0.049 |
| Lactate | | | 0.001 | 0.009 | 0.92 | 0.008 | 0.003 | 0.003 | 0.007 | 0.003 | 0.005 | 0.01 |
| Pyruvate | | | -0.001 | 0.009 | 0.94 | 0.012 | 0.003 | **1.5E-05** | 0.011 | 0.003 | **3.7E-05** | 0.0002 |
| Citrate | | | 0.004 | 0.009 | 0.64 | -0.006 | 0.003 | 0.01 | -0.006 | 0.002 | 0.02 | 0.03 |
| Glycerol | | | 0.013 | 0.009 | 0.16 | 0.007 | 0.003 | 0.03 | 0.007 | 0.003 | 0.01 | 0.01 |
| Alanine | | | 0.002 | 0.009 | 0.79 | 0.002 | 0.003 | 0.44 | 0.002 | 0.003 | 0.41 | 0.45 |
| Glutamine | | | -0.005 | 0.009 | 0.61 | -0.003 | 0.003 | 0.30 | -0.003 | 0.003 | 0.26 | 0.30 |
| Glycine | | | -0.021 | 0.008 | 0.01 | 0.006 | 0.003 | 0.04 | 0.003 | 0.003 | 0.26 | 0.30 |
| Histidine | | | -0.005 | 0.009 | 0.55 | -0.001 | 0.003 | 0.84 | -0.001 | 0.003 | 0.70 | 0.73 |
| Isoleucine | | | 0.022 | 0.009 | 0.01 | 0.007 | 0.003 | 0.01 | 0.009 | 0.003 | **0.001** | 0.002 |
| Leucine | | | 0.015 | 0.009 | 0.09 | 0.007 | 0.003 | 0.005 | 0.008 | 0.003 | **0.001** | 0.002 |
| Valine | | | 0.013 | 0.009 | 0.12 | 0.006 | 0.003 | 0.03 | 0.007 | 0.003 | 0.01 | 0.01 |
| Phenylalanine | | | -0.008 | 0.009 | 0.38 | 0.008 | 0.003 | 0.004 | 0.006 | 0.003 | 0.01 | 0.01 |
| Tyrosine | | | 0.006 | 0.009 | 0.47 | 0.007 | 0.003 | 0.01 | 0.007 | 0.003 | 0.01 | 0.01 |
| Acetate | | | 0.008 | 0.009 | 0.40 | 0.005 | 0.003 | 0.06 | 0.006 | 0.003 | 0.04 | 0.049 |
| Acetoacetate | | | -0.002 | 0.009 | 0.86 | 0.009 | 0.003 | **0.002** | 0.008 | 0.003 | 0.00 | 0.000 |
| 3-hydroxybutyrate | | | -0.023 | 0.009 | 0.01 | 0.005 | 0.003 | 0.08 | 0.002 | 0.003 | 0.36 | 0.40 |
| Creatinine | | | 0.005 | 0.009 | 0.55 | -0.006 | 0.003 | 0.02 | -0.005 | 0.002 | 0.03 | 0.04 |
| Albumin | | | 0.001 | 0.009 | 0.94 | 0.000 | 0.003 | 0.89 | 0.000 | 0.003 | 0.87 | 0.88 |
| GlycA (glycoprotein acetylation) | | | 0.009 | 0.008 | 0.26 | 0.011 | 0.003 | **7.2E-05** | 0.011 | 0.003 | **3.7E-05** | 0.0002 |

| **PIBI** | | | | | | | | | | | | |
| --- | --- | --- | --- | --- | --- | --- | --- | --- | --- | --- | --- | --- |
| **Metabolite** | | | **Parogene** | | | **Health 2000** | | | **Meta-analysis** | | | |
|  | | | Beta | SE | p | Beta | SE | p | Beta | SE | p | FDR |
| VLDL | XXL | particles | 0.021 | 0.009 | 0.01 | 0.006 | 0.003 | 0.03 | 0.007 | 0.003 | 0.01 | 0.01 |
|  |  | lipid | -0.011 | 0.009 | 0.22 | -0.001 | 0.003 | 0.83 | -0.001 | 0.003 | 0.57 | 0.61 |
|  |  | phospholipid | -0.016 | 0.008 | 0.05 | -0.009 | 0.003 | **0.001** | -0.009 | 0.002 | **0.0001** | 0.0004 |
|  |  | cholesterol | 0.016 | 0.009 | 0.07 | 0.006 | 0.003 | 0.02 | 0.007 | 0.003 | 0.01 | 0.01 |
|  |  | cholesteryl ester | 0.028 | 0.009 | **0.002** | 0.011 | 0.003 | **3.9E-05** | 0.012 | 0.003 | **1.2E-06** | 6.3E-05 |
|  |  | free cholesterol | 0.025 | 0.009 | 0.004 | 0.011 | 0.003 | **0.0001** | 0.012 | 0.003 | **2.9E-06** | 6.5E-05 |
|  |  | triglycerides | 0.015 | 0.009 | 0.08 | 0.007 | 0.003 | 0.01 | 0.008 | 0.003 | 0.003 | 0.01 |
|  | XL | particles | -0.011 | 0.008 | 0.19 | -0.006 | 0.003 | 0.03 | -0.006 | 0.003 | 0.01 | 0.01 |
|  |  | lipid | -0.012 | 0.008 | 0.14 | -0.006 | 0.003 | 0.02 | -0.007 | 0.003 | 0.01 | 0.01 |
|  |  | phospholipid | 0.003 | 0.008 | 0.69 | -0.001 | 0.003 | 0.73 | -0.001 | 0.003 | 0.83 | 0.85 |
|  |  | cholesterol | 0.016 | 0.009 | 0.07 | 0.006 | 0.003 | 0.03 | 0.007 | 0.003 | 0.01 | 0.01 |
|  |  | cholesteryl ester | 0.015 | 0.009 | 0.08 | 0.007 | 0.003 | 0.02 | 0.007 | 0.003 | 0.01 | 0.01 |
|  |  | free cholesterol | 0.025 | 0.009 | 0.005 | 0.010 | 0.003 | **0.0002** | 0.011 | 0.003 | **1.2E-05** | 0.0001 |
|  |  | triglycerides | 0.025 | 0.009 | 0.005 | 0.009 | 0.003 | **0.001** | 0.010 | 0.003 | **0.0001** | 0.0004 |
|  | L | particles | 0.011 | 0.009 | 0.23 | 0.010 | 0.003 | **0.0001** | 0.011 | 0.003 | **4.6E-05** | 0.0003 |
|  |  | lipid | 0.020 | 0.009 | 0.02 | 0.008 | 0.003 | 0.003 | 0.009 | 0.003 | **0.0005** | 0.0014 |
|  |  | phospholipid | 0.013 | 0.009 | 0.15 | 0.006 | 0.003 | 0.04 | 0.006 | 0.003 | 0.02 | 0.03 |
|  |  | cholesterol | 0.013 | 0.009 | 0.15 | 0.005 | 0.003 | 0.06 | 0.006 | 0.003 | 0.02 | 0.03 |
|  |  | cholesteryl ester | 0.006 | 0.009 | 0.46 | 0.004 | 0.003 | 0.18 | 0.004 | 0.003 | 0.13 | 0.15 |
|  |  | free cholesterol | 0.010 | 0.009 | 0.23 | 0.005 | 0.003 | 0.07 | 0.006 | 0.003 | 0.04 | 0.049 |
|  |  | triglycerides | -0.002 | 0.008 | 0.85 | -0.001 | 0.003 | 0.708 | -0.001 | 0.003 | 0.68 | 0.72 |
|  | M | particles | 0.026 | 0.009 | 0.003 | 0.011 | 0.003 | **0.0001** | 0.012 | 0.003 | **3.5E-06** | 6.9E-05 |
|  |  | lipid | 0.026 | 0.009 | 0.003 | 0.012 | 0.003 | **2.5E-06** | 0.013 | 0.002 | **7.8E-08** | 6.1E-06 |
|  |  | phospholipid | 0.022 | 0.009 | 0.02 | 0.009 | 0.003 | **0.001** | 0.010 | 0.003 | **0.0001** | 0.0004 |
|  |  | cholesterol | -0.016 | 0.009 | 0.07 | -0.008 | 0.003 | 0.003 | -0.009 | 0.003 | **0.001** | 0.002 |
|  |  | cholesteryl ester | 0.000 | 0.009 | 0.96 | 0.005 | 0.003 | 0.06 | 0.005 | 0.003 | 0.07 | 0.08 |
|  |  | free cholesterol | 0.020 | 0.009 | 0.02 | 0.006 | 0.003 | 0.03 | 0.007 | 0.003 | 0.01 | 0.01 |
|  |  | triglycerides | 0.005 | 0.009 | 0.54 | 0.006 | 0.003 | 0.05 | 0.006 | 0.003 | 0.04 | 0.049 |
|  | S | particles | 0.019 | 0.009 | 0.03 | 0.006 | 0.003 | 0.02 | 0.008 | 0.003 | 0.005 | 0.01 |
|  |  | lipid | 0.017 | 0.009 | 0.05 | 0.006 | 0.003 | 0.02 | 0.007 | 0.003 | 0.01 | 0.01 |
|  |  | phospholipid | 0.023 | 0.009 | 0.01 | 0.011 | 0.003 | **0.0001** | 0.012 | 0.003 | **5.2E-06** | 8.7E-05 |
|  |  | cholesterol | 0.019 | 0.009 | 0.04 | 0.009 | 0.003 | **0.001** | 0.010 | 0.003 | **0.0002** | 0.001 |
|  |  | cholesteryl ester | -0.012 | 0.009 | 0.17 | -0.001 | 0.003 | 0.71 | -0.002 | 0.003 | 0.44 | 0.48 |
|  |  | free cholesterol | 0.000 | 0.009 | 0.98 | -0.007 | 0.003 | 0.01 | -0.007 | 0.003 | 0.01 | 0.01 |
|  |  | triglycerides | -0.008 | 0.009 | 0.37 | 0.000 | 0.003 | 0.93 | -0.001 | 0.003 | 0.73 | 0.76 |
|  | XS | particles | -0.008 | 0.009 | 0.38 | -0.010 | 0.003 | **0.0004** | -0.009 | 0.003 | **0.0003** | 0.001 |
|  |  | lipid | -0.009 | 0.009 | 0.30 | -0.009 | 0.003 | **0.001** | -0.009 | 0.003 | **0.0005** | 0.001 |
|  |  | phospholipid | 0.020 | 0.009 | 0.02 | 0.010 | 0.003 | **0.0002** | 0.011 | 0.003 | **2.13E-05** | 0.0002 |
|  |  | cholesterol | -0.012 | 0.009 | 0.21 | 0.002 | 0.003 | 0.57 | 0.000 | 0.003 | 0.87 | 0.88 |
|  |  | cholesteryl ester | -0.011 | 0.008 | 0.18 | 0.007 | 0.003 | 0.01 | 0.005 | 0.003 | 0.04 | 0.049 |
|  |  | free cholesterol | 0.001 | 0.009 | 0.92 | 0.008 | 0.003 | 0.003 | 0.007 | 0.003 | 0.005 | 0.01 |
|  |  | triglycerides | -0.001 | 0.009 | 0.94 | 0.012 | 0.003 | **1.5E-05** | 0.011 | 0.003 | **3.7E-05** | 0.0002 |
| IDL | | particles | 0.004 | 0.009 | 0.64 | -0.006 | 0.003 | 0.01 | -0.006 | 0.002 | 0.02 | 0.03 |
|  |  | lipid | 0.013 | 0.009 | 0.16 | 0.007 | 0.003 | 0.03 | 0.007 | 0.003 | 0.01 | 0.01 |
|  |  | phospholipid | 0.002 | 0.009 | 0.79 | 0.002 | 0.003 | 0.44 | 0.002 | 0.003 | 0.41 | 0.45 |
|  |  | cholesterol | -0.005 | 0.009 | 0.61 | -0.003 | 0.003 | 0.30 | -0.003 | 0.003 | 0.26 | 0.30 |
|  |  | cholesteryl ester | -0.021 | 0.008 | 0.01 | 0.006 | 0.003 | 0.04 | 0.003 | 0.003 | 0.26 | 0.30 |
|  |  | free cholesterol | -0.005 | 0.009 | 0.55 | -0.001 | 0.003 | 0.84 | -0.001 | 0.003 | 0.70 | 0.73 |
|  |  | triglycerides | 0.022 | 0.009 | 0.01 | 0.007 | 0.003 | 0.01 | 0.009 | 0.003 | **0.001** | 0.002 |
| LDL | L | particles | 0.015 | 0.009 | 0.09 | 0.007 | 0.003 | 0.005 | 0.008 | 0.003 | **0.001** | 0.002 |
|  |  | lipid | 0.013 | 0.009 | 0.12 | 0.006 | 0.003 | 0.03 | 0.007 | 0.003 | 0.01 | 0.01 |
|  |  | phospholipid | -0.008 | 0.009 | 0.38 | 0.008 | 0.003 | 0.004 | 0.006 | 0.003 | 0.01 | 0.01 |
|  |  | cholesterol | 0.006 | 0.009 | 0.47 | 0.007 | 0.003 | 0.01 | 0.007 | 0.003 | 0.01 | 0.01 |
|  |  | cholesteryl ester | 0.008 | 0.009 | 0.40 | 0.005 | 0.003 | 0.06 | 0.006 | 0.003 | 0.04 | 0.049 |
|  |  | free cholesterol | -0.002 | 0.009 | 0.86 | 0.009 | 0.003 | **0.002** | 0.008 | 0.003 | 0.00 | 0.000 |
|  |  | triglycerides | -0.023 | 0.009 | 0.01 | 0.005 | 0.003 | 0.08 | 0.002 | 0.003 | 0.36 | 0.40 |
|  | M | particles | 0.005 | 0.009 | 0.55 | -0.006 | 0.003 | 0.02 | -0.005 | 0.002 | 0.03 | 0.04 |
|  |  | lipid | 0.001 | 0.009 | 0.94 | 0.000 | 0.003 | 0.89 | 0.000 | 0.003 | 0.87 | 0.88 |
|  |  | phospholipid | 0.009 | 0.008 | 0.26 | 0.011 | 0.003 | **7.2E-05** | 0.011 | 0.003 | **3.7E-05** | 0.0002 |
|  |  | cholesterol | 0.012 | 0.006 | 0.06 | 0.006 | 0.002 | 0.004 | 0.007 | 0.002 | **0.001** | 0.003 |
|  |  | cholesteryl ester | 0.011 | 0.006 | 0.06 | 0.006 | 0.002 | 0.003 | 0.007 | 0.002 | **0.001** | 0.003 |
|  |  | free cholesterol | 0.012 | 0.006 | 0.06 | 0.005 | 0.002 | 0.01 | 0.006 | 0.002 | **0.002** | 0.004 |
|  |  | triglycerides | 0.008 | 0.006 | 0.20 | 0.008 | 0.002 | **4.1E-05** | 0.008 | 0.002 | **1.7E-05** | 0.0002 |
|  | S | particles | 0.013 | 0.006 | 0.03 | 0.006 | 0.002 | 0.004 | 0.007 | 0.002 | **0.001** | 0.003 |
|  |  | lipid | 0.013 | 0.006 | 0.03 | 0.006 | 0.002 | 0.01 | 0.006 | 0.002 | **0.001** | 0.003 |
|  |  | phospholipid | 0.015 | 0.006 | 0.01 | 0.005 | 0.002 | 0.02 | 0.006 | 0.002 | 0.003 | 0.01 |
|  |  | cholesterol | 0.011 | 0.006 | 0.06 | 0.006 | 0.002 | 0.01 | 0.006 | 0.002 | **0.002** | 0.004 |
|  |  | cholesteryl ester | 0.011 | 0.006 | 0.07 | 0.006 | 0.002 | 0.004 | 0.006 | 0.002 | **0.001** | 0.003 |
|  |  | free cholesterol | 0.012 | 0.006 | 0.05 | 0.004 | 0.002 | 0.04 | 0.005 | 0.002 | 0.01 | 0.01 |
|  |  | triglycerides | 0.014 | 0.006 | 0.02 | 0.007 | 0.002 | **0.001** | 0.007 | 0.002 | **0.0001** | 0.001 |
| HDL | XL | particles | -0.005 | 0.006 | 0.38 | -0.004 | 0.002 | 0.04 | -0.004 | 0.002 | 0.03 | 0.04 |
|  |  | lipid | -0.004 | 0.006 | 0.48 | -0.004 | 0.002 | 0.03 | -0.004 | 0.002 | 0.02 | 0.03 |
|  |  | phospholipid | -0.007 | 0.006 | 0.24 | -0.004 | 0.002 | 0.03 | -0.004 | 0.002 | 0.01 | 0.01 |
|  |  | cholesterol | -0.002 | 0.006 | 0.67 | -0.004 | 0.002 | 0.03 | -0.004 | 0.002 | 0.02 | 0.03 |
|  |  | cholesteryl ester | -0.002 | 0.006 | 0.78 | -0.004 | 0.002 | 0.03 | -0.004 | 0.002 | 0.03 | 0.04 |
|  |  | free cholesterol | -0.005 | 0.006 | 0.37 | -0.004 | 0.002 | 0.02 | -0.004 | 0.002 | 0.01 | 0.01 |
|  |  | triglycerides | 0.004 | 0.006 | 0.48 | 0.003 | 0.002 | 0.16 | 0.003 | 0.002 | 0.12 | 0.14 |
|  | L | particles | -0.010 | 0.006 | 0.08 | -0.004 | 0.002 | 0.05 | -0.004 | 0.002 | 0.01 | 0.01 |
|  |  | lipid | -0.010 | 0.006 | 0.07 | -0.004 | 0.002 | 0.02 | -0.005 | 0.002 | 0.01 | 0.01 |
|  |  | phospholipid | -0.009 | 0.006 | 0.12 | -0.004 | 0.002 | 0.06 | -0.004 | 0.002 | 0.02 | 0.03 |
|  |  | cholesterol | -0.011 | 0.006 | 0.04 | -0.005 | 0.002 | 0.01 | -0.006 | 0.002 | **0.002** | 0.004 |
|  |  | cholesteryl ester | -0.011 | 0.006 | 0.05 | -0.005 | 0.002 | 0.01 | -0.006 | 0.002 | **0.002** | 0.004 |
|  |  | free cholesterol | -0.011 | 0.006 | 0.04 | -0.005 | 0.002 | 0.01 | -0.006 | 0.002 | **0.002** | 0.004 |
|  |  | triglycerides | -0.004 | 0.006 | 0.45 | 0.000 | 0.002 | 0.99 | 0.000 | 0.002 | 0.81 | 0.84 |
|  | M | particles | -0.001 | 0.006 | 0.91 | 0.000 | 0.002 | 0.96 | 0.000 | 0.002 | 0.93 | 0.94 |
|  |  | lipid | -0.001 | 0.006 | 0.84 | 0.000 | 0.002 | 0.84 | -0.001 | 0.002 | 0.80 | 0.84 |
|  |  | phospholipid | 0.000 | 0.006 | 0.94 | 0.000 | 0.002 | 0.95 | 0.000 | 0.002 | 0.93 | 0.94 |
|  |  | cholesterol | -0.004 | 0.006 | 0.48 | -0.001 | 0.002 | 0.53 | -0.002 | 0.002 | 0.41 | 0.46 |
|  |  | cholesteryl ester | -0.005 | 0.006 | 0.45 | -0.001 | 0.002 | 0.47 | -0.002 | 0.002 | 0.35 | 0.39 |
|  |  | free cholesterol | -0.003 | 0.006 | 0.65 | -0.001 | 0.002 | 0.79 | -0.001 | 0.002 | 0.69 | 0.75 |
|  |  | triglycerides | 0.018 | 0.006 | 0.00 | 0.006 | 0.002 | 0.00 | 0.007 | 0.002 | **0.0002** | 0.001 |
|  | S | particles | 0.009 | 0.006 | 0.16 | 0.003 | 0.002 | 0.22 | 0.003 | 0.002 | 0.10 | 0.12 |
|  |  | lipid | 0.008 | 0.006 | 0.19 | 0.002 | 0.002 | 0.31 | 0.003 | 0.002 | 0.17 | 0.20 |
|  |  | phospholipid | 0.005 | 0.006 | 0.37 | 0.001 | 0.002 | 0.79 | 0.001 | 0.002 | 0.58 | 0.64 |
|  |  | cholesterol | 0.006 | 0.006 | 0.34 | 0.003 | 0.002 | 0.23 | 0.003 | 0.002 | 0.15 | 0.17 |
|  |  | cholesteryl ester | 0.006 | 0.006 | 0.33 | 0.003 | 0.002 | 0.18 | 0.003 | 0.002 | 0.11 | 0.13 |
|  |  | free cholesterol | 0.003 | 0.006 | 0.67 | 0.001 | 0.002 | 0.77 | 0.001 | 0.002 | 0.68 | 0.74 |
|  |  | triglycerides | 0.016 | 0.006 | 0.01 | 0.006 | 0.002 | **0.002** | 0.007 | 0.002 | **0.0002** | 0.001 |
| VLDL diameter | | | 0.014 | 0.006 | 0.02 | 0.003 | 0.002 | 0.14 | 0.004 | 0.002 | 0.03 | 0.04 |
| LDL diameter | | | -0.006 | 0.006 | 0.31 | 0.000 | 0.002 | 0.93 | 0.000 | 0.002 | 0.81 | 0.84 |
| HDL diameter | | | -0.012 | 0.006 | 0.04 | -0.005 | 0.002 | 0.01 | -0.006 | 0.002 | **0.002** | 0.004 |
| Cholesterol | serum total | | 0.011 | 0.006 | 0.06 | 0.005 | 0.002 | 0.02 | 0.006 | 0.002 | 0.004 | 0.01 |
|  | VLDL | | 0.018 | 0.006 | 0.003 | 0.008 | 0.002 | **0.0001** | 0.009 | 0.002 | **4.5E-06** | 0.0001 |
|  | remnant | | 0.017 | 0.006 | 0.004 | 0.008 | 0.002 | **0.0001** | 0.009 | 0.002 | **4.3E-06** | 0.0001 |
|  | LDL | | 0.012 | 0.006 | 0.05 | 0.006 | 0.002 | 0.01 | 0.006 | 0.002 | **0.001** | 0.003 |
|  | HDL | | -0.008 | 0.006 | 0.15 | -0.003 | 0.002 | 0.10 | -0.004 | 0.002 | 0.04 | 0.049 |
|  | HDL2 | | -0.009 | 0.006 | 0.11 | -0.004 | 0.002 | 0.07 | -0.004 | 0.002 | 0.02 | 0.03 |
|  | HDL3 | | 0.001 | 0.006 | 0.86 | 0.000 | 0.002 | 0.99 | 0.000 | 0.002 | 0.97 | 0.97 |
|  | esterified | | 0.012 | 0.006 | 0.06 | 0.005 | 0.002 | 0.02 | 0.006 | 0.002 | 0.004 | 0.01 |
|  | free | | 0.011 | 0.006 | 0.06 | 0.005 | 0.002 | 0.01 | 0.006 | 0.002 | **0.002** | 0.004 |
| Triglycerides | serum total | | 0.016 | 0.006 | 0.01 | 0.006 | 0.002 | **0.001** | 0.007 | 0.002 | **0.0001** | 0.001 |
|  | VLDL | | 0.016 | 0.006 | 0.01 | 0.005 | 0.002 | 0.01 | 0.006 | 0.002 | **0.001** | 0.003 |
|  | LDL | | 0.007 | 0.006 | 0.24 | 0.008 | 0.002 | **3.9E-05** | 0.008 | 0.002 | **1.9E-05** | 0.0002 |
|  | HDL | | 0.014 | 0.006 | 0.02 | 0.005 | 0.002 | 0.01 | 0.006 | 0.002 | **0.001** | 0.003 |
| Total phosphoglycerides | | | 0.008 | 0.006 | 0.19 | 0.005 | 0.002 | 0.02 | 0.005 | 0.002 | 0.01 | 0.01 |
| Phosphatidylcholine | | | 0.008 | 0.006 | 0.19 | 0.005 | 0.002 | 0.03 | 0.005 | 0.002 | 0.01 | 0.01 |
| Sphingomyelins | | | 0.005 | 0.006 | 0.40 | 0.004 | 0.002 | 0.06 | 0.004 | 0.002 | 0.04 | 0.049 |
| Total cholines | | | 0.007 | 0.006 | 0.27 | 0.005 | 0.002 | 0.03 | 0.005 | 0.002 | 0.01 | 0.01 |
| Apolipoprotein A-I | | | -0.002 | 0.006 | 0.74 | 0.000 | 0.002 | 0.93 | 0.000 | 0.002 | 0.85 | 0.87 |
| Apolipoprotein B | | | 0.018 | 0.006 | 0.004 | 0.008 | 0.002 | **0.0002** | 0.009 | 0.002 | **7.5E-06** | 0.0002 |
| Apo-B / apoA-I | | | 0.018 | 0.006 | **0.002** | 0.008 | 0.002 | **2.0E-05** | 0.009 | 0.002 | **5.5E-07** | 8.6E-05 |
| Fatty acids | total | | 0.014 | 0.006 | 0.02 | 0.007 | 0.002 | **0.001** | 0.008 | 0.002 | **0.0001** | 0.001 |
|  | unsaturated | | -0.011 | 0.006 | 0.07 | -0.004 | 0.002 | 0.03 | -0.005 | 0.002 | 0.01 | 0.01 |
|  | DHA (docosahexaenoic acid) | | -0.001 | 0.006 | 0.89 | 0.005 | 0.002 | 0.02 | 0.004 | 0.002 | 0.04 | 0.049 |
|  | LA (linoleic acid) | | 0.014 | 0.006 | 0.02 | 0.005 | 0.002 | 0.01 | 0.006 | 0.002 | **0.002** | 0.004 |
|  | omega-3 | | 0.002 | 0.006 | 0.70 | 0.005 | 0.002 | 0.01 | 0.005 | 0.002 | 0.01 | 0.01 |
|  | omega-6 | | 0.013 | 0.006 | 0.04 | 0.006 | 0.002 | 0.01 | 0.006 | 0.002 | **0.001** | 0.003 |
|  | PUFA (polyunsaturated) | | 0.011 | 0.006 | 0.06 | 0.006 | 0.002 | 0.01 | 0.006 | 0.002 | **0.002** | 0.004 |
|  | MUFA (monounsaturated) | | 0.016 | 0.006 | 0.01 | 0.007 | 0.002 | **0.0003** | 0.008 | 0.002 | **2.4E-05** | 0.0003 |
|  | SAFA (saturated) | | 0.012 | 0.006 | 0.05 | 0.007 | 0.002 | **0.002** | 0.007 | 0.002 | **0.0003** | 0.001 |
|  | DHA% | | -0.009 | 0.006 | 0.12 | 0.001 | 0.002 | 0.77 | 0.000 | 0.002 | 0.81 | 0.84 |
|  | LA% | | 0.001 | 0.006 | 0.84 | -0.004 | 0.002 | 0.06 | -0.003 | 0.002 | 0.09 | 0.11 |
|  | omega-3% | | -0.007 | 0.006 | 0.28 | 0.001 | 0.002 | 0.51 | 0.001 | 0.002 | 0.78 | 0.83 |
|  | omega-6% | | -0.004 | 0.006 | 0.48 | -0.005 | 0.002 | 0.01 | -0.005 | 0.002 | 0.01 | 0.01 |
|  | PUFA% | | -0.006 | 0.006 | 0.33 | -0.005 | 0.002 | 0.02 | -0.005 | 0.002 | 0.01 | 0.01 |
|  | MUFA% | | 0.014 | 0.006 | 0.02 | 0.006 | 0.002 | 0.01 | 0.006 | 0.002 | **0.001** | 0.003 |
|  | SAFA% | | -0.008 | 0.006 | 0.19 | 0.000 | 0.002 | 0.97 | -0.001 | 0.002 | 0.70 | 0.75 |
| Glucose | | | -0.006 | 0.006 | 0.32 | 0.004 | 0.002 | 0.04 | 0.003 | 0.002 | 0.10 | 0.12 |
| Lactate | | | 0.001 | 0.006 | 0.83 | 0.005 | 0.002 | 0.013 | 0.005 | 0.002 | 0.01 | 0.01 |
| Pyruvate | | | 0.000 | 0.006 | 0.96 | 0.008 | 0.002 | **6.1E-05** | 0.007 | 0.002 | **0.0001** | 0.001 |
| Citrate | | | 0.003 | 0.006 | 0.62 | -0.007 | 0.002 | **0.0001** | -0.007 | 0.002 | **0.0004** | 0.001 |
| Glycerol | | | 0.008 | 0.006 | 0.20 | 0.003 | 0.002 | 0.17 | 0.004 | 0.002 | 0.08 | 0.10 |
| Alanine | | | 0.003 | 0.006 | 0.64 | 0.002 | 0.002 | 0.38 | 0.002 | 0.002 | 0.33 | 0.37 |
| Glutamine | | | -0.004 | 0.006 | 0.52 | -0.002 | 0.002 | 0.26 | -0.003 | 0.002 | 0.20 | 0.23 |
| Glycine | | | -0.014 | 0.006 | 0.01 | 0.004 | 0.002 | 0.04 | 0.002 | 0.002 | 0.31 | 0.35 |
| Histidine | | | -0.006 | 0.006 | 0.34 | 0.000 | 0.002 | 0.98 | -0.001 | 0.002 | 0.77 | 0.82 |
| Isoleucine | | | 0.013 | 0.006 | 0.03 | 0.005 | 0.002 | 0.02 | 0.006 | 0.002 | 0.003 | 0.01 |
| Leucine | | | 0.008 | 0.006 | 0.17 | 0.005 | 0.002 | 0.01 | 0.006 | 0.002 | 0.003 | 0.01 |
| Valine | | | 0.008 | 0.006 | 0.18 | 0.006 | 0.002 | 0.01 | 0.006 | 0.002 | **0.002** | 0.004 |
| Phenylalanine | | | -0.004 | 0.006 | 0.57 | 0.007 | 0.002 | **0.001** | 0.006 | 0.002 | 0.004 | 0.01 |
| Tyrosine | | | 0.003 | 0.006 | 0.64 | 0.006 | 0.002 | 0.005 | 0.005 | 0.002 | 0.005 | 0.01 |
| Acetate | | | 0.004 | 0.006 | 0.56 | 0.005 | 0.002 | 0.02 | 0.005 | 0.002 | 0.02 | 0.03 |
| Acetoacetate | | | -0.003 | 0.006 | 0.66 | 0.005 | 0.002 | 0.01 | 0.005 | 0.002 | 0.02 | 0.03 |
| 3-hydroxybutyrate | | | -0.014 | 0.006 | 0.02 | 0.003 | 0.002 | 0.18 | 0.001 | 0.002 | 0.58 | 0.64 |
| Creatinine | | | 0.003 | 0.006 | 0.57 | -0.004 | 0.002 | 0.05 | -0.003 | 0.002 | 0.09 | 0.11 |
| Albumin | | | 0.002 | 0.006 | 0.74 | 0.000 | 0.002 | 1.00 | 0.000 | 0.002 | 0.91 | 0.93 |
| GlycA (glycoprotein acetylation) | | | 0.007 | 0.006 | 0.20 | 0.008 | 0.002 | **8.0E-05** | 0.008 | 0.002 | **3.3E-05** | 0.0003 |

**APPENDIX TABLE 3.** **Change in the levels of metabolites (n=157) from the baseline.** The significance derives from paired t-test and is indicated by red color.

| **Metabolite** | | | **Change from baseline (SD)** | **p-value*** |
| --- | --- | --- | --- | --- |
| VLDL | XXL | particles | 0.09 | 6.0E-07 |
|  |  | lipid | 0.09 | 3.9E-07 |
|  |  | phospholipid | 0.09 | 4.2E-07 |
|  |  | cholesterol | 0.10 | 4.7E-08 |
|  |  | cholesteryl ester | 0.10 | 4.5E-09 |
|  |  | free cholesterol | 0.09 | 1.1E-07 |
|  |  | triglyceride | 0.08 | 2.2E-06 |
|  | XL | particles | 0.09 | 1.2E-07 |
|  |  | lipid | 0.09 | 2.7E-08 |
|  |  | phospholipid | 0.09 | 9.7E-08 |
|  |  | cholesterol | 0.09 | 2.4E-07 |
|  |  | cholesteryl ester | 0.09 | 1.5E-07 |
|  |  | free cholesterol | 0.09 | 9.1E-08 |
|  |  | triglyceride | 0.09 | 5.6E-08 |
|  | L | particles | 0.09 | 4.1E-09 |
|  |  | lipid | 0.10 | 4.2E-11 |
|  |  | phospholipid | 0.10 | 2.4E-10 |
|  |  | cholesterol | 0.10 | 9.2E-10 |
|  |  | cholesteryl ester | 0.10 | 1.9E-09 |
|  |  | free cholesterol | 0.10 | 2.4E-09 |
|  |  | triglyceride | 0.10 | 1.9E-10 |
|  | M | particles | 0.10 | 1.2E-10 |
|  |  | lipid | 0.11 | 1.0E-12 |
|  |  | phospholipid | 0.11 | 4.1E-12 |
|  |  | cholesterol | 0.12 | 1.7E-12 |
|  |  | cholesteryl ester | 0.11 | 3.1E-11 |
|  |  | free cholesterol | 0.11 | 1.1E-12 |
|  |  | triglyceride | 0.10 | 7.8E-11 |
|  | S | particles | 0.13 | 8.3E-17 |
|  |  | lipid | 0.14 | 3.0E-18 |
|  |  | phospholipid | 0.14 | 2.7E-18 |
|  |  | cholesterol | 0.14 | 1.1E-13 |
|  |  | cholesteryl ester | 0.12 | 1.2E-10 |
|  |  | free cholesterol | 0.15 | 3.3E-18 |
|  |  | triglyceride | 0.12 | 3.7E-16 |
|  | XS | particles | 0.15 | 3.3E-15 |
|  |  | lipid | 0.15 | 4.1E-14 |
|  |  | phospholipid | 0.12 | 4.9E-10 |
|  |  | cholesterol | 0.13 | 3.9E-10 |
|  |  | cholesteryl ester | 0.12 | 1.2E-09 |
|  |  | free cholesterol | 0.13 | 1.4E-10 |
|  |  | triglyceride | 0.16 | 3.9E-25 |
| IDL |  | particles | 0.10 | 1.2E-06 |
|  |  | lipid | 0.09 | 1.1E-05 |
|  |  | phospholipid | 0.08 | 0.0001 |
|  |  | cholesterol | 0.07 | 0.0004 |
|  |  | cholesteryl ester | 0.08 | 0.0003 |
|  |  | free cholesterol | 0.07 | 0.0010 |
|  |  | triglyceride | 0.19 | 1.2E-28 |
| LDL | L | particles | 0.09 | 2.3E-05 |
|  |  | lipid | 0.08 | 0.0001 |
|  |  | phospholipid | 0.07 | 0.0005 |
|  |  | cholesterol | 0.07 | 0.0008 |
|  |  | cholesteryl ester | 0.07 | 0.0004 |
|  |  | free cholesterol | 0.06 | 0.0033 |
|  |  | triglyceride | 0.18 | 7.8E-24 |
|  | M | particles | 0.09 | 2.1E-05 |
|  |  | lipid | 0.08 | 7.5E-05 |
|  |  | phospholipid | 0.08 | 4.8E-05 |
|  |  | cholesterol | 0.07 | 0.0006 |
|  |  | cholesteryl ester | 0.07 | 0.0005 |
|  |  | free cholesterol | 0.07 | 0.0011 |
|  |  | triglyceride | 0.18 | 4.1E-24 |
|  | S | particles | 0.08 | 4.7E-05 |
|  |  | lipid | 0.08 | 0.0001 |
|  |  | phospholipid | 0.08 | 0.0002 |
|  |  | cholesterol | 0.07 | 0.0012 |
|  |  | cholesteryl ester | 0.07 | 0.0011 |
|  |  | free cholesterol | 0.06 | 0.0022 |
|  |  | triglyceride | 0.18 | 6.7E-22 |
| HDL | XL | particles | 0.00 | 0.7305 |
|  |  | lipid | -0.01 | 0.5703 |
|  |  | phospholipid | -0.02 | 0.0827 |
|  |  | cholesterol | 0.00 | 0.9544 |
|  |  | cholesteryl ester | 0.00 | 0.7284 |
|  |  | free cholesterol | -0.01 | 0.5993 |
|  |  | triglyceride | 0.12 | 2.1E-09 |
|  | L | particles | -0.03 | 0.0466 |
|  |  | lipid | -0.03 | 0.0228 |
|  |  | phospholipid | -0.03 | 0.0592 |
|  |  | cholesterol | -0.04 | 0.0012 |
|  |  | cholesteryl ester | -0.04 | 0.0018 |
|  |  | free cholesterol | -0.04 | 0.0007 |
|  |  | triglyceride | 0.06 | 0.0002 |
|  | M | particles | -0.03 | 0.0830 |
|  |  | lipid | -0.04 | 0.0489 |
|  |  | phospholipid | -0.02 | 0.2420 |
|  |  | cholesterol | -0.06 | 0.0012 |
|  |  | cholesteryl ester | -0.07 | 0.0004 |
|  |  | free cholesterol | -0.04 | 0.0492 |
|  |  | triglyceride | 0.09 | 1.2E-06 |
|  | S | particles | 0.01 | 0.7353 |
|  |  | lipid | 0.00 | 0.9010 |
|  |  | phospholipid | -0.02 | 0.2685 |
|  |  | cholesterol | 0.00 | 0.9848 |
|  |  | cholesteryl ester | 0.00 | 0.9614 |
|  |  | free cholesterol | 0.00 | 0.9395 |
|  |  | triglyceride | 0.13 | 6.6E-16 |
| VLDL diameter | |  | 0.07 | 6.3E-06 |
| LDL diameter |  |  | 0.00 | 0.8455 |
| HDL diameter |  |  | -0.03 | 0.0049 |
| cholesterol | serum total |  | 0.07 | 0.0004 |
|  | VLDL |  | 0.14 | 1.2E-15 |
|  | remnant |  | 0.13 | 2.5E-10 |
|  | LDL |  | 0.07 | 0.0001 |
|  | HDL |  | -0.04 | 0.0094 |
|  | HDL2 |  | -0.05 | 0.0014 |
|  | HDL3 |  | 0.04 | 0.0226 |
|  | esterified |  | 0.07 | 0.0016 |
|  | free |  | 0.10 | 2.6E-06 |
| triglyceride | serum total |  | 0.14 | 2.0E-18 |
|  | VLDL |  | 0.11 | 9.4E-14 |
|  | LDL |  | 0.19 | 2.3E-27 |
|  | HDL |  | 0.15 | 4.2E-15 |
| total phospohoglycerides | |  | 0.09 | 1.5E-05 |
| phosphatidylcholine | |  | 0.09 | 2.5E-05 |
| sphingomyelins | |  | 0.08 | 1.4E-05 |
| total cholines |  |  | 0.08 | 2.2E-05 |
| apolipoprotein A-I | |  | 0.01 | 0.5309 |
| apolipoprotein B | |  | 0.12 | 5.6E-10 |
| apo-B / apoA-I | |  | 0.12 | 1.5E-14 |
| fatty acids | total FA |  | 0.12 | 4.7E-10 |
|  | unsaturated |  | -0.11 | 8.7E-11 |
|  | DHA (docosahexaenoic acid) | | 0.07 | 0.0001 |
|  | LA (linoleic acid) |  | 0.05 | 0.0188 |
|  | omega-3 |  | 0.08 | 1.1E-05 |
|  | omega-6 |  | 0.07 | 0.0009 |
|  | PUFA (polyunsatureted) | | 0.08 | 0.0002 |
|  | MUFA (monounsaturated) | | 0.15 | 2.9E-16 |
|  | SAFA (saturated) | | 0.12 | 6.2E-10 |
|  | DHA% |  | 0.01 | 0.6801 |
|  | LA% |  | -0.15 | 2.8E-19 |
|  | omega-3 % |  | 0.02 | 0.2502 |
|  | omega-6 % |  | -0.16 | 1.8E-22 |
|  | PUFA% |  | -0.15 | 4.0E-19 |
|  | MUFA% |  | 0.16 | 1.3E-24 |
|  | SAFA% |  | 0.06 | 0.0028 |
| glucose |  |  | 0.18 | 9.2E-20 |
| lactate |  |  | 0.13 | 1.5E-12 |
| pyruvate |  |  | 0.15 | 2.8E-16 |
| citrate |  |  | 0.18 | 1.9E-22 |
| glycerol |  |  | 0.20 | 1.7E-20 |
| alanine |  |  | 0.15 | 3.0E-14 |
| glutamine |  |  | 0.08 | 7.5E-05 |
| glycine |  |  | 0.05 | 0.0013 |
| histidine |  |  | -0.08 | 0.0004 |
| isoleucine |  |  | 0.06 | 0.0030 |
| leucine |  |  | 0.02 | 0.3679 |
| valine |  |  | 0.00 | 0.8085 |
| phenylalanine |  |  | 0.16 | 5.0E-15 |
| tyrosine |  |  | 0.13 | 5.6E-11 |
| acetate |  |  | 0.02 | 0.4390 |
| acetoacetate |  |  | 0.07 | 0.0020 |
| 3-hydroxybutyrate | |  | 0.07 | 0.0011 |
| creatinine |  |  | 0.09 | 4.3E-08 |
| albumin |  |  | -0.04 | 0.1222 |
| glycoprotein acetylation | |  | 0.14 | 2.9E-13 |

**APPENDIX TABLE 4.** **Associations of 157 metabolic measures in 11-year follow-up with caries and endodontic parameters in participants with or without advanced tooth loss at baseline in Health-2000 study.** Linear regression adjusted for age, gender, smoking, number of teeth, diabetes, baseline metabolite level, and level of education. Significant p-values (< 0.0028) are indicated by red colour. Significant FDRs (< 0.05) are indicated by purple colour.

| **CARIES** | | | | | | | | | | |
| --- | --- | --- | --- | --- | --- | --- | --- | --- | --- | --- |
|  | |  | **≥ 20 teeth** | | | | **< 20 teeth** | | | |
| **Metabolite** | |  | Beta | SE | p | FDR | Beta | SE | p | FDR |
| VLDL | XXL | particles | 0.014 | 0.018 | 0.44 | 0.53 | -0.050 | 0.026 | 0.06 | 0.11 |
|  |  | lipid | 0.015 | 0.018 | 0.40 | 0.51 | -0.051 | 0.026 | 0.05 | 0.10 |
|  |  | phospholipid | 0.013 | 0.018 | 0.46 | 0.54 | -0.054 | 0.027 | 0.04 | 0.10 |
|  |  | cholesterol | 0.009 | 0.018 | 0.61 | 0.69 | -0.059 | 0.026 | 0.02 | 0.07 |
|  |  | cholesteryl ester | -0.002 | 0.018 | 0.93 | 0.94 | -0.056 | 0.025 | 0.03 | 0.09 |
|  |  | free cholesterol | 0.017 | 0.018 | 0.32 | 0.44 | -0.056 | 0.026 | 0.03 | 0.09 |
|  |  | triglycerides | 0.016 | 0.018 | 0.35 | 0.47 | -0.048 | 0.026 | 0.07 | 0.12 |
|  | XL | particles | 0.023 | 0.017 | 0.18 | 0.28 | -0.064 | 0.025 | 0.01 | 0.04 |
|  |  | lipid | 0.024 | 0.017 | 0.16 | 0.25 | -0.065 | 0.025 | 0.01 | 0.04 |
|  |  | phospholipid | 0.018 | 0.017 | 0.30 | 0.42 | -0.065 | 0.026 | 0.01 | 0.04 |
|  |  | cholesterol | 0.015 | 0.018 | 0.41 | 0.51 | -0.062 | 0.025 | 0.01 | 0.04 |
|  |  | cholesteryl ester | 0.014 | 0.017 | 0.43 | 0.53 | -0.059 | 0.025 | 0.02 | 0.07 |
|  |  | free cholesterol | 0.014 | 0.018 | 0.44 | 0.53 | -0.060 | 0.025 | 0.02 | 0.07 |
|  |  | triglycerides | 0.027 | 0.017 | 0.11 | 0.20 | -0.065 | 0.025 | 0.01 | 0.04 |
|  | L | particles | 0.029 | 0.017 | 0.09 | 0.17 | -0.064 | 0.023 | 0.01 | 0.04 |
|  |  | lipid | 0.029 | 0.016 | 0.08 | 0.16 | -0.064 | 0.023 | 0.01 | 0.04 |
|  |  | phospholipid | 0.027 | 0.017 | 0.11 | 0.20 | -0.066 | 0.024 | 0.01 | 0.04 |
|  |  | cholesterol | 0.022 | 0.017 | 0.20 | 0.29 | -0.064 | 0.024 | 0.01 | 0.04 |
|  |  | cholesteryl ester | 0.016 | 0.017 | 0.33 | 0.44 | -0.060 | 0.023 | 0.01 | 0.04 |
|  |  | free cholesterol | 0.026 | 0.017 | 0.13 | 0.22 | -0.067 | 0.024 | 0.01 | 0.04 |
|  |  | triglycerides | 0.032 | 0.016 | 0.05 | 0.10 | -0.065 | 0.023 | 0.01 | 0.04 |
|  | M | particles | 0.025 | 0.016 | 0.13 | 0.22 | -0.069 | 0.023 | **0.002** | 0.03 |
|  |  | lipid | 0.023 | 0.016 | 0.15 | 0.24 | -0.070 | 0.023 | **0.002** | 0.03 |
|  |  | phospholipid | 0.021 | 0.016 | 0.19 | 0.28 | -0.071 | 0.023 | **0.002** | 0.03 |
|  |  | cholesterol | 0.008 | 0.017 | 0.65 | 0.72 | -0.071 | 0.023 | **0.002** | 0.03 |
|  |  | cholesteryl ester | -0.006 | 0.017 | 0.72 | 0.79 | -0.068 | 0.024 | 0.01 | 0.04 |
|  |  | free cholesterol | 0.022 | 0.016 | 0.17 | 0.26 | -0.073 | 0.023 | **0.002** | 0.03 |
|  |  | triglycerides | 0.030 | 0.016 | 0.05 | 0.10 | -0.070 | 0.022 | **0.002** | 0.03 |
|  | S | particles | 0.005 | 0.016 | 0.75 | 0.81 | -0.067 | 0.023 | 0.004 | 0.04 |
|  |  | lipid | 0.001 | 0.016 | 0.93 | 0.94 | -0.066 | 0.023 | 0.005 | 0.04 |
|  |  | phospholipid | -0.001 | 0.016 | 0.96 | 0.97 | -0.060 | 0.023 | 0.01 | 0.04 |
|  |  | cholesterol | -0.025 | 0.018 | 0.15 | 0.24 | -0.066 | 0.026 | 0.01 | 0.04 |
|  |  | cholesteryl ester | -0.037 | 0.018 | 0.04 | 0.08 | -0.060 | 0.027 | 0.02 | 0.07 |
|  |  | free cholesterol | -0.006 | 0.017 | 0.73 | 0.79 | -0.068 | 0.024 | 0.01 | 0.04 |
|  |  | triglycerides | 0.022 | 0.016 | 0.17 | 0.26 | -0.066 | 0.022 | 0.003 | 0.03 |
|  | XS | particles | -0.039 | 0.018 | 0.03 | 0.07 | -0.059 | 0.028 | 0.03 | 0.09 |
|  |  | lipid | -0.042 | 0.018 | 0.02 | 0.046 | -0.058 | 0.028 | 0.04 | 0.10 |
|  |  | phospholipid | -0.047 | 0.018 | 0.01 | 0.02 | -0.055 | 0.028 | 0.05 | 0.10 |
|  |  | cholesterol | -0.055 | 0.018 | **0.0025** | 0.01 | -0.041 | 0.028 | 0.15 | 0.20 |
|  |  | cholesteryl ester | -0.056 | 0.018 | **0.002** | 0.01 | -0.033 | 0.029 | 0.25 | 0.30 |
|  |  | free cholesterol | -0.051 | 0.018 | 0.005 | 0.02 | -0.052 | 0.028 | 0.06 | 0.11 |
|  |  | triglycerides | 0.002 | 0.016 | 0.91 | 0.94 | -0.066 | 0.024 | 0.01 | 0.04 |
| IDL |  | particles | -0.055 | 0.018 | **0.0026** | 0.01 | -0.050 | 0.029 | 0.08 | 0.14 |
|  |  | lipid | -0.055 | 0.018 | **0.002** | 0.01 | -0.050 | 0.029 | 0.09 | 0.15 |
|  |  | phospholipid | -0.058 | 0.018 | **0.001** | 0.01 | -0.040 | 0.029 | 0.16 | 0.21 |
|  |  | cholesterol | -0.054 | 0.018 | 0.003 | 0.01 | -0.048 | 0.029 | 0.10 | 0.15 |
|  |  | cholesteryl ester | -0.050 | 0.018 | 0.01 | 0.02 | -0.052 | 0.029 | 0.07 | 0.12 |
|  |  | free cholesterol | -0.064 | 0.018 | **0.0004** | 0.01 | -0.035 | 0.029 | 0.23 | 0.28 |
|  |  | triglycerides | -0.017 | 0.016 | 0.29 | 0.41 | -0.045 | 0.026 | 0.09 | 0.15 |
| LDL | L | particles | -0.054 | 0.018 | 0.004 | 0.01 | -0.054 | 0.028 | 0.06 | 0.11 |
|  |  | lipid | -0.051 | 0.018 | 0.005 | 0.02 | -0.055 | 0.029 | 0.06 | 0.11 |
|  |  | phospholipid | -0.052 | 0.018 | 0.004 | 0.01 | -0.052 | 0.029 | 0.07 | 0.12 |
|  |  | cholesterol | -0.053 | 0.018 | 0.004 | 0.01 | -0.055 | 0.029 | 0.06 | 0.11 |
|  |  | cholesteryl ester | -0.051 | 0.018 | 0.01 | 0.02 | -0.059 | 0.029 | 0.04 | 0.10 |
|  |  | free cholesterol | -0.060 | 0.018 | **0.001** | 0.01 | -0.039 | 0.029 | 0.17 | 0.22 |
|  |  | triglycerides | -0.035 | 0.016 | 0.04 | 0.08 | -0.037 | 0.027 | 0.17 | 0.22 |
|  | M | particles | -0.050 | 0.018 | 0.01 | 0.02 | -0.058 | 0.028 | 0.04 | 0.10 |
|  |  | lipid | -0.048 | 0.018 | 0.01 | 0.02 | -0.059 | 0.029 | 0.04 | 0.10 |
|  |  | phospholipid | -0.038 | 0.018 | 0.04 | 0.08 | -0.059 | 0.028 | 0.04 | 0.10 |
|  |  | cholesterol | -0.051 | 0.018 | 0.005 | 0.02 | -0.058 | 0.028 | 0.04 | 0.10 |
|  |  | cholesteryl ester | -0.052 | 0.018 | 0.004 | 0.01 | -0.060 | 0.028 | 0.04 | 0.10 |
|  |  | free cholesterol | -0.050 | 0.018 | 0.01 | 0.02 | -0.050 | 0.028 | 0.07 | 0.12 |
|  |  | triglycerides | -0.037 | 0.016 | 0.02 | 0.046 | -0.033 | 0.028 | 0.22 | 0.27 |
|  | S | particles | -0.050 | 0.018 | 0.01 | 0.02 | -0.058 | 0.028 | 0.04 | 0.10 |
|  |  | lipid | -0.049 | 0.018 | 0.01 | 0.02 | -0.058 | 0.029 | 0.04 | 0.10 |
|  |  | phospholipid | -0.038 | 0.018 | 0.03 | 0.07 | -0.053 | 0.029 | 0.07 | 0.12 |
|  |  | cholesterol | -0.055 | 0.018 | **0.0026** | 0.01 | -0.055 | 0.028 | 0.05 | 0.10 |
|  |  | cholesteryl ester | -0.055 | 0.018 | **0.002** | 0.01 | -0.056 | 0.028 | 0.05 | 0.10 |
|  |  | free cholesterol | -0.053 | 0.018 | 0.003 | 0.01 | -0.048 | 0.028 | 0.09 | 0.15 |
|  |  | triglycerides | -0.014 | 0.017 | 0.42 | 0.52 | -0.060 | 0.027 | 0.03 | 0.09 |
| HDL | XL | particles | -0.038 | 0.012 | **0.002** | 0.01 | 0.030 | 0.019 | 0.11 | 0.16 |
|  |  | lipid | -0.041 | 0.012 | **0.001** | 0.01 | 0.032 | 0.019 | 0.08 | 0.14 |
|  |  | phospholipid | -0.035 | 0.012 | 0.004 | 0.01 | 0.034 | 0.018 | 0.06 | 0.11 |
|  |  | cholesterol | -0.043 | 0.013 | **0.001** | 0.01 | 0.032 | 0.020 | 0.10 | 0.15 |
|  |  | cholesteryl ester | -0.045 | 0.013 | **0.001** | 0.01 | 0.031 | 0.020 | 0.12 | 0.17 |
|  |  | free cholesterol | -0.036 | 0.013 | 0.005 | 0.02 | 0.032 | 0.019 | 0.09 | 0.15 |
|  |  | triglycerides | -0.042 | 0.018 | 0.02 | 0.046 | -0.044 | 0.028 | 0.12 | 0.17 |
|  | L | particles | -0.041 | 0.014 | 0.004 | 0.01 | 0.029 | 0.020 | 0.14 | 0.19 |
|  |  | lipid | -0.043 | 0.014 | **0.002** | 0.01 | 0.033 | 0.020 | 0.09 | 0.15 |
|  |  | phospholipid | -0.047 | 0.014 | **0.001** | 0.01 | 0.033 | 0.021 | 0.11 | 0.16 |
|  |  | cholesterol | -0.035 | 0.013 | 0.01 | 0.02 | 0.032 | 0.019 | 0.10 | 0.15 |
|  |  | cholesteryl ester | -0.035 | 0.013 | 0.01 | 0.02 | 0.032 | 0.019 | 0.10 | 0.15 |
|  |  | free cholesterol | -0.033 | 0.013 | 0.01 | 0.02 | 0.028 | 0.019 | 0.14 | 0.19 |
|  |  | triglycerides | -0.055 | 0.016 | **0.001** | 0.01 | 0.002 | 0.024 | 0.93 | 0.95 |
|  | M | particles | -0.016 | 0.018 | 0.38 | 0.49 | -0.001 | 0.025 | 0.96 | 0.96 |
|  |  | lipid | -0.015 | 0.018 | 0.41 | 0.51 | -0.001 | 0.026 | 0.96 | 0.96 |
|  |  | phospholipid | -0.018 | 0.018 | 0.32 | 0.44 | 0.003 | 0.025 | 0.92 | 0.95 |
|  |  | cholesterol | -0.016 | 0.018 | 0.38 | 0.49 | 0.002 | 0.026 | 0.93 | 0.95 |
|  |  | cholesteryl ester | -0.014 | 0.018 | 0.45 | 0.53 | 0.003 | 0.026 | 0.92 | 0.95 |
|  |  | free cholesterol | -0.026 | 0.018 | 0.14 | 0.23 | 0.003 | 0.025 | 0.90 | 0.94 |
|  |  | triglycerides | 0.016 | 0.018 | 0.36 | 0.47 | -0.040 | 0.025 | 0.10 | 0.15 |
|  | S | particles | 0.011 | 0.018 | 0.56 | 0.64 | -0.010 | 0.028 | 0.72 | 0.79 |
|  |  | lipid | 0.009 | 0.019 | 0.64 | 0.71 | -0.006 | 0.030 | 0.83 | 0.88 |
|  |  | phospholipid | 0.028 | 0.019 | 0.14 | 0.23 | 0.006 | 0.029 | 0.83 | 0.88 |
|  |  | cholesterol | -0.021 | 0.019 | 0.26 | 0.37 | -0.016 | 0.028 | 0.56 | 0.62 |
|  |  | cholesteryl ester | -0.027 | 0.019 | 0.14 | 0.23 | -0.016 | 0.028 | 0.56 | 0.62 |
|  |  | free cholesterol | 0.011 | 0.019 | 0.57 | 0.65 | -0.007 | 0.028 | 0.81 | 0.87 |
|  |  | triglycerides | 0.029 | 0.016 | 0.07 | 0.14 | -0.039 | 0.025 | 0.11 | 0.16 |
| VLDL diameter |  |  | 0.041 | 0.015 | 0.01 | 0.02 | -0.047 | 0.022 | 0.04 | 0.10 |
| LDL diameter |  |  | 0.016 | 0.016 | 0.32 | 0.44 | 0.058 | 0.026 | 0.02 | 0.07 |
| HDL diameter |  |  | -0.035 | 0.012 | 0.003 | 0.01 | 0.029 | 0.018 | 0.11 | 0.16 |
| Cholesterol | serum total |  | -0.059 | 0.018 | **0.001** | 0.01 | -0.057 | 0.029 | 0.05 | 0.10 |
|  | VLDL |  | -0.013 | 0.017 | 0.45 | 0.53 | -0.071 | 0.025 | 0.01 | 0.04 |
|  | remnant |  | -0.033 | 0.018 | 0.06 | 0.12 | -0.072 | 0.028 | 0.01 | 0.04 |
|  | LDL |  | -0.051 | 0.018 | 0.005 | 0.02 | -0.057 | 0.029 | 0.05 | 0.10 |
|  | HDL |  | -0.044 | 0.015 | 0.004 | 0.01 | 0.022 | 0.022 | 0.32 | 0.37 |
|  | HDL2 |  | -0.040 | 0.015 | 0.01 | 0.02 | 0.025 | 0.022 | 0.26 | 0.31 |
|  | HDL3 |  | -0.063 | 0.017 | **0.0002** | 0.01 | -0.005 | 0.025 | 0.84 | 0.89 |
|  | esterified |  | -0.058 | 0.018 | **0.001** | 0.01 | -0.055 | 0.029 | 0.06 | 0.11 |
|  | free |  | -0.063 | 0.018 | **0.0005** | 0.01 | -0.057 | 0.029 | 0.05 | 0.10 |
| Triglycerides | serum total |  | 0.018 | 0.016 | 0.25 | 0.36 | -0.068 | 0.023 | 0.003 | 0.03 |
|  | VLDL |  | 0.026 | 0.016 | 0.09 | 0.17 | -0.066 | 0.023 | 0.003 | 0.03 |
|  | LDL |  | -0.037 | 0.016 | 0.02 | 0.046 | -0.044 | 0.027 | 0.10 | 0.15 |
|  | HDL |  | -0.013 | 0.018 | 0.49 | 0.57 | -0.044 | 0.025 | 0.08 | 0.14 |
| Total phosphoglycerides | | | -0.059 | 0.018 | **0.001** | 0.01 | -0.053 | 0.026 | 0.05 | 0.10 |
| Phosphatidylcholine | | | -0.062 | 0.018 | **0.001** | 0.01 | -0.053 | 0.026 | 0.05 | 0.10 |
| Sphingomyelins | | | -0.074 | 0.017 | **1.3E-05** | 0.002 | -0.020 | 0.027 | 0.46 | 0.52 |
| Total cholines | | | -0.071 | 0.018 | **0.0001** | 0.01 | -0.051 | 0.026 | 0.05 | 0.10 |
| Apolipoprotein A-I | | | -0.054 | 0.017 | **0.002** | 0.01 | -0.018 | 0.026 | 0.49 | 0.55 |
| Apolipoprotein B | | | -0.027 | 0.018 | 0.13 | 0.22 | -0.081 | 0.027 | **0.0026** | 0.03 |
| Apo-B / apoA-I | | | 0.000 | 0.015 | 0.97 | 0.97 | -0.060 | 0.023 | 0.01 | 0.04 |
| Fatty acids | total | | -0.023 | 0.018 | 0.20 | 0.29 | -0.085 | 0.026 | **0.001** | 0.03 |
|  | unsaturated | | -0.043 | 0.017 | 0.01 | 0.02 | -0.018 | 0.028 | 0.51 | 0.57 |
|  | DHA (docosahexaenoic acid) | | -0.062 | 0.017 | **0.0003** | 0.01 | -0.064 | 0.026 | 0.01 | 0.04 |
|  | LA (linoleic acid) | | -0.044 | 0.019 | 0.02 | 0.046 | -0.066 | 0.030 | 0.03 | 0.09 |
|  | omega-3 | | -0.060 | 0.017 | **0.001** | 0.01 | -0.086 | 0.027 | **0.001** | 0.03 |
|  | omega-6 | | -0.057 | 0.018 | **0.002** | 0.01 | -0.082 | 0.028 | 0.004 | 0.04 |
|  | PUFA (polyunsaturated) | | -0.063 | 0.018 | **0.001** | 0.01 | -0.091 | 0.028 | **0.001** | 0.03 |
|  | MUFA (monounsaturated) | | 0.002 | 0.017 | 0.90 | 0.94 | -0.068 | 0.025 | 0.01 | 0.04 |
|  | SAFA (saturated) | | -0.014 | 0.018 | 0.45 | 0.53 | -0.072 | 0.027 | 0.01 | 0.04 |
|  | DHA% | | -0.054 | 0.016 | **0.001** | 0.01 | -0.067 | 0.027 | 0.01 | 0.04 |
|  | LA% | | -0.022 | 0.017 | 0.19 | 0.28 | 0.043 | 0.025 | 0.08 | 0.14 |
|  | omega-3% | | -0.052 | 0.017 | **0.002** | 0.01 | -0.092 | 0.028 | **0.001** | 0.03 |
|  | omega-6% | | -0.028 | 0.017 | 0.09 | 0.17 | 0.052 | 0.024 | 0.03 | 0.09 |
|  | PUFA% | | -0.041 | 0.016 | 0.01 | 0.02 | 0.031 | 0.025 | 0.22 | 0.27 |
|  | MUFA% | | 0.034 | 0.016 | 0.03 | 0.07 | -0.035 | 0.022 | 0.11 | 0.16 |
|  | SAFA% | | 0.028 | 0.018 | 0.13 | 0.22 | 0.011 | 0.029 | 0.70 | 0.77 |
| Glucose | | | 0.035 | 0.018 | 0.05 | 0.10 | 0.037 | 0.029 | 0.20 | 0.26 |
| Lactate | | | 0.018 | 0.017 | 0.30 | 0.42 | 0.025 | 0.029 | 0.38 | 0.44 |
| Pyruvate | | | 0.049 | 0.017 | 0.01 | 0.02 | 0.002 | 0.028 | 0.96 | 0.96 |
| Citrate | | | 0.011 | 0.018 | 0.54 | 0.62 | 0.044 | 0.027 | 0.11 | 0.16 |
| Glycerol | | | 0.067 | 0.021 | **0.001** | 0.01 | 0.039 | 0.036 | 0.28 | 0.33 |
| Alanine | | | -0.027 | 0.018 | 0.13 | 0.22 | -0.041 | 0.027 | 0.13 | 0.18 |
| Glutamine | | | -0.017 | 0.019 | 0.36 | 0.47 | -0.031 | 0.028 | 0.27 | 0.32 |
| Glycine | | | -0.002 | 0.016 | 0.90 | 0.94 | -0.025 | 0.025 | 0.31 | 0.36 |
| Histidine | | | -0.027 | 0.019 | 0.16 | 0.25 | -0.040 | 0.028 | 0.14 | 0.19 |
| Isoleucine | | | 0.009 | 0.018 | 0.63 | 0.71 | -0.063 | 0.025 | 0.01 | 0.04 |
| Leucine | | | -0.002 | 0.018 | 0.93 | 0.94 | -0.067 | 0.025 | 0.01 | 0.04 |
| Valine | | | -0.002 | 0.018 | 0.91 | 0.94 | -0.030 | 0.025 | 0.25 | 0.30 |
| Phenylalanine | | | 0.018 | 0.019 | 0.33 | 0.44 | -0.008 | 0.026 | 0.76 | 0.82 |
| Tyrosine | | | -0.015 | 0.018 | 0.42 | 0.52 | -0.029 | 0.028 | 0.31 | 0.36 |
| Acetate | | | -0.025 | 0.019 | 0.19 | 0.28 | -0.046 | 0.030 | 0.13 | 0.18 |
| Acetoacetate | | | -0.007 | 0.020 | 0.72 | 0.79 | 0.040 | 0.028 | 0.15 | 0.20 |
| 3-hydroxybutyrate | | | -0.003 | 0.020 | 0.88 | 0.93 | 0.039 | 0.027 | 0.15 | 0.20 |
| Creatinine | | | -0.048 | 0.015 | **0.002** | 0.01 | 0.032 | 0.031 | 0.29 | 0.34 |
| Albumin | | | -0.003 | 0.019 | 0.88 | 0.93 | -0.034 | 0.027 | 0.22 | 0.27 |
| GlycA (glycoprotein acetylation) | | | 0.027 | 0.018 | 0.15 | 0.24 | -0.054 | 0.026 | 0.04 | 0.10 |

| **ROOT FILLINGS** | | | | | | | | | | |
| --- | --- | --- | --- | --- | --- | --- | --- | --- | --- | --- |
|  | | | ≥ 20 teeth | | | | < 20 teeth | | | |
| **Metabolite** | | | Beta | SE | p | FDR | Beta | SE | p | FDR |
| VLDL | XXL | particles | 0.021 | 0.010 | 0.03 | 0.14 | -0.006 | 0.021 | 0.78 | 0.89 |
|  |  | lipid | 0.021 | 0.010 | 0.03 | 0.14 | -0.008 | 0.021 | 0.72 | 0.85 |
|  |  | phospholipid | 0.022 | 0.010 | 0.03 | 0.14 | -0.005 | 0.022 | 0.82 | 0.90 |
|  |  | cholesterol | 0.018 | 0.010 | 0.06 | 0.18 | -0.014 | 0.021 | 0.52 | 0.71 |
|  |  | cholesteryl ester | 0.015 | 0.010 | 0.12 | 0.27 | -0.017 | 0.021 | 0.42 | 0.66 |
|  |  | free cholesterol | 0.022 | 0.010 | 0.02 | 0.12 | -0.005 | 0.021 | 0.82 | 0.90 |
|  |  | triglycerides | 0.021 | 0.010 | 0.03 | 0.14 | -0.007 | 0.021 | 0.75 | 0.88 |
|  | XL | particles | 0.020 | 0.009 | 0.03 | 0.14 | -0.013 | 0.020 | 0.52 | 0.71 |
|  |  | lipid | 0.021 | 0.009 | 0.02 | 0.12 | -0.015 | 0.020 | 0.46 | 0.69 |
|  |  | phospholipid | 0.020 | 0.010 | 0.04 | 0.14 | -0.012 | 0.021 | 0.55 | 0.73 |
|  |  | cholesterol | 0.020 | 0.010 | 0.04 | 0.14 | -0.014 | 0.021 | 0.51 | 0.71 |
|  |  | cholesteryl ester | 0.020 | 0.010 | 0.04 | 0.14 | -0.014 | 0.020 | 0.50 | 0.71 |
|  |  | free cholesterol | 0.019 | 0.010 | 0.05 | 0.15 | -0.008 | 0.020 | 0.68 | 0.83 |
|  |  | triglycerides | 0.021 | 0.009 | 0.02 | 0.12 | -0.015 | 0.020 | 0.47 | 0.70 |
|  | L | particles | 0.021 | 0.009 | 0.02 | 0.12 | -0.016 | 0.019 | 0.40 | 0.63 |
|  |  | lipid | 0.022 | 0.009 | 0.01 | 0.12 | -0.021 | 0.019 | 0.27 | 0.51 |
|  |  | phospholipid | 0.022 | 0.009 | 0.02 | 0.12 | -0.018 | 0.019 | 0.36 | 0.59 |
|  |  | cholesterol | 0.021 | 0.009 | 0.02 | 0.12 | -0.019 | 0.019 | 0.32 | 0.55 |
|  |  | cholesteryl ester | 0.019 | 0.009 | 0.04 | 0.14 | -0.021 | 0.019 | 0.25 | 0.51 |
|  |  | free cholesterol | 0.022 | 0.009 | 0.02 | 0.12 | -0.015 | 0.020 | 0.44 | 0.68 |
|  |  | triglycerides | 0.022 | 0.009 | 0.01 | 0.12 | -0.019 | 0.019 | 0.32 | 0.55 |
|  | M | particles | 0.020 | 0.009 | 0.02 | 0.12 | -0.022 | 0.018 | 0.23 | 0.51 |
|  |  | lipid | 0.021 | 0.009 | 0.01 | 0.12 | -0.028 | 0.018 | 0.13 | 0.50 |
|  |  | phospholipid | 0.021 | 0.009 | 0.02 | 0.12 | -0.025 | 0.019 | 0.18 | 0.51 |
|  |  | cholesterol | 0.019 | 0.009 | 0.04 | 0.14 | -0.028 | 0.019 | 0.14 | 0.50 |
|  |  | cholesteryl ester | 0.016 | 0.009 | 0.09 | 0.24 | -0.030 | 0.020 | 0.12 | 0.50 |
|  |  | free cholesterol | 0.021 | 0.009 | 0.02 | 0.12 | -0.024 | 0.019 | 0.20 | 0.51 |
|  |  | triglycerides | 0.021 | 0.009 | 0.01 | 0.12 | -0.025 | 0.018 | 0.18 | 0.51 |
|  | S | particles | 0.018 | 0.009 | 0.05 | 0.15 | -0.035 | 0.019 | 0.06 | 0.50 |
|  |  | lipid | 0.019 | 0.009 | 0.04 | 0.14 | -0.037 | 0.019 | 0.05 | 0.50 |
|  |  | phospholipid | 0.018 | 0.009 | 0.04 | 0.14 | -0.035 | 0.019 | 0.07 | 0.50 |
|  |  | cholesterol | 0.014 | 0.010 | 0.15 | 0.29 | -0.039 | 0.021 | 0.06 | 0.50 |
|  |  | cholesteryl ester | 0.010 | 0.010 | 0.31 | 0.54 | -0.040 | 0.022 | 0.07 | 0.50 |
|  |  | free cholesterol | 0.018 | 0.009 | 0.05 | 0.15 | -0.035 | 0.020 | 0.07 | 0.50 |
|  |  | triglycerides | 0.019 | 0.009 | 0.03 | 0.14 | -0.034 | 0.018 | 0.06 | 0.50 |
|  | XS | particles | 0.007 | 0.010 | 0.46 | 0.72 | -0.042 | 0.022 | 0.06 | 0.50 |
|  |  | lipid | 0.007 | 0.010 | 0.48 | 0.74 | -0.041 | 0.023 | 0.07 | 0.50 |
|  |  | phospholipid | 0.002 | 0.010 | 0.82 | 0.95 | -0.035 | 0.023 | 0.13 | 0.50 |
|  |  | cholesterol | 0.000 | 0.010 | 0.97 | 0.995 | -0.039 | 0.023 | 0.09 | 0.50 |
|  |  | cholesteryl ester | -0.001 | 0.010 | 0.95 | 0.995 | -0.040 | 0.023 | 0.09 | 0.50 |
|  |  | free cholesterol | 0.001 | 0.010 | 0.88 | 0.95 | -0.033 | 0.023 | 0.14 | 0.50 |
|  |  | triglycerides | 0.017 | 0.009 | 0.05 | 0.15 | -0.040 | 0.020 | 0.04 | 0.50 |
| IDL | | particles | -0.002 | 0.010 | 0.85 | 0.95 | -0.030 | 0.023 | 0.20 | 0.51 |
|  |  | lipid | -0.002 | 0.010 | 0.86 | 0.95 | -0.032 | 0.024 | 0.18 | 0.51 |
|  |  | phospholipid | -0.004 | 0.010 | 0.72 | 0.95 | -0.028 | 0.023 | 0.22 | 0.51 |
|  |  | cholesterol | -0.002 | 0.010 | 0.84 | 0.95 | -0.029 | 0.023 | 0.21 | 0.51 |
|  |  | cholesteryl ester | 0.000 | 0.010 | 0.999 | 1.00 | -0.030 | 0.023 | 0.21 | 0.51 |
|  |  | free cholesterol | -0.008 | 0.010 | 0.40 | 0.65 | -0.024 | 0.023 | 0.30 | 0.54 |
|  |  | triglycerides | 0.014 | 0.009 | 0.11 | 0.26 | -0.034 | 0.021 | 0.11 | 0.50 |
| LDL | L | particles | -0.002 | 0.010 | 0.86 | 0.95 | -0.026 | 0.023 | 0.25 | 0.51 |
|  |  | lipid | -0.001 | 0.010 | 0.91 | 0.98 | -0.030 | 0.023 | 0.21 | 0.51 |
|  |  | phospholipid | -0.001 | 0.010 | 0.88 | 0.95 | -0.026 | 0.023 | 0.25 | 0.51 |
|  |  | cholesterol | -0.003 | 0.010 | 0.80 | 0.95 | -0.029 | 0.023 | 0.21 | 0.51 |
|  |  | cholesteryl ester | -0.002 | 0.010 | 0.88 | 0.95 | -0.029 | 0.023 | 0.21 | 0.51 |
|  |  | free cholesterol | -0.007 | 0.010 | 0.50 | 0.76 | -0.024 | 0.023 | 0.30 | 0.54 |
|  |  | triglycerides | 0.007 | 0.009 | 0.41 | 0.66 | -0.024 | 0.022 | 0.27 | 0.51 |
|  | M | particles | 0.000 | 0.010 | 0.97 | 0.995 | -0.029 | 0.023 | 0.21 | 0.51 |
|  |  | lipid | 0.000 | 0.010 | 1.00 | 1.00 | -0.033 | 0.023 | 0.16 | 0.50 |
|  |  | phospholipid | 0.005 | 0.010 | 0.65 | 0.90 | -0.036 | 0.023 | 0.11 | 0.50 |
|  |  | cholesterol | -0.002 | 0.010 | 0.82 | 0.95 | -0.029 | 0.023 | 0.21 | 0.51 |
|  |  | cholesteryl ester | -0.003 | 0.010 | 0.80 | 0.95 | -0.027 | 0.023 | 0.23 | 0.51 |
|  |  | free cholesterol | -0.002 | 0.010 | 0.83 | 0.95 | -0.029 | 0.023 | 0.21 | 0.51 |
|  |  | triglycerides | 0.007 | 0.009 | 0.46 | 0.72 | -0.027 | 0.022 | 0.23 | 0.51 |
|  | S | particles | 0.000 | 0.010 | 0.96 | 0.995 | -0.026 | 0.023 | 0.26 | 0.51 |
|  |  | lipid | 0.000 | 0.010 | 0.98 | 0.999 | -0.028 | 0.023 | 0.23 | 0.51 |
|  |  | phospholipid | 0.005 | 0.010 | 0.60 | 0.88 | -0.025 | 0.023 | 0.27 | 0.51 |
|  |  | cholesterol | -0.003 | 0.010 | 0.73 | 0.95 | -0.025 | 0.023 | 0.27 | 0.51 |
|  |  | cholesteryl ester | -0.004 | 0.010 | 0.70 | 0.95 | -0.025 | 0.023 | 0.27 | 0.51 |
|  |  | free cholesterol | -0.002 | 0.010 | 0.86 | 0.95 | -0.022 | 0.023 | 0.34 | 0.57 |
|  |  | triglycerides | 0.020 | 0.009 | 0.04 | 0.14 | -0.035 | 0.022 | 0.11 | 0.50 |
| HDL | XL | particles | -0.011 | 0.007 | 0.10 | 0.25 | 0.026 | 0.015 | 0.08 | 0.50 |
|  |  | lipid | -0.010 | 0.007 | 0.14 | 0.28 | 0.022 | 0.015 | 0.15 | 0.50 |
|  |  | phospholipid | -0.010 | 0.007 | 0.13 | 0.27 | 0.025 | 0.015 | 0.08 | 0.50 |
|  |  | cholesterol | -0.011 | 0.007 | 0.13 | 0.27 | 0.023 | 0.016 | 0.16 | 0.50 |
|  |  | cholesteryl ester | -0.012 | 0.007 | 0.11 | 0.26 | 0.023 | 0.016 | 0.16 | 0.50 |
|  |  | free cholesterol | -0.011 | 0.007 | 0.13 | 0.27 | 0.025 | 0.015 | 0.10 | 0.50 |
|  |  | triglycerides | -0.004 | 0.010 | 0.72 | 0.95 | -0.004 | 0.023 | 0.88 | 0.91 |
|  | L | particles | -0.012 | 0.008 | 0.13 | 0.27 | 0.036 | 0.016 | 0.03 | 0.50 |
|  |  | lipid | -0.011 | 0.008 | 0.15 | 0.29 | 0.030 | 0.016 | 0.06 | 0.50 |
|  |  | phospholipid | -0.012 | 0.008 | 0.13 | 0.27 | 0.034 | 0.017 | 0.05 | 0.50 |
|  |  | cholesterol | -0.010 | 0.007 | 0.15 | 0.29 | 0.033 | 0.015 | 0.03 | 0.50 |
|  |  | cholesteryl ester | -0.010 | 0.007 | 0.16 | 0.31 | 0.034 | 0.015 | 0.03 | 0.50 |
|  |  | free cholesterol | -0.012 | 0.007 | 0.11 | 0.26 | 0.036 | 0.015 | 0.02 | 0.50 |
|  |  | triglycerides | -0.005 | 0.009 | 0.55 | 0.81 | 0.003 | 0.020 | 0.88 | 0.91 |
|  | M | particles | 0.002 | 0.010 | 0.86 | 0.95 | 0.032 | 0.020 | 0.11 | 0.50 |
|  |  | lipid | -0.001 | 0.010 | 0.93 | 0.99 | 0.032 | 0.021 | 0.13 | 0.50 |
|  |  | phospholipid | 0.000 | 0.010 | 0.998 | 1.00 | 0.031 | 0.020 | 0.13 | 0.50 |
|  |  | cholesterol | -0.004 | 0.010 | 0.71 | 0.95 | 0.036 | 0.021 | 0.09 | 0.50 |
|  |  | cholesteryl ester | -0.003 | 0.010 | 0.75 | 0.95 | 0.037 | 0.021 | 0.07 | 0.50 |
|  |  | free cholesterol | -0.004 | 0.010 | 0.70 | 0.95 | 0.030 | 0.020 | 0.14 | 0.50 |
|  |  | triglycerides | 0.023 | 0.010 | 0.02 | 0.12 | -0.021 | 0.020 | 0.30 | 0.54 |
|  | S | particles | 0.020 | 0.010 | 0.05 | 0.15 | -0.004 | 0.023 | 0.85 | 0.91 |
|  |  | lipid | 0.018 | 0.010 | 0.07 | 0.20 | -0.010 | 0.024 | 0.67 | 0.83 |
|  |  | phospholipid | 0.022 | 0.010 | 0.03 | 0.14 | -0.005 | 0.023 | 0.84 | 0.90 |
|  |  | cholesterol | 0.002 | 0.010 | 0.81 | 0.95 | 0.000 | 0.023 | 0.99 | 0.99 |
|  |  | cholesteryl ester | -0.002 | 0.010 | 0.87 | 0.95 | 0.001 | 0.023 | 0.97 | 0.98 |
|  |  | free cholesterol | 0.018 | 0.010 | 0.09 | 0.24 | 0.005 | 0.023 | 0.81 | 0.90 |
|  |  | triglycerides | 0.028 | 0.009 | **0.002** | 0.10 | -0.045 | 0.020 | 0.02 | 0.50 |
| VLDL diameter | | | 0.020 | 0.009 | 0.02 | 0.12 | -0.021 | 0.018 | 0.24 | 0.51 |
| LDL diameter | | | 0.005 | 0.009 | 0.55 | 0.81 | -0.009 | 0.021 | 0.66 | 0.83 |
| HDL diameter | | | -0.011 | 0.006 | 0.10 | 0.25 | 0.018 | 0.014 | 0.22 | 0.51 |
| Cholesterol | serum total | | -0.004 | 0.010 | 0.66 | 0.91 | -0.019 | 0.024 | 0.43 | 0.67 |
|  | VLDL | | 0.017 | 0.009 | 0.07 | 0.20 | -0.035 | 0.021 | 0.09 | 0.50 |
|  | remnant | | 0.010 | 0.010 | 0.32 | 0.55 | -0.036 | 0.022 | 0.11 | 0.50 |
|  | LDL | | -0.002 | 0.010 | 0.81 | 0.95 | -0.030 | 0.024 | 0.21 | 0.51 |
|  | HDL | | -0.013 | 0.008 | 0.13 | 0.27 | 0.030 | 0.018 | 0.10 | 0.50 |
|  | HDL2 | | -0.013 | 0.008 | 0.12 | 0.27 | 0.031 | 0.018 | 0.09 | 0.50 |
|  | HDL3 | | -0.009 | 0.009 | 0.33 | 0.56 | 0.011 | 0.021 | 0.60 | 0.78 |
|  | esterified | | -0.005 | 0.010 | 0.62 | 0.89 | -0.017 | 0.023 | 0.47 | 0.70 |
|  | free | | -0.003 | 0.010 | 0.76 | 0.95 | -0.020 | 0.023 | 0.39 | 0.62 |
| Triglycerides | serum total | | 0.022 | 0.009 | 0.01 | 0.12 | -0.027 | 0.019 | 0.15 | 0.50 |
|  | VLDL | | 0.021 | 0.009 | 0.01 | 0.12 | -0.028 | 0.018 | 0.13 | 0.50 |
|  | LDL | | 0.008 | 0.009 | 0.35 | 0.59 | -0.023 | 0.022 | 0.30 | 0.54 |
|  | HDL | | 0.016 | 0.010 | 0.10 | 0.25 | -0.018 | 0.021 | 0.39 | 0.62 |
| Total phosphoglycerides | | | 0.003 | 0.010 | 0.79 | 0.95 | 0.009 | 0.021 | 0.67 | 0.83 |
| Phosphatidylcholine | | | 0.001 | 0.010 | 0.93 | 0.99 | 0.004 | 0.022 | 0.84 | 0.90 |
| Sphingomyelins | | | -0.015 | 0.009 | 0.11 | 0.26 | -0.006 | 0.022 | 0.80 | 0.90 |
| Total cholines | | | -0.004 | 0.010 | 0.65 | 0.90 | 0.005 | 0.021 | 0.82 | 0.90 |
| Apolipoprotein A-I | | | -0.011 | 0.009 | 0.23 | 0.42 | 0.023 | 0.021 | 0.27 | 0.51 |
| Apolipoprotein B | | | 0.010 | 0.010 | 0.31 | 0.54 | -0.033 | 0.022 | 0.13 | 0.50 |
| Apo-B / apoA-I | | | 0.011 | 0.008 | 0.20 | 0.37 | -0.044 | 0.019 | 0.02 | 0.50 |
| Fatty acids | total | | 0.021 | 0.010 | 0.04 | 0.14 | -0.009 | 0.022 | 0.69 | 0.83 |
|  | unsaturated | | -0.029 | 0.009 | **0.002** | 0.10 | 0.014 | 0.023 | 0.54 | 0.72 |
|  | DHA (docosahexaenoic acid) | | -0.010 | 0.010 | 0.27 | 0.48 | -0.002 | 0.021 | 0.92 | 0.94 |
|  | LA (linoleic acid) | | 0.006 | 0.010 | 0.54 | 0.81 | -0.013 | 0.024 | 0.59 | 0.77 |
|  | omega-3 | | -0.007 | 0.010 | 0.46 | 0.72 | -0.003 | 0.022 | 0.88 | 0.91 |
|  | omega-6 | | 0.005 | 0.010 | 0.65 | 0.90 | -0.010 | 0.023 | 0.67 | 0.83 |
|  | PUFA (polyunsaturated) | | 0.002 | 0.010 | 0.87 | 0.95 | -0.009 | 0.023 | 0.70 | 0.83 |
|  | MUFA (monounsaturated) | | 0.027 | 0.009 | 0.004 | 0.12 | -0.013 | 0.021 | 0.52 | 0.71 |
|  | SAFA (saturated) | | 0.024 | 0.010 | 0.02 | 0.12 | -0.005 | 0.022 | 0.83 | 0.90 |
|  | DHA% | | -0.018 | 0.009 | 0.04 | 0.14 | 0.011 | 0.022 | 0.62 | 0.79 |
|  | LA% | | -0.019 | 0.009 | 0.04 | 0.14 | -0.013 | 0.020 | 0.50 | 0.71 |
|  | omega-3% | | -0.015 | 0.009 | 0.09 | 0.24 | 0.015 | 0.023 | 0.52 | 0.71 |
|  | omega-6% | | -0.025 | 0.009 | 0.01 | 0.12 | 0.001 | 0.020 | 0.96 | 0.97 |
|  | PUFA% | | -0.027 | 0.009 | **0.002** | 0.10 | 0.003 | 0.020 | 0.87 | 0.91 |
|  | MUFA% | | 0.025 | 0.009 | 0.003 | 0.12 | -0.018 | 0.018 | 0.32 | 0.55 |
|  | SAFA% | | 0.023 | 0.010 | 0.02 | 0.12 | 0.014 | 0.023 | 0.54 | 0.72 |
| Glucose | | | 0.008 | 0.010 | 0.41 | 0.66 | -0.007 | 0.023 | 0.78 | 0.89 |
| Lactate | | | 0.017 | 0.010 | 0.07 | 0.20 | 0.018 | 0.023 | 0.45 | 0.69 |
| Pyruvate | | | 0.018 | 0.010 | 0.05 | 0.15 | 0.020 | 0.023 | 0.39 | 0.62 |
| Citrate | | | -0.005 | 0.010 | 0.64 | 0.90 | 0.028 | 0.022 | 0.21 | 0.51 |
| Glycerol | | | 0.014 | 0.011 | 0.21 | 0.39 | -0.025 | 0.026 | 0.34 | 0.57 |
| Alanine | | | 0.018 | 0.010 | 0.07 | 0.20 | -0.025 | 0.022 | 0.25 | 0.51 |
| Glutamine | | | 0.003 | 0.010 | 0.80 | 0.95 | -0.014 | 0.023 | 0.54 | 0.72 |
| Glycine | | | 0.001 | 0.009 | 0.88 | 0.95 | -0.020 | 0.020 | 0.31 | 0.55 |
| Histidine | | | -0.003 | 0.011 | 0.74 | 0.95 | -0.009 | 0.022 | 0.69 | 0.83 |
| Isoleucine | | | 0.013 | 0.010 | 0.20 | 0.37 | -0.010 | 0.020 | 0.61 | 0.79 |
| Leucine | | | 0.012 | 0.010 | 0.23 | 0.42 | -0.003 | 0.021 | 0.89 | 0.91 |
| Valine | | | 0.005 | 0.010 | 0.61 | 0.89 | -0.014 | 0.021 | 0.50 | 0.71 |
| Phenylalanine | | | 0.009 | 0.010 | 0.38 | 0.63 | 0.014 | 0.021 | 0.49 | 0.71 |
| Tyrosine | | | 0.003 | 0.010 | 0.80 | 0.95 | -0.006 | 0.023 | 0.78 | 0.89 |
| Acetate | | | -0.009 | 0.010 | 0.37 | 0.62 | 0.053 | 0.024 | 0.03 | 0.50 |
| Acetoacetate | | | -0.016 | 0.011 | 0.14 | 0.28 | 0.032 | 0.023 | 0.16 | 0.50 |
| 3-hydroxybutyrate | | | -0.017 | 0.011 | 0.12 | 0.27 | 0.003 | 0.022 | 0.88 | 0.91 |
| Creatinine | | | -0.021 | 0.009 | 0.01 | 0.12 | -0.010 | 0.025 | 0.69 | 0.83 |
| Albumin | | | 0.000 | 0.011 | 0.97 | 0.995 | -0.005 | 0.022 | 0.84 | 0.90 |
| GlycA (glycoprotein acetylation) | | | 0.017 | 0.010 | 0.09 | 0.24 | -0.020 | 0.021 | 0.35 | 0.58 |

| **INADEQUATE ROOT FILLINGS** | | | | | | | | | | |
| --- | --- | --- | --- | --- | --- | --- | --- | --- | --- | --- |
|  | |  | ≥ 20 teeth | | | | < 20 teeth | | | |
| **Metabolite** | |  | Beta | SE | p | FDR | Beta | SE | p | FDR |
| VLDL | XXL | particles | 0.036 | 0.015 | 0.02 | 0.10 | 0.004 | 0.039 | 0.91 | 0.99 |
|  |  | lipid | 0.036 | 0.015 | 0.02 | 0.10 | 0.003 | 0.039 | 0.95 | 0.99 |
|  |  | phospholipid | 0.036 | 0.015 | 0.02 | 0.10 | 0.009 | 0.040 | 0.83 | 0.99 |
|  |  | cholesterol | 0.032 | 0.015 | 0.04 | 0.14 | -0.001 | 0.039 | 0.98 | 0.99 |
|  |  | cholesteryl ester | 0.027 | 0.015 | 0.08 | 0.22 | -0.007 | 0.038 | 0.85 | 0.99 |
|  |  | free cholesterol | 0.038 | 0.015 | 0.01 | 0.10 | 0.014 | 0.039 | 0.72 | 0.99 |
|  |  | triglycerides | 0.036 | 0.015 | 0.02 | 0.10 | 0.002 | 0.039 | 0.95 | 0.99 |
|  | XL | particles | 0.033 | 0.015 | 0.02 | 0.10 | 0.009 | 0.038 | 0.81 | 0.99 |
|  |  | lipid | 0.035 | 0.015 | 0.02 | 0.10 | 0.007 | 0.037 | 0.86 | 0.99 |
|  |  | phospholipid | 0.033 | 0.015 | 0.03 | 0.12 | 0.006 | 0.039 | 0.87 | 0.99 |
|  |  | cholesterol | 0.034 | 0.015 | 0.03 | 0.12 | 0.005 | 0.038 | 0.90 | 0.99 |
|  |  | cholesteryl ester | 0.032 | 0.015 | 0.03 | 0.12 | 0.006 | 0.037 | 0.88 | 0.99 |
|  |  | free cholesterol | 0.033 | 0.015 | 0.03 | 0.12 | 0.010 | 0.037 | 0.80 | 0.99 |
|  |  | triglycerides | 0.035 | 0.015 | 0.02 | 0.10 | 0.009 | 0.037 | 0.81 | 0.99 |
|  | L | particles | 0.033 | 0.014 | 0.02 | 0.10 | 0.007 | 0.035 | 0.85 | 0.99 |
|  |  | lipid | 0.034 | 0.014 | 0.01 | 0.10 | 0.001 | 0.035 | 0.98 | 0.99 |
|  |  | phospholipid | 0.033 | 0.014 | 0.02 | 0.10 | 0.004 | 0.035 | 0.91 | 0.99 |
|  |  | cholesterol | 0.033 | 0.014 | 0.02 | 0.10 | 0.000 | 0.036 | 0.995 | 0.998 |
|  |  | cholesteryl ester | 0.031 | 0.014 | 0.03 | 0.12 | -0.006 | 0.035 | 0.87 | 0.99 |
|  |  | free cholesterol | 0.034 | 0.015 | 0.02 | 0.10 | 0.007 | 0.036 | 0.84 | 0.99 |
|  |  | triglycerides | 0.034 | 0.014 | 0.02 | 0.10 | 0.005 | 0.035 | 0.89 | 0.99 |
|  | M | particles | 0.028 | 0.014 | 0.04 | 0.14 | -0.003 | 0.034 | 0.93 | 0.99 |
|  |  | lipid | 0.029 | 0.014 | 0.03 | 0.12 | -0.013 | 0.034 | 0.71 | 0.99 |
|  |  | phospholipid | 0.028 | 0.014 | 0.04 | 0.14 | -0.009 | 0.034 | 0.80 | 0.99 |
|  |  | cholesterol | 0.026 | 0.014 | 0.07 | 0.20 | -0.021 | 0.035 | 0.56 | 0.93 |
|  |  | cholesteryl ester | 0.022 | 0.015 | 0.14 | 0.27 | -0.032 | 0.036 | 0.37 | 0.81 |
|  |  | free cholesterol | 0.029 | 0.014 | 0.04 | 0.14 | -0.006 | 0.035 | 0.87 | 0.99 |
|  |  | triglycerides | 0.030 | 0.014 | 0.03 | 0.12 | -0.003 | 0.034 | 0.93 | 0.99 |
|  | S | particles | 0.022 | 0.014 | 0.11 | 0.25 | -0.032 | 0.035 | 0.35 | 0.81 |
|  |  | lipid | 0.022 | 0.014 | 0.12 | 0.25 | -0.039 | 0.035 | 0.27 | 0.81 |
|  |  | phospholipid | 0.023 | 0.014 | 0.10 | 0.24 | -0.033 | 0.035 | 0.34 | 0.81 |
|  |  | cholesterol | 0.013 | 0.015 | 0.39 | 0.53 | -0.056 | 0.039 | 0.15 | 0.74 |
|  |  | cholesteryl ester | 0.006 | 0.016 | 0.69 | 0.79 | -0.065 | 0.040 | 0.11 | 0.74 |
|  |  | free cholesterol | 0.021 | 0.014 | 0.14 | 0.27 | -0.035 | 0.036 | 0.33 | 0.81 |
|  |  | triglycerides | 0.027 | 0.014 | 0.05 | 0.17 | -0.022 | 0.033 | 0.52 | 0.88 |
|  | XS | particles | 0.004 | 0.015 | 0.80 | 0.88 | -0.066 | 0.041 | 0.11 | 0.74 |
|  |  | lipid | 0.001 | 0.015 | 0.97 | 0.98 | -0.070 | 0.042 | 0.09 | 0.74 |
|  |  | phospholipid | -0.009 | 0.016 | 0.59 | 0.73 | -0.063 | 0.042 | 0.14 | 0.74 |
|  |  | cholesterol | -0.007 | 0.016 | 0.65 | 0.76 | -0.078 | 0.042 | 0.07 | 0.74 |
|  |  | cholesteryl ester | -0.006 | 0.016 | 0.72 | 0.82 | -0.086 | 0.043 | 0.05 | 0.74 |
|  |  | free cholesterol | -0.010 | 0.016 | 0.51 | 0.65 | -0.056 | 0.042 | 0.18 | 0.74 |
|  |  | triglycerides | 0.023 | 0.014 | 0.09 | 0.24 | -0.036 | 0.036 | 0.32 | 0.81 |
| IDL | | particles | -0.012 | 0.016 | 0.46 | 0.61 | -0.064 | 0.043 | 0.14 | 0.74 |
|  |  | lipid | -0.014 | 0.016 | 0.38 | 0.52 | -0.074 | 0.044 | 0.09 | 0.74 |
|  |  | phospholipid | -0.017 | 0.016 | 0.29 | 0.45 | -0.068 | 0.043 | 0.11 | 0.74 |
|  |  | cholesterol | -0.015 | 0.016 | 0.35 | 0.50 | -0.073 | 0.043 | 0.09 | 0.74 |
|  |  | cholesteryl ester | -0.010 | 0.016 | 0.51 | 0.65 | -0.073 | 0.043 | 0.09 | 0.74 |
|  |  | free cholesterol | -0.025 | 0.016 | 0.11 | 0.25 | -0.062 | 0.043 | 0.15 | 0.74 |
|  |  | triglycerides | 0.016 | 0.014 | 0.26 | 0.42 | -0.041 | 0.039 | 0.29 | 0.81 |
| LDL | L | particles | -0.015 | 0.016 | 0.36 | 0.50 | -0.060 | 0.042 | 0.16 | 0.74 |
|  |  | lipid | -0.016 | 0.016 | 0.31 | 0.46 | -0.072 | 0.043 | 0.10 | 0.74 |
|  |  | phospholipid | -0.015 | 0.016 | 0.35 | 0.50 | -0.067 | 0.043 | 0.12 | 0.74 |
|  |  | cholesterol | -0.018 | 0.016 | 0.24 | 0.41 | -0.072 | 0.043 | 0.10 | 0.74 |
|  |  | cholesteryl ester | -0.017 | 0.016 | 0.29 | 0.45 | -0.070 | 0.043 | 0.10 | 0.74 |
|  |  | free cholesterol | -0.024 | 0.016 | 0.12 | 0.25 | -0.063 | 0.043 | 0.14 | 0.74 |
|  |  | triglycerides | 0.007 | 0.014 | 0.60 | 0.74 | -0.034 | 0.040 | 0.41 | 0.81 |
|  | M | particles | -0.015 | 0.016 | 0.33 | 0.48 | -0.061 | 0.042 | 0.15 | 0.74 |
|  |  | lipid | -0.016 | 0.016 | 0.30 | 0.45 | -0.072 | 0.043 | 0.09 | 0.74 |
|  |  | phospholipid | -0.005 | 0.016 | 0.76 | 0.85 | -0.076 | 0.042 | 0.07 | 0.74 |
|  |  | cholesterol | -0.021 | 0.016 | 0.18 | 0.32 | -0.066 | 0.043 | 0.12 | 0.74 |
|  |  | cholesteryl ester | -0.022 | 0.016 | 0.17 | 0.31 | -0.063 | 0.042 | 0.14 | 0.74 |
|  |  | free cholesterol | -0.018 | 0.016 | 0.25 | 0.41 | -0.066 | 0.042 | 0.12 | 0.74 |
|  |  | triglycerides | 0.007 | 0.014 | 0.64 | 0.76 | -0.039 | 0.041 | 0.35 | 0.81 |
|  | S | particles | -0.015 | 0.016 | 0.36 | 0.50 | -0.056 | 0.042 | 0.18 | 0.74 |
|  |  | lipid | -0.016 | 0.016 | 0.30 | 0.45 | -0.064 | 0.043 | 0.14 | 0.74 |
|  |  | phospholipid | -0.004 | 0.016 | 0.81 | 0.88 | -0.059 | 0.043 | 0.17 | 0.74 |
|  |  | cholesterol | -0.023 | 0.016 | 0.14 | 0.27 | -0.060 | 0.042 | 0.16 | 0.74 |
|  |  | cholesteryl ester | -0.024 | 0.016 | 0.12 | 0.25 | -0.059 | 0.042 | 0.16 | 0.74 |
|  |  | free cholesterol | -0.018 | 0.016 | 0.25 | 0.41 | -0.053 | 0.042 | 0.21 | 0.79 |
|  |  | triglycerides | 0.024 | 0.015 | 0.10 | 0.24 | -0.042 | 0.041 | 0.30 | 0.81 |
| HDL | XL | particles | -0.018 | 0.011 | 0.10 | 0.24 | 0.027 | 0.028 | 0.34 | 0.81 |
|  |  | lipid | -0.015 | 0.011 | 0.17 | 0.31 | 0.020 | 0.028 | 0.49 | 0.86 |
|  |  | phospholipid | -0.016 | 0.010 | 0.13 | 0.27 | 0.018 | 0.027 | 0.50 | 0.86 |
|  |  | cholesterol | -0.017 | 0.011 | 0.13 | 0.27 | 0.025 | 0.030 | 0.39 | 0.81 |
|  |  | cholesteryl ester | -0.019 | 0.011 | 0.10 | 0.24 | 0.026 | 0.030 | 0.39 | 0.81 |
|  |  | free cholesterol | -0.015 | 0.011 | 0.16 | 0.30 | 0.028 | 0.028 | 0.33 | 0.81 |
|  |  | triglycerides | 0.008 | 0.016 | 0.62 | 0.74 | 0.007 | 0.043 | 0.88 | 0.99 |
|  | L | particles | -0.021 | 0.012 | 0.09 | 0.24 | 0.035 | 0.030 | 0.23 | 0.80 |
|  |  | lipid | -0.020 | 0.012 | 0.10 | 0.24 | 0.025 | 0.030 | 0.40 | 0.81 |
|  |  | phospholipid | -0.022 | 0.013 | 0.08 | 0.22 | 0.029 | 0.031 | 0.34 | 0.81 |
|  |  | cholesterol | -0.018 | 0.011 | 0.11 | 0.25 | 0.031 | 0.028 | 0.28 | 0.81 |
|  |  | cholesteryl ester | -0.018 | 0.012 | 0.12 | 0.25 | 0.031 | 0.028 | 0.27 | 0.81 |
|  |  | free cholesterol | -0.021 | 0.011 | 0.06 | 0.18 | 0.038 | 0.028 | 0.17 | 0.74 |
|  |  | triglycerides | -0.006 | 0.014 | 0.68 | 0.79 | -0.001 | 0.036 | 0.98 | 0.99 |
|  | M | particles | 0.003 | 0.016 | 0.84 | 0.90 | 0.033 | 0.038 | 0.38 | 0.81 |
|  |  | lipid | -0.002 | 0.016 | 0.92 | 0.95 | 0.032 | 0.038 | 0.40 | 0.81 |
|  |  | phospholipid | 0.001 | 0.015 | 0.96 | 0.97 | 0.033 | 0.038 | 0.38 | 0.81 |
|  |  | cholesterol | -0.008 | 0.016 | 0.62 | 0.74 | 0.035 | 0.038 | 0.37 | 0.81 |
|  |  | cholesteryl ester | -0.007 | 0.016 | 0.65 | 0.76 | 0.037 | 0.038 | 0.34 | 0.81 |
|  |  | free cholesterol | -0.008 | 0.016 | 0.63 | 0.75 | 0.028 | 0.038 | 0.45 | 0.84 |
|  |  | triglycerides | 0.039 | 0.015 | 0.01 | 0.10 | -0.018 | 0.037 | 0.63 | 0.99 |
|  | S | particles | 0.020 | 0.016 | 0.20 | 0.35 | -0.012 | 0.042 | 0.77 | 0.99 |
|  |  | lipid | 0.017 | 0.016 | 0.29 | 0.45 | -0.023 | 0.045 | 0.60 | 0.98 |
|  |  | phospholipid | 0.032 | 0.016 | 0.04 | 0.14 | -0.008 | 0.043 | 0.85 | 0.99 |
|  |  | cholesterol | -0.016 | 0.016 | 0.33 | 0.48 | -0.018 | 0.042 | 0.67 | 0.99 |
|  |  | cholesteryl ester | -0.023 | 0.016 | 0.14 | 0.27 | -0.020 | 0.042 | 0.63 | 0.99 |
|  |  | free cholesterol | 0.021 | 0.016 | 0.20 | 0.35 | 0.012 | 0.042 | 0.79 | 0.99 |
|  |  | triglycerides | 0.044 | 0.014 | **0.002** | 0.06 | -0.040 | 0.037 | 0.28 | 0.81 |
| VLDL diameter | | | 0.038 | 0.013 | 0.005 | 0.10 | 0.002 | 0.034 | 0.94 | 0.99 |
| LDL diameter | | | 0.025 | 0.013 | 0.07 | 0.20 | -0.041 | 0.039 | 0.29 | 0.81 |
| HDL diameter | | | -0.016 | 0.010 | 0.12 | 0.25 | 0.009 | 0.027 | 0.72 | 0.99 |
| Cholesterol | serum total | | -0.018 | 0.016 | 0.26 | 0.42 | -0.058 | 0.044 | 0.19 | 0.76 |
|  | VLDL | | 0.018 | 0.015 | 0.22 | 0.38 | -0.046 | 0.038 | 0.23 | 0.80 |
|  | remnant | | 0.005 | 0.016 | 0.73 | 0.82 | -0.066 | 0.041 | 0.11 | 0.74 |
|  | LDL | | -0.020 | 0.016 | 0.20 | 0.35 | -0.073 | 0.043 | 0.09 | 0.74 |
|  | HDL | | -0.025 | 0.013 | 0.05 | 0.17 | 0.024 | 0.033 | 0.47 | 0.86 |
|  | HDL2 | | -0.025 | 0.013 | 0.06 | 0.18 | 0.026 | 0.033 | 0.43 | 0.83 |
|  | HDL3 | | -0.026 | 0.014 | 0.07 | 0.20 | 0.000 | 0.038 | 0.998 | 0.998 |
|  | esterified | | -0.019 | 0.016 | 0.22 | 0.38 | -0.055 | 0.043 | 0.21 | 0.79 |
|  | free | | -0.016 | 0.016 | 0.31 | 0.46 | -0.054 | 0.043 | 0.21 | 0.79 |
| Triglycerides | serum total | | 0.032 | 0.014 | 0.02 | 0.10 | -0.011 | 0.035 | 0.76 | 0.99 |
|  | VLDL | | 0.032 | 0.013 | 0.02 | 0.10 | -0.009 | 0.034 | 0.80 | 0.99 |
|  | LDL | | 0.008 | 0.014 | 0.59 | 0.73 | -0.028 | 0.041 | 0.49 | 0.86 |
|  | HDL | | 0.033 | 0.016 | 0.04 | 0.14 | -0.010 | 0.038 | 0.79 | 0.99 |
| Total phosphoglycerides | | | 0.002 | 0.015 | 0.89 | 0.93 | 0.002 | 0.040 | 0.95 | 0.99 |
| Phosphatidylcholine | | | 0.000 | 0.015 | 0.99 | 0.99 | -0.004 | 0.040 | 0.92 | 0.99 |
| Sphingomyelins | | | -0.033 | 0.015 | 0.03 | 0.12 | -0.041 | 0.041 | 0.31 | 0.81 |
| Total cholines | | | -0.010 | 0.015 | 0.49 | 0.64 | -0.005 | 0.040 | 0.89 | 0.99 |
| Apolipoprotein A-I | | | -0.022 | 0.015 | 0.14 | 0.27 | 0.016 | 0.038 | 0.68 | 0.99 |
| Apolipoprotein B | | | 0.005 | 0.015 | 0.77 | 0.86 | -0.057 | 0.040 | 0.16 | 0.74 |
| Apo-B / apoA-I | | | 0.010 | 0.013 | 0.47 | 0.62 | -0.056 | 0.035 | 0.11 | 0.74 |
| Fatty acids | total | | 0.023 | 0.016 | 0.15 | 0.29 | -0.015 | 0.040 | 0.70 | 0.99 |
|  | unsaturated | | -0.057 | 0.015 | **0.0001** | 0.02 | 0.013 | 0.042 | 0.76 | 0.99 |
|  | DHA (docosahexaenoic acid) | | -0.036 | 0.015 | 0.02 | 0.10 | 0.008 | 0.039 | 0.84 | 0.99 |
|  | LA (linoleic acid) | | 0.003 | 0.016 | 0.84 | 0.90 | -0.051 | 0.044 | 0.25 | 0.81 |
|  | omega-3 | | -0.030 | 0.015 | 0.04 | 0.14 | 0.002 | 0.041 | 0.96 | 0.99 |
|  | omega-6 | | -0.002 | 0.016 | 0.89 | 0.93 | -0.042 | 0.042 | 0.32 | 0.81 |
|  | PUFA (polyunsaturated) | | -0.009 | 0.016 | 0.55 | 0.70 | -0.036 | 0.042 | 0.39 | 0.81 |
|  | MUFA (monounsaturated) | | 0.036 | 0.015 | 0.02 | 0.10 | -0.003 | 0.038 | 0.95 | 0.99 |
|  | SAFA (saturated) | | 0.029 | 0.016 | 0.07 | 0.20 | -0.008 | 0.040 | 0.84 | 0.99 |
|  | DHA% | | -0.044 | 0.014 | **0.002** | 0.06 | 0.031 | 0.041 | 0.45 | 0.84 |
|  | LA% | | -0.027 | 0.014 | 0.06 | 0.18 | -0.059 | 0.037 | 0.11 | 0.74 |
|  | omega-3% | | -0.038 | 0.014 | 0.01 | 0.10 | 0.033 | 0.042 | 0.43 | 0.83 |
|  | omega-6% | | -0.038 | 0.014 | 0.01 | 0.10 | -0.034 | 0.037 | 0.36 | 0.81 |
|  | PUFA% | | -0.048 | 0.014 | **0.001** | 0.05 | -0.026 | 0.038 | 0.49 | 0.86 |
|  | MUFA% | | 0.044 | 0.013 | **0.001** | 0.05 | 0.004 | 0.033 | 0.91 | 0.99 |
|  | SAFA% | | 0.038 | 0.016 | 0.02 | 0.10 | 0.027 | 0.043 | 0.54 | 0.90 |
| Glucose | | | -0.004 | 0.015 | 0.80 | 0.88 | -0.016 | 0.043 | 0.72 | 0.99 |
| Lactate | | | 0.015 | 0.015 | 0.33 | 0.48 | 0.045 | 0.043 | 0.30 | 0.81 |
| Pyruvate | | | 0.028 | 0.015 | 0.06 | 0.18 | 0.051 | 0.042 | 0.23 | 0.80 |
| Citrate | | | -0.024 | 0.015 | 0.12 | 0.25 | 0.076 | 0.041 | 0.06 | 0.74 |
| Glycerol | | | 0.001 | 0.018 | 0.96 | 0.97 | -0.036 | 0.049 | 0.46 | 0.85 |
| Alanine | | | 0.018 | 0.016 | 0.26 | 0.42 | -0.001 | 0.041 | 0.97 | 0.99 |
| Glutamine | | | -0.002 | 0.016 | 0.91 | 0.95 | -0.008 | 0.042 | 0.85 | 0.99 |
| Glycine | | | -0.010 | 0.014 | 0.46 | 0.61 | -0.024 | 0.037 | 0.51 | 0.87 |
| Histidine | | | 0.003 | 0.017 | 0.86 | 0.91 | 0.006 | 0.041 | 0.88 | 0.99 |
| Isoleucine | | | 0.011 | 0.016 | 0.48 | 0.63 | 0.010 | 0.038 | 0.79 | 0.99 |
| Leucine | | | 0.008 | 0.016 | 0.62 | 0.74 | 0.030 | 0.038 | 0.44 | 0.84 |
| Valine | | | -0.013 | 0.016 | 0.39 | 0.53 | 0.019 | 0.038 | 0.61 | 0.99 |
| Phenylalanine | | | -0.001 | 0.016 | 0.95 | 0.97 | 0.026 | 0.039 | 0.50 | 0.86 |
| Tyrosine | | | -0.004 | 0.016 | 0.81 | 0.88 | 0.003 | 0.042 | 0.95 | 0.99 |
| Acetate | | | -0.023 | 0.016 | 0.16 | 0.30 | 0.009 | 0.045 | 0.84 | 0.99 |
| Acetoacetate | | | -0.045 | 0.017 | 0.01 | 0.10 | -0.004 | 0.042 | 0.93 | 0.99 |
| 3-hydroxybutyrate | | | -0.030 | 0.017 | 0.08 | 0.22 | -0.035 | 0.040 | 0.39 | 0.81 |
| Creatinine | | | -0.031 | 0.013 | 0.02 | 0.10 | -0.042 | 0.046 | 0.36 | 0.81 |
| Albumin | | | -0.010 | 0.017 | 0.57 | 0.72 | -0.017 | 0.041 | 0.68 | 0.99 |
| GlycA (glycoprotein acetylation) | | | 0.016 | 0.016 | 0.30 | 0.45 | -0.034 | 0.039 | 0.39 | 0.81 |

| **APICAL RAREFACTIONS** | | | | | | | | | | |
| --- | --- | --- | --- | --- | --- | --- | --- | --- | --- | --- |
|  | | | ≥ 20 teeth | | | | < 20 teeth | | | |
| **Metabolite** | |  | Beta | SE | p | FDR | Beta | SE | p | FDR |
| VLDL | XXL | particles | 0.016 | 0.023 | 0.48 | 0.63 | -0.045 | 0.036 | 0.21 | 0.29 |
|  |  | lipid | 0.017 | 0.023 | 0.45 | 0.61 | -0.046 | 0.036 | 0.20 | 0.29 |
|  |  | phospholipid | 0.017 | 0.023 | 0.46 | 0.61 | -0.053 | 0.036 | 0.15 | 0.23 |
|  |  | cholesterol | 0.008 | 0.023 | 0.73 | 0.81 | -0.056 | 0.036 | 0.12 | 0.22 |
|  |  | cholesteryl ester | 0.000 | 0.024 | 0.99 | 0.99 | -0.050 | 0.035 | 0.15 | 0.23 |
|  |  | free cholesterol | 0.022 | 0.023 | 0.35 | 0.56 | -0.052 | 0.036 | 0.15 | 0.23 |
|  |  | triglycerides | 0.018 | 0.023 | 0.44 | 0.61 | -0.044 | 0.036 | 0.22 | 0.31 |
|  | XL | particles | 0.016 | 0.023 | 0.49 | 0.64 | -0.058 | 0.034 | 0.09 | 0.20 |
|  |  | lipid | 0.020 | 0.022 | 0.37 | 0.57 | -0.058 | 0.034 | 0.09 | 0.20 |
|  |  | phospholipid | 0.013 | 0.023 | 0.56 | 0.71 | -0.063 | 0.035 | 0.08 | 0.20 |
|  |  | cholesterol | 0.013 | 0.023 | 0.58 | 0.73 | -0.057 | 0.035 | 0.10 | 0.20 |
|  |  | cholesteryl ester | 0.012 | 0.023 | 0.61 | 0.74 | -0.050 | 0.034 | 0.14 | 0.23 |
|  |  | free cholesterol | 0.011 | 0.023 | 0.64 | 0.76 | -0.055 | 0.034 | 0.10 | 0.20 |
|  |  | triglycerides | 0.021 | 0.022 | 0.34 | 0.56 | -0.057 | 0.034 | 0.09 | 0.20 |
|  | L | particles | 0.020 | 0.022 | 0.36 | 0.57 | -0.060 | 0.032 | 0.06 | 0.20 |
|  |  | lipid | 0.027 | 0.021 | 0.21 | 0.41 | -0.060 | 0.032 | 0.06 | 0.20 |
|  |  | phospholipid | 0.022 | 0.022 | 0.32 | 0.55 | -0.062 | 0.032 | 0.05 | 0.20 |
|  |  | cholesterol | 0.018 | 0.022 | 0.42 | 0.60 | -0.059 | 0.032 | 0.07 | 0.20 |
|  |  | cholesteryl ester | 0.011 | 0.022 | 0.60 | 0.74 | -0.053 | 0.032 | 0.09 | 0.20 |
|  |  | free cholesterol | 0.021 | 0.022 | 0.35 | 0.56 | -0.064 | 0.033 | 0.06 | 0.20 |
|  |  | triglycerides | 0.027 | 0.021 | 0.21 | 0.41 | -0.060 | 0.032 | 0.06 | 0.20 |
|  | M | particles | 0.019 | 0.021 | 0.37 | 0.57 | -0.066 | 0.031 | 0.03 | 0.17 |
|  |  | lipid | 0.025 | 0.021 | 0.23 | 0.42 | -0.068 | 0.031 | 0.03 | 0.17 |
|  |  | phospholipid | 0.020 | 0.021 | 0.34 | 0.56 | -0.069 | 0.031 | 0.03 | 0.17 |
|  |  | cholesterol | 0.013 | 0.022 | 0.56 | 0.71 | -0.070 | 0.032 | 0.03 | 0.17 |
|  |  | cholesteryl ester | 0.002 | 0.023 | 0.91 | 0.93 | -0.066 | 0.033 | 0.05 | 0.20 |
|  |  | free cholesterol | 0.021 | 0.021 | 0.33 | 0.56 | -0.071 | 0.032 | 0.03 | 0.17 |
|  |  | triglycerides | 0.027 | 0.021 | 0.20 | 0.41 | -0.065 | 0.031 | 0.04 | 0.18 |
|  | S | particles | 0.010 | 0.021 | 0.65 | 0.76 | -0.077 | 0.032 | 0.02 | 0.17 |
|  |  | lipid | 0.013 | 0.021 | 0.55 | 0.71 | -0.078 | 0.032 | 0.01 | 0.14 |
|  |  | phospholipid | 0.010 | 0.021 | 0.64 | 0.76 | -0.080 | 0.032 | 0.01 | 0.14 |
|  |  | cholesterol | -0.008 | 0.023 | 0.73 | 0.81 | -0.087 | 0.035 | 0.01 | 0.14 |
|  |  | cholesteryl ester | -0.017 | 0.024 | 0.47 | 0.62 | -0.077 | 0.037 | 0.04 | 0.18 |
|  |  | free cholesterol | 0.008 | 0.022 | 0.72 | 0.81 | -0.089 | 0.033 | 0.01 | 0.14 |
|  |  | triglycerides | 0.021 | 0.020 | 0.32 | 0.55 | -0.067 | 0.030 | 0.03 | 0.17 |
|  | XS | particles | -0.020 | 0.023 | 0.40 | 0.58 | -0.083 | 0.038 | 0.03 | 0.17 |
|  |  | lipid | -0.019 | 0.023 | 0.41 | 0.59 | -0.079 | 0.038 | 0.04 | 0.18 |
|  |  | phospholipid | -0.029 | 0.024 | 0.23 | 0.42 | -0.068 | 0.039 | 0.08 | 0.20 |
|  |  | cholesterol | -0.032 | 0.024 | 0.18 | 0.40 | -0.059 | 0.039 | 0.13 | 0.23 |
|  |  | cholesteryl ester | -0.033 | 0.024 | 0.17 | 0.39 | -0.052 | 0.039 | 0.18 | 0.26 |
|  |  | free cholesterol | -0.031 | 0.024 | 0.20 | 0.41 | -0.070 | 0.038 | 0.07 | 0.20 |
|  |  | triglycerides | 0.018 | 0.021 | 0.39 | 0.58 | -0.082 | 0.033 | 0.01 | 0.14 |
| IDL | | particles | -0.038 | 0.024 | 0.11 | 0.33 | -0.064 | 0.039 | 0.10 | 0.20 |
|  |  | lipid | -0.036 | 0.024 | 0.12 | 0.35 | -0.058 | 0.040 | 0.15 | 0.23 |
|  |  | phospholipid | -0.039 | 0.024 | 0.10 | 0.32 | -0.050 | 0.040 | 0.20 | 0.29 |
|  |  | cholesterol | -0.039 | 0.024 | 0.10 | 0.32 | -0.054 | 0.040 | 0.17 | 0.25 |
|  |  | cholesteryl ester | -0.036 | 0.024 | 0.13 | 0.36 | -0.061 | 0.040 | 0.13 | 0.23 |
|  |  | free cholesterol | -0.047 | 0.024 | 0.05 | 0.28 | -0.040 | 0.039 | 0.32 | 0.40 |
|  |  | triglycerides | 0.016 | 0.021 | 0.45 | 0.61 | -0.063 | 0.036 | 0.08 | 0.20 |
| LDL | L | particles | -0.041 | 0.024 | 0.09 | 0.32 | -0.067 | 0.039 | 0.09 | 0.20 |
|  |  | lipid | -0.037 | 0.024 | 0.12 | 0.35 | -0.061 | 0.040 | 0.12 | 0.22 |
|  |  | phospholipid | -0.040 | 0.024 | 0.10 | 0.32 | -0.063 | 0.039 | 0.11 | 0.21 |
|  |  | cholesterol | -0.040 | 0.024 | 0.09 | 0.32 | -0.059 | 0.040 | 0.13 | 0.23 |
|  |  | cholesteryl ester | -0.039 | 0.024 | 0.10 | 0.32 | -0.065 | 0.039 | 0.10 | 0.20 |
|  |  | free cholesterol | -0.046 | 0.024 | 0.05 | 0.28 | -0.045 | 0.039 | 0.25 | 0.33 |
|  |  | triglycerides | -0.003 | 0.022 | 0.88 | 0.91 | -0.061 | 0.037 | 0.10 | 0.20 |
|  | M | particles | -0.041 | 0.024 | 0.09 | 0.32 | -0.073 | 0.038 | 0.06 | 0.20 |
|  |  | lipid | -0.038 | 0.024 | 0.11 | 0.33 | -0.071 | 0.039 | 0.07 | 0.20 |
|  |  | phospholipid | -0.034 | 0.024 | 0.15 | 0.39 | -0.083 | 0.039 | 0.03 | 0.17 |
|  |  | cholesterol | -0.041 | 0.024 | 0.08 | 0.32 | -0.065 | 0.039 | 0.10 | 0.20 |
|  |  | cholesteryl ester | -0.041 | 0.024 | 0.08 | 0.32 | -0.064 | 0.039 | 0.10 | 0.20 |
|  |  | free cholesterol | -0.045 | 0.024 | 0.06 | 0.29 | -0.068 | 0.039 | 0.08 | 0.20 |
|  |  | triglycerides | -0.011 | 0.022 | 0.61 | 0.74 | -0.064 | 0.038 | 0.09 | 0.20 |
|  | S | particles | -0.043 | 0.024 | 0.07 | 0.31 | -0.076 | 0.038 | 0.05 | 0.20 |
|  |  | lipid | -0.041 | 0.024 | 0.08 | 0.32 | -0.073 | 0.039 | 0.06 | 0.20 |
|  |  | phospholipid | -0.038 | 0.024 | 0.11 | 0.33 | -0.082 | 0.039 | 0.04 | 0.18 |
|  |  | cholesterol | -0.045 | 0.024 | 0.06 | 0.29 | -0.064 | 0.039 | 0.10 | 0.20 |
|  |  | cholesteryl ester | -0.045 | 0.024 | 0.06 | 0.29 | -0.062 | 0.039 | 0.11 | 0.21 |
|  |  | free cholesterol | -0.048 | 0.024 | 0.05 | 0.28 | -0.072 | 0.039 | 0.07 | 0.20 |
|  |  | triglycerides | 0.002 | 0.023 | 0.91 | 0.93 | -0.094 | 0.038 | 0.01 | 0.14 |
| HDL | XL | particles | -0.039 | 0.016 | 0.02 | 0.22 | 0.039 | 0.025 | 0.12 | 0.22 |
|  |  | lipid | -0.043 | 0.016 | 0.01 | 0.14 | 0.036 | 0.026 | 0.16 | 0.25 |
|  |  | phospholipid | -0.034 | 0.016 | 0.03 | 0.26 | 0.042 | 0.025 | 0.08 | 0.20 |
|  |  | cholesterol | -0.048 | 0.017 | 0.005 | 0.14 | 0.034 | 0.027 | 0.21 | 0.29 |
|  |  | cholesteryl ester | -0.051 | 0.017 | 0.004 | 0.14 | 0.031 | 0.028 | 0.27 | 0.36 |
|  |  | free cholesterol | -0.037 | 0.017 | 0.03 | 0.26 | 0.042 | 0.026 | 0.10 | 0.20 |
|  |  | triglycerides | -0.028 | 0.024 | 0.24 | 0.44 | -0.041 | 0.039 | 0.30 | 0.38 |
|  | L | particles | -0.023 | 0.018 | 0.21 | 0.41 | 0.032 | 0.027 | 0.25 | 0.33 |
|  |  | lipid | -0.025 | 0.018 | 0.17 | 0.39 | 0.032 | 0.027 | 0.24 | 0.32 |
|  |  | phospholipid | -0.026 | 0.019 | 0.16 | 0.39 | 0.029 | 0.028 | 0.30 | 0.38 |
|  |  | cholesterol | -0.023 | 0.017 | 0.19 | 0.41 | 0.036 | 0.026 | 0.17 | 0.25 |
|  |  | cholesteryl ester | -0.022 | 0.017 | 0.22 | 0.42 | 0.035 | 0.026 | 0.17 | 0.25 |
|  |  | free cholesterol | -0.025 | 0.017 | 0.15 | 0.39 | 0.037 | 0.026 | 0.15 | 0.23 |
|  |  | triglycerides | -0.011 | 0.021 | 0.60 | 0.74 | 0.000 | 0.033 | 0.995 | 0.995 |
|  | M | particles | -0.002 | 0.024 | 0.94 | 0.95 | -0.031 | 0.034 | 0.37 | 0.45 |
|  |  | lipid | -0.006 | 0.024 | 0.80 | 0.86 | -0.027 | 0.035 | 0.44 | 0.51 |
|  |  | phospholipid | -0.005 | 0.023 | 0.83 | 0.88 | -0.028 | 0.034 | 0.42 | 0.49 |
|  |  | cholesterol | -0.009 | 0.024 | 0.71 | 0.81 | -0.018 | 0.035 | 0.60 | 0.65 |
|  |  | cholesteryl ester | -0.008 | 0.024 | 0.72 | 0.81 | -0.017 | 0.035 | 0.63 | 0.67 |
|  |  | free cholesterol | -0.008 | 0.024 | 0.73 | 0.81 | -0.024 | 0.035 | 0.48 | 0.53 |
|  |  | triglycerides | 0.032 | 0.023 | 0.17 | 0.39 | -0.058 | 0.034 | 0.09 | 0.20 |
|  | S | particles | 0.030 | 0.024 | 0.21 | 0.41 | -0.069 | 0.038 | 0.07 | 0.20 |
|  |  | lipid | 0.027 | 0.024 | 0.27 | 0.48 | -0.064 | 0.041 | 0.12 | 0.22 |
|  |  | phospholipid | 0.046 | 0.024 | 0.06 | 0.29 | -0.047 | 0.039 | 0.24 | 0.32 |
|  |  | cholesterol | -0.010 | 0.024 | 0.67 | 0.78 | -0.055 | 0.039 | 0.15 | 0.23 |
|  |  | cholesteryl ester | -0.020 | 0.024 | 0.40 | 0.58 | -0.046 | 0.038 | 0.23 | 0.32 |
|  |  | free cholesterol | 0.034 | 0.024 | 0.17 | 0.39 | -0.063 | 0.039 | 0.11 | 0.21 |
|  |  | triglycerides | 0.046 | 0.022 | 0.03 | 0.26 | -0.068 | 0.034 | 0.04 | 0.18 |
| VLDL diameter | | | 0.040 | 0.020 | 0.05 | 0.28 | -0.053 | 0.031 | 0.09 | 0.20 |
| LDL diameter | | | 0.027 | 0.020 | 0.19 | 0.41 | 0.083 | 0.035 | 0.02 | 0.17 |
| HDL diameter | | | -0.032 | 0.015 | 0.04 | 0.28 | 0.045 | 0.024 | 0.07 | 0.20 |
| Cholesterol | serum total | | -0.046 | 0.024 | 0.05 | 0.28 | -0.068 | 0.040 | 0.09 | 0.20 |
|  | VLDL | | 0.004 | 0.023 | 0.86 | 0.89 | -0.082 | 0.035 | 0.02 | 0.17 |
|  | remnant | | -0.018 | 0.024 | 0.46 | 0.61 | -0.085 | 0.038 | 0.03 | 0.17 |
|  | LDL | | -0.039 | 0.024 | 0.10 | 0.32 | -0.061 | 0.040 | 0.13 | 0.23 |
|  | HDL | | -0.034 | 0.020 | 0.09 | 0.32 | 0.013 | 0.031 | 0.67 | 0.70 |
|  | HDL2 | | -0.032 | 0.020 | 0.10 | 0.32 | 0.019 | 0.030 | 0.53 | 0.58 |
|  | HDL3 | | -0.046 | 0.022 | 0.04 | 0.28 | -0.046 | 0.035 | 0.18 | 0.26 |
|  | esterified | | -0.047 | 0.024 | 0.05 | 0.28 | -0.067 | 0.040 | 0.09 | 0.20 |
|  | free | | -0.043 | 0.024 | 0.07 | 0.31 | -0.066 | 0.040 | 0.10 | 0.20 |
| Triglycerides | serum total | | 0.029 | 0.021 | 0.16 | 0.39 | -0.071 | 0.032 | 0.02 | 0.17 |
|  | VLDL | | 0.029 | 0.020 | 0.16 | 0.39 | -0.066 | 0.031 | 0.03 | 0.17 |
|  | LDL | | -0.004 | 0.021 | 0.86 | 0.89 | -0.071 | 0.037 | 0.06 | 0.20 |
|  | HDL | | 0.017 | 0.024 | 0.46 | 0.61 | -0.061 | 0.035 | 0.08 | 0.20 |
| Total phosphoglycerides | | | -0.026 | 0.023 | 0.27 | 0.48 | -0.078 | 0.036 | 0.03 | 0.17 |
| Phosphatidylcholine | | | -0.029 | 0.023 | 0.21 | 0.41 | -0.076 | 0.036 | 0.04 | 0.18 |
| Sphingomyelins | | | -0.070 | 0.022 | **0.002** | 0.10 | -0.028 | 0.037 | 0.46 | 0.52 |
| Total cholines | | | -0.047 | 0.023 | 0.04 | 0.28 | -0.070 | 0.036 | 0.05 | 0.20 |
| Apolipoprotein A-I | | | -0.045 | 0.022 | 0.05 | 0.28 | -0.029 | 0.035 | 0.41 | 0.49 |
| Apolipoprotein B | | | -0.020 | 0.023 | 0.39 | 0.58 | -0.092 | 0.037 | 0.01 | 0.14 |
| Apo-B / apoA-I | | | 0.000 | 0.020 | 0.99 | 0.99 | -0.053 | 0.032 | 0.10 | 0.20 |
| Fatty acids | total | | 0.006 | 0.024 | 0.81 | 0.87 | -0.103 | 0.036 | 0.005 | 0.14 |
|  | unsaturated | | -0.070 | 0.022 | **0.002** | 0.10 | 0.054 | 0.038 | 0.15 | 0.23 |
|  | DHA (docosahexaenoic acid) | | -0.062 | 0.023 | 0.01 | 0.14 | -0.009 | 0.036 | 0.80 | 0.82 |
|  | LA (linoleic acid) | | -0.027 | 0.024 | 0.28 | 0.49 | -0.093 | 0.041 | 0.02 | 0.17 |
|  | omega-3 | | -0.054 | 0.023 | 0.02 | 0.22 | -0.026 | 0.037 | 0.48 | 0.53 |
|  | omega-6 | | -0.036 | 0.024 | 0.14 | 0.37 | -0.096 | 0.039 | 0.01 | 0.14 |
|  | PUFA (polyunsaturated) | | -0.043 | 0.024 | 0.07 | 0.31 | -0.086 | 0.038 | 0.02 | 0.17 |
|  | MUFA (monounsaturated) | | 0.030 | 0.023 | 0.18 | 0.40 | -0.091 | 0.035 | 0.01 | 0.14 |
|  | SAFA (saturated) | | 0.019 | 0.024 | 0.43 | 0.61 | -0.096 | 0.037 | 0.01 | 0.14 |
|  | DHA% | | -0.057 | 0.021 | 0.01 | 0.14 | 0.024 | 0.037 | 0.53 | 0.58 |
|  | LA% | | -0.028 | 0.022 | 0.21 | 0.41 | 0.027 | 0.034 | 0.42 | 0.49 |
|  | omega-3% | | -0.048 | 0.022 | 0.03 | 0.26 | 0.012 | 0.039 | 0.76 | 0.79 |
|  | omega-6% | | -0.041 | 0.022 | 0.06 | 0.29 | 0.050 | 0.034 | 0.14 | 0.23 |
|  | PUFA% | | -0.056 | 0.022 | 0.01 | 0.14 | 0.051 | 0.034 | 0.14 | 0.23 |
|  | MUFA% | | 0.055 | 0.020 | 0.01 | 0.14 | -0.039 | 0.030 | 0.19 | 0.28 |
|  | SAFA% | | 0.059 | 0.024 | 0.01 | 0.14 | -0.025 | 0.040 | 0.53 | 0.58 |
| Glucose | | | 0.053 | 0.023 | 0.02 | 0.22 | -0.039 | 0.039 | 0.32 | 0.40 |
| Lactate | | | 0.022 | 0.023 | 0.34 | 0.56 | -0.003 | 0.039 | 0.93 | 0.94 |
| Pyruvate | | | 0.035 | 0.023 | 0.13 | 0.36 | -0.040 | 0.039 | 0.30 | 0.38 |
| Citrate | | | -0.012 | 0.023 | 0.60 | 0.74 | 0.035 | 0.038 | 0.35 | 0.43 |
| Glycerol | | | 0.038 | 0.026 | 0.14 | 0.37 | -0.020 | 0.043 | 0.64 | 0.67 |
| Alanine | | | 0.026 | 0.024 | 0.28 | 0.49 | -0.054 | 0.038 | 0.15 | 0.23 |
| Glutamine | | | 0.007 | 0.025 | 0.76 | 0.83 | 0.003 | 0.039 | 0.94 | 0.95 |
| Glycine | | | 0.006 | 0.021 | 0.79 | 0.86 | -0.029 | 0.034 | 0.40 | 0.48 |
| Histidine | | | -0.008 | 0.025 | 0.74 | 0.82 | -0.040 | 0.038 | 0.29 | 0.38 |
| Isoleucine | | | 0.022 | 0.024 | 0.36 | 0.57 | -0.033 | 0.034 | 0.34 | 0.42 |
| Leucine | | | 0.018 | 0.024 | 0.46 | 0.61 | -0.031 | 0.035 | 0.38 | 0.46 |
| Valine | | | 0.029 | 0.024 | 0.23 | 0.42 | 0.028 | 0.035 | 0.42 | 0.49 |
| Phenylalanine | | | 0.031 | 0.025 | 0.21 | 0.41 | 0.030 | 0.035 | 0.40 | 0.48 |
| Tyrosine | | | 0.037 | 0.024 | 0.13 | 0.36 | 0.029 | 0.038 | 0.45 | 0.52 |
| Acetate | | | 0.005 | 0.025 | 0.84 | 0.89 | -0.011 | 0.041 | 0.79 | 0.81 |
| Acetoacetate | | | -0.012 | 0.026 | 0.65 | 0.76 | -0.019 | 0.038 | 0.61 | 0.66 |
| 3-hydroxybutyrate | | | -0.022 | 0.026 | 0.39 | 0.58 | -0.027 | 0.037 | 0.47 | 0.53 |
| Creatinine | | | -0.075 | 0.020 | **0.0002** | 0.03 | 0.014 | 0.042 | 0.73 | 0.76 |
| Albumin | | | 0.007 | 0.025 | 0.78 | 0.85 | -0.018 | 0.037 | 0.63 | 0.67 |
| GlycA (glycoprotein acetylation) | | | 0.021 | 0.024 | 0.38 | 0.58 | -0.055 | 0.036 | 0.12 | 0.22 |

**APPENDIX TABLE 5.** **Associations of 157 metabolic measures in 11-year follow-up with periodontal parameters in participants with or without advanced tooth loss at baseline in Health-2000 study.** Linear regression adjusted for age, gender, smoking, number of teeth, diabetes, baseline metabolite level, and level of education. Significant p-values (< 0.0028) are indicated by red colour. Significant FDRs (< 0.05) are indicated by purple colour.

| **PPD ≥ 4 mm** | | | | | | | | | | |
| --- | --- | --- | --- | --- | --- | --- | --- | --- | --- | --- |
|  | | | ≥ 20 teeth | | | | < 20 teeth | | | |
| **Metabolite** | | | Beta | SE | p | FDR | Beta | SE | p | FDR |
| VLDL | XXL | particles | -0.002 | 0.003 | 0.56 | 0.80 | 0.001 | 0.010 | 0.93 | 0.95 |
|  |  | lipid | -0.002 | 0.003 | 0.61 | 0.81 | 0.001 | 0.010 | 0.95 | 0.96 |
|  |  | phospholipid | -0.002 | 0.003 | 0.51 | 0.76 | -0.001 | 0.010 | 0.92 | 0.95 |
|  |  | cholesterol | -0.003 | 0.003 | 0.42 | 0.69 | -0.001 | 0.010 | 0.93 | 0.95 |
|  |  | cholesteryl ester | -0.003 | 0.003 | 0.39 | 0.68 | 0.002 | 0.010 | 0.85 | 0.95 |
|  |  | free cholesterol | -0.002 | 0.003 | 0.65 | 0.81 | -0.002 | 0.010 | 0.87 | 0.95 |
|  |  | triglycerides | -0.002 | 0.003 | 0.60 | 0.81 | 0.001 | 0.010 | 0.92 | 0.95 |
|  | XL | particles | -0.003 | 0.003 | 0.37 | 0.68 | -0.007 | 0.010 | 0.48 | 0.95 |
|  |  | lipid | -0.003 | 0.003 | 0.43 | 0.70 | -0.007 | 0.010 | 0.47 | 0.95 |
|  |  | phospholipid | -0.003 | 0.003 | 0.37 | 0.68 | -0.006 | 0.010 | 0.56 | 0.95 |
|  |  | cholesterol | -0.003 | 0.003 | 0.38 | 0.68 | -0.004 | 0.010 | 0.69 | 0.95 |
|  |  | cholesteryl ester | -0.003 | 0.003 | 0.39 | 0.68 | -0.003 | 0.010 | 0.72 | 0.95 |
|  |  | free cholesterol | -0.003 | 0.003 | 0.35 | 0.68 | -0.003 | 0.010 | 0.78 | 0.95 |
|  |  | triglycerides | -0.003 | 0.003 | 0.42 | 0.69 | -0.008 | 0.010 | 0.41 | 0.95 |
|  | L | particles | -0.003 | 0.003 | 0.30 | 0.68 | -0.009 | 0.009 | 0.33 | 0.95 |
|  |  | lipid | -0.002 | 0.003 | 0.50 | 0.76 | -0.010 | 0.009 | 0.27 | 0.92 |
|  |  | phospholipid | -0.003 | 0.003 | 0.34 | 0.68 | -0.009 | 0.009 | 0.32 | 0.95 |
|  |  | cholesterol | -0.003 | 0.003 | 0.37 | 0.68 | -0.008 | 0.009 | 0.41 | 0.95 |
|  |  | cholesteryl ester | -0.003 | 0.003 | 0.38 | 0.68 | -0.007 | 0.009 | 0.47 | 0.95 |
|  |  | free cholesterol | -0.003 | 0.003 | 0.33 | 0.68 | -0.008 | 0.010 | 0.38 | 0.95 |
|  |  | triglycerides | -0.003 | 0.003 | 0.40 | 0.69 | -0.010 | 0.009 | 0.27 | 0.92 |
|  | M | particles | -0.004 | 0.003 | 0.20 | 0.68 | -0.011 | 0.009 | 0.21 | 0.89 |
|  |  | lipid | -0.002 | 0.003 | 0.46 | 0.73 | -0.013 | 0.009 | 0.15 | 0.89 |
|  |  | phospholipid | -0.004 | 0.003 | 0.26 | 0.68 | -0.012 | 0.009 | 0.20 | 0.89 |
|  |  | cholesterol | -0.003 | 0.003 | 0.30 | 0.68 | -0.010 | 0.009 | 0.29 | 0.95 |
|  |  | cholesteryl ester | -0.003 | 0.003 | 0.30 | 0.68 | -0.007 | 0.009 | 0.48 | 0.95 |
|  |  | free cholesterol | -0.004 | 0.003 | 0.26 | 0.68 | -0.011 | 0.009 | 0.21 | 0.89 |
|  |  | triglycerides | -0.003 | 0.003 | 0.34 | 0.68 | -0.013 | 0.009 | 0.14 | 0.89 |
|  | S | particles | -0.004 | 0.003 | 0.17 | 0.68 | -0.012 | 0.009 | 0.17 | 0.89 |
|  |  | lipid | -0.003 | 0.003 | 0.34 | 0.68 | -0.013 | 0.009 | 0.15 | 0.89 |
|  |  | phospholipid | -0.004 | 0.003 | 0.26 | 0.68 | -0.012 | 0.009 | 0.19 | 0.89 |
|  |  | cholesterol | -0.004 | 0.003 | 0.28 | 0.68 | -0.011 | 0.010 | 0.26 | 0.92 |
|  |  | cholesteryl ester | -0.004 | 0.003 | 0.28 | 0.68 | -0.008 | 0.010 | 0.42 | 0.95 |
|  |  | free cholesterol | -0.004 | 0.003 | 0.26 | 0.68 | -0.014 | 0.009 | 0.15 | 0.89 |
|  |  | triglycerides | -0.003 | 0.003 | 0.28 | 0.68 | -0.013 | 0.009 | 0.15 | 0.89 |
|  | XS | particles | -0.003 | 0.003 | 0.32 | 0.68 | -0.005 | 0.011 | 0.66 | 0.95 |
|  |  | lipid | -0.003 | 0.003 | 0.34 | 0.68 | -0.005 | 0.011 | 0.64 | 0.95 |
|  |  | phospholipid | -0.004 | 0.003 | 0.29 | 0.68 | -0.002 | 0.011 | 0.83 | 0.95 |
|  |  | cholesterol | -0.004 | 0.004 | 0.31 | 0.68 | 0.002 | 0.011 | 0.87 | 0.95 |
|  |  | cholesteryl ester | -0.003 | 0.004 | 0.39 | 0.68 | 0.006 | 0.011 | 0.58 | 0.95 |
|  |  | free cholesterol | -0.004 | 0.004 | 0.20 | 0.68 | -0.006 | 0.011 | 0.58 | 0.95 |
|  |  | triglycerides | -0.002 | 0.003 | 0.43 | 0.70 | -0.014 | 0.010 | 0.13 | 0.89 |
| IDL | | particles | -0.004 | 0.004 | 0.30 | 0.68 | 0.004 | 0.011 | 0.72 | 0.95 |
|  |  | lipid | -0.004 | 0.003 | 0.25 | 0.68 | 0.002 | 0.011 | 0.85 | 0.95 |
|  |  | phospholipid | -0.004 | 0.003 | 0.31 | 0.68 | 0.005 | 0.011 | 0.69 | 0.95 |
|  |  | cholesterol | -0.005 | 0.004 | 0.19 | 0.68 | 0.003 | 0.011 | 0.77 | 0.95 |
|  |  | cholesteryl ester | -0.005 | 0.004 | 0.20 | 0.68 | 0.003 | 0.011 | 0.77 | 0.95 |
|  |  | free cholesterol | -0.004 | 0.003 | 0.24 | 0.68 | 0.005 | 0.011 | 0.66 | 0.95 |
|  |  | triglycerides | 0.000 | 0.003 | 0.89 | 0.93 | -0.002 | 0.010 | 0.84 | 0.95 |
| LDL | L | particles | -0.004 | 0.004 | 0.23 | 0.68 | 0.002 | 0.011 | 0.88 | 0.95 |
|  |  | lipid | -0.005 | 0.003 | 0.20 | 0.68 | 0.000 | 0.011 | 0.97 | 0.97 |
|  |  | phospholipid | -0.004 | 0.004 | 0.22 | 0.68 | 0.002 | 0.011 | 0.89 | 0.95 |
|  |  | cholesterol | -0.005 | 0.004 | 0.17 | 0.68 | -0.001 | 0.011 | 0.94 | 0.95 |
|  |  | cholesteryl ester | -0.005 | 0.004 | 0.17 | 0.68 | -0.002 | 0.011 | 0.88 | 0.95 |
|  |  | free cholesterol | -0.004 | 0.004 | 0.22 | 0.68 | 0.004 | 0.011 | 0.74 | 0.95 |
|  |  | triglycerides | -0.001 | 0.003 | 0.87 | 0.93 | 0.006 | 0.011 | 0.57 | 0.95 |
|  | M | particles | -0.005 | 0.004 | 0.17 | 0.68 | -0.002 | 0.011 | 0.85 | 0.95 |
|  |  | lipid | -0.005 | 0.003 | 0.15 | 0.68 | -0.004 | 0.011 | 0.69 | 0.95 |
|  |  | phospholipid | -0.005 | 0.004 | 0.15 | 0.68 | -0.004 | 0.011 | 0.75 | 0.95 |
|  |  | cholesterol | -0.005 | 0.004 | 0.14 | 0.68 | -0.004 | 0.011 | 0.69 | 0.95 |
|  |  | cholesteryl ester | -0.005 | 0.004 | 0.15 | 0.68 | -0.005 | 0.011 | 0.68 | 0.95 |
|  |  | free cholesterol | -0.006 | 0.004 | 0.11 | 0.68 | -0.001 | 0.011 | 0.90 | 0.95 |
|  |  | triglycerides | -0.001 | 0.003 | 0.81 | 0.90 | 0.007 | 0.011 | 0.53 | 0.95 |
|  | S | particles | -0.006 | 0.004 | 0.09 | 0.68 | -0.002 | 0.011 | 0.83 | 0.95 |
|  |  | lipid | -0.006 | 0.003 | 0.08 | 0.68 | -0.004 | 0.011 | 0.74 | 0.95 |
|  |  | phospholipid | -0.007 | 0.003 | 0.05 | 0.68 | -0.002 | 0.011 | 0.89 | 0.95 |
|  |  | cholesterol | -0.006 | 0.004 | 0.10 | 0.68 | -0.004 | 0.011 | 0.75 | 0.95 |
|  |  | cholesteryl ester | -0.005 | 0.004 | 0.12 | 0.68 | -0.004 | 0.011 | 0.74 | 0.95 |
|  |  | free cholesterol | -0.007 | 0.003 | 0.05 | 0.68 | -0.001 | 0.011 | 0.91 | 0.95 |
|  |  | triglycerides | -0.004 | 0.003 | 0.26 | 0.68 | -0.003 | 0.011 | 0.76 | 0.95 |
| HDL | XL | particles | -0.001 | 0.002 | 0.62 | 0.81 | 0.018 | 0.007 | 0.01 | 0.52 |
|  |  | lipid | -0.001 | 0.002 | 0.57 | 0.80 | 0.016 | 0.007 | 0.03 | 0.67 |
|  |  | phospholipid | 0.000 | 0.002 | 0.91 | 0.94 | 0.015 | 0.007 | 0.04 | 0.79 |
|  |  | cholesterol | -0.003 | 0.003 | 0.31 | 0.68 | 0.018 | 0.008 | 0.02 | 0.63 |
|  |  | cholesteryl ester | -0.003 | 0.003 | 0.24 | 0.68 | 0.019 | 0.008 | 0.02 | 0.63 |
|  |  | free cholesterol | -0.001 | 0.002 | 0.57 | 0.80 | 0.017 | 0.007 | 0.03 | 0.67 |
|  |  | triglycerides | -0.005 | 0.003 | 0.12 | 0.68 | 0.012 | 0.011 | 0.27 | 0.92 |
|  | L | particles | 0.000 | 0.003 | 0.97 | 0.97 | 0.015 | 0.008 | 0.06 | 0.89 |
|  |  | lipid | 0.001 | 0.003 | 0.75 | 0.88 | 0.013 | 0.008 | 0.08 | 0.89 |
|  |  | phospholipid | 0.000 | 0.003 | 0.88 | 0.93 | 0.014 | 0.008 | 0.08 | 0.89 |
|  |  | cholesterol | 0.001 | 0.003 | 0.77 | 0.88 | 0.013 | 0.007 | 0.09 | 0.89 |
|  |  | cholesteryl ester | 0.001 | 0.003 | 0.79 | 0.89 | 0.013 | 0.007 | 0.08 | 0.89 |
|  |  | free cholesterol | 0.000 | 0.003 | 0.92 | 0.94 | 0.012 | 0.007 | 0.11 | 0.89 |
|  |  | triglycerides | 0.000 | 0.003 | 0.88 | 0.93 | 0.015 | 0.009 | 0.10 | 0.89 |
|  | M | particles | -0.001 | 0.004 | 0.83 | 0.91 | 0.003 | 0.010 | 0.77 | 0.95 |
|  |  | lipid | -0.001 | 0.004 | 0.76 | 0.88 | 0.002 | 0.010 | 0.82 | 0.95 |
|  |  | phospholipid | 0.000 | 0.003 | 0.96 | 0.97 | 0.005 | 0.010 | 0.64 | 0.95 |
|  |  | cholesterol | -0.002 | 0.004 | 0.55 | 0.80 | 0.001 | 0.010 | 0.91 | 0.95 |
|  |  | cholesteryl ester | -0.002 | 0.004 | 0.51 | 0.76 | 0.001 | 0.010 | 0.94 | 0.95 |
|  |  | free cholesterol | -0.001 | 0.003 | 0.77 | 0.88 | 0.003 | 0.010 | 0.77 | 0.95 |
|  |  | triglycerides | 0.003 | 0.003 | 0.38 | 0.68 | -0.006 | 0.010 | 0.53 | 0.95 |
|  | S | particles | 0.001 | 0.004 | 0.76 | 0.88 | -0.014 | 0.011 | 0.20 | 0.89 |
|  |  | lipid | 0.000 | 0.004 | 0.90 | 0.94 | -0.015 | 0.012 | 0.21 | 0.89 |
|  |  | phospholipid | 0.002 | 0.004 | 0.52 | 0.77 | -0.013 | 0.011 | 0.24 | 0.92 |
|  |  | cholesterol | -0.002 | 0.004 | 0.62 | 0.81 | -0.010 | 0.011 | 0.35 | 0.95 |
|  |  | cholesteryl ester | -0.002 | 0.004 | 0.61 | 0.81 | -0.008 | 0.011 | 0.44 | 0.95 |
|  |  | free cholesterol | 0.000 | 0.004 | 0.94 | 0.95 | -0.012 | 0.011 | 0.27 | 0.92 |
|  |  | triglycerides | 0.001 | 0.003 | 0.81 | 0.90 | -0.010 | 0.010 | 0.29 | 0.95 |
| VLDL diameter | | | 0.003 | 0.003 | 0.35 | 0.68 | -0.013 | 0.009 | 0.16 | 0.89 |
| LDL diameter | | | 0.006 | 0.003 | 0.04 | 0.68 | 0.015 | 0.010 | 0.13 | 0.89 |
| HDL diameter | | | 0.000 | 0.002 | 0.87 | 0.93 | 0.013 | 0.007 | 0.07 | 0.89 |
| Cholesterol | serum total | | -0.006 | 0.003 | 0.08 | 0.68 | 0.002 | 0.011 | 0.89 | 0.95 |
|  | VLDL | | -0.003 | 0.003 | 0.33 | 0.68 | -0.010 | 0.010 | 0.34 | 0.95 |
|  | remnant | | -0.004 | 0.003 | 0.28 | 0.68 | -0.005 | 0.011 | 0.61 | 0.95 |
|  | LDL | | -0.005 | 0.003 | 0.14 | 0.68 | -0.003 | 0.011 | 0.77 | 0.95 |
|  | HDL | | -0.002 | 0.003 | 0.41 | 0.69 | 0.010 | 0.009 | 0.25 | 0.92 |
|  | HDL2 | | -0.002 | 0.003 | 0.53 | 0.78 | 0.010 | 0.009 | 0.24 | 0.92 |
|  | HDL3 | | -0.006 | 0.003 | 0.07 | 0.68 | 0.007 | 0.010 | 0.45 | 0.95 |
|  | esterified | | -0.006 | 0.003 | 0.08 | 0.68 | 0.002 | 0.011 | 0.86 | 0.95 |
|  | free | | -0.006 | 0.003 | 0.10 | 0.68 | 0.002 | 0.011 | 0.85 | 0.95 |
| Triglycerides | serum total | | -0.001 | 0.003 | 0.64 | 0.81 | -0.011 | 0.009 | 0.21 | 0.89 |
|  | VLDL | | -0.002 | 0.003 | 0.59 | 0.81 | -0.013 | 0.009 | 0.15 | 0.89 |
|  | LDL | | -0.001 | 0.003 | 0.74 | 0.88 | 0.004 | 0.011 | 0.68 | 0.95 |
|  | HDL | | 0.001 | 0.004 | 0.85 | 0.93 | 0.003 | 0.010 | 0.80 | 0.95 |
| Total phosphoglycerides | | | -0.004 | 0.003 | 0.28 | 0.68 | 0.003 | 0.010 | 0.74 | 0.95 |
| Phosphatidylcholine | | | -0.005 | 0.003 | 0.16 | 0.68 | 0.003 | 0.010 | 0.76 | 0.95 |
| Sphingomyelins | | | -0.005 | 0.003 | 0.16 | 0.68 | 0.006 | 0.011 | 0.55 | 0.95 |
| Total cholines | | | -0.005 | 0.003 | 0.15 | 0.68 | 0.003 | 0.010 | 0.74 | 0.95 |
| Apolipoprotein A-I | | | -0.006 | 0.003 | 0.06 | 0.68 | 0.008 | 0.010 | 0.41 | 0.95 |
| Apolipoprotein B | | | -0.005 | 0.003 | 0.16 | 0.68 | -0.008 | 0.011 | 0.43 | 0.95 |
| Apo-B / apoA-I | | | -0.004 | 0.003 | 0.13 | 0.68 | -0.008 | 0.009 | 0.36 | 0.95 |
| Fatty acids | total | | -0.002 | 0.004 | 0.64 | 0.81 | -0.007 | 0.010 | 0.52 | 0.95 |
|  | unsaturated | | -0.005 | 0.003 | 0.14 | 0.68 | -0.003 | 0.011 | 0.75 | 0.95 |
|  | DHA (docosahexaenoic acid) | | -0.001 | 0.003 | 0.71 | 0.86 | 0.001 | 0.010 | 0.90 | 0.95 |
|  | LA (linoleic acid) | | -0.005 | 0.004 | 0.14 | 0.68 | -0.003 | 0.012 | 0.82 | 0.95 |
|  | omega-3 | | -0.003 | 0.003 | 0.32 | 0.68 | -0.007 | 0.011 | 0.50 | 0.95 |
|  | omega-6 | | -0.006 | 0.004 | 0.12 | 0.68 | -0.003 | 0.011 | 0.82 | 0.95 |
|  | PUFA (polyunsaturated) | | -0.005 | 0.004 | 0.14 | 0.68 | -0.004 | 0.011 | 0.70 | 0.95 |
|  | MUFA (monounsaturated) | | 0.000 | 0.003 | 0.93 | 0.95 | -0.009 | 0.010 | 0.37 | 0.95 |
|  | SAFA (saturated) | | -0.001 | 0.004 | 0.77 | 0.88 | -0.004 | 0.010 | 0.72 | 0.95 |
|  | DHA% | | -0.002 | 0.003 | 0.60 | 0.81 | 0.001 | 0.011 | 0.90 | 0.95 |
|  | LA% | | -0.002 | 0.003 | 0.50 | 0.76 | 0.004 | 0.010 | 0.69 | 0.95 |
|  | omega-3% | | -0.004 | 0.003 | 0.17 | 0.68 | -0.006 | 0.011 | 0.59 | 0.95 |
|  | omega-6% | | -0.002 | 0.003 | 0.57 | 0.80 | 0.006 | 0.010 | 0.54 | 0.95 |
|  | PUFA% | | -0.002 | 0.003 | 0.45 | 0.72 | 0.004 | 0.010 | 0.67 | 0.95 |
|  | MUFA% | | 0.004 | 0.003 | 0.18 | 0.68 | -0.011 | 0.009 | 0.21 | 0.89 |
|  | SAFA% | | 0.001 | 0.004 | 0.68 | 0.83 | 0.009 | 0.011 | 0.40 | 0.95 |
| Glucose | | | 0.003 | 0.003 | 0.36 | 0.68 | -0.012 | 0.011 | 0.27 | 0.92 |
| Lactate | | | 0.002 | 0.003 | 0.62 | 0.81 | 0.004 | 0.011 | 0.73 | 0.95 |
| Pyruvate | | | 0.004 | 0.003 | 0.19 | 0.68 | -0.002 | 0.011 | 0.89 | 0.95 |
| Citrate | | | -0.002 | 0.003 | 0.49 | 0.76 | 0.006 | 0.011 | 0.59 | 0.95 |
| Glycerol | | | 0.002 | 0.004 | 0.64 | 0.81 | 0.006 | 0.013 | 0.67 | 0.95 |
| Alanine | | | -0.001 | 0.003 | 0.72 | 0.86 | -0.018 | 0.011 | 0.10 | 0.89 |
| Glutamine | | | -0.008 | 0.004 | 0.03 | 0.68 | -0.004 | 0.011 | 0.69 | 0.95 |
| Glycine | | | -0.005 | 0.003 | 0.10 | 0.68 | -0.010 | 0.010 | 0.30 | 0.95 |
| Histidine | | | -0.008 | 0.004 | 0.03 | 0.68 | 0.003 | 0.011 | 0.76 | 0.95 |
| Isoleucine | | | -0.003 | 0.004 | 0.42 | 0.69 | -0.006 | 0.010 | 0.52 | 0.95 |
| Leucine | | | -0.002 | 0.004 | 0.65 | 0.81 | 0.001 | 0.010 | 0.90 | 0.95 |
| Valine | | | 0.001 | 0.004 | 0.68 | 0.83 | -0.008 | 0.010 | 0.45 | 0.95 |
| Phenylalanine | | | 0.001 | 0.004 | 0.80 | 0.90 | -0.001 | 0.010 | 0.91 | 0.95 |
| Tyrosine | | | 0.002 | 0.004 | 0.65 | 0.81 | -0.019 | 0.011 | 0.09 | 0.89 |
| Acetate | | | 0.004 | 0.004 | 0.23 | 0.68 | 0.010 | 0.012 | 0.40 | 0.95 |
| Acetoacetate | | | 0.003 | 0.004 | 0.47 | 0.74 | 0.037 | 0.011 | **0.001** | 0.16 |
| 3-hydroxybutyrate | | | -0.001 | 0.004 | 0.86 | 0.93 | 0.032 | 0.011 | **0.0027** | 0.21 |
| Creatinine | | | -0.012 | 0.003 | **9.4E-05** | **0.01** | -0.015 | 0.012 | 0.20 | 0.89 |
| Albumin | | | -0.005 | 0.004 | 0.20 | 0.68 | -0.003 | 0.011 | 0.75 | 0.95 |
| GlycA (glycoprotein acetylation) | | | -0.001 | 0.004 | 0.70 | 0.85 | -0.006 | 0.010 | 0.59 | 0.95 |

| **PPD ≥ 4 mm + BOP** | | | | | | | | | | |
| --- | --- | --- | --- | --- | --- | --- | --- | --- | --- | --- |
|  | | | ≥ 20 teeth | | | | < 20 teeth | | | |
| **Metabolite** | | | Beta | SE | p | FDR | Beta | SE | p | FDR |
| VLDL | XXL | particles | -0.002 | 0.004 | 0.67 | 0.79 | 0.006 | 0.012 | 0.59 | 0.996 |
|  |  | lipid | -0.001 | 0.004 | 0.75 | 0.83 | 0.006 | 0.012 | 0.60 | 0.996 |
|  |  | phospholipid | -0.002 | 0.004 | 0.61 | 0.74 | 0.005 | 0.012 | 0.69 | 0.996 |
|  |  | cholesterol | -0.003 | 0.004 | 0.44 | 0.65 | 0.004 | 0.012 | 0.71 | 0.996 |
|  |  | cholesteryl ester | -0.003 | 0.004 | 0.40 | 0.65 | 0.007 | 0.011 | 0.53 | 0.996 |
|  |  | free cholesterol | -0.001 | 0.004 | 0.78 | 0.84 | 0.004 | 0.012 | 0.73 | 0.996 |
|  |  | triglycerides | -0.001 | 0.004 | 0.72 | 0.82 | 0.006 | 0.012 | 0.60 | 0.996 |
|  | XL | particles | -0.003 | 0.003 | 0.47 | 0.65 | -0.002 | 0.011 | 0.85 | 0.996 |
|  |  | lipid | -0.002 | 0.003 | 0.58 | 0.72 | -0.002 | 0.011 | 0.86 | 0.996 |
|  |  | phospholipid | -0.003 | 0.004 | 0.43 | 0.65 | -0.001 | 0.012 | 0.94 | 0.996 |
|  |  | cholesterol | -0.003 | 0.004 | 0.47 | 0.65 | 0.001 | 0.011 | 0.93 | 0.996 |
|  |  | cholesteryl ester | -0.002 | 0.004 | 0.51 | 0.68 | 0.001 | 0.011 | 0.90 | 0.996 |
|  |  | free cholesterol | -0.003 | 0.004 | 0.39 | 0.65 | 0.003 | 0.011 | 0.78 | 0.996 |
|  |  | triglycerides | -0.002 | 0.003 | 0.57 | 0.72 | -0.003 | 0.011 | 0.78 | 0.996 |
|  | L | particles | -0.003 | 0.003 | 0.43 | 0.65 | -0.004 | 0.011 | 0.70 | 0.996 |
|  |  | lipid | -0.001 | 0.003 | 0.74 | 0.83 | -0.005 | 0.010 | 0.63 | 0.996 |
|  |  | phospholipid | -0.002 | 0.003 | 0.48 | 0.66 | -0.004 | 0.011 | 0.70 | 0.996 |
|  |  | cholesterol | -0.002 | 0.003 | 0.53 | 0.69 | -0.003 | 0.011 | 0.81 | 0.996 |
|  |  | cholesteryl ester | -0.002 | 0.003 | 0.55 | 0.71 | -0.001 | 0.010 | 0.89 | 0.996 |
|  |  | free cholesterol | -0.003 | 0.003 | 0.45 | 0.65 | -0.003 | 0.011 | 0.77 | 0.996 |
|  |  | triglycerides | -0.002 | 0.003 | 0.62 | 0.74 | -0.005 | 0.010 | 0.62 | 0.996 |
|  | M | particles | -0.003 | 0.003 | 0.32 | 0.61 | -0.006 | 0.010 | 0.58 | 0.996 |
|  |  | lipid | -0.001 | 0.003 | 0.68 | 0.79 | -0.007 | 0.010 | 0.48 | 0.996 |
|  |  | phospholipid | -0.003 | 0.003 | 0.40 | 0.65 | -0.006 | 0.010 | 0.56 | 0.996 |
|  |  | cholesterol | -0.003 | 0.003 | 0.38 | 0.65 | -0.004 | 0.011 | 0.72 | 0.996 |
|  |  | cholesteryl ester | -0.004 | 0.003 | 0.31 | 0.61 | -0.001 | 0.011 | 0.93 | 0.996 |
|  |  | free cholesterol | -0.003 | 0.003 | 0.40 | 0.65 | -0.006 | 0.010 | 0.59 | 0.996 |
|  |  | triglycerides | -0.002 | 0.003 | 0.59 | 0.72 | -0.008 | 0.010 | 0.45 | 0.996 |
|  | S | particles | -0.004 | 0.003 | 0.23 | 0.52 | -0.006 | 0.010 | 0.57 | 0.996 |
|  |  | lipid | -0.003 | 0.003 | 0.42 | 0.65 | -0.007 | 0.011 | 0.53 | 0.996 |
|  |  | phospholipid | -0.003 | 0.003 | 0.32 | 0.61 | -0.006 | 0.011 | 0.57 | 0.996 |
|  |  | cholesterol | -0.004 | 0.004 | 0.23 | 0.52 | -0.005 | 0.012 | 0.65 | 0.996 |
|  |  | cholesteryl ester | -0.005 | 0.004 | 0.20 | 0.47 | -0.003 | 0.012 | 0.78 | 0.996 |
|  |  | free cholesterol | -0.004 | 0.003 | 0.29 | 0.61 | -0.007 | 0.011 | 0.52 | 0.996 |
|  |  | triglycerides | -0.002 | 0.003 | 0.46 | 0.65 | -0.006 | 0.010 | 0.52 | 0.996 |
|  | XS | particles | -0.005 | 0.004 | 0.18 | 0.43 | 0.000 | 0.012 | 0.99 | 0.996 |
|  |  | lipid | -0.005 | 0.004 | 0.18 | 0.43 | 0.000 | 0.013 | 0.98 | 0.996 |
|  |  | phospholipid | -0.005 | 0.004 | 0.14 | 0.38 | 0.000 | 0.013 | 0.98 | 0.996 |
|  |  | cholesterol | -0.006 | 0.004 | 0.12 | 0.36 | 0.004 | 0.013 | 0.75 | 0.996 |
|  |  | cholesteryl ester | -0.005 | 0.004 | 0.14 | 0.38 | 0.008 | 0.013 | 0.53 | 0.996 |
|  |  | free cholesterol | -0.006 | 0.004 | 0.11 | 0.33 | -0.003 | 0.013 | 0.79 | 0.996 |
|  |  | triglycerides | -0.002 | 0.003 | 0.53 | 0.69 | -0.007 | 0.011 | 0.54 | 0.996 |
| IDL | | particles | -0.006 | 0.004 | 0.10 | 0.33 | 0.004 | 0.013 | 0.75 | 0.996 |
|  |  | lipid | -0.006 | 0.004 | 0.08 | 0.29 | 0.002 | 0.013 | 0.88 | 0.996 |
|  |  | phospholipid | -0.006 | 0.004 | 0.11 | 0.33 | 0.004 | 0.013 | 0.79 | 0.996 |
|  |  | cholesterol | -0.007 | 0.004 | 0.06 | 0.28 | 0.002 | 0.013 | 0.86 | 0.996 |
|  |  | cholesteryl ester | -0.007 | 0.004 | 0.07 | 0.28 | 0.003 | 0.013 | 0.83 | 0.996 |
|  |  | free cholesterol | -0.007 | 0.004 | 0.07 | 0.28 | 0.003 | 0.013 | 0.81 | 0.996 |
|  |  | triglycerides | -0.001 | 0.003 | 0.87 | 0.90 | 0.005 | 0.012 | 0.70 | 0.996 |
| LDL | L | particles | -0.006 | 0.004 | 0.09 | 0.31 | 0.002 | 0.013 | 0.90 | 0.996 |
|  |  | lipid | -0.006 | 0.004 | 0.08 | 0.29 | -0.001 | 0.013 | 0.96 | 0.996 |
|  |  | phospholipid | -0.006 | 0.004 | 0.08 | 0.29 | 0.001 | 0.013 | 0.95 | 0.996 |
|  |  | cholesterol | -0.007 | 0.004 | 0.06 | 0.28 | -0.001 | 0.013 | 0.92 | 0.996 |
|  |  | cholesteryl ester | -0.007 | 0.004 | 0.07 | 0.28 | -0.002 | 0.013 | 0.90 | 0.996 |
|  |  | free cholesterol | -0.007 | 0.004 | 0.07 | 0.28 | 0.002 | 0.013 | 0.88 | 0.996 |
|  |  | triglycerides | -0.002 | 0.003 | 0.55 | 0.71 | 0.010 | 0.012 | 0.44 | 0.996 |
|  | M | particles | -0.007 | 0.004 | 0.08 | 0.29 | -0.002 | 0.013 | 0.87 | 0.996 |
|  |  | lipid | -0.007 | 0.004 | 0.07 | 0.28 | -0.005 | 0.013 | 0.72 | 0.996 |
|  |  | phospholipid | -0.007 | 0.004 | 0.07 | 0.28 | -0.004 | 0.013 | 0.73 | 0.996 |
|  |  | cholesterol | -0.007 | 0.004 | 0.06 | 0.28 | -0.004 | 0.013 | 0.73 | 0.996 |
|  |  | cholesteryl ester | -0.007 | 0.004 | 0.07 | 0.28 | -0.004 | 0.013 | 0.75 | 0.996 |
|  |  | free cholesterol | -0.008 | 0.004 | 0.04 | 0.28 | -0.003 | 0.013 | 0.79 | 0.996 |
|  |  | triglycerides | -0.002 | 0.003 | 0.49 | 0.67 | 0.009 | 0.012 | 0.47 | 0.996 |
|  | S | particles | -0.008 | 0.004 | 0.04 | 0.28 | -0.003 | 0.013 | 0.84 | 0.996 |
|  |  | lipid | -0.008 | 0.004 | 0.04 | 0.28 | -0.004 | 0.013 | 0.75 | 0.996 |
|  |  | phospholipid | -0.008 | 0.004 | 0.02 | 0.28 | -0.004 | 0.013 | 0.78 | 0.996 |
|  |  | cholesterol | -0.007 | 0.004 | 0.05 | 0.28 | -0.004 | 0.013 | 0.77 | 0.996 |
|  |  | cholesteryl ester | -0.007 | 0.004 | 0.06 | 0.28 | -0.003 | 0.013 | 0.79 | 0.996 |
|  |  | free cholesterol | -0.009 | 0.004 | 0.02 | 0.28 | -0.003 | 0.013 | 0.79 | 0.996 |
|  |  | triglycerides | -0.004 | 0.004 | 0.27 | 0.58 | 0.001 | 0.012 | 0.94 | 0.996 |
| HDL | XL | particles | -0.004 | 0.003 | 0.14 | 0.38 | 0.017 | 0.008 | 0.05 | 0.996 |
|  |  | lipid | -0.004 | 0.002 | 0.13 | 0.37 | 0.015 | 0.008 | 0.08 | 0.996 |
|  |  | phospholipid | -0.002 | 0.002 | 0.40 | 0.65 | 0.014 | 0.008 | 0.09 | 0.996 |
|  |  | cholesterol | -0.005 | 0.003 | 0.06 | 0.28 | 0.016 | 0.009 | 0.08 | 0.996 |
|  |  | cholesteryl ester | -0.005 | 0.003 | 0.05 | 0.28 | 0.017 | 0.009 | 0.06 | 0.996 |
|  |  | free cholesterol | -0.004 | 0.003 | 0.15 | 0.39 | 0.014 | 0.009 | 0.09 | 0.996 |
|  |  | triglycerides | -0.008 | 0.004 | 0.02 | 0.28 | 0.015 | 0.013 | 0.25 | 0.996 |
|  | L | particles | -0.003 | 0.003 | 0.26 | 0.57 | 0.011 | 0.009 | 0.20 | 0.996 |
|  |  | lipid | -0.002 | 0.003 | 0.42 | 0.65 | 0.011 | 0.009 | 0.24 | 0.996 |
|  |  | phospholipid | -0.003 | 0.003 | 0.30 | 0.61 | 0.010 | 0.009 | 0.27 | 0.996 |
|  |  | cholesterol | -0.002 | 0.003 | 0.45 | 0.65 | 0.010 | 0.009 | 0.23 | 0.996 |
|  |  | cholesteryl ester | -0.002 | 0.003 | 0.43 | 0.65 | 0.011 | 0.009 | 0.22 | 0.996 |
|  |  | free cholesterol | -0.002 | 0.003 | 0.36 | 0.65 | 0.010 | 0.008 | 0.26 | 0.996 |
|  |  | triglycerides | -0.003 | 0.003 | 0.30 | 0.61 | 0.014 | 0.011 | 0.20 | 0.996 |
|  | M | particles | -0.004 | 0.004 | 0.32 | 0.61 | -0.006 | 0.011 | 0.57 | 0.996 |
|  |  | lipid | -0.004 | 0.004 | 0.31 | 0.61 | -0.007 | 0.011 | 0.55 | 0.996 |
|  |  | phospholipid | -0.003 | 0.004 | 0.41 | 0.65 | -0.004 | 0.011 | 0.71 | 0.996 |
|  |  | cholesterol | -0.005 | 0.004 | 0.18 | 0.43 | -0.009 | 0.011 | 0.45 | 0.996 |
|  |  | cholesteryl ester | -0.005 | 0.004 | 0.16 | 0.40 | -0.009 | 0.011 | 0.43 | 0.996 |
|  |  | free cholesterol | -0.004 | 0.004 | 0.26 | 0.57 | -0.007 | 0.011 | 0.55 | 0.996 |
|  |  | triglycerides | 0.003 | 0.004 | 0.44 | 0.65 | -0.004 | 0.011 | 0.75 | 0.996 |
|  | S | particles | 0.001 | 0.004 | 0.76 | 0.83 | -0.020 | 0.013 | 0.11 | 0.996 |
|  |  | lipid | 0.001 | 0.004 | 0.80 | 0.85 | -0.022 | 0.013 | 0.11 | 0.996 |
|  |  | phospholipid | 0.003 | 0.004 | 0.46 | 0.65 | -0.020 | 0.013 | 0.12 | 0.996 |
|  |  | cholesterol | -0.003 | 0.004 | 0.50 | 0.68 | -0.016 | 0.013 | 0.20 | 0.996 |
|  |  | cholesteryl ester | -0.003 | 0.004 | 0.46 | 0.65 | -0.013 | 0.012 | 0.30 | 0.996 |
|  |  | free cholesterol | -0.001 | 0.004 | 0.88 | 0.90 | -0.020 | 0.013 | 0.12 | 0.996 |
|  |  | triglycerides | 0.003 | 0.003 | 0.33 | 0.62 | -0.007 | 0.011 | 0.55 | 0.996 |
| VLDL diameter |  |  | 0.005 | 0.003 | 0.13 | 0.37 | -0.008 | 0.010 | 0.41 | 0.996 |
| LDL diameter |  |  | 0.005 | 0.003 | 0.08 | 0.29 | 0.015 | 0.012 | 0.20 | 0.996 |
| HDL diameter |  |  | -0.002 | 0.002 | 0.47 | 0.65 | 0.013 | 0.008 | 0.11 | 0.996 |
| Cholesterol | serum total | | -0.009 | 0.004 | 0.02 | 0.28 | -0.001 | 0.013 | 0.92 | 0.996 |
|  | VLDL | | -0.003 | 0.003 | 0.32 | 0.61 | -0.003 | 0.011 | 0.81 | 0.996 |
|  | remnant | | -0.005 | 0.004 | 0.16 | 0.40 | -0.001 | 0.012 | 0.96 | 0.996 |
|  | LDL | | -0.007 | 0.004 | 0.05 | 0.28 | -0.004 | 0.013 | 0.78 | 0.996 |
|  | HDL | | -0.006 | 0.003 | 0.06 | 0.28 | 0.004 | 0.010 | 0.68 | 0.996 |
|  | HDL2 | | -0.005 | 0.003 | 0.09 | 0.31 | 0.004 | 0.010 | 0.66 | 0.996 |
|  | HDL3 | | -0.009 | 0.003 | 0.01 | 0.28 | 0.003 | 0.011 | 0.81 | 0.996 |
|  | esterified | | -0.009 | 0.004 | 0.02 | 0.28 | -0.001 | 0.013 | 0.92 | 0.996 |
|  | free | | -0.008 | 0.004 | 0.02 | 0.28 | 0.001 | 0.013 | 0.96 | 0.996 |
| Triglycerides | serum total | | -0.001 | 0.003 | 0.82 | 0.87 | -0.005 | 0.010 | 0.60 | 0.996 |
|  | VLDL | | 0.000 | 0.003 | 0.87 | 0.90 | -0.007 | 0.010 | 0.47 | 0.996 |
|  | LDL | | -0.003 | 0.003 | 0.45 | 0.65 | 0.008 | 0.012 | 0.50 | 0.996 |
|  | HDL | | -0.001 | 0.004 | 0.83 | 0.87 | 0.005 | 0.011 | 0.65 | 0.996 |
| Total phosphoglycerides | | | -0.007 | 0.004 | 0.07 | 0.28 | 0.000 | 0.012 | 0.97 | 0.996 |
| Phosphatidylcholine | | | -0.008 | 0.004 | 0.03 | 0.28 | 0.000 | 0.012 | 0.97 | 0.996 |
| Sphingomyelins | | | -0.007 | 0.003 | 0.04 | 0.28 | 0.001 | 0.012 | 0.91 | 0.996 |
| Total cholines | | | -0.008 | 0.004 | 0.03 | 0.28 | 0.000 | 0.012 | 0.996 | 0.996 |
| Apolipoprotein A-I | | | -0.010 | 0.003 | 0.003 | 0.24 | 0.001 | 0.011 | 0.95 | 0.996 |
| Apolipoprotein B | | | -0.006 | 0.004 | 0.10 | 0.33 | -0.003 | 0.012 | 0.78 | 0.996 |
| Apo-B / apoA-I | | | -0.004 | 0.003 | 0.24 | 0.54 | -0.003 | 0.010 | 0.80 | 0.996 |
| Fatty acids | total | | -0.003 | 0.004 | 0.46 | 0.65 | -0.002 | 0.012 | 0.86 | 0.996 |
|  | unsaturated | | -0.005 | 0.003 | 0.11 | 0.33 | -0.008 | 0.012 | 0.54 | 0.996 |
|  | DHA (docosahexaenoic acid) | | -0.003 | 0.004 | 0.32 | 0.61 | 0.004 | 0.012 | 0.72 | 0.996 |
|  | LA (linoleic acid) | | -0.007 | 0.004 | 0.07 | 0.28 | 0.001 | 0.013 | 0.96 | 0.996 |
|  | omega-3 | | -0.005 | 0.004 | 0.15 | 0.39 | -0.006 | 0.012 | 0.65 | 0.996 |
|  | omega-6 | | -0.007 | 0.004 | 0.04 | 0.28 | -0.001 | 0.013 | 0.93 | 0.996 |
|  | PUFA (polyunsaturated) | | -0.007 | 0.004 | 0.05 | 0.28 | -0.003 | 0.012 | 0.82 | 0.996 |
|  | MUFA (monounsaturated) | | 0.000 | 0.003 | 0.94 | 0.95 | -0.003 | 0.011 | 0.81 | 0.996 |
|  | SAFA (saturated) | | -0.002 | 0.004 | 0.62 | 0.74 | 0.000 | 0.012 | 0.99 | 0.996 |
|  | DHA% | | -0.003 | 0.003 | 0.40 | 0.65 | 0.002 | 0.012 | 0.86 | 0.996 |
|  | LA% | | -0.002 | 0.003 | 0.58 | 0.72 | 0.003 | 0.011 | 0.77 | 0.996 |
|  | omega-3% | | -0.005 | 0.003 | 0.11 | 0.33 | -0.007 | 0.013 | 0.61 | 0.996 |
|  | omega-6% | | -0.002 | 0.003 | 0.56 | 0.71 | 0.003 | 0.011 | 0.82 | 0.996 |
|  | PUFA% | | -0.003 | 0.003 | 0.37 | 0.65 | 0.001 | 0.011 | 0.92 | 0.996 |
|  | MUFA% | | 0.005 | 0.003 | 0.15 | 0.39 | -0.007 | 0.010 | 0.49 | 0.996 |
|  | SAFA% | | 0.002 | 0.004 | 0.56 | 0.71 | 0.008 | 0.013 | 0.55 | 0.996 |
| Glucose | | | 0.002 | 0.004 | 0.53 | 0.69 | -0.016 | 0.013 | 0.22 | 0.996 |
| Lactate | | | 0.000 | 0.004 | 0.99 | 0.99 | 0.009 | 0.013 | 0.51 | 0.996 |
| Pyruvate | | | 0.003 | 0.004 | 0.34 | 0.63 | 0.007 | 0.013 | 0.58 | 0.996 |
| Citrate | | | -0.001 | 0.004 | 0.70 | 0.80 | 0.009 | 0.012 | 0.49 | 0.996 |
| Glycerol | | | 0.000 | 0.004 | 0.92 | 0.94 | 0.014 | 0.016 | 0.38 | 0.996 |
| Alanine | | | -0.002 | 0.004 | 0.64 | 0.76 | -0.019 | 0.012 | 0.13 | 0.996 |
| Glutamine | | | -0.008 | 0.004 | 0.03 | 0.28 | -0.015 | 0.013 | 0.22 | 0.996 |
| Glycine | | | -0.007 | 0.003 | 0.04 | 0.28 | -0.008 | 0.011 | 0.47 | 0.996 |
| Histidine | | | -0.007 | 0.004 | 0.07 | 0.28 | 0.000 | 0.012 | 0.97 | 0.996 |
| Isoleucine | | | -0.001 | 0.004 | 0.75 | 0.83 | -0.003 | 0.011 | 0.79 | 0.996 |
| Leucine | | | -0.001 | 0.004 | 0.79 | 0.85 | 0.002 | 0.011 | 0.87 | 0.996 |
| Valine | | | 0.002 | 0.004 | 0.59 | 0.72 | -0.005 | 0.011 | 0.67 | 0.996 |
| Phenylalanine | | | -0.001 | 0.004 | 0.84 | 0.88 | 0.002 | 0.012 | 0.86 | 0.996 |
| Tyrosine | | | 0.001 | 0.004 | 0.77 | 0.84 | -0.014 | 0.012 | 0.28 | 0.996 |
| Acetate | | | 0.001 | 0.004 | 0.74 | 0.83 | -0.002 | 0.014 | 0.86 | 0.996 |
| Acetoacetate | | | 0.000 | 0.004 | 0.99 | 0.99 | 0.033 | 0.013 | 0.01 | 0.996 |
| 3-hydroxybutyrate | | | -0.004 | 0.004 | 0.36 | 0.65 | 0.023 | 0.012 | 0.07 | 0.996 |
| Creatinine | | | -0.013 | 0.003 | **1.8E-05** | **0.003** | -0.012 | 0.013 | 0.39 | 0.996 |
| Albumin | | | -0.007 | 0.004 | 0.06 | 0.28 | -0.013 | 0.012 | 0.29 | 0.996 |
| GlycA (glycoprotein acetylation) | | | -0.002 | 0.004 | 0.68 | 0.79 | 0.002 | 0.012 | 0.86 | 0.996 |

| **BOP** | | | | | | | | | | |
| --- | --- | --- | --- | --- | --- | --- | --- | --- | --- | --- |
|  | | | ≥ 20 teeth | | | | < 20 teeth | | | |
| **Metabolite** | | | Beta | SE | p | FDR | Beta | SE | p | FDR |
| VLDL | XXL | particles | 0.003 | 0.008 | 0.69 | 0.84 | 0.016 | 0.022 | 0.45 | 0.94 |
|  |  | lipid | 0.004 | 0.008 | 0.59 | 0.78 | 0.016 | 0.022 | 0.47 | 0.94 |
|  |  | phospholipid | 0.003 | 0.008 | 0.74 | 0.84 | 0.014 | 0.022 | 0.51 | 0.94 |
|  |  | cholesterol | 0.000 | 0.008 | 0.96 | 0.98 | 0.011 | 0.022 | 0.60 | 0.94 |
|  |  | cholesteryl ester | -0.001 | 0.008 | 0.87 | 0.92 | 0.012 | 0.021 | 0.57 | 0.94 |
|  |  | free cholesterol | 0.004 | 0.008 | 0.65 | 0.81 | 0.013 | 0.022 | 0.55 | 0.94 |
|  |  | triglycerides | 0.004 | 0.008 | 0.59 | 0.78 | 0.017 | 0.022 | 0.45 | 0.94 |
|  | XL | particles | 0.003 | 0.008 | 0.72 | 0.84 | 0.002 | 0.021 | 0.93 | 0.999 |
|  |  | lipid | 0.005 | 0.008 | 0.54 | 0.75 | 0.002 | 0.021 | 0.93 | 0.999 |
|  |  | phospholipid | 0.002 | 0.008 | 0.85 | 0.92 | 0.005 | 0.021 | 0.83 | 0.999 |
|  |  | cholesterol | 0.002 | 0.008 | 0.82 | 0.90 | 0.006 | 0.021 | 0.78 | 0.999 |
|  |  | cholesteryl ester | 0.003 | 0.008 | 0.72 | 0.84 | 0.005 | 0.020 | 0.81 | 0.999 |
|  |  | free cholesterol | 0.001 | 0.008 | 0.93 | 0.96 | 0.009 | 0.021 | 0.67 | 0.97 |
|  |  | triglycerides | 0.005 | 0.008 | 0.54 | 0.75 | 0.000 | 0.020 | 0.99 | 0.999 |
|  | L | particles | 0.003 | 0.008 | 0.71 | 0.84 | -0.004 | 0.019 | 0.82 | 0.999 |
|  |  | lipid | 0.008 | 0.008 | 0.28 | 0.47 | -0.006 | 0.019 | 0.76 | 0.999 |
|  |  | phospholipid | 0.004 | 0.008 | 0.64 | 0.81 | -0.005 | 0.020 | 0.81 | 0.999 |
|  |  | cholesterol | 0.004 | 0.008 | 0.57 | 0.77 | -0.003 | 0.020 | 0.89 | 0.999 |
|  |  | cholesteryl ester | 0.005 | 0.008 | 0.53 | 0.75 | -0.002 | 0.019 | 0.91 | 0.999 |
|  |  | free cholesterol | 0.002 | 0.008 | 0.76 | 0.85 | -0.002 | 0.020 | 0.91 | 0.999 |
|  |  | triglycerides | 0.007 | 0.008 | 0.36 | 0.57 | -0.006 | 0.019 | 0.76 | 0.999 |
|  | M | particles | 0.002 | 0.008 | 0.77 | 0.85 | -0.010 | 0.019 | 0.58 | 0.94 |
|  |  | lipid | 0.008 | 0.007 | 0.30 | 0.49 | -0.013 | 0.019 | 0.49 | 0.94 |
|  |  | phospholipid | 0.003 | 0.008 | 0.67 | 0.83 | -0.012 | 0.019 | 0.54 | 0.94 |
|  |  | cholesterol | 0.000 | 0.008 | 0.98 | 0.99 | -0.009 | 0.019 | 0.63 | 0.97 |
|  |  | cholesteryl ester | -0.004 | 0.008 | 0.64 | 0.81 | -0.007 | 0.020 | 0.73 | 0.999 |
|  |  | free cholesterol | 0.003 | 0.008 | 0.71 | 0.84 | -0.011 | 0.019 | 0.58 | 0.94 |
|  |  | triglycerides | 0.008 | 0.007 | 0.27 | 0.46 | -0.013 | 0.019 | 0.49 | 0.94 |
|  | S | particles | -0.002 | 0.008 | 0.83 | 0.90 | -0.015 | 0.019 | 0.42 | 0.94 |
|  |  | lipid | 0.001 | 0.008 | 0.87 | 0.92 | -0.017 | 0.019 | 0.38 | 0.94 |
|  |  | phospholipid | -0.001 | 0.008 | 0.94 | 0.96 | -0.014 | 0.019 | 0.46 | 0.94 |
|  |  | cholesterol | -0.007 | 0.008 | 0.38 | 0.58 | -0.020 | 0.021 | 0.35 | 0.94 |
|  |  | cholesteryl ester | -0.010 | 0.009 | 0.24 | 0.41 | -0.020 | 0.022 | 0.37 | 0.94 |
|  |  | free cholesterol | -0.003 | 0.008 | 0.72 | 0.84 | -0.018 | 0.020 | 0.38 | 0.94 |
|  |  | triglycerides | 0.005 | 0.007 | 0.48 | 0.70 | -0.014 | 0.018 | 0.43 | 0.94 |
|  | XS | particles | -0.015 | 0.009 | 0.08 | 0.17 | -0.017 | 0.023 | 0.47 | 0.94 |
|  |  | lipid | -0.015 | 0.008 | 0.07 | 0.15 | -0.018 | 0.023 | 0.42 | 0.94 |
|  |  | phospholipid | -0.017 | 0.009 | 0.04 | 0.09 | -0.020 | 0.023 | 0.40 | 0.94 |
|  |  | cholesterol | -0.019 | 0.009 | 0.03 | 0.08 | -0.012 | 0.023 | 0.60 | 0.94 |
|  |  | cholesteryl ester | -0.019 | 0.009 | 0.03 | 0.08 | -0.005 | 0.024 | 0.83 | 0.999 |
|  |  | free cholesterol | -0.017 | 0.009 | 0.04 | 0.09 | -0.025 | 0.023 | 0.29 | 0.94 |
|  |  | triglycerides | -0.001 | 0.008 | 0.92 | 0.96 | -0.017 | 0.020 | 0.39 | 0.94 |
| IDL |  | particles | -0.020 | 0.009 | 0.02 | 0.06 | -0.013 | 0.024 | 0.59 | 0.94 |
|  |  | lipid | -0.020 | 0.009 | 0.02 | 0.06 | -0.018 | 0.024 | 0.46 | 0.94 |
|  |  | phospholipid | -0.019 | 0.009 | 0.02 | 0.06 | -0.014 | 0.024 | 0.56 | 0.94 |
|  |  | cholesterol | -0.020 | 0.009 | 0.02 | 0.06 | -0.016 | 0.024 | 0.50 | 0.94 |
|  |  | cholesteryl ester | -0.020 | 0.009 | 0.02 | 0.06 | -0.014 | 0.024 | 0.54 | 0.94 |
|  |  | free cholesterol | -0.022 | 0.009 | 0.01 | 0.05 | -0.015 | 0.024 | 0.52 | 0.94 |
|  |  | triglycerides | -0.005 | 0.008 | 0.52 | 0.75 | -0.005 | 0.022 | 0.83 | 0.999 |
| LDL | L | particles | -0.019 | 0.009 | 0.03 | 0.08 | -0.016 | 0.023 | 0.49 | 0.94 |
|  |  | lipid | -0.019 | 0.009 | 0.03 | 0.08 | -0.021 | 0.024 | 0.38 | 0.94 |
|  |  | phospholipid | -0.019 | 0.009 | 0.03 | 0.08 | -0.017 | 0.024 | 0.47 | 0.94 |
|  |  | cholesterol | -0.020 | 0.009 | 0.02 | 0.06 | -0.022 | 0.024 | 0.35 | 0.94 |
|  |  | cholesteryl ester | -0.019 | 0.009 | 0.03 | 0.08 | -0.022 | 0.024 | 0.35 | 0.94 |
|  |  | free cholesterol | -0.021 | 0.009 | 0.01 | 0.05 | -0.017 | 0.024 | 0.47 | 0.94 |
|  |  | triglycerides | -0.011 | 0.008 | 0.16 | 0.29 | 0.003 | 0.022 | 0.89 | 0.999 |
|  | M | particles | -0.018 | 0.009 | 0.04 | 0.09 | -0.023 | 0.023 | 0.31 | 0.94 |
|  |  | lipid | -0.018 | 0.009 | 0.04 | 0.09 | -0.029 | 0.024 | 0.22 | 0.94 |
|  |  | phospholipid | -0.018 | 0.009 | 0.04 | 0.09 | -0.027 | 0.023 | 0.25 | 0.94 |
|  |  | cholesterol | -0.018 | 0.009 | 0.03 | 0.08 | -0.028 | 0.023 | 0.23 | 0.94 |
|  |  | cholesteryl ester | -0.018 | 0.009 | 0.04 | 0.09 | -0.027 | 0.023 | 0.25 | 0.94 |
|  |  | free cholesterol | -0.021 | 0.009 | 0.02 | 0.06 | -0.026 | 0.023 | 0.26 | 0.94 |
|  |  | triglycerides | -0.010 | 0.008 | 0.20 | 0.35 | 0.001 | 0.023 | 0.98 | 0.999 |
|  | S | particles | -0.020 | 0.009 | 0.02 | 0.06 | -0.022 | 0.023 | 0.35 | 0.94 |
|  |  | lipid | -0.019 | 0.009 | 0.02 | 0.06 | -0.026 | 0.024 | 0.28 | 0.94 |
|  |  | phospholipid | -0.021 | 0.009 | 0.02 | 0.06 | -0.020 | 0.024 | 0.39 | 0.94 |
|  |  | cholesterol | -0.019 | 0.009 | 0.02 | 0.06 | -0.026 | 0.023 | 0.27 | 0.94 |
|  |  | cholesteryl ester | -0.019 | 0.009 | 0.03 | 0.08 | -0.025 | 0.023 | 0.27 | 0.94 |
|  |  | free cholesterol | -0.022 | 0.009 | 0.01 | **0.048** | -0.023 | 0.023 | 0.34 | 0.94 |
|  |  | triglycerides | -0.008 | 0.008 | 0.36 | 0.57 | -0.007 | 0.023 | 0.76 | 0.999 |
| HDL | XL | particles | -0.018 | 0.006 | **0.002** | **0.04** | 0.033 | 0.015 | 0.03 | 0.94 |
|  |  | lipid | -0.017 | 0.006 | 0.004 | **0.048** | 0.024 | 0.016 | 0.12 | 0.94 |
|  |  | phospholipid | -0.016 | 0.006 | 0.01 | **0.048** | 0.025 | 0.015 | 0.09 | 0.94 |
|  |  | cholesterol | -0.017 | 0.006 | 0.01 | **0.048** | 0.029 | 0.016 | 0.08 | 0.94 |
|  |  | cholesteryl ester | -0.017 | 0.006 | 0.01 | **0.048** | 0.031 | 0.017 | 0.06 | 0.94 |
|  |  | free cholesterol | -0.019 | 0.006 | **0.002** | **0.04** | 0.030 | 0.016 | 0.05 | 0.94 |
|  |  | triglycerides | -0.023 | 0.009 | 0.01 | **0.048** | 0.019 | 0.024 | 0.43 | 0.94 |
|  | L | particles | -0.020 | 0.007 | 0.003 | **0.047** | 0.027 | 0.016 | 0.10 | 0.94 |
|  |  | lipid | -0.016 | 0.007 | 0.01 | **0.048** | 0.021 | 0.016 | 0.19 | 0.94 |
|  |  | phospholipid | -0.019 | 0.007 | 0.01 | **0.048** | 0.022 | 0.017 | 0.20 | 0.94 |
|  |  | cholesterol | -0.017 | 0.006 | 0.01 | **0.048** | 0.024 | 0.016 | 0.13 | 0.94 |
|  |  | cholesteryl ester | -0.017 | 0.006 | 0.01 | **0.048** | 0.025 | 0.016 | 0.11 | 0.94 |
|  |  | free cholesterol | -0.019 | 0.006 | 0.003 | **0.047** | 0.024 | 0.016 | 0.13 | 0.94 |
|  |  | triglycerides | -0.014 | 0.008 | 0.08 | 0.17 | 0.021 | 0.020 | 0.30 | 0.94 |
|  | M | particles | -0.013 | 0.009 | 0.13 | 0.26 | 0.000 | 0.021 | 0.996 | 0.999 |
|  |  | lipid | -0.012 | 0.009 | 0.15 | 0.28 | -0.002 | 0.021 | 0.92 | 0.999 |
|  |  | phospholipid | -0.012 | 0.008 | 0.14 | 0.27 | 0.002 | 0.021 | 0.93 | 0.999 |
|  |  | cholesterol | -0.015 | 0.009 | 0.09 | 0.18 | -0.004 | 0.021 | 0.85 | 0.999 |
|  |  | cholesteryl ester | -0.015 | 0.009 | 0.09 | 0.18 | -0.003 | 0.021 | 0.87 | 0.999 |
|  |  | free cholesterol | -0.016 | 0.009 | 0.07 | 0.15 | -0.004 | 0.021 | 0.84 | 0.999 |
|  |  | triglycerides | 0.007 | 0.008 | 0.41 | 0.62 | -0.001 | 0.020 | 0.95 | 0.999 |
|  | S | particles | 0.004 | 0.009 | 0.69 | 0.84 | -0.036 | 0.023 | 0.12 | 0.94 |
|  |  | lipid | 0.004 | 0.009 | 0.65 | 0.81 | -0.043 | 0.025 | 0.08 | 0.94 |
|  |  | phospholipid | 0.008 | 0.009 | 0.38 | 0.58 | -0.029 | 0.024 | 0.23 | 0.94 |
|  |  | cholesterol | -0.007 | 0.009 | 0.44 | 0.66 | -0.039 | 0.023 | 0.10 | 0.94 |
|  |  | cholesteryl ester | -0.008 | 0.009 | 0.34 | 0.54 | -0.035 | 0.023 | 0.13 | 0.94 |
|  |  | free cholesterol | 0.000 | 0.009 | 0.97 | 0.98 | -0.030 | 0.023 | 0.20 | 0.94 |
|  |  | triglycerides | 0.017 | 0.008 | 0.03 | 0.08 | -0.018 | 0.020 | 0.37 | 0.94 |
| VLDL diameter | | | 0.024 | 0.007 | **0.001** | **0.04** | -0.005 | 0.019 | 0.80 | 0.999 |
| LDL diameter | | | 0.004 | 0.007 | 0.57 | 0.77 | 0.044 | 0.021 | 0.04 | 0.94 |
| HDL diameter | | | -0.013 | 0.006 | 0.02 | 0.06 | 0.024 | 0.015 | 0.10 | 0.94 |
| Cholesterol | serum total | | -0.024 | 0.009 | 0.005 | **0.048** | -0.020 | 0.024 | 0.41 | 0.94 |
|  | VLDL | | -0.004 | 0.008 | 0.59 | 0.78 | -0.012 | 0.021 | 0.56 | 0.94 |
|  | remnant | | -0.013 | 0.009 | 0.14 | 0.27 | -0.016 | 0.023 | 0.49 | 0.94 |
|  | LDL | | -0.019 | 0.009 | 0.02 | 0.06 | -0.027 | 0.024 | 0.27 | 0.94 |
|  | HDL | | -0.024 | 0.007 | **0.001** | **0.04** | 0.011 | 0.019 | 0.57 | 0.94 |
|  | HDL2 | | -0.022 | 0.007 | **0.002** | **0.04** | 0.012 | 0.018 | 0.53 | 0.94 |
|  | HDL3 | | -0.025 | 0.008 | **0.002** | **0.04** | 0.000 | 0.021 | 0.996 | 0.999 |
|  | esterified | | -0.025 | 0.009 | 0.004 | **0.048** | -0.019 | 0.024 | 0.42 | 0.94 |
|  | free | | -0.023 | 0.009 | 0.01 | **0.048** | -0.017 | 0.024 | 0.49 | 0.94 |
| Triglycerides | serum total | | 0.007 | 0.008 | 0.34 | 0.54 | -0.009 | 0.019 | 0.65 | 0.97 |
|  | VLDL | | 0.011 | 0.007 | 0.15 | 0.28 | -0.012 | 0.019 | 0.54 | 0.94 |
|  | LDL | | -0.011 | 0.008 | 0.17 | 0.30 | 0.001 | 0.022 | 0.96 | 0.999 |
|  | HDL | | -0.005 | 0.009 | 0.53 | 0.75 | 0.009 | 0.021 | 0.67 | 0.97 |
| Total phosphoglycerides | | | -0.019 | 0.008 | 0.02 | 0.06 | -0.002 | 0.022 | 0.94 | 0.999 |
| Phosphatidylcholine | | | -0.022 | 0.008 | 0.01 | **0.048** | -0.003 | 0.022 | 0.89 | 0.999 |
| Sphingomyelins | | | -0.022 | 0.008 | 0.01 | **0.048** | -0.009 | 0.022 | 0.68 | 0.98 |
| Total cholines | | | -0.023 | 0.008 | 0.01 | **0.048** | -0.002 | 0.022 | 0.92 | 0.999 |
| Apolipoprotein A-I | | | -0.031 | 0.008 | **0.0001** | **0.02** | 0.002 | 0.021 | 0.91 | 0.999 |
| Apolipoprotein B | | | -0.013 | 0.008 | 0.12 | 0.24 | -0.019 | 0.022 | 0.38 | 0.94 |
| Apo-B / apoA-I | | | -0.001 | 0.007 | 0.89 | 0.94 | -0.023 | 0.019 | 0.24 | 0.94 |
| Fatty acids | total | | -0.006 | 0.009 | 0.45 | 0.67 | -0.009 | 0.022 | 0.67 | 0.97 |
|  | unsaturated | | -0.019 | 0.008 | 0.02 | 0.06 | -0.007 | 0.023 | 0.77 | 0.999 |
|  | DHA (docosahexaenoic acid) | | -0.019 | 0.008 | 0.02 | 0.06 | 0.011 | 0.022 | 0.60 | 0.94 |
|  | LA (linoleic acid) | | -0.016 | 0.009 | 0.08 | 0.17 | -0.018 | 0.024 | 0.46 | 0.94 |
|  | omega-3 | | -0.022 | 0.008 | 0.01 | **0.048** | -0.010 | 0.022 | 0.65 | 0.97 |
|  | omega-6 | | -0.019 | 0.009 | 0.03 | 0.08 | -0.016 | 0.023 | 0.49 | 0.94 |
|  | PUFA (polyunsaturated) | | -0.021 | 0.009 | 0.02 | 0.06 | -0.016 | 0.023 | 0.48 | 0.94 |
|  | MUFA (monounsaturated) | | 0.003 | 0.008 | 0.73 | 0.84 | -0.006 | 0.021 | 0.78 | 0.999 |
|  | SAFA (saturated) | | -0.004 | 0.009 | 0.63 | 0.81 | -0.003 | 0.022 | 0.88 | 0.999 |
|  | DHA% | | -0.019 | 0.008 | 0.01 | **0.048** | 0.018 | 0.022 | 0.42 | 0.94 |
|  | LA% | | -0.003 | 0.008 | 0.73 | 0.84 | -0.013 | 0.020 | 0.51 | 0.94 |
|  | omega-3% | | -0.025 | 0.008 | **0.002** | **0.04** | -0.001 | 0.023 | 0.97 | 0.999 |
|  | omega-6% | | -0.008 | 0.008 | 0.29 | 0.48 | -0.006 | 0.020 | 0.78 | 0.999 |
|  | PUFA% | | -0.015 | 0.008 | 0.06 | 0.13 | -0.008 | 0.021 | 0.69 | 0.98 |
|  | MUFA% | | 0.021 | 0.007 | 0.004 | **0.048** | -0.006 | 0.018 | 0.72 | 0.999 |
|  | SAFA% | | 0.005 | 0.009 | 0.56 | 0.77 | 0.024 | 0.024 | 0.32 | 0.94 |
| Glucose | | | -0.002 | 0.008 | 0.86 | 0.92 | -0.042 | 0.024 | 0.08 | 0.94 |
| Lactate | | | -0.011 | 0.008 | 0.17 | 0.30 | 0.000 | 0.024 | 0.99 | 0.999 |
| Pyruvate | | | 0.002 | 0.008 | 0.76 | 0.85 | 0.000 | 0.023 | 0.999 | 0.999 |
| Citrate | | | -0.004 | 0.008 | 0.61 | 0.79 | 0.000 | 0.023 | 0.998 | 0.999 |
| Glycerol | | | 0.001 | 0.010 | 0.90 | 0.94 | 0.005 | 0.029 | 0.86 | 0.999 |
| Alanine | | | -0.007 | 0.009 | 0.43 | 0.65 | -0.040 | 0.023 | 0.08 | 0.94 |
| Glutamine | | | -0.013 | 0.009 | 0.15 | 0.28 | -0.040 | 0.023 | 0.08 | 0.94 |
| Glycine | | | -0.012 | 0.008 | 0.13 | 0.26 | -0.028 | 0.020 | 0.17 | 0.94 |
| Histidine | | | -0.020 | 0.009 | 0.03 | 0.08 | -0.011 | 0.023 | 0.64 | 0.97 |
| Isoleucine | | | 0.004 | 0.009 | 0.61 | 0.79 | 0.001 | 0.021 | 0.95 | 0.999 |
| Leucine | | | -0.003 | 0.009 | 0.75 | 0.85 | 0.011 | 0.021 | 0.61 | 0.95 |
| Valine | | | 0.006 | 0.009 | 0.52 | 0.75 | 0.001 | 0.021 | 0.95 | 0.999 |
| Phenylalanine | | | -0.010 | 0.009 | 0.26 | 0.44 | 0.003 | 0.022 | 0.88 | 0.999 |
| Tyrosine | | | 0.000 | 0.009 | 0.99 | 0.99 | -0.020 | 0.023 | 0.39 | 0.94 |
| Acetate | | | -0.021 | 0.009 | 0.02 | 0.06 | -0.030 | 0.025 | 0.23 | 0.94 |
| Acetoacetate | | | -0.013 | 0.009 | 0.16 | 0.29 | 0.017 | 0.023 | 0.46 | 0.94 |
| 3-hydroxybutyrate | | | -0.019 | 0.009 | 0.04 | 0.09 | 0.012 | 0.022 | 0.60 | 0.94 |
| Creatinine | | | -0.020 | 0.007 | 0.01 | **0.048** | -0.028 | 0.025 | 0.27 | 0.94 |
| Albumin | | | -0.023 | 0.009 | 0.01 | **0.048** | -0.030 | 0.023 | 0.19 | 0.94 |
| GlycA (glycoprotein acetylation) | | | 0.003 | 0.009 | 0.72 | 0.84 | -0.002 | 0.022 | 0.93 | 0.999 |

| **PIBI** | | | | | | | | | | |
| --- | --- | --- | --- | --- | --- | --- | --- | --- | --- | --- |
|  | | | ≥ 20 teeth | | | | < 20 teeth | | | |
| **Metabolite** | | | Beta | SE | p | FDR | Beta | SE | p | FDR |
| VLDL | XXL | particles | -0.002 | 0.003 | 0.51 | 0.76 | -0.001 | 0.008 | 0.92 | 1.00 |
|  |  | lipid | -0.002 | 0.003 | 0.56 | 0.79 | -0.001 | 0.008 | 0.91 | 1.00 |
|  |  | phospholipid | -0.002 | 0.003 | 0.47 | 0.74 | -0.002 | 0.008 | 0.79 | 1.00 |
|  |  | cholesterol | -0.002 | 0.003 | 0.43 | 0.70 | -0.002 | 0.008 | 0.78 | 1.00 |
|  |  | cholesteryl ester | -0.002 | 0.003 | 0.41 | 0.70 | 0.000 | 0.008 | 1.00 | 1.00 |
|  |  | free cholesterol | -0.001 | 0.003 | 0.64 | 0.81 | -0.003 | 0.008 | 0.74 | 1.00 |
|  |  | triglycerides | -0.002 | 0.003 | 0.53 | 0.79 | -0.001 | 0.008 | 0.93 | 1.00 |
|  | XL | particles | -0.002 | 0.003 | 0.36 | 0.70 | -0.007 | 0.007 | 0.38 | 1.00 |
|  |  | lipid | -0.002 | 0.003 | 0.43 | 0.70 | -0.007 | 0.007 | 0.38 | 1.00 |
|  |  | phospholipid | -0.002 | 0.003 | 0.37 | 0.70 | -0.006 | 0.008 | 0.44 | 1.00 |
|  |  | cholesterol | -0.002 | 0.003 | 0.40 | 0.70 | -0.004 | 0.008 | 0.56 | 1.00 |
|  |  | cholesteryl ester | -0.002 | 0.003 | 0.42 | 0.70 | -0.004 | 0.007 | 0.60 | 1.00 |
|  |  | free cholesterol | -0.003 | 0.003 | 0.35 | 0.69 | -0.003 | 0.007 | 0.64 | 1.00 |
|  |  | triglycerides | -0.002 | 0.003 | 0.41 | 0.70 | -0.007 | 0.007 | 0.33 | 1.00 |
|  | L | particles | -0.003 | 0.003 | 0.29 | 0.67 | -0.007 | 0.007 | 0.28 | 1.00 |
|  |  | lipid | -0.002 | 0.003 | 0.49 | 0.74 | -0.008 | 0.007 | 0.24 | 1.00 |
|  |  | phospholipid | -0.003 | 0.003 | 0.33 | 0.67 | -0.008 | 0.007 | 0.28 | 1.00 |
|  |  | cholesterol | -0.002 | 0.003 | 0.38 | 0.70 | -0.007 | 0.007 | 0.35 | 1.00 |
|  |  | cholesteryl ester | -0.002 | 0.003 | 0.40 | 0.70 | -0.006 | 0.007 | 0.41 | 1.00 |
|  |  | free cholesterol | -0.003 | 0.003 | 0.33 | 0.67 | -0.007 | 0.007 | 0.32 | 1.00 |
|  |  | triglycerides | -0.002 | 0.003 | 0.39 | 0.70 | -0.008 | 0.007 | 0.24 | 1.00 |
|  | M | particles | -0.003 | 0.003 | 0.20 | 0.67 | -0.009 | 0.007 | 0.20 | 1.00 |
|  |  | lipid | -0.002 | 0.002 | 0.43 | 0.70 | -0.010 | 0.007 | 0.15 | 1.00 |
|  |  | phospholipid | -0.003 | 0.002 | 0.25 | 0.67 | -0.009 | 0.007 | 0.19 | 1.00 |
|  |  | cholesterol | -0.003 | 0.003 | 0.31 | 0.67 | -0.007 | 0.007 | 0.30 | 1.00 |
|  |  | cholesteryl ester | -0.003 | 0.003 | 0.32 | 0.67 | -0.005 | 0.007 | 0.48 | 1.00 |
|  |  | free cholesterol | -0.003 | 0.003 | 0.26 | 0.67 | -0.009 | 0.007 | 0.21 | 1.00 |
|  |  | triglycerides | -0.002 | 0.002 | 0.32 | 0.67 | -0.010 | 0.007 | 0.14 | 1.00 |
|  | S | particles | -0.003 | 0.003 | 0.17 | 0.67 | -0.009 | 0.007 | 0.21 | 1.00 |
|  |  | lipid | -0.002 | 0.002 | 0.32 | 0.67 | -0.009 | 0.007 | 0.20 | 1.00 |
|  |  | phospholipid | -0.003 | 0.003 | 0.27 | 0.67 | -0.008 | 0.007 | 0.24 | 1.00 |
|  |  | cholesterol | -0.003 | 0.003 | 0.28 | 0.67 | -0.007 | 0.008 | 0.35 | 1.00 |
|  |  | cholesteryl ester | -0.003 | 0.003 | 0.28 | 0.67 | -0.005 | 0.008 | 0.55 | 1.00 |
|  |  | free cholesterol | -0.003 | 0.003 | 0.25 | 0.67 | -0.009 | 0.007 | 0.20 | 1.00 |
|  |  | triglycerides | -0.003 | 0.002 | 0.28 | 0.67 | -0.009 | 0.007 | 0.17 | 1.00 |
|  | XS | particles | -0.003 | 0.003 | 0.34 | 0.68 | -0.002 | 0.008 | 0.78 | 1.00 |
|  |  | lipid | -0.003 | 0.003 | 0.37 | 0.70 | -0.002 | 0.008 | 0.77 | 1.00 |
|  |  | phospholipid | -0.003 | 0.003 | 0.30 | 0.67 | 0.000 | 0.008 | 0.995 | 1.00 |
|  |  | cholesterol | -0.003 | 0.003 | 0.33 | 0.67 | 0.002 | 0.008 | 0.78 | 1.00 |
|  |  | cholesteryl ester | -0.002 | 0.003 | 0.41 | 0.70 | 0.005 | 0.009 | 0.55 | 1.00 |
|  |  | free cholesterol | -0.004 | 0.003 | 0.21 | 0.67 | -0.003 | 0.008 | 0.75 | 1.00 |
|  |  | triglycerides | -0.002 | 0.002 | 0.44 | 0.70 | -0.010 | 0.007 | 0.18 | 1.00 |
| IDL | | particles | -0.003 | 0.003 | 0.30 | 0.67 | 0.004 | 0.009 | 0.64 | 1.00 |
|  |  | lipid | -0.003 | 0.003 | 0.26 | 0.67 | 0.003 | 0.009 | 0.75 | 1.00 |
|  |  | phospholipid | -0.003 | 0.003 | 0.32 | 0.67 | 0.004 | 0.009 | 0.61 | 1.00 |
|  |  | cholesterol | -0.004 | 0.003 | 0.21 | 0.67 | 0.003 | 0.009 | 0.70 | 1.00 |
|  |  | cholesteryl ester | -0.003 | 0.003 | 0.22 | 0.67 | 0.003 | 0.009 | 0.71 | 1.00 |
|  |  | free cholesterol | -0.003 | 0.003 | 0.23 | 0.67 | 0.005 | 0.009 | 0.57 | 1.00 |
|  |  | triglycerides | 0.000 | 0.003 | 0.97 | 0.995 | 0.000 | 0.008 | 0.97 | 1.00 |
| LDL | L | particles | -0.004 | 0.003 | 0.22 | 0.67 | 0.003 | 0.008 | 0.76 | 1.00 |
|  |  | lipid | -0.004 | 0.003 | 0.20 | 0.67 | 0.001 | 0.009 | 0.89 | 1.00 |
|  |  | phospholipid | -0.003 | 0.003 | 0.22 | 0.67 | 0.002 | 0.008 | 0.79 | 1.00 |
|  |  | cholesterol | -0.004 | 0.003 | 0.16 | 0.67 | 0.001 | 0.009 | 0.92 | 1.00 |
|  |  | cholesteryl ester | -0.004 | 0.003 | 0.16 | 0.67 | 0.000 | 0.008 | 0.98 | 1.00 |
|  |  | free cholesterol | -0.004 | 0.003 | 0.20 | 0.67 | 0.004 | 0.008 | 0.64 | 1.00 |
|  |  | triglycerides | 0.000 | 0.003 | 0.99 | 0.996 | 0.005 | 0.008 | 0.50 | 1.00 |
|  | M | particles | -0.004 | 0.003 | 0.15 | 0.67 | 0.000 | 0.008 | 0.98 | 1.00 |
|  |  | lipid | -0.004 | 0.003 | 0.15 | 0.67 | -0.001 | 0.008 | 0.89 | 1.00 |
|  |  | phospholipid | -0.004 | 0.003 | 0.18 | 0.67 | -0.001 | 0.008 | 0.90 | 1.00 |
|  |  | cholesterol | -0.004 | 0.003 | 0.12 | 0.67 | -0.001 | 0.008 | 0.88 | 1.00 |
|  |  | cholesteryl ester | -0.004 | 0.003 | 0.13 | 0.67 | -0.001 | 0.008 | 0.87 | 1.00 |
|  |  | free cholesterol | -0.005 | 0.003 | 0.11 | 0.67 | 0.001 | 0.008 | 0.95 | 1.00 |
|  |  | triglycerides | 0.000 | 0.003 | 0.94 | 0.98 | 0.006 | 0.008 | 0.43 | 1.00 |
|  | S | particles | -0.005 | 0.003 | 0.08 | 0.67 | 0.000 | 0.008 | 0.999 | 1.00 |
|  |  | lipid | -0.005 | 0.003 | 0.08 | 0.67 | -0.001 | 0.008 | 0.93 | 1.00 |
|  |  | phospholipid | -0.005 | 0.003 | 0.06 | 0.67 | 0.000 | 0.008 | 0.98 | 1.00 |
|  |  | cholesterol | -0.005 | 0.003 | 0.09 | 0.67 | -0.001 | 0.008 | 0.94 | 1.00 |
|  |  | cholesteryl ester | -0.005 | 0.003 | 0.10 | 0.67 | -0.001 | 0.008 | 0.93 | 1.00 |
|  |  | free cholesterol | -0.006 | 0.003 | 0.05 | 0.67 | 0.001 | 0.008 | 0.93 | 1.00 |
|  |  | triglycerides | -0.003 | 0.003 | 0.34 | 0.68 | -0.001 | 0.008 | 0.91 | 1.00 |
| HDL | XL | particles | -0.001 | 0.002 | 0.66 | 0.82 | 0.012 | 0.006 | 0.03 | 1.00 |
|  |  | lipid | -0.001 | 0.002 | 0.58 | 0.79 | 0.010 | 0.006 | 0.07 | 1.00 |
|  |  | phospholipid | 0.000 | 0.002 | 0.88 | 0.95 | 0.009 | 0.005 | 0.08 | 1.00 |
|  |  | cholesterol | -0.002 | 0.002 | 0.31 | 0.67 | 0.012 | 0.006 | 0.04 | 1.00 |
|  |  | cholesteryl ester | -0.002 | 0.002 | 0.24 | 0.67 | 0.013 | 0.006 | 0.03 | 1.00 |
|  |  | free cholesterol | -0.001 | 0.002 | 0.63 | 0.81 | 0.010 | 0.006 | 0.06 | 1.00 |
|  |  | triglycerides | -0.004 | 0.003 | 0.15 | 0.67 | 0.007 | 0.008 | 0.37 | 1.00 |
|  | L | particles | 0.000 | 0.002 | 0.95 | 0.98 | 0.010 | 0.006 | 0.09 | 1.00 |
|  |  | lipid | 0.001 | 0.002 | 0.75 | 0.88 | 0.009 | 0.006 | 0.12 | 1.00 |
|  |  | phospholipid | 0.000 | 0.002 | 0.90 | 0.95 | 0.010 | 0.006 | 0.10 | 1.00 |
|  |  | cholesterol | 0.001 | 0.002 | 0.72 | 0.87 | 0.008 | 0.006 | 0.14 | 1.00 |
|  |  | cholesteryl ester | 0.001 | 0.002 | 0.74 | 0.87 | 0.009 | 0.006 | 0.12 | 1.00 |
|  |  | free cholesterol | 0.000 | 0.002 | 0.85 | 0.94 | 0.008 | 0.006 | 0.16 | 1.00 |
|  |  | triglycerides | 0.000 | 0.003 | 0.88 | 0.95 | 0.011 | 0.007 | 0.13 | 1.00 |
|  | M | particles | 0.000 | 0.003 | 0.92 | 0.97 | 0.002 | 0.007 | 0.77 | 1.00 |
|  |  | lipid | -0.001 | 0.003 | 0.86 | 0.94 | 0.002 | 0.008 | 0.82 | 1.00 |
|  |  | phospholipid | 0.000 | 0.003 | 0.93 | 0.97 | 0.004 | 0.007 | 0.63 | 1.00 |
|  |  | cholesterol | -0.001 | 0.003 | 0.60 | 0.80 | 0.001 | 0.008 | 0.93 | 1.00 |
|  |  | cholesteryl ester | -0.002 | 0.003 | 0.55 | 0.79 | 0.000 | 0.008 | 0.97 | 1.00 |
|  |  | free cholesterol | -0.001 | 0.003 | 0.83 | 0.92 | 0.002 | 0.007 | 0.75 | 1.00 |
|  |  | triglycerides | 0.003 | 0.003 | 0.30 | 0.67 | -0.004 | 0.007 | 0.54 | 1.00 |
|  | S | particles | 0.001 | 0.003 | 0.73 | 0.87 | -0.008 | 0.008 | 0.36 | 1.00 |
|  |  | lipid | 0.000 | 0.003 | 0.87 | 0.95 | -0.008 | 0.009 | 0.38 | 1.00 |
|  |  | phospholipid | 0.002 | 0.003 | 0.47 | 0.74 | -0.008 | 0.009 | 0.36 | 1.00 |
|  |  | cholesterol | -0.002 | 0.003 | 0.58 | 0.79 | -0.005 | 0.008 | 0.59 | 1.00 |
|  |  | cholesteryl ester | -0.002 | 0.003 | 0.55 | 0.79 | -0.003 | 0.008 | 0.71 | 1.00 |
|  |  | free cholesterol | 0.000 | 0.003 | 0.99 | 0.996 | -0.007 | 0.008 | 0.37 | 1.00 |
|  |  | triglycerides | 0.001 | 0.003 | 0.78 | 0.90 | -0.006 | 0.007 | 0.40 | 1.00 |
| VLDL diameter | | | 0.002 | 0.002 | 0.44 | 0.70 | -0.010 | 0.007 | 0.14 | 1.00 |
| LDL diameter | | | 0.006 | 0.002 | 0.02 | 0.67 | 0.008 | 0.008 | 0.32 | 1.00 |
| HDL diameter | | | 0.001 | 0.002 | 0.74 | 0.87 | 0.008 | 0.005 | 0.13 | 1.00 |
| Cholesterol | serum total | | -0.005 | 0.003 | 0.09 | 0.67 | 0.002 | 0.009 | 0.79 | 1.00 |
|  | VLDL | | -0.003 | 0.003 | 0.33 | 0.67 | -0.007 | 0.008 | 0.39 | 1.00 |
|  | remnant | | -0.003 | 0.003 | 0.29 | 0.67 | -0.003 | 0.008 | 0.71 | 1.00 |
|  | LDL | | -0.004 | 0.003 | 0.13 | 0.67 | -0.001 | 0.009 | 0.95 | 1.00 |
|  | HDL | | -0.002 | 0.002 | 0.43 | 0.70 | 0.007 | 0.007 | 0.29 | 1.00 |
|  | HDL2 | | -0.001 | 0.002 | 0.56 | 0.79 | 0.007 | 0.007 | 0.29 | 1.00 |
|  | HDL3 | | -0.005 | 0.003 | 0.05 | 0.67 | 0.007 | 0.008 | 0.37 | 1.00 |
|  | esterified | | -0.005 | 0.003 | 0.08 | 0.67 | 0.003 | 0.009 | 0.77 | 1.00 |
|  | free | | -0.005 | 0.003 | 0.10 | 0.67 | 0.003 | 0.009 | 0.75 | 1.00 |
| Triglycerides | serum total | | -0.001 | 0.002 | 0.61 | 0.80 | -0.008 | 0.007 | 0.22 | 1.00 |
|  | VLDL | | -0.001 | 0.002 | 0.55 | 0.79 | -0.010 | 0.007 | 0.15 | 1.00 |
|  | LDL | | -0.001 | 0.003 | 0.82 | 0.92 | 0.004 | 0.008 | 0.59 | 1.00 |
|  | HDL | | 0.001 | 0.003 | 0.74 | 0.87 | 0.001 | 0.008 | 0.85 | 1.00 |
| Total phosphoglycerides | | | -0.003 | 0.003 | 0.31 | 0.67 | 0.002 | 0.008 | 0.76 | 1.00 |
| Phosphatidylcholine | | | -0.004 | 0.003 | 0.18 | 0.67 | 0.002 | 0.008 | 0.79 | 1.00 |
| Sphingomyelins | | | -0.004 | 0.003 | 0.17 | 0.67 | 0.006 | 0.008 | 0.48 | 1.00 |
| Total cholines | | | -0.004 | 0.003 | 0.17 | 0.67 | 0.003 | 0.008 | 0.74 | 1.00 |
| Apolipoprotein A-I | | | -0.005 | 0.003 | 0.06 | 0.67 | 0.006 | 0.008 | 0.45 | 1.00 |
| Apolipoprotein B | | | -0.004 | 0.003 | 0.15 | 0.67 | -0.005 | 0.008 | 0.51 | 1.00 |
| Apo-B / apoA-I | | | -0.004 | 0.002 | 0.12 | 0.67 | -0.005 | 0.007 | 0.45 | 1.00 |
| Fatty acids | total | | -0.001 | 0.003 | 0.62 | 0.80 | -0.004 | 0.008 | 0.58 | 1.00 |
|  | unsaturated | | -0.004 | 0.003 | 0.13 | 0.67 | 0.000 | 0.008 | 0.99 | 1.00 |
|  | DHA (docosahexaenoic acid) | | -0.001 | 0.003 | 0.66 | 0.82 | 0.005 | 0.008 | 0.56 | 1.00 |
|  | LA (linoleic acid) | | -0.004 | 0.003 | 0.13 | 0.67 | -0.001 | 0.009 | 0.88 | 1.00 |
|  | omega-3 | | -0.003 | 0.003 | 0.30 | 0.67 | -0.002 | 0.008 | 0.78 | 1.00 |
|  | omega-6 | | -0.005 | 0.003 | 0.11 | 0.67 | -0.001 | 0.008 | 0.93 | 1.00 |
|  | PUFA (polyunsaturated) | | -0.004 | 0.003 | 0.13 | 0.67 | -0.001 | 0.008 | 0.87 | 1.00 |
|  | MUFA (monounsaturated) | | 0.000 | 0.003 | 0.99 | 0.996 | -0.006 | 0.008 | 0.40 | 1.00 |
|  | SAFA (saturated) | | -0.001 | 0.003 | 0.78 | 0.90 | -0.003 | 0.008 | 0.70 | 1.00 |
|  | DHA% | | -0.001 | 0.003 | 0.61 | 0.80 | 0.005 | 0.008 | 0.51 | 1.00 |
|  | LA% | | -0.002 | 0.003 | 0.48 | 0.74 | 0.003 | 0.007 | 0.65 | 1.00 |
|  | omega-3% | | -0.003 | 0.003 | 0.20 | 0.67 | 0.000 | 0.008 | 0.96 | 1.00 |
|  | omega-6% | | -0.001 | 0.003 | 0.58 | 0.79 | 0.005 | 0.007 | 0.48 | 1.00 |
|  | PUFA% | | -0.002 | 0.003 | 0.48 | 0.74 | 0.005 | 0.007 | 0.51 | 1.00 |
|  | MUFA% | | 0.003 | 0.002 | 0.24 | 0.67 | -0.007 | 0.006 | 0.25 | 1.00 |
|  | SAFA% | | 0.002 | 0.003 | 0.54 | 0.79 | 0.003 | 0.009 | 0.68 | 1.00 |
| Glucose | | | 0.003 | 0.003 | 0.26 | 0.67 | -0.009 | 0.008 | 0.29 | 1.00 |
| Lactate | | | 0.001 | 0.003 | 0.73 | 0.87 | 0.004 | 0.008 | 0.66 | 1.00 |
| Pyruvate | | | 0.003 | 0.003 | 0.29 | 0.67 | -0.002 | 0.008 | 0.83 | 1.00 |
| Citrate | | | -0.001 | 0.003 | 0.79 | 0.90 | 0.003 | 0.008 | 0.72 | 1.00 |
| Glycerol | | | 0.001 | 0.003 | 0.83 | 0.92 | 0.003 | 0.010 | 0.73 | 1.00 |
| Alanine | | | 0.000 | 0.003 | 0.89 | 0.95 | -0.013 | 0.008 | 0.12 | 1.00 |
| Glutamine | | | -0.006 | 0.003 | 0.04 | 0.67 | -0.004 | 0.008 | 0.63 | 1.00 |
| Glycine | | | -0.004 | 0.002 | 0.07 | 0.67 | -0.005 | 0.007 | 0.47 | 1.00 |
| Histidine | | | -0.006 | 0.003 | 0.03 | 0.67 | 0.001 | 0.008 | 0.88 | 1.00 |
| Isoleucine | | | -0.002 | 0.003 | 0.49 | 0.74 | -0.003 | 0.007 | 0.67 | 1.00 |
| Leucine | | | -0.001 | 0.003 | 0.73 | 0.87 | 0.003 | 0.008 | 0.67 | 1.00 |
| Valine | | | 0.002 | 0.003 | 0.58 | 0.79 | -0.004 | 0.008 | 0.62 | 1.00 |
| Phenylalanine | | | 0.001 | 0.003 | 0.79 | 0.90 | 0.001 | 0.008 | 0.93 | 1.00 |
| Tyrosine | | | 0.001 | 0.003 | 0.64 | 0.81 | -0.015 | 0.008 | 0.06 | 1.00 |
| Acetate | | | 0.003 | 0.003 | 0.30 | 0.67 | 0.008 | 0.009 | 0.39 | 1.00 |
| Acetoacetate | | | 0.002 | 0.003 | 0.44 | 0.70 | 0.029 | 0.008 | **0.0004** | 0.06 |
| 3-hydroxybutyrate | | | 0.000 | 0.003 | 0.999 | 0.999 | 0.026 | 0.008 | **0.002** | 0.16 |
| Creatinine | | | -0.009 | 0.002 | **0.0002** | **0.03** | -0.006 | 0.009 | 0.50 | 1.00 |
| Albumin | | | -0.004 | 0.003 | 0.21 | 0.67 | -0.004 | 0.008 | 0.64 | 1.00 |
| GlycA (glycoprotein acetylation) | | | -0.001 | 0.003 | 0.62 | 0.80 | -0.003 | 0.008 | 0.74 | 1.00 |

| **ABL** | | | | | | | | | | |
| --- | --- | --- | --- | --- | --- | --- | --- | --- | --- | --- |
|  | | | ≥ 20 teeth | | | | < 20 teeth | | | |
| **Metabolite** | | | Beta | SE | p | FDR | Beta | SE | p | FDR |
| VLDL | XXL | particles | -0.028 | 0.036 | 0.44 | 0.73 | -0.057 | 0.047 | 0.22 | 0.56 |
|  |  | lipid | -0.025 | 0.036 | 0.49 | 0.73 | -0.054 | 0.046 | 0.25 | 0.56 |
|  |  | phospholipid | -0.029 | 0.036 | 0.42 | 0.72 | -0.058 | 0.047 | 0.22 | 0.56 |
|  |  | cholesterol | -0.030 | 0.036 | 0.40 | 0.72 | -0.052 | 0.047 | 0.27 | 0.56 |
|  |  | cholesteryl ester | -0.030 | 0.037 | 0.41 | 0.72 | -0.027 | 0.046 | 0.55 | 0.77 |
|  |  | free cholesterol | -0.025 | 0.036 | 0.49 | 0.73 | -0.053 | 0.046 | 0.25 | 0.56 |
|  |  | triglycerides | -0.026 | 0.036 | 0.46 | 0.73 | -0.059 | 0.047 | 0.21 | 0.56 |
|  | XL | particles | -0.038 | 0.035 | 0.28 | 0.72 | -0.074 | 0.045 | 0.10 | 0.56 |
|  |  | lipid | -0.035 | 0.035 | 0.31 | 0.72 | -0.068 | 0.044 | 0.13 | 0.56 |
|  |  | phospholipid | -0.038 | 0.035 | 0.29 | 0.72 | -0.074 | 0.046 | 0.11 | 0.56 |
|  |  | cholesterol | -0.034 | 0.036 | 0.34 | 0.72 | -0.056 | 0.046 | 0.22 | 0.56 |
|  |  | cholesteryl ester | -0.033 | 0.035 | 0.35 | 0.72 | -0.044 | 0.045 | 0.33 | 0.57 |
|  |  | free cholesterol | -0.037 | 0.036 | 0.30 | 0.72 | -0.047 | 0.044 | 0.28 | 0.56 |
|  |  | triglycerides | -0.036 | 0.035 | 0.29 | 0.72 | -0.073 | 0.044 | 0.10 | 0.56 |
|  | L | particles | -0.039 | 0.034 | 0.25 | 0.72 | -0.064 | 0.041 | 0.12 | 0.56 |
|  |  | lipid | -0.035 | 0.033 | 0.28 | 0.72 | -0.057 | 0.041 | 0.16 | 0.56 |
|  |  | phospholipid | -0.039 | 0.034 | 0.25 | 0.72 | -0.060 | 0.042 | 0.15 | 0.56 |
|  |  | cholesterol | -0.034 | 0.034 | 0.31 | 0.72 | -0.051 | 0.042 | 0.23 | 0.56 |
|  |  | cholesteryl ester | -0.034 | 0.034 | 0.31 | 0.72 | -0.040 | 0.041 | 0.33 | 0.57 |
|  |  | free cholesterol | -0.035 | 0.035 | 0.32 | 0.72 | -0.062 | 0.043 | 0.15 | 0.56 |
|  |  | triglycerides | -0.038 | 0.033 | 0.26 | 0.72 | -0.062 | 0.041 | 0.13 | 0.56 |
|  | M | particles | -0.040 | 0.033 | 0.22 | 0.72 | -0.051 | 0.040 | 0.20 | 0.56 |
|  |  | lipid | -0.037 | 0.032 | 0.24 | 0.72 | -0.046 | 0.039 | 0.24 | 0.56 |
|  |  | phospholipid | -0.041 | 0.033 | 0.21 | 0.72 | -0.046 | 0.040 | 0.25 | 0.56 |
|  |  | cholesterol | -0.033 | 0.034 | 0.33 | 0.72 | -0.029 | 0.041 | 0.48 | 0.71 |
|  |  | cholesteryl ester | -0.029 | 0.035 | 0.41 | 0.72 | -0.010 | 0.042 | 0.80 | 0.92 |
|  |  | free cholesterol | -0.037 | 0.033 | 0.26 | 0.72 | -0.046 | 0.040 | 0.25 | 0.56 |
|  |  | triglycerides | -0.041 | 0.032 | 0.20 | 0.72 | -0.055 | 0.039 | 0.17 | 0.56 |
|  | S | particles | -0.043 | 0.033 | 0.19 | 0.72 | -0.013 | 0.040 | 0.75 | 0.90 |
|  |  | lipid | -0.041 | 0.033 | 0.21 | 0.72 | -0.009 | 0.040 | 0.82 | 0.93 |
|  |  | phospholipid | -0.039 | 0.033 | 0.24 | 0.72 | -0.008 | 0.040 | 0.85 | 0.94 |
|  |  | cholesterol | -0.041 | 0.036 | 0.25 | 0.72 | 0.017 | 0.044 | 0.70 | 0.87 |
|  |  | cholesteryl ester | -0.038 | 0.037 | 0.30 | 0.72 | 0.032 | 0.046 | 0.48 | 0.71 |
|  |  | free cholesterol | -0.040 | 0.034 | 0.24 | 0.72 | -0.003 | 0.041 | 0.95 | 0.97 |
|  |  | triglycerides | -0.044 | 0.032 | 0.17 | 0.72 | -0.025 | 0.038 | 0.51 | 0.73 |
|  | XS | particles | -0.030 | 0.036 | 0.41 | 0.72 | 0.031 | 0.046 | 0.50 | 0.72 |
|  |  | lipid | -0.023 | 0.036 | 0.52 | 0.74 | 0.039 | 0.047 | 0.41 | 0.65 |
|  |  | phospholipid | -0.025 | 0.037 | 0.50 | 0.73 | 0.054 | 0.048 | 0.26 | 0.56 |
|  |  | cholesterol | -0.020 | 0.037 | 0.60 | 0.78 | 0.055 | 0.048 | 0.26 | 0.56 |
|  |  | cholesteryl ester | -0.014 | 0.037 | 0.72 | 0.84 | 0.049 | 0.049 | 0.32 | 0.57 |
|  |  | free cholesterol | -0.035 | 0.037 | 0.35 | 0.72 | 0.062 | 0.047 | 0.19 | 0.56 |
|  |  | triglycerides | -0.030 | 0.032 | 0.36 | 0.72 | -0.010 | 0.041 | 0.81 | 0.92 |
| IDL |  | particles | -0.027 | 0.037 | 0.47 | 0.73 | 0.060 | 0.049 | 0.23 | 0.56 |
|  |  | lipid | -0.020 | 0.037 | 0.59 | 0.78 | 0.069 | 0.050 | 0.17 | 0.56 |
|  |  | phospholipid | -0.017 | 0.037 | 0.64 | 0.78 | 0.070 | 0.050 | 0.16 | 0.56 |
|  |  | cholesterol | -0.019 | 0.037 | 0.61 | 0.78 | 0.065 | 0.050 | 0.19 | 0.56 |
|  |  | cholesteryl ester | -0.022 | 0.037 | 0.56 | 0.76 | 0.055 | 0.050 | 0.27 | 0.56 |
|  |  | free cholesterol | -0.018 | 0.037 | 0.63 | 0.78 | 0.080 | 0.050 | 0.11 | 0.56 |
|  |  | triglycerides | -0.001 | 0.033 | 0.97 | 0.97 | 0.055 | 0.044 | 0.22 | 0.56 |
| LDL | L | particles | -0.036 | 0.037 | 0.34 | 0.72 | 0.055 | 0.049 | 0.26 | 0.56 |
|  |  | lipid | -0.028 | 0.037 | 0.45 | 0.73 | 0.063 | 0.050 | 0.20 | 0.56 |
|  |  | phospholipid | -0.030 | 0.037 | 0.42 | 0.72 | 0.054 | 0.049 | 0.28 | 0.56 |
|  |  | cholesterol | -0.032 | 0.037 | 0.38 | 0.72 | 0.060 | 0.050 | 0.23 | 0.56 |
|  |  | cholesteryl ester | -0.036 | 0.037 | 0.33 | 0.72 | 0.052 | 0.050 | 0.29 | 0.56 |
|  |  | free cholesterol | -0.025 | 0.037 | 0.49 | 0.73 | 0.072 | 0.050 | 0.15 | 0.56 |
|  |  | triglycerides | 0.002 | 0.034 | 0.95 | 0.97 | 0.077 | 0.046 | 0.09 | 0.56 |
|  | M | particles | -0.046 | 0.037 | 0.22 | 0.72 | 0.052 | 0.049 | 0.28 | 0.56 |
|  |  | lipid | -0.040 | 0.037 | 0.28 | 0.72 | 0.058 | 0.050 | 0.24 | 0.56 |
|  |  | phospholipid | -0.038 | 0.037 | 0.30 | 0.72 | 0.040 | 0.050 | 0.42 | 0.66 |
|  |  | cholesterol | -0.044 | 0.037 | 0.23 | 0.72 | 0.057 | 0.049 | 0.24 | 0.56 |
|  |  | cholesteryl ester | -0.045 | 0.037 | 0.22 | 0.72 | 0.055 | 0.049 | 0.26 | 0.56 |
|  |  | free cholesterol | -0.047 | 0.037 | 0.21 | 0.72 | 0.059 | 0.049 | 0.23 | 0.56 |
|  |  | triglycerides | -0.002 | 0.033 | 0.95 | 0.97 | 0.075 | 0.047 | 0.11 | 0.56 |
|  | S  XL | particles | -0.047 | 0.037 | 0.20 | 0.72 | 0.048 | 0.048 | 0.32 | 0.57 |
|  |  | lipid | -0.043 | 0.037 | 0.24 | 0.72 | 0.052 | 0.049 | 0.29 | 0.56 |
|  |  | phospholipid | -0.040 | 0.037 | 0.27 | 0.72 | 0.036 | 0.049 | 0.46 | 0.70 |
|  |  | cholesterol | -0.047 | 0.037 | 0.20 | 0.72 | 0.057 | 0.049 | 0.25 | 0.56 |
|  |  | cholesteryl ester | -0.047 | 0.037 | 0.21 | 0.72 | 0.055 | 0.049 | 0.26 | 0.56 |
|  |  | free cholesterol | -0.052 | 0.037 | 0.16 | 0.72 | 0.058 | 0.049 | 0.23 | 0.56 |
|  |  | triglycerides | -0.013 | 0.035 | 0.71 | 0.83 | 0.025 | 0.047 | 0.60 | 0.80 |
| HDL |  | particles | 0.048 | 0.025 | 0.06 | 0.72 | 0.044 | 0.033 | 0.18 | 0.56 |
|  |  | lipid | 0.042 | 0.025 | 0.10 | 0.72 | 0.039 | 0.033 | 0.23 | 0.56 |
|  |  | phospholipid | 0.054 | 0.025 | 0.03 | 0.52 | 0.040 | 0.032 | 0.21 | 0.56 |
|  |  | cholesterol | 0.032 | 0.026 | 0.22 | 0.72 | 0.043 | 0.034 | 0.21 | 0.56 |
|  |  | cholesteryl ester | 0.030 | 0.027 | 0.26 | 0.72 | 0.046 | 0.035 | 0.19 | 0.56 |
|  |  | free cholesterol | 0.041 | 0.026 | 0.11 | 0.72 | 0.038 | 0.033 | 0.25 | 0.56 |
|  |  | triglycerides | 0.017 | 0.037 | 0.63 | 0.78 | 0.002 | 0.051 | 0.97 | 0.98 |
|  | L | particles | 0.066 | 0.029 | 0.02 | 0.45 | 0.049 | 0.035 | 0.17 | 0.56 |
|  |  | lipid | 0.064 | 0.028 | 0.02 | 0.45 | 0.049 | 0.035 | 0.17 | 0.56 |
|  |  | phospholipid | 0.063 | 0.030 | 0.03 | 0.52 | 0.047 | 0.037 | 0.19 | 0.56 |
|  |  | cholesterol | 0.065 | 0.027 | 0.02 | 0.45 | 0.043 | 0.034 | 0.21 | 0.56 |
|  |  | cholesteryl ester | 0.066 | 0.027 | 0.02 | 0.45 | 0.043 | 0.034 | 0.21 | 0.56 |
|  |  | free cholesterol | 0.062 | 0.027 | 0.02 | 0.45 | 0.044 | 0.033 | 0.19 | 0.56 |
|  |  | triglycerides | 0.054 | 0.033 | 0.10 | 0.72 | 0.095 | 0.042 | 0.02 | 0.56 |
|  | M | particles | 0.040 | 0.037 | 0.28 | 0.72 | -0.013 | 0.045 | 0.78 | 0.91 |
|  |  | lipid | 0.034 | 0.037 | 0.36 | 0.72 | -0.008 | 0.046 | 0.86 | 0.94 |
|  |  | phospholipid | 0.042 | 0.036 | 0.25 | 0.72 | 0.004 | 0.045 | 0.94 | 0.97 |
|  |  | cholesterol | 0.030 | 0.037 | 0.42 | 0.72 | -0.017 | 0.046 | 0.71 | 0.88 |
|  |  | cholesteryl ester | 0.029 | 0.037 | 0.44 | 0.73 | -0.024 | 0.046 | 0.61 | 0.80 |
|  |  | free cholesterol | 0.036 | 0.037 | 0.32 | 0.72 | 0.005 | 0.045 | 0.92 | 0.97 |
|  |  | triglycerides | 0.028 | 0.036 | 0.43 | 0.73 | -0.023 | 0.043 | 0.59 | 0.79 |
|  | S | particles | -0.001 | 0.038 | 0.97 | 0.97 | -0.004 | 0.052 | 0.93 | 0.97 |
|  |  | lipid | -0.001 | 0.038 | 0.97 | 0.97 | 0.010 | 0.056 | 0.86 | 0.94 |
|  |  | phospholipid | 0.033 | 0.038 | 0.39 | 0.72 | -0.031 | 0.054 | 0.56 | 0.77 |
|  |  | cholesterol | -0.038 | 0.038 | 0.32 | 0.72 | 0.048 | 0.051 | 0.35 | 0.58 |
|  |  | cholesteryl ester | -0.041 | 0.038 | 0.28 | 0.72 | 0.055 | 0.050 | 0.27 | 0.56 |
|  |  | free cholesterol | -0.005 | 0.038 | 0.89 | 0.97 | -0.003 | 0.052 | 0.95 | 0.97 |
|  |  | triglycerides | -0.015 | 0.033 | 0.66 | 0.79 | 0.014 | 0.044 | 0.74 | 0.90 |
| VLDL diameter | | | -0.031 | 0.032 | 0.33 | 0.72 | -0.077 | 0.039 | 0.05 | 0.56 |
| LDL diameter | | | 0.088 | 0.032 | 0.01 | 0.45 | 0.041 | 0.046 | 0.38 | 0.63 |
| HDL diameter | | | 0.059 | 0.024 | 0.01 | 0.45 | 0.045 | 0.032 | 0.16 | 0.56 |
| Cholesterol | serum total | | -0.026 | 0.037 | 0.48 | 0.73 | 0.062 | 0.050 | 0.22 | 0.56 |
|  | VLDL | | -0.031 | 0.035 | 0.38 | 0.72 | -0.009 | 0.043 | 0.83 | 0.93 |
|  | remnant | | -0.031 | 0.037 | 0.40 | 0.72 | 0.021 | 0.048 | 0.65 | 0.82 |
|  | LDL | | -0.036 | 0.037 | 0.33 | 0.72 | 0.061 | 0.050 | 0.22 | 0.56 |
|  | HDL | | 0.045 | 0.031 | 0.14 | 0.72 | 0.044 | 0.039 | 0.26 | 0.56 |
|  | HDL2 | | 0.050 | 0.031 | 0.10 | 0.72 | 0.039 | 0.039 | 0.32 | 0.57 |
|  | HDL3 | | -0.007 | 0.034 | 0.83 | 0.93 | 0.093 | 0.044 | 0.03 | 0.56 |
|  | esterified | | -0.034 | 0.037 | 0.36 | 0.72 | 0.056 | 0.050 | 0.26 | 0.56 |
|  | free | | -0.007 | 0.037 | 0.85 | 0.94 | 0.070 | 0.050 | 0.16 | 0.56 |
| Triglycerides | serum total | | -0.028 | 0.032 | 0.39 | 0.72 | -0.037 | 0.040 | 0.35 | 0.58 |
|  | VLDL | | -0.036 | 0.032 | 0.26 | 0.72 | -0.050 | 0.039 | 0.20 | 0.56 |
|  | LDL | | -0.003 | 0.033 | 0.93 | 0.97 | 0.063 | 0.046 | 0.17 | 0.56 |
|  | HDL | | 0.022 | 0.037 | 0.55 | 0.75 | 0.033 | 0.043 | 0.44 | 0.68 |
| Total phosphoglycerides | | | 0.019 | 0.036 | 0.61 | 0.78 | 0.008 | 0.045 | 0.87 | 0.94 |
| Phosphatidylcholine | | | 0.018 | 0.036 | 0.63 | 0.78 | 0.023 | 0.045 | 0.61 | 0.80 |
| Sphingomyelins | | | 0.023 | 0.034 | 0.51 | 0.73 | 0.076 | 0.046 | 0.10 | 0.56 |
| Total cholines | | | 0.024 | 0.036 | 0.50 | 0.73 | 0.022 | 0.045 | 0.62 | 0.80 |
| Apolipoprotein A-I | | | 0.013 | 0.035 | 0.71 | 0.83 | 0.037 | 0.045 | 0.41 | 0.65 |
| Apolipoprotein B | | | -0.044 | 0.036 | 0.23 | 0.72 | 0.004 | 0.046 | 0.94 | 0.97 |
| Apo-B / apoA-I | | | -0.056 | 0.031 | 0.07 | 0.72 | 0.003 | 0.041 | 0.93 | 0.97 |
| Fatty acids | total | | -0.003 | 0.037 | 0.93 | 0.97 | -0.025 | 0.046 | 0.59 | 0.79 |
|  | unsaturated | | -0.019 | 0.035 | 0.59 | 0.78 | 0.023 | 0.048 | 0.64 | 0.82 |
|  | DHA (docosahexaenoic acid) | | -0.018 | 0.035 | 0.61 | 0.78 | 0.035 | 0.044 | 0.44 | 0.68 |
|  | LA (linoleic acid) | | -0.023 | 0.038 | 0.55 | 0.75 | -0.002 | 0.052 | 0.97 | 0.98 |
|  | omega-3 | | -0.026 | 0.036 | 0.47 | 0.73 | 0.012 | 0.044 | 0.78 | 0.91 |
|  | omega-6 | | -0.012 | 0.038 | 0.76 | 0.87 | -0.001 | 0.049 | 0.98 | 0.99 |
|  | PUFA (polyunsaturated) | | -0.018 | 0.038 | 0.64 | 0.78 | 0.001 | 0.048 | 0.99 | 0.99 |
|  | MUFA (monounsaturated) | | -0.002 | 0.035 | 0.95 | 0.97 | -0.026 | 0.045 | 0.56 | 0.77 |
|  | SAFA (saturated) | | 0.009 | 0.037 | 0.81 | 0.91 | -0.032 | 0.046 | 0.49 | 0.72 |
|  | DHA% | | -0.021 | 0.033 | 0.54 | 0.75 | 0.046 | 0.045 | 0.31 | 0.57 |
|  | LA% | | -0.017 | 0.034 | 0.63 | 0.78 | 0.041 | 0.041 | 0.32 | 0.57 |
|  | omega-3% | | -0.034 | 0.034 | 0.32 | 0.72 | 0.033 | 0.046 | 0.47 | 0.71 |
|  | omega-6% | | 0.005 | 0.034 | 0.88 | 0.97 | 0.043 | 0.042 | 0.31 | 0.57 |
|  | PUFA% | | 0.004 | 0.033 | 0.90 | 0.97 | 0.050 | 0.044 | 0.25 | 0.56 |
|  | MUFA% | | -0.014 | 0.032 | 0.65 | 0.79 | -0.017 | 0.039 | 0.66 | 0.83 |
|  | SAFA% | | 0.046 | 0.037 | 0.22 | 0.72 | -0.044 | 0.046 | 0.35 | 0.58 |
| Glucose | | | -0.011 | 0.036 | 0.76 | 0.87 | -0.047 | 0.048 | 0.33 | 0.57 |
| Lactate | | | -0.043 | 0.036 | 0.23 | 0.72 | -0.008 | 0.050 | 0.87 | 0.94 |
| Pyruvate | | | -0.007 | 0.035 | 0.85 | 0.94 | -0.050 | 0.049 | 0.31 | 0.57 |
| Citrate | | | -0.025 | 0.036 | 0.49 | 0.73 | -0.011 | 0.046 | 0.81 | 0.92 |
| Glycerol | | | 0.004 | 0.041 | 0.93 | 0.97 | 0.031 | 0.053 | 0.56 | 0.77 |
| Alanine | | | 0.014 | 0.037 | 0.71 | 0.83 | -0.021 | 0.047 | 0.65 | 0.82 |
| Glutamine | | | -0.049 | 0.038 | 0.20 | 0.72 | 0.016 | 0.049 | 0.74 | 0.90 |
| Glycine | | | -0.024 | 0.032 | 0.46 | 0.73 | 0.036 | 0.043 | 0.40 | 0.65 |
| Histidine | | | -0.047 | 0.039 | 0.23 | 0.72 | 0.015 | 0.050 | 0.76 | 0.90 |
| Isoleucine | | | 0.010 | 0.037 | 0.79 | 0.90 | -0.052 | 0.045 | 0.25 | 0.56 |
| Leucine | | | 0.003 | 0.037 | 0.94 | 0.97 | -0.058 | 0.046 | 0.21 | 0.56 |
| Valine | | | 0.025 | 0.037 | 0.49 | 0.73 | -0.053 | 0.045 | 0.24 | 0.56 |
| Phenylalanine | | | 0.023 | 0.039 | 0.55 | 0.75 | 0.031 | 0.046 | 0.50 | 0.72 |
| Tyrosine | | | 0.048 | 0.038 | 0.20 | 0.72 | -0.027 | 0.048 | 0.57 | 0.78 |
| Acetate | | | 0.066 | 0.038 | 0.08 | 0.72 | -0.008 | 0.051 | 0.87 | 0.94 |
| Acetoacetate | | | 0.002 | 0.041 | 0.96 | 0.97 | 0.016 | 0.049 | 0.75 | 0.90 |
| 3-hydroxybutyrate | | | -0.027 | 0.040 | 0.50 | 0.73 | 0.013 | 0.047 | 0.79 | 0.92 |
| Creatinine | | | -0.015 | 0.032 | 0.64 | 0.78 | 0.093 | 0.052 | 0.07 | 0.56 |
| Albumin | | | -0.025 | 0.040 | 0.53 | 0.75 | 0.042 | 0.049 | 0.39 | 0.64 |
| GlycA (glycoprotein acetylation) | | | 0.001 | 0.037 | 0.97 | 0.97 | -0.049 | 0.046 | 0.29 | 0.56 |

| **ABL categorical (no and mild vs. moderate and severe)** | | | | | | | | | | |
| --- | --- | --- | --- | --- | --- | --- | --- | --- | --- | --- |
|  | | | ≥ 20 teeth | | | | < 20 teeth | | | |
| **Metabolite** | | | Beta | SE | p | FDR | Beta | SE | p | FDR |
| VLDL | XXL | particles | -0.264 | 0.092 | 0.004 | **0.04** | -0.156 | 0.098 | 0.11 | 0.37 |
|  |  | lipid | -0.261 | 0.092 | 0.005 | **0.04** | -0.148 | 0.097 | 0.13 | 0.39 |
|  |  | phospholipid | -0.267 | 0.092 | 0.004 | **0.04** | -0.161 | 0.099 | 0.10 | 0.36 |
|  |  | cholesterol | -0.249 | 0.093 | 0.01 | **0.04** | -0.122 | 0.099 | 0.22 | 0.48 |
|  |  | cholesteryl ester | -0.232 | 0.094 | 0.01 | **0.04** | -0.063 | 0.096 | 0.52 | 0.75 |
|  |  | free cholesterol | -0.259 | 0.092 | 0.01 | **0.04** | -0.152 | 0.097 | 0.12 | 0.37 |
|  |  | triglycerides | -0.264 | 0.092 | 0.004 | **0.04** | -0.162 | 0.098 | 0.10 | 0.36 |
|  | XL | particles | -0.259 | 0.090 | 0.004 | **0.04** | -0.174 | 0.094 | 0.07 | 0.35 |
|  |  | lipid | -0.257 | 0.089 | 0.004 | **0.04** | -0.160 | 0.093 | 0.09 | 0.36 |
|  |  | phospholipid | -0.259 | 0.091 | 0.004 | **0.04** | -0.173 | 0.097 | 0.08 | 0.36 |
|  |  | cholesterol | -0.250 | 0.091 | 0.01 | **0.04** | -0.132 | 0.096 | 0.17 | 0.45 |
|  |  | cholesteryl ester | -0.241 | 0.091 | 0.01 | **0.04** | -0.106 | 0.094 | 0.26 | 0.54 |
|  |  | free cholesterol | -0.263 | 0.093 | 0.005 | **0.04** | -0.130 | 0.092 | 0.16 | 0.43 |
|  |  | triglycerides | -0.258 | 0.089 | 0.004 | **0.04** | -0.174 | 0.092 | 0.06 | 0.35 |
|  | L | particles | -0.256 | 0.088 | 0.004 | **0.04** | -0.141 | 0.087 | 0.10 | 0.36 |
|  |  | lipid | -0.250 | 0.085 | 0.003 | **0.04** | -0.116 | 0.085 | 0.18 | 0.46 |
|  |  | phospholipid | -0.257 | 0.087 | 0.003 | **0.04** | -0.130 | 0.087 | 0.14 | 0.39 |
|  |  | cholesterol | -0.243 | 0.088 | 0.01 | **0.04** | -0.108 | 0.088 | 0.22 | 0.48 |
|  |  | cholesteryl ester | -0.228 | 0.086 | 0.01 | **0.04** | -0.081 | 0.085 | 0.34 | 0.62 |
|  |  | free cholesterol | -0.253 | 0.089 | 0.004 | **0.04** | -0.139 | 0.090 | 0.12 | 0.37 |
|  |  | triglycerides | -0.255 | 0.085 | **0.0027** | **0.04** | -0.133 | 0.085 | 0.12 | 0.37 |
|  | M | particles | -0.237 | 0.084 | 0.005 | **0.04** | -0.105 | 0.083 | 0.21 | 0.48 |
|  |  | lipid | -0.230 | 0.082 | 0.01 | **0.04** | -0.076 | 0.083 | 0.36 | 0.64 |
|  |  | phospholipid | -0.235 | 0.084 | 0.01 | **0.04** | -0.085 | 0.084 | 0.31 | 0.57 |
|  |  | cholesterol | -0.212 | 0.087 | 0.02 | 0.06 | -0.040 | 0.085 | 0.64 | 0.77 |
|  |  | cholesteryl ester | -0.183 | 0.090 | 0.04 | 0.11 | 0.002 | 0.088 | 0.98 | 0.99 |
|  |  | free cholesterol | -0.236 | 0.085 | 0.01 | **0.04** | -0.085 | 0.084 | 0.31 | 0.57 |
|  |  | triglycerides | -0.241 | 0.082 | 0.003 | **0.04** | -0.105 | 0.083 | 0.20 | 0.46 |
|  | S | particles | -0.203 | 0.085 | 0.02 | 0.06 | 0.012 | 0.083 | 0.89 | 0.92 |
|  |  | lipid | -0.195 | 0.084 | 0.02 | 0.06 | 0.033 | 0.084 | 0.69 | 0.80 |
|  |  | phospholipid | -0.192 | 0.085 | 0.02 | 0.06 | 0.033 | 0.084 | 0.69 | 0.80 |
|  |  | cholesterol | -0.156 | 0.092 | 0.09 | 0.21 | 0.108 | 0.092 | 0.24 | 0.50 |
|  |  | cholesteryl ester | -0.122 | 0.094 | 0.20 | 0.39 | 0.140 | 0.095 | 0.14 | 0.39 |
|  |  | free cholesterol | -0.196 | 0.087 | 0.02 | 0.06 | 0.053 | 0.086 | 0.54 | 0.76 |
|  |  | triglycerides | -0.211 | 0.082 | 0.01 | **0.04** | -0.028 | 0.080 | 0.73 | 0.81 |
|  | XS | particles | -0.079 | 0.094 | 0.40 | 0.68 | 0.153 | 0.097 | 0.12 | 0.37 |
|  |  | lipid | -0.059 | 0.094 | 0.53 | 0.83 | 0.169 | 0.098 | 0.09 | 0.36 |
|  |  | phospholipid | -0.021 | 0.095 | 0.82 | 0.99 | 0.190 | 0.100 | 0.06 | 0.35 |
|  |  | cholesterol | -0.012 | 0.096 | 0.90 | 0.99 | 0.192 | 0.101 | 0.06 | 0.35 |
|  |  | cholesteryl ester | 0.003 | 0.096 | 0.97 | 0.995 | 0.176 | 0.102 | 0.09 | 0.36 |
|  |  | free cholesterol | -0.048 | 0.095 | 0.61 | 0.88 | 0.211 | 0.099 | 0.03 | 0.35 |
|  |  | triglycerides | -0.170 | 0.083 | 0.04 | 0.11 | 0.053 | 0.086 | 0.54 | 0.76 |
| IDL |  | particles | -0.007 | 0.096 | 0.95 | 0.995 | 0.189 | 0.103 | 0.07 | 0.35 |
|  |  | lipid | 0.011 | 0.095 | 0.91 | 0.99 | 0.206 | 0.105 | 0.05 | 0.35 |
|  |  | phospholipid | 0.035 | 0.095 | 0.71 | 0.96 | 0.209 | 0.104 | 0.04 | 0.35 |
|  |  | cholesterol | 0.017 | 0.096 | 0.85 | 0.99 | 0.190 | 0.105 | 0.07 | 0.35 |
|  |  | cholesteryl ester | -0.002 | 0.096 | 0.98 | 0.995 | 0.166 | 0.104 | 0.11 | 0.37 |
|  |  | free cholesterol | 0.057 | 0.095 | 0.55 | 0.83 | 0.226 | 0.104 | 0.03 | 0.35 |
|  |  | triglycerides | -0.051 | 0.085 | 0.55 | 0.83 | 0.195 | 0.092 | 0.04 | 0.35 |
| LDL | L | particles | -0.015 | 0.096 | 0.88 | 0.99 | 0.179 | 0.102 | 0.08 | 0.36 |
|  |  | lipid | 0.003 | 0.095 | 0.98 | 0.995 | 0.197 | 0.105 | 0.06 | 0.35 |
|  |  | phospholipid | -0.001 | 0.096 | 0.99 | 0.995 | 0.171 | 0.104 | 0.10 | 0.36 |
|  |  | cholesterol | 0.002 | 0.095 | 0.99 | 0.995 | 0.188 | 0.105 | 0.07 | 0.35 |
|  |  | cholesteryl ester | -0.016 | 0.096 | 0.87 | 0.99 | 0.172 | 0.104 | 0.10 | 0.36 |
|  |  | free cholesterol | 0.047 | 0.095 | 0.62 | 0.88 | 0.212 | 0.104 | 0.04 | 0.35 |
|  |  | triglycerides | -0.009 | 0.086 | 0.92 | 0.99 | 0.238 | 0.095 | 0.01 | 0.35 |
|  | M | particles | -0.027 | 0.096 | 0.78 | 0.99 | 0.182 | 0.102 | 0.07 | 0.35 |
|  |  | lipid | -0.011 | 0.095 | 0.91 | 0.99 | 0.194 | 0.104 | 0.06 | 0.35 |
|  |  | phospholipid | -0.031 | 0.096 | 0.75 | 0.97 | 0.146 | 0.104 | 0.16 | 0.43 |
|  |  | cholesterol | -0.013 | 0.095 | 0.89 | 0.99 | 0.191 | 0.103 | 0.06 | 0.35 |
|  |  | cholesteryl ester | -0.020 | 0.095 | 0.83 | 0.99 | 0.187 | 0.103 | 0.07 | 0.35 |
|  |  | free cholesterol | 0.002 | 0.096 | 0.98 | 0.995 | 0.190 | 0.104 | 0.07 | 0.35 |
|  |  | triglycerides | 0.008 | 0.086 | 0.92 | 0.99 | 0.245 | 0.098 | 0.01 | 0.35 |
|  | S | particles | -0.034 | 0.095 | 0.72 | 0.96 | 0.170 | 0.101 | 0.09 | 0.36 |
|  |  | lipid | -0.019 | 0.095 | 0.84 | 0.99 | 0.181 | 0.103 | 0.08 | 0.36 |
|  |  | phospholipid | -0.038 | 0.095 | 0.69 | 0.94 | 0.131 | 0.103 | 0.20 | 0.46 |
|  |  | cholesterol | -0.010 | 0.095 | 0.91 | 0.99 | 0.190 | 0.102 | 0.06 | 0.35 |
|  |  | cholesteryl ester | -0.012 | 0.095 | 0.90 | 0.99 | 0.187 | 0.102 | 0.07 | 0.35 |
|  |  | free cholesterol | -0.015 | 0.095 | 0.87 | 0.99 | 0.186 | 0.102 | 0.07 | 0.35 |
|  |  | triglycerides | -0.117 | 0.090 | 0.19 | 0.38 | 0.127 | 0.099 | 0.20 | 0.46 |
| HDL | XL | particles | 0.154 | 0.065 | 0.02 | 0.06 | 0.055 | 0.069 | 0.43 | 0.70 |
|  |  | lipid | 0.137 | 0.065 | 0.03 | 0.09 | 0.040 | 0.069 | 0.56 | 0.76 |
|  |  | phospholipid | 0.167 | 0.063 | 0.01 | **0.04** | 0.047 | 0.067 | 0.48 | 0.73 |
|  |  | cholesterol | 0.123 | 0.068 | 0.07 | 0.18 | 0.050 | 0.072 | 0.49 | 0.73 |
|  |  | cholesteryl ester | 0.117 | 0.070 | 0.09 | 0.21 | 0.057 | 0.073 | 0.44 | 0.70 |
|  |  | free cholesterol | 0.151 | 0.066 | 0.02 | 0.06 | 0.043 | 0.070 | 0.53 | 0.76 |
|  |  | triglycerides | -0.068 | 0.094 | 0.47 | 0.75 | -0.008 | 0.106 | 0.94 | 0.96 |
|  | L | particles | 0.169 | 0.074 | 0.02 | 0.06 | 0.058 | 0.075 | 0.44 | 0.70 |
|  |  | lipid | 0.175 | 0.072 | 0.02 | 0.06 | 0.057 | 0.074 | 0.44 | 0.70 |
|  |  | phospholipid | 0.172 | 0.076 | 0.02 | 0.06 | 0.058 | 0.077 | 0.45 | 0.71 |
|  |  | cholesterol | 0.172 | 0.069 | 0.01 | **0.04** | 0.040 | 0.071 | 0.57 | 0.76 |
|  |  | cholesteryl ester | 0.171 | 0.070 | 0.01 | **0.04** | 0.039 | 0.071 | 0.59 | 0.77 |
|  |  | free cholesterol | 0.173 | 0.069 | 0.01 | **0.04** | 0.048 | 0.070 | 0.49 | 0.73 |
|  |  | triglycerides | 0.079 | 0.085 | 0.35 | 0.60 | 0.178 | 0.088 | 0.04 | 0.35 |
|  | M | particles | 0.010 | 0.095 | 0.92 | 0.99 | -0.082 | 0.095 | 0.39 | 0.67 |
|  |  | lipid | 0.019 | 0.095 | 0.84 | 0.99 | -0.071 | 0.097 | 0.47 | 0.73 |
|  |  | phospholipid | 0.031 | 0.094 | 0.74 | 0.97 | -0.037 | 0.094 | 0.69 | 0.80 |
|  |  | cholesterol | 0.033 | 0.095 | 0.73 | 0.96 | -0.099 | 0.097 | 0.31 | 0.57 |
|  |  | cholesteryl ester | 0.027 | 0.096 | 0.78 | 0.99 | -0.119 | 0.097 | 0.22 | 0.48 |
|  |  | free cholesterol | 0.053 | 0.094 | 0.57 | 0.85 | -0.031 | 0.095 | 0.75 | 0.82 |
|  |  | triglycerides | -0.136 | 0.093 | 0.14 | 0.30 | -0.033 | 0.090 | 0.71 | 0.80 |
|  | S | particles | -0.090 | 0.097 | 0.35 | 0.60 | 0.002 | 0.109 | 0.99 | 0.99 |
|  |  | lipid | -0.078 | 0.097 | 0.42 | 0.68 | 0.031 | 0.118 | 0.80 | 0.85 |
|  |  | phospholipid | -0.096 | 0.097 | 0.32 | 0.57 | -0.092 | 0.113 | 0.42 | 0.70 |
|  |  | cholesterol | 0.013 | 0.098 | 0.89 | 0.99 | 0.144 | 0.107 | 0.18 | 0.46 |
|  |  | cholesteryl ester | 0.050 | 0.097 | 0.61 | 0.88 | 0.168 | 0.104 | 0.11 | 0.37 |
|  |  | free cholesterol | -0.125 | 0.098 | 0.20 | 0.39 | -0.023 | 0.108 | 0.83 | 0.88 |
|  |  | triglycerides | -0.201 | 0.086 | 0.02 | 0.06 | 0.085 | 0.093 | 0.36 | 0.64 |
| VLDL diameter | | | -0.244 | 0.081 | **0.0026** | **0.04** | -0.155 | 0.081 | 0.06 | 0.35 |
| LDL diameter | | | 0.115 | 0.082 | 0.16 | 0.33 | -0.015 | 0.098 | 0.87 | 0.90 |
| HDL diameter | | | 0.172 | 0.062 | 0.01 | **0.04** | 0.047 | 0.066 | 0.48 | 0.73 |
| Cholesterol | serum total | | -0.007 | 0.095 | 0.94 | 0.995 | 0.171 | 0.105 | 0.10 | 0.36 |
|  | VLDL | | -0.184 | 0.090 | 0.04 | 0.11 | 0.034 | 0.091 | 0.71 | 0.80 |
|  | remnant | | -0.113 | 0.095 | 0.23 | 0.43 | 0.106 | 0.100 | 0.29 | 0.57 |
|  | LDL | | -0.001 | 0.095 | 0.995 | 0.995 | 0.197 | 0.105 | 0.06 | 0.35 |
|  | HDL | | 0.161 | 0.079 | 0.04 | 0.11 | 0.059 | 0.083 | 0.47 | 0.73 |
|  | HDL2 | | 0.171 | 0.078 | 0.03 | 0.09 | 0.040 | 0.082 | 0.62 | 0.77 |
|  | HDL3 | | 0.036 | 0.088 | 0.68 | 0.94 | 0.224 | 0.092 | 0.02 | 0.35 |
|  | esterified | | -0.017 | 0.095 | 0.86 | 0.99 | 0.156 | 0.105 | 0.14 | 0.39 |
|  | free | | 0.010 | 0.095 | 0.92 | 0.99 | 0.192 | 0.104 | 0.07 | 0.35 |
| Triglycerides | serum total | | -0.222 | 0.083 | 0.01 | **0.04** | -0.047 | 0.084 | 0.58 | 0.77 |
|  | VLDL | | -0.236 | 0.081 | 0.004 | **0.04** | -0.083 | 0.082 | 0.31 | 0.57 |
|  | LDL | | -0.039 | 0.086 | 0.65 | 0.91 | 0.212 | 0.096 | 0.03 | 0.35 |
|  | HDL | | -0.139 | 0.095 | 0.14 | 0.30 | 0.083 | 0.091 | 0.36 | 0.64 |
| Total phosphoglycerides | | | -0.046 | 0.094 | 0.62 | 0.88 | 0.019 | 0.094 | 0.84 | 0.89 |
| Phosphatidylcholine | | | -0.045 | 0.094 | 0.63 | 0.89 | 0.054 | 0.095 | 0.57 | 0.76 |
| Sphingomyelins | | | 0.140 | 0.088 | 0.11 | 0.25 | 0.181 | 0.097 | 0.06 | 0.35 |
| Total cholines | | | 0.007 | 0.092 | 0.94 | 0.995 | 0.056 | 0.094 | 0.55 | 0.76 |
| Apolipoprotein A-I | | | 0.054 | 0.090 | 0.55 | 0.83 | 0.047 | 0.094 | 0.62 | 0.77 |
| Apolipoprotein B | | | -0.138 | 0.094 | 0.14 | 0.30 | 0.066 | 0.097 | 0.50 | 0.73 |
| Apo-B / apoA-I | | | -0.155 | 0.080 | 0.05 | 0.13 | 0.074 | 0.085 | 0.39 | 0.67 |
| Fatty acids | total | | -0.168 | 0.095 | 0.08 | 0.20 | -0.024 | 0.097 | 0.80 | 0.85 |
|  | unsaturated | | 0.137 | 0.089 | 0.12 | 0.27 | 0.026 | 0.101 | 0.79 | 0.85 |
|  | DHA (docosahexaenoic acid) | | -0.017 | 0.091 | 0.85 | 0.99 | 0.035 | 0.093 | 0.70 | 0.80 |
|  | LA (linoleic acid) | | -0.104 | 0.097 | 0.29 | 0.53 | 0.057 | 0.109 | 0.60 | 0.77 |
|  | omega-3 | | -0.049 | 0.092 | 0.60 | 0.88 | 0.001 | 0.093 | 0.99 | 0.99 |
|  | omega-6 | | -0.077 | 0.097 | 0.42 | 0.68 | 0.049 | 0.103 | 0.63 | 0.77 |
|  | PUFA (polyunsaturated) | | -0.079 | 0.097 | 0.42 | 0.68 | 0.040 | 0.100 | 0.69 | 0.80 |
|  | MUFA (monounsaturated) | | -0.207 | 0.090 | 0.02 | 0.06 | -0.030 | 0.093 | 0.75 | 0.82 |
|  | SAFA (saturated) | | -0.156 | 0.096 | 0.10 | 0.23 | -0.056 | 0.098 | 0.57 | 0.76 |
|  | DHA% | | 0.024 | 0.085 | 0.77 | 0.99 | 0.045 | 0.095 | 0.64 | 0.77 |
|  | LA% | | 0.126 | 0.087 | 0.15 | 0.31 | 0.130 | 0.086 | 0.13 | 0.39 |
|  | omega-3% | | -0.001 | 0.087 | 0.99 | 0.995 | 0.042 | 0.097 | 0.66 | 0.79 |
|  | omega-6% | | 0.207 | 0.087 | 0.02 | 0.06 | 0.116 | 0.088 | 0.19 | 0.46 |
|  | PUFA% | | 0.213 | 0.086 | 0.01 | **0.04** | 0.117 | 0.091 | 0.20 | 0.46 |
|  | MUFA% | | -0.219 | 0.082 | 0.01 | **0.04** | -0.039 | 0.081 | 0.63 | 0.77 |
|  | SAFA% | | -0.041 | 0.096 | 0.67 | 0.93 | -0.125 | 0.097 | 0.20 | 0.46 |
| Glucose | | | -0.110 | 0.092 | 0.23 | 0.43 | -0.121 | 0.101 | 0.23 | 0.49 |
| Lactate | | | -0.140 | 0.092 | 0.13 | 0.29 | 0.046 | 0.105 | 0.66 | 0.79 |
| Pyruvate | | | -0.075 | 0.092 | 0.41 | 0.68 | -0.111 | 0.102 | 0.28 | 0.57 |
| Citrate | | | 0.056 | 0.093 | 0.55 | 0.83 | -0.057 | 0.097 | 0.56 | 0.76 |
| Glycerol | | | 0.038 | 0.106 | 0.72 | 0.96 | 0.117 | 0.111 | 0.29 | 0.57 |
| Alanine | | | -0.117 | 0.094 | 0.22 | 0.42 | -0.010 | 0.098 | 0.92 | 0.94 |
| Glutamine | | | -0.096 | 0.099 | 0.33 | 0.58 | -0.020 | 0.102 | 0.85 | 0.89 |
| Glycine | | | -0.068 | 0.083 | 0.41 | 0.68 | 0.047 | 0.090 | 0.60 | 0.77 |
| Histidine | | | -0.168 | 0.100 | 0.09 | 0.21 | 0.038 | 0.105 | 0.72 | 0.81 |
| Isoleucine | | | -0.177 | 0.096 | 0.06 | 0.16 | -0.097 | 0.095 | 0.31 | 0.57 |
| Leucine | | | -0.207 | 0.096 | 0.03 | 0.09 | -0.119 | 0.098 | 0.23 | 0.49 |
| Valine | | | -0.095 | 0.096 | 0.32 | 0.57 | -0.100 | 0.095 | 0.30 | 0.57 |
| Phenylalanine | | | -0.121 | 0.099 | 0.22 | 0.42 | 0.067 | 0.097 | 0.49 | 0.73 |
| Tyrosine | | | -0.065 | 0.097 | 0.50 | 0.79 | -0.051 | 0.101 | 0.62 | 0.77 |
| Acetate | | | 0.195 | 0.099 | 0.05 | 0.13 | -0.032 | 0.108 | 0.77 | 0.84 |
| Acetoacetate | | | 0.157 | 0.105 | 0.14 | 0.30 | -0.066 | 0.103 | 0.52 | 0.75 |
| 3-hydroxybutyrate | | | 0.149 | 0.104 | 0.15 | 0.31 | -0.079 | 0.100 | 0.43 | 0.70 |
| Creatinine | | | 0.088 | 0.081 | 0.28 | 0.52 | 0.141 | 0.109 | 0.19 | 0.46 |
| Albumin | | | -0.028 | 0.102 | 0.79 | 0.99 | 0.049 | 0.102 | 0.63 | 0.77 |
| GlycA (glycoprotein acetylation) | | | -0.165 | 0.096 | 0.08 | 0.20 | -0.081 | 0.098 | 0.41 | 0.70 |

**APPENDIX TABLE 6.** Mean changes in metabolite levels during the 11-year follow-up in Health-2000. Means are calculated for subgroups with: 0-3 sextants with bleeding on probing; 4-6 sextants with bleeding on probing; 0-4 teeth with caries; ≥ 5 teeth with caries.

|  |  |  | **mean change in metabolite level during follow-up** | | | |
| --- | --- | --- | --- | --- | --- | --- |
|  |  |  | number of sextants with BOP: 0-3 | number of sextants with BOP: 4-6 | number of decayed teeth: 0-4 | number of decayed teeth: ≥ 5 |
| VLDL | XXL | particles | 0,105 | 0,090 | 0,102 | 0,045 |
|  |  | lipid | 0,104 | 0,098 | 0,103 | 0,070 |
|  |  | phospholipid | 0,105 | 0,089 | 0,103 | 0,021 |
|  |  | cholesterol | 0,123 | 0,080 | 0,113 | 0,048 |
|  |  | cholesteryl ester | 0,142 | 0,090 | 0,130 | 0,027 |
|  |  | free cholesterol | 0,095 | 0,095 | 0,098 | 0,092 |
|  |  | triglyceride | 0,101 | 0,089 | 0,098 | 0,056 |
|  | XL | particles | 0,102 | 0,083 | 0,098 | 0,168 |
|  |  | lipid | 0,104 | 0,096 | 0,104 | 0,177 |
|  |  | phospholipid | 0,113 | 0,082 | 0,106 | 0,112 |
|  |  | cholesterol | 0,107 | 0,076 | 0,100 | 0,096 |
|  |  | cholesteryl ester | 0,102 | 0,082 | 0,098 | 0,114 |
|  |  | free cholesterol | 0,115 | 0,084 | 0,108 | 0,080 |
|  |  | triglyceride | 0,099 | 0,091 | 0,098 | 0,203 |
|  | L | particles | 0,110 | 0,092 | 0,105 | 0,273 |
|  |  | lipid | 0,113 | 0,121 | 0,119 | 0,251 |
|  |  | phospholipid | 0,120 | 0,103 | 0,117 | 0,252 |
|  |  | cholesterol | 0,116 | 0,105 | 0,116 | 0,214 |
|  |  | cholesteryl ester | 0,116 | 0,104 | 0,115 | 0,195 |
|  |  | free cholesterol | 0,113 | 0,094 | 0,110 | 0,224 |
|  |  | triglyceride | 0,108 | 0,112 | 0,111 | 0,277 |
|  | M | particles | 0,118 | 0,104 | 0,116 | 0,289 |
|  |  | lipid | 0,126 | 0,131 | 0,131 | 0,256 |
|  |  | phospholipid | 0,132 | 0,116 | 0,130 | 0,250 |
|  |  | cholesterol | 0,161 | 0,119 | 0,152 | 0,191 |
|  |  | cholesteryl ester | 0,183 | 0,111 | 0,165 | 0,127 |
|  |  | free cholesterol | 0,129 | 0,117 | 0,129 | 0,256 |
|  |  | triglyceride | 0,104 | 0,120 | 0,111 | 0,296 |
|  | S | particles | 0,177 | 0,139 | 0,173 | 0,182 |
|  |  | lipid | 0,190 | 0,156 | 0,187 | 0,149 |
|  |  | phospholipid | 0,192 | 0,155 | 0,190 | 0,111 |
|  |  | cholesterol | 0,231 | 0,128 | 0,208 | -0,039 |
|  |  | cholesteryl ester | 0,240 | 0,109 | 0,206 | -0,108 |
|  |  | free cholesterol | 0,198 | 0,150 | 0,195 | 0,076 |
|  |  | triglyceride | 0,135 | 0,142 | 0,143 | 0,278 |
|  | XS | particles | 0,272 | 0,122 | 0,237 | -0,165 |
|  |  | lipid | 0,274 | 0,118 | 0,236 | -0,205 |
|  |  | phospholipid | 0,274 | 0,095 | 0,227 | -0,207 |
|  |  | cholesterol | 0,269 | 0,084 | 0,220 | -0,306 |
|  |  | cholesteryl ester | 0,272 | 0,074 | 0,217 | -0,331 |
|  |  | free cholesterol | 0,254 | 0,100 | 0,217 | -0,233 |
|  |  | triglyceride | 0,189 | 0,174 | 0,200 | 0,101 |
| IDL |  | particles | 0,268 | 0,061 | 0,212 | -0,254 |
|  |  | lipid | 0,262 | 0,053 | 0,203 | -0,289 |
|  |  | phospholipid | 0,247 | 0,052 | 0,193 | -0,313 |
|  |  | cholesterol | 0,248 | 0,040 | 0,187 | -0,281 |
|  |  | cholesteryl ester | 0,252 | 0,042 | 0,190 | -0,249 |
|  |  | free cholesterol | 0,233 | 0,034 | 0,177 | -0,327 |
|  |  | triglyceride | 0,234 | 0,194 | 0,244 | -0,085 |
| LDL | L | particles | 0,261 | 0,055 | 0,203 | -0,233 |
|  |  | lipid | 0,254 | 0,048 | 0,194 | -0,256 |
|  |  | phospholipid | 0,252 | 0,039 | 0,190 | -0,240 |
|  |  | cholesterol | 0,249 | 0,039 | 0,186 | -0,249 |
|  |  | cholesteryl ester | 0,253 | 0,043 | 0,190 | -0,228 |
|  |  | free cholesterol | 0,235 | 0,030 | 0,175 | -0,293 |
|  |  | triglyceride | 0,252 | 0,155 | 0,244 | -0,235 |
|  | M | particles | 0,260 | 0,066 | 0,206 | -0,180 |
|  |  | lipid | 0,256 | 0,062 | 0,200 | -0,184 |
|  |  | phospholipid | 0,262 | 0,056 | 0,201 | -0,106 |
|  |  | cholesterol | 0,246 | 0,052 | 0,189 | -0,202 |
|  |  | cholesteryl ester | 0,246 | 0,055 | 0,190 | -0,208 |
|  |  | free cholesterol | 0,246 | 0,039 | 0,185 | -0,174 |
|  |  | triglyceride | 0,259 | 0,173 | 0,256 | -0,219 |
|  | S | particles | 0,260 | 0,063 | 0,204 | -0,181 |
|  |  | lipid | 0,255 | 0,060 | 0,199 | -0,189 |
|  |  | phospholipid | 0,256 | 0,047 | 0,196 | -0,112 |
|  |  | cholesterol | 0,243 | 0,050 | 0,186 | -0,219 |
|  |  | cholesteryl ester | 0,243 | 0,052 | 0,187 | -0,223 |
|  |  | free cholesterol | 0,239 | 0,039 | 0,182 | -0,195 |
|  |  | triglyceride | 0,251 | 0,185 | 0,249 | -0,063 |
| HDL | XL | particles | 0,016 | -0,041 | 0,008 | -0,276 |
|  |  | lipid | 0,016 | -0,038 | 0,008 | -0,259 |
|  |  | phospholipid | -0,010 | -0,054 | -0,015 | -0,226 |
|  |  | cholesterol | 0,032 | -0,026 | 0,023 | -0,291 |
|  |  | cholesteryl ester | 0,037 | -0,018 | 0,029 | -0,318 |
|  |  | free cholesterol | 0,019 | -0,046 | 0,007 | -0,236 |
|  |  | triglyceride | 0,159 | 0,039 | 0,135 | -0,327 |
|  | L | particles | -0,021 | -0,078 | -0,029 | -0,366 |
|  |  | lipid | -0,030 | -0,072 | -0,034 | -0,369 |
|  |  | phospholipid | -0,012 | -0,071 | -0,021 | -0,400 |
|  |  | cholesterol | -0,048 | -0,088 | -0,053 | -0,313 |
|  |  | cholesteryl ester | -0,048 | -0,087 | -0,052 | -0,320 |
|  |  | free cholesterol | -0,046 | -0,094 | -0,053 | -0,295 |
|  |  | triglyceride | 0,055 | 0,029 | 0,067 | -0,422 |
|  | M | particles | 0,030 | -0,068 | 0,000 | -0,071 |
|  |  | lipid | 0,022 | -0,069 | -0,006 | -0,031 |
|  |  | phospholipid | 0,040 | -0,048 | 0,015 | -0,074 |
|  |  | cholesterol | -0,005 | -0,107 | -0,039 | -0,076 |
|  |  | cholesteryl ester | -0,013 | -0,114 | -0,049 | -0,054 |
|  |  | free cholesterol | 0,029 | -0,082 | -0,003 | -0,197 |
|  |  | triglyceride | 0,119 | 0,120 | 0,126 | 0,290 |
|  | S | particles | 0,064 | 0,036 | 0,057 | 0,247 |
|  |  | lipid | 0,050 | 0,036 | 0,047 | 0,307 |
|  |  | phospholipid | -0,020 | 0,010 | -0,013 | 0,335 |
|  |  | cholesterol | 0,110 | 0,012 | 0,082 | 0,044 |
|  |  | cholesteryl ester | 0,128 | 0,010 | 0,093 | -0,011 |
|  |  | free cholesterol | 0,007 | -0,001 | 0,010 | 0,166 |
|  |  | triglyceride | 0,117 | 0,198 | 0,154 | 0,435 |
| VLDL diameter | |  | 0,026 | 0,130 | 0,060 | 0,329 |
| LDL diameter | |  | -0,097 | -0,033 | -0,074 | -0,127 |
| HDL diameter | |  | -0,039 | -0,067 | -0,041 | -0,278 |
| cholesterol | serum total C | | 0,244 | 0,024 | 0,180 | -0,296 |
|  | VLDL C |  | 0,224 | 0,138 | 0,204 | 0,037 |
|  | remnant C | | 0,267 | 0,098 | 0,219 | -0,104 |
|  | LDL C |  | 0,245 | 0,043 | 0,185 | -0,235 |
|  | HDL C |  | 0,010 | -0,091 | -0,016 | -0,299 |
|  | HDL2 C |  | -0,004 | -0,098 | -0,029 | -0,288 |
|  | HDL3 C |  | 0,126 | -0,002 | 0,100 | -0,345 |
|  | esterified C | | 0,240 | 0,019 | 0,175 | -0,292 |
|  | free C |  | 0,252 | 0,048 | 0,196 | -0,296 |
| triglyceride | serum total TG | | 0,153 | 0,161 | 0,164 | 0,184 |
|  | VLDL TG |  | 0,117 | 0,143 | 0,130 | 0,244 |
|  | LDL TG |  | 0,265 | 0,178 | 0,261 | -0,236 |
|  | HDL TG |  | 0,167 | 0,119 | 0,169 | -0,059 |
| total phospohoglycerides | | | 0,201 | 0,030 | 0,159 | -0,341 |
| phosphatidylcholine | |  | 0,199 | 0,022 | 0,155 | -0,402 |
| sphingomyelins | |  | 0,226 | 0,028 | 0,173 | -0,323 |
| total cholines | |  | 0,205 | 0,019 | 0,158 | -0,445 |
| apolipoprotein A-I | |  | 0,108 | -0,071 | 0,058 | -0,325 |
| apolipoprotein B | |  | 0,260 | 0,095 | 0,213 | -0,036 |
| apo-B / apoA-I | |  | 0,206 | 0,132 | 0,183 | 0,135 |
| fatty acids | total FA |  | 0,208 | 0,110 | 0,186 | -0,085 |
|  | unsaturated | | -0,107 | -0,166 | -0,131 | -0,048 |
|  | DHA (docosahexaenoic acid) | | 0,146 | 0,014 | 0,113 | -0,064 |
|  | LA (linoleic acid) | | 0,180 | 0,043 | 0,145 | -0,307 |
|  | omega-3 |  | 0,172 | 0,020 | 0,131 | -0,052 |
|  | omega-6 |  | 0,210 | 0,044 | 0,166 | -0,297 |
|  | PUFA (polyunsatureted) | | 0,213 | 0,043 | 0,168 | -0,267 |
|  | MUFA (monounsaturated) | | 0,189 | 0,151 | 0,187 | -0,005 |
|  | SAFA (saturated) | | 0,202 | 0,108 | 0,182 | -0,008 |
|  | DHA% |  | 0,033 | -0,044 | 0,013 | 0,033 |
|  | LA% |  | -0,125 | -0,120 | -0,127 | -0,275 |
|  | omega-3 % | | 0,075 | -0,047 | 0,037 | 0,128 |
|  | omega-6 % | | -0,139 | -0,155 | -0,150 | -0,228 |
|  | PUFA% |  | -0,108 | -0,162 | -0,131 | -0,179 |
|  | MUFA% |  | 0,104 | 0,208 | 0,143 | 0,085 |
|  | SAFA% |  | 0,058 | 0,037 | 0,053 | 0,234 |
| glucose |  |  | 0,164 | 0,118 | 0,143 | 0,422 |
| lactate |  |  | 0,165 | 0,083 | 0,137 | 0,324 |
| pyruvate |  |  | 0,148 | 0,151 | 0,151 | 0,390 |
| citrate |  |  | 0,152 | 0,132 | 0,143 | 0,063 |
| glycerol |  |  | 0,183 | 0,168 | 0,171 | 0,641 |
| alanine |  |  | 0,121 | 0,104 | 0,119 | -0,080 |
| glutamine |  |  | 0,075 | 0,020 | 0,052 | -0,125 |
| glycine |  |  | 0,072 | -0,011 | 0,036 | 0,052 |
| histidine |  |  | -0,038 | -0,160 | -0,078 | 0,147 |
| isoleucine |  |  | 0,069 | 0,052 | 0,066 | 0,171 |
| leucine |  |  | 0,061 | 0,010 | 0,046 | 0,033 |
| valine |  |  | 0,023 | 0,004 | 0,016 | 0,036 |
| phenylalanine | |  | 0,178 | 0,103 | 0,162 | 0,173 |
| tyrosine |  |  | 0,125 | 0,103 | 0,120 | 0,020 |
| acetate |  |  | 0,058 | -0,020 | 0,036 | 0,048 |
| acetoacetate | |  | 0,086 | 0,011 | 0,068 | 0,149 |
| 3-hydroxybutyrate | |  | 0,107 | -0,016 | 0,074 | 0,029 |
| creatinine |  |  | 0,069 | 0,018 | 0,046 | -0,250 |
| albumin |  |  | 0,021 | -0,138 | -0,037 | -0,078 |
| glycoprotein acetylation | | | 0,144 | 0,120 | 0,139 | 0,252 |
